# Subacute calorie restriction and rapamycin discordantly alter mouse liver proteome homeostasis and reverse aging effects

**DOI:** 10.1111/acel.12317

**Published:** 2015-03-23

**Authors:** Pabalu P Karunadharma, Nathan Basisty, Dao-Fu Dai, Ying A Chiao, Ellen K Quarles, Edward J Hsieh, David Crispin, Jason H Bielas, Nolan G Ericson, Richard P Beyer, Vivian L MacKay, Michael J MacCoss, Peter S Rabinovitch

**Affiliations:** 1 Department of Pathology, University of WashingtonSeattle, WA, 98195, USA; 2 Department of Genome Sciences, University of WashingtonSeattle, WA, 98195, USA; 3 Human Biology Division, Fred Hutchinson Cancer Research CenterSeattle, WA, 98109, USA; 4 Public Health Sciences Division, Fred Hutchinson Cancer Research CenterSeattle, WA, 98109, USA; 5 Department of Environmental Health and Biostatistics, University of WashingtonSeattle, WA, 98195, USA; 6 Department of Biochemistry, University of WashingtonSeattle, WA, 98195, USA

**Keywords:** aging, calorie restriction, liver, mammalian target of rapamycin, protein turnover, rapamycin

## Abstract

Calorie restriction (CR) and rapamycin (RP) extend lifespan and improve health across model organisms. Both treatments inhibit mammalian target of rapamycin (mTOR) signaling, a conserved longevity pathway and a key regulator of protein homeostasis, yet their effects on proteome homeostasis are relatively unknown. To comprehensively study the effects of aging, CR, and RP on protein homeostasis, we performed the first simultaneous measurement of mRNA translation, protein turnover, and abundance in livers of young (3 month) and old (25 month) mice subjected to 10-week RP or 40% CR. Protein abundance and turnover were measured *in vivo* using ^2^H_3_–leucine heavy isotope labeling followed by LC-MS/MS, and translation was assessed by polysome profiling. We observed 35–60% increased protein half-lives after CR and 15% increased half-lives after RP compared to age-matched controls. Surprisingly, the effects of RP and CR on protein turnover and abundance differed greatly between canonical pathways, with opposite effects in mitochondrial (mt) dysfunction and eIF2 signaling pathways. CR most closely recapitulated the young phenotype in the top pathways. Polysome profiles indicated that CR reduced polysome loading while RP increased polysome loading in young and old mice, suggesting distinct mechanisms of reduced protein synthesis. CR and RP both attenuated protein oxidative damage. Our findings collectively suggest that CR and RP extend lifespan in part through the reduction of protein synthetic burden and damage and a concomitant increase in protein quality. However, these results challenge the notion that RP is a faithful CR mimetic and highlight mechanistic differences between the two interventions.

## Introduction

Protein homeostasis is the equilibrium between protein synthesis, maintenance, and degradation. The dysregulation of this dynamic process has been implicated in aging and age-related diseases (Finkel & Holbrook, 2000; Ryazanov & Nefsky, 2002). Converging evidence indicates that aging is accompanied by an increase in damaged proteins (Hipkiss, 2006) caused by misfolding, translation errors, and post-translational modifications such as oxidation, as well as a decline in damage clearance pathways such as autophagy and proteasomal degradation (Chondrogianni & Gonos, 2005).

Calorie restriction (CR) is the best characterized and most effective intervention known to extend lifespan across a spectrum of species (Stanfel *et al*., 2009), with up to 40% extension in mice (Blackwell *et al*., 1995). In addition to increasing longevity, CR is capable of decreasing morbidity by retarding the occurrence of many chronic diseases such as cancer and diabetes (Colman *et al*., 2009). The mammalian target of rapamycin (mTOR) pathway is widely regarded to be a central modulator of nutrient sensing and growth, which is known to adjust metabolism, increase autophagy, and reduce protein synthesis during CR (Johnson *et al*., 2013). In 2009, Harrison *et al*. reported increased longevity in mice fed with rapamycin (RP), a macrolide antibiotic that inhibits the mTOR kinase (Harrison *et al*., 2009). While the magnitude of longevity extension by RP at doses tested so far is only ∼8–17%, considerably less than that of CR, RP has also been reported to improve functional (‘healthspan’) characteristics of aging mice (Miller *et al*., 2011; Wilkinson *et al*., 2012). While mTOR inhibition is a central theme in the research of CR and RP, decreased protein synthesis and increased autophagy are together not a sustainable cellular state, suggesting that cells may assume a new state of homeostatic balance over the course of treatment, and this is in need of further study.

In this report, we examine protein turnover, abundance, and synthesis changes following subacute (10 week) CR and RP in young and old mice. Protein turnover was measured *in vivo* using LC-MS/MS and calculated using Topograph, a software that deconvolutes isotopologue distributions and calculates amino acid precursor pool enrichment levels; this permits the accurate determination of the proportion of newly synthesized protein (Hsieh *et al*., 2012). To further investigate mRNA translation, we examined ribosome loading by polysome profiling. These methods revealed the effects of the two treatments on liver protein dynamics and proteome remodeling.

## Results

### Liver total and mitochondrial proteome dynamics with aging and short-term CR and RP

**Figure 1:**
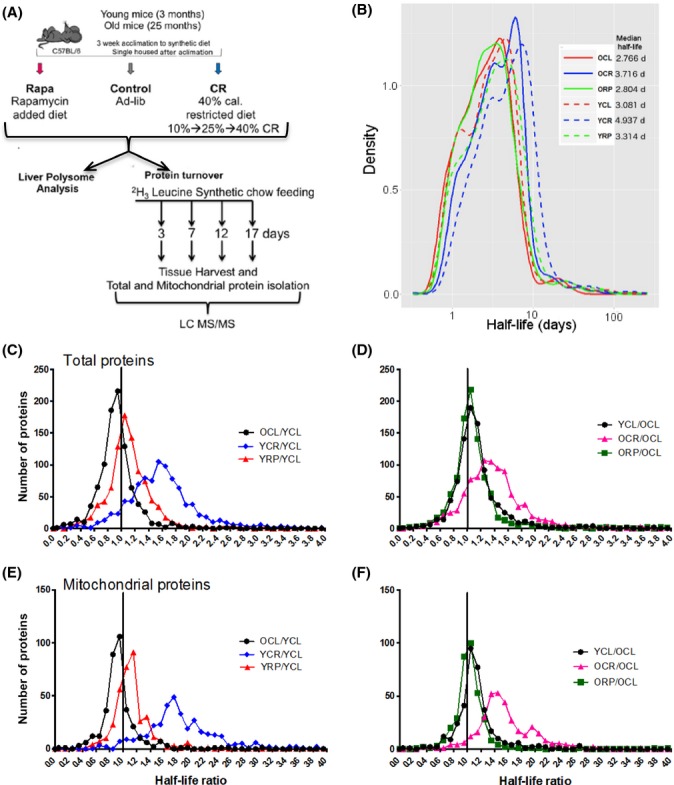
Subacute treatment of CR and RP prolong liver protein half-lives. (A) Summary of experimental design showing young (3 months at the start of treatment) and old (25 mo at the start of treatment) C57BL/6 female mice started on an *ad libitum*, encapsulated RP (14 ppm)-containing or CR (40% restricted) diet for 10 weeks. Livers were harvested and frozen for polysome analyses from five mice per cohort at the conclusion of 10 weeks, and the rest were switched to the same diets except that leucine was replaced with ^3^H_2–_leucine. Mice were euthanized 3, 7, 12, and 17 days post-^3^H_2–_leucine diet feeding. Liver tissue was harvested and LC-MS/MS performed on tryptic peptides of total and mt proteins. Topograph software enabled the calculation of protein half-lives. (B) Protein half-life density plot illustrates the distribution of half-lives of young (dashed) and old (solid) control (CL), calorie restricted (CR), and rapamycin (RP) cohorts. Median half-life in days is indicated on the right of the legend. Protein half-life ratio histograms for two group comparisons: (C,D) total protein half-lives as ratio to those same proteins in YCL (C) or OCL (D). (E,F) Mt protein half-lives as a ratio to YCL (E) and OCL (F). All comparison mean ratios were significantly different from 1.0 with a *P* < 0.01 by z-test for proportions. OCL/YCL < 1.0, *P* < 0.001, and all others > 1.0 *P* < 0.01.

To investigate the effects of CR and RP on hepatic protein turnover rates *in vivo*, we performed stable isotope metabolic labeling in mice with a synthetic diet containing ^3^H_2_–leucine (Fig.1A). CR was a 40% reduction in total calories introduced over 3 weeks, and RP was delivered using the dose (14 ppm) and formulation previously shown to extend mouse lifespan (Harrison *et al*., 2009) and healthspan (Miller *et al*., 2011). Both CR and RP are known to inhibit mTORC1 (Johnson *et al*., 2013), which is suspected to mediate much of their longevity and healthspan benefits. Immunoblotting showed that CR and RP both substantially decreased S6 ribosomal protein phosphorylation in both young and old livers (Fig. S1A,C), but only RP significantly increased eEF2 phosphorylation (Fig.4C,D) and only young RP showed a significant reduction in phosphorylation of 4ebp1 (Fig. S1B). The absence of a chronic effect of RP on 4ebp1 phosphorylation has been previously reported (Choo *et al*., 2008).

Tissues harvested over time were analyzed by shotgun nLC/MS-MS followed by protein turnover and abundance measurements using the software topograph (Hsieh *et al*., 2012). We identified an average of 3110 peptides per sample in total lysates that mapped uniquely to one of 950 proteins and an average of 4001 peptides in purified mt fractions that uniquely mapped to 750 proteins. At each of four time points following the switch to heavy-labeled diet, the percent of newly synthesized protein was determined. The rate of synthesis followed first-order kinetics (Fig. S2A), and the turnover rate constant was calculated (Hsieh *et al*., 2012). Old mouse weights were essentially constant during the time period of heavy labeling and young mouse weights varied by less than 1% per week (Fig. S3), suggesting a condition of steady state where protein synthesis and degradation rates would be approximately equal. Furthermore, the abundance of all peptides identified did not change significantly over the labeling period (Fig. S4, regression slopes centered over zero), also confirming that proteins were at steady state. We therefore refer to the half-time of appearance of newly synthesized protein as the protein half-life (HL).

Protein HLs were seen to have a broad distribution, ranging over at least two log_10_ orders of magnitude, as shown in Fig.1B. While differences between groups are evident in these histograms, a more sensitive analysis is to compare HLs of the same protein in two groups (pairwise analysis of HL ratios, Fig.1C–F). Total proteome median HLs decreased from 3.1 (YCL) to 2.8 (OCL) days and the mean ratio of OCL/YCL is significantly less than 1.0 by ∼20% (*P* < 0.0001, Fig.1C), suggesting a small overall increase in hepatic protein turnover rates with age. CR induced an increase in both total and mt fraction protein HLs in both young (Fig.1C,E) and old (Fig.1D,F) livers. CR significantly increased young total proteome HLs by ∼60% (to median of 4.9 days, *P* < 0.0001, Fig.1B,C) and old HLs by ∼44% (to 3.7 days, *P* < 0.0001, Fig.1B,D) compared to their respective controls. Rapamycin treatment increased total proteome HLs modestly but significantly by 15% for young mice (to 3.3 days, *P* < 0.0001, Fig.1B,C) and 15% for old mice (2.8 days, *P* < 0.0001, Fig.1B,D).

HLs of mt proteins from purified mitochondria behaved similarly to total proteins, with median HLs decreasing significantly with age, from median 3.5 (YCL) to 3.3 (OCL) days (*P* < 0.0001, Fig.1 E). For proteins in common between total and mt extracts, the protein turnover rates were in high agreement (Fig. S5, Spearman’s ρ > 0.65 with *P* < 0.001 for all slope comparisons), demonstrating the high reproducibility of the turnover rate measurements. Mt protein turnover was altered to a greater extent with CR than was that of total proteins, increasing HLs by ∼85% to 6.4 days (*P* < 0.0001, Fig.1E) in YCR and by 59% to 5.0 days in OCR (*P* < 0.0001, Fig.1F). RP increased young mt protein HLs by 12% (3.9 days, *P* < 0.0026) and old HLs by 7% (3.4 days, *P* < 0.0001, Fig.1 E, F). These changes indicate that the CR effect on protein HLs is considerably larger than that of RP at the 14 ppm dose and that the mt proteins have preferentially slower synthesis (longer HLs) after CR.

### Shortest and longest lived proteins, canonical pathways, and subcellular locations

**Figure 2:**
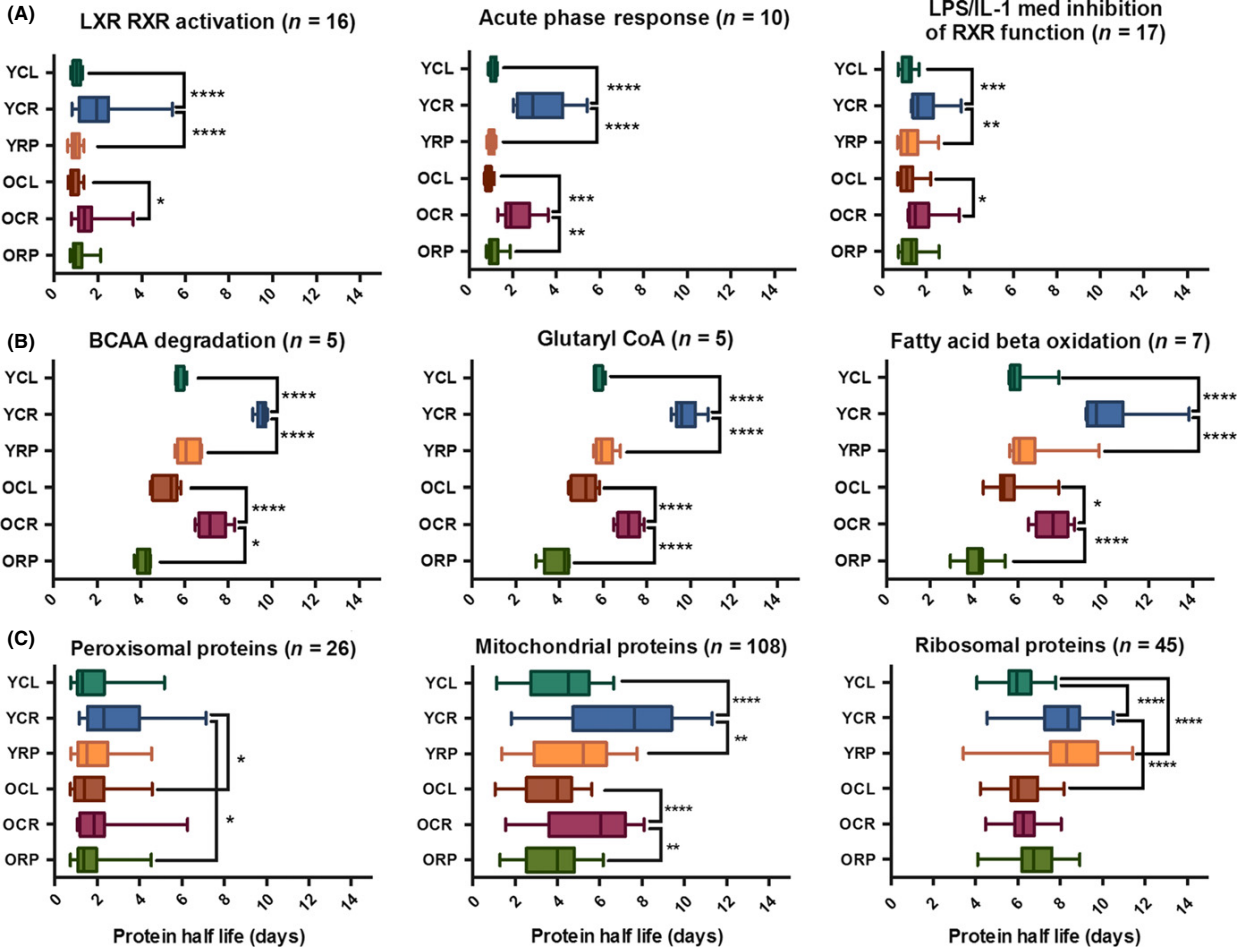
Box plots of liver protein half-lives of CR and RP by pathway and location. (A) Three representative IPA canonical pathways that are among the top fastest turnover (shortest half-lives). Boxes represent the interquartile range for protein HLs, and whiskers extend from 5 to 95% of the data. (B) Three representative canonical pathways with the longest living proteins. Most pathways were metabolic indicating less turnover and higher stability of the metabolic proteins. BCAA, branched-chain amino acid degradation. Old CR extended median HLs of all shortest and longest living pathways except the eIF2 signaling pathway that contains a majority of ribosomal proteins (ribosomal protein HLs shown in panel 2C). (C) Protein HLs by cellular compartment. Peroxisomal proteins were shortest lived, while ribosomal proteins were longest lived. Statistical significance tested with one-way anova, *P < 0.05, **P < 0.01, ***P < 0.001, and ****P < 0.0001. Numerical detail and HLs for these pathways are in Table S1 (Supporting infomation).

The top 20% of shortest and longest living proteins in the YCL cohort were analyzed for enrichment of canonical pathways, as defined by Ingenuity Pathway Analysis (IPA, Tables S1A,B). The shortest lived protein pathways (Table S1A) had mean HLs of 0.894 to 1.524 days in YCL livers and were predominantly signaling pathways, including LXR/RXR activation, acute phase response signaling, and LPS/IL-1-mediated inhibition of RXR function; these three examples are illustrated in Fig.2A. CR extended the mean half-life of a majority of the short-lived protein pathways significantly (Table S1A). Even though YRP increased the mean half-life of most pathways, none of these changes was statistically significant (Table S1A, Fig.2A).

The protein pathways with the longest HLs are shown in Table S1B, ordered based on the YCL average half-life, which ranged from 5.55 to 11.89 days. The longest lived proteins belonged to amino acid metabolism, intermediary metabolism, and energy (ketolysis, acetyl-CoA biosynthesis, fatty acid beta oxidation) pathways. Pathways of these three categories are shown in Fig.2B. The average half-life of a majority of these pathways was significantly extended by CR in old mice (*P* < 0.05, Table S1B).

We also compared protein HLs in several cellular compartments as shown in Table S1C (Supporting information) and Fig.2C. Of these, peroxisomal proteins had the shortest HLs (ranging from 1.8 to 3 days) and ribosomal proteins had the longest HLs (ranging from 6 to 8.4 days) that were extended by ∼40% in young RP-treated mice (*P* < 0.0001). RP did not have nearly as large nor as significant an effect on ribosomal protein half-lives in old mice (Fig.2C). Mt proteins were the second longest lived category after ribosomal proteins and the only cellular compartment to have a significant increase in HLs with CR in both young and old groups (Table S1C, and Fig.2C).

### Pathway analysis of significant changes in hepatic proteome turnover

**Figure 3:**
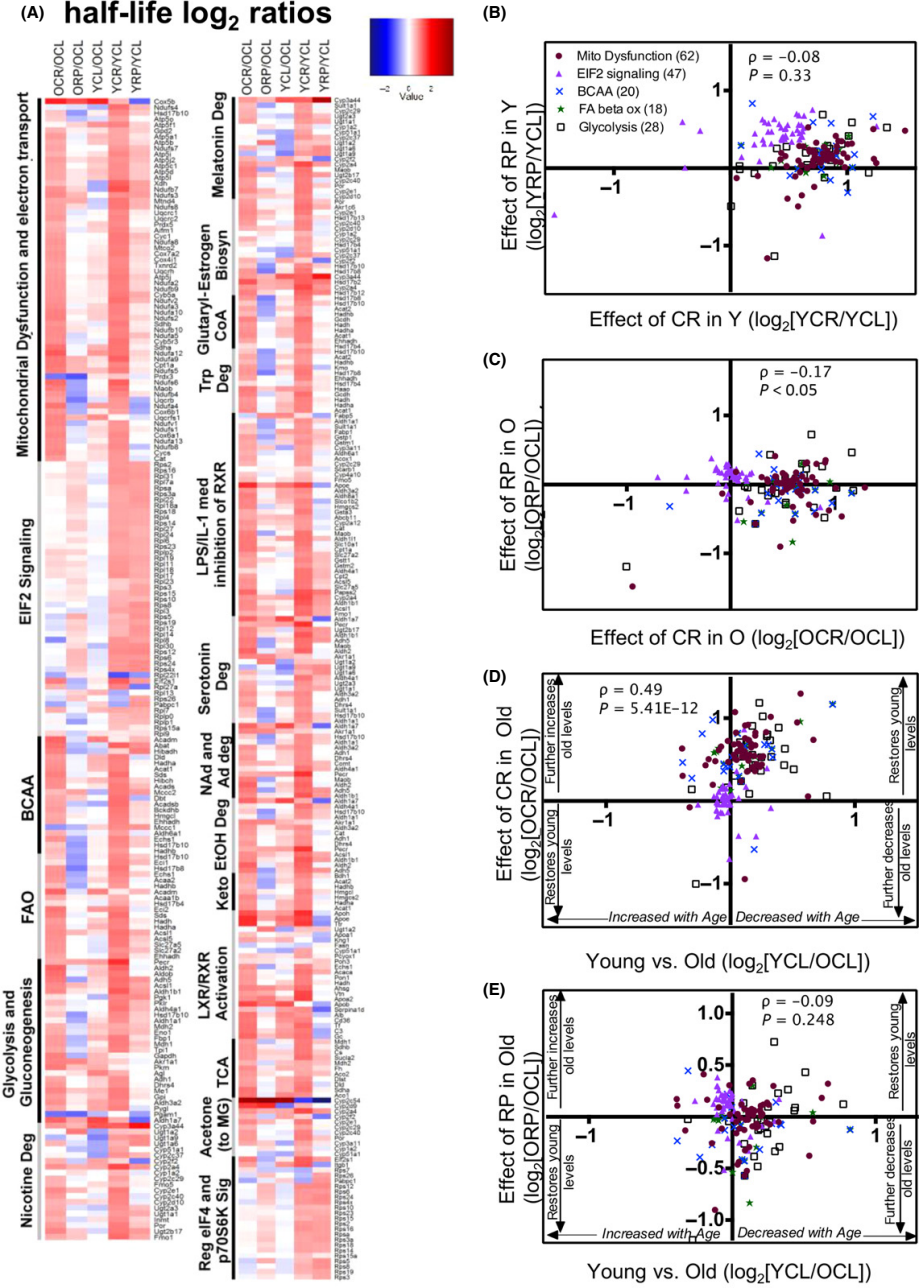
Heatmap of protein half-life differences and scatter plots of top pathway HL ratio comparisons. (A) Total proteins that were significantly altered (*q* < 0.05) were categorized into the top 19 canonical pathways using IPA. Red indicates longer half-life in the numerator, and blue indicates longer half-life in the denominator. Ratio values ranging from log_2_ -3 to +4 are depicted in a color gradient from blue to red. BCAA, branched-chain amino acid degradation; FAO, fatty acid beta oxidation; Keto, ketogenesis; and acetone (to MG), acetone degradation to methylglyoxal. (B–E) Scatter plots of log_2_ ratio comparisons of top metabolic pathways for (B) YRP/YCL vs. YCR/YCL, (C) ORP/OCL vs. OCR/OCL, (D) OCR/OCL vs. YCL/OCL, and (E) ORP/OCL vs. YCL/OCL. (D,E) If changes with aging are reversed, those proteins are located in the top right or bottom left quadrants of the scatter plots. The number of significantly changed proteins that mapped to each pathway is listed in parenthesis in panel (B). All heatmap proteins and their half-life log_2_ ratios are listed in Table S2 (Supporting information). Values and summary statistics for all other proteins, in addition to these, are in Table S4 (Supporting information).

To identify the pathways differentially affected by CR and RP, we categorized proteins whose half-lives were significantly altered (*q* < 0.05) by age or one or more intervention and assigned those to canonical pathways using IPA. The top pathways most significantly altered by the treatments are shown in a heatmap (Fig.3A).

Of the significantly changed protein HLs in the liver proteome, the majority (81%) belonged to metabolic pathways, including energy, amino acid, hormone, and intermediary metabolism. Other functional groups included signaling, transcriptional regulation (i.e., PXR/RXR activation), and biosynthetic pathways. Mitochondrial dysfunction (defined in the supplementary methods) is the top pathway significantly altered by the 10-week intervention. As noted above, HLs from total and mt fractions were highly concordant, and the mt fraction HL heatmap (Fig. S6) is very similar to the total protein heatmap.

In almost all pathways and both ages, CR globally extended protein HLs (decreased turnover) compared to controls, as demonstrated by consistent red colors (log_2_ ratios>0) of the CR/CL ratios in the total (Fig.3A) and mt protein (Fig. S6) heatmaps. The only exceptions to this phenotype are eIF2 signaling and regulation of eIF4 and p70S6K signaling pathways (Fig.3A) where young, but not old HLs were increased by CR compared to controls. Notably, these are the pathways most directly downstream of mTORC1 and include many large and small ribosomal proteins. Although fewer than 12% of the total proteins included in the heatmap had HLs that were shorter in OCR than OCL (blue colors in Fig.3A), 45% of all observed ribosomal proteins in old livers had shorter HLs after CR. RP treatment increased mean HLs 5–15% in nine of 19 pathways in young and old groups.

It is interesting to note that despite the similarities in lifespan and healthspan benefits of CR and RP, the effects on hepatic proteome dynamics are dramatically different between the two treatments. In young livers, the magnitude of the change induced by RP is about 40% smaller than CR (Figs.1C and 3B), and in old livers, the direction of change was often opposite to that of CR (Fig.3C; negative Spearman’s ρ = −0.17, *P* < 0.0505). Moreover, whereas CR in old liver lysates had an effect that was generally toward making the HLs more similar to those of young livers (Fig.3D, significant positive correlation shown by Spearman’s ρ = 0.5, *P* < 0.0001), RP produced effects that were highly variable, but often in an opposite direction to that of young control livers (Fig.3E, many proteins in the lower right and upper left quadrants). It is also apparent that while RP treatment in young and old mice visually resembles one another and correlate well in the heatmap (3A), the magnitude of the changes induced by RP in old mice is smaller than the response from young mice. Consistent with this, blood levels of rapamycin (Fig. S11) were found to be lower in old mice compared with the young cohort. Thus, diminished bioavailability of the drug in old mice likely explains part or all of the difference.

### Protein quality control: altered protein carbonylation, autophagy, and translational elongation by CR and RP

**Figure 4:**
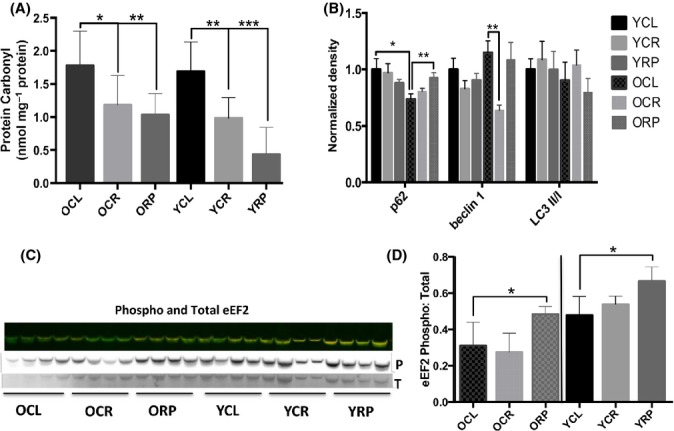
Protein carbonyl content, autophagic degradation, and translation elongation marker with treatment and aging. (A) Protein carbonyls are decreased with treatments. (B) Autophagic marker p62 show a decrease with aging and an increase with ORP, while beclin-1 is decreased with OCR. LC3 II/I ratios did not change with age or treatments. (C) Representative fluorescent Western blot of phospho-eEF2 and total eEF2. Phosphorylation of this elongation factor disrupts translational elongation. The ratio of phospho-eEF2 over total eEF2 is quantified in (D). Rapamycin treatment significantly increased this ratio in both young and old mice. *n* = 4–6 per group., *P < 0.05,** *P* < 0.01, ***P < 0.001.

Protein carbonylation can be used as a readout for oxidative stress and damage. We observed a significant decrease in protein carbonylation with CR and RP in both young and old animals after 10 weeks of treatment (Fig.4A). Autophagy is a major proteolytic system involved in protein quality control and degradation that is regulated by mTORC1. To examine the effects on autophagy, we measured the content of several autophagy markers with age and treatments. A significant decrease in p62 with aging was reversed by ORP (*P* < 0.01, Fig.4B). Beclin-1 levels decreased with OCR (*P* < 0.01, Fig.4B), and LC3 II/I ratios did not alter with aging or treatments. Collectively, these markers are consistent with a decline in autophagy in old mouse treatment groups, which is also consistent with reduced turnover (longer HLs) associated with OCR and ORP (Fig.1C–F).

### IPA analysis of significant changes in protein abundance

Topograph calculates peptide abundances by chromatographic peak areas under the curve (AUC), which includes the sum of the unlabeled and labeled peaks for each peptide. We found no systematic difference in the AUC estimates between samples from early heavy labeling times vs late (Fig. S4A), and the data from all 12 mice in each cohort were used in the analysis. From the 3110 unique peptides in the total protein fraction, protein abundances were calculated and the significantly altered proteins (*q* < 0.05, an average of 200 proteins in each pairwise comparison) were analyzed using IPA to define the canonical pathways affected by CR and RP.

**Figure 5:**
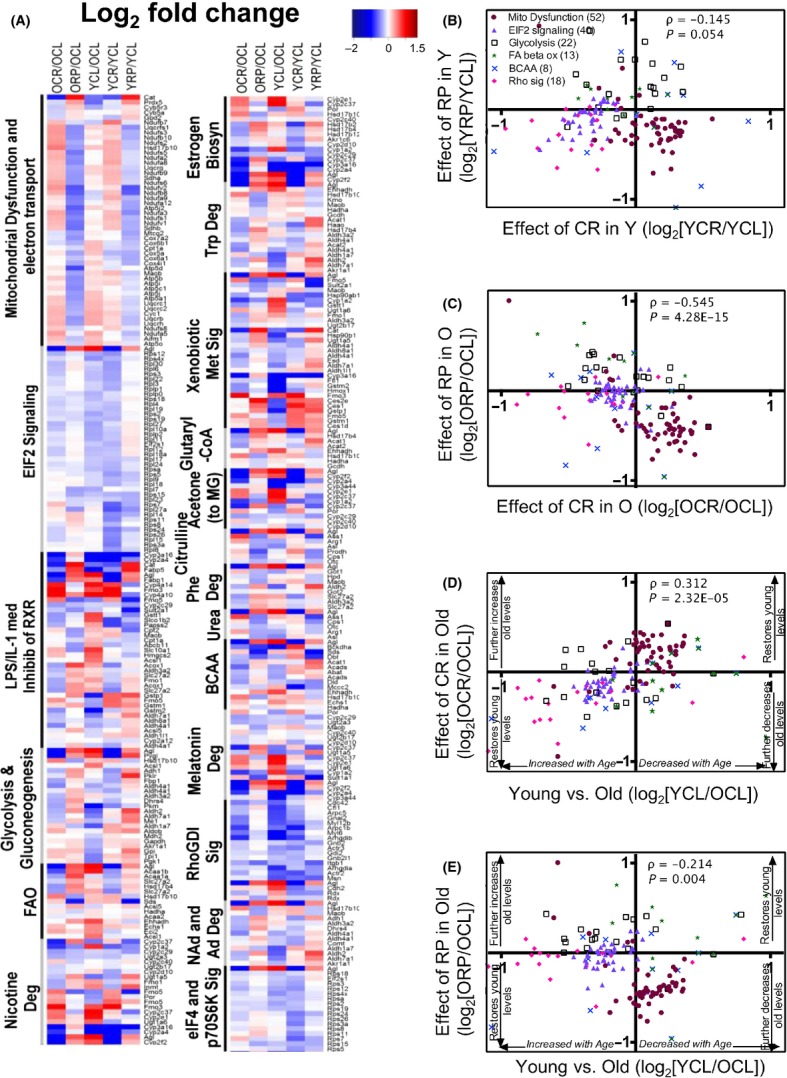
Heatmap of protein abundance differences and scatter plots of top pathway abundance comparisons. (A) Proteins that were significantly altered at q < 0.05 were categorized into canonical pathways using IPA. The top 19 pathways are reported. Log_2_ fold-changes ranging from −2 to +2 are gradually colored from blue to red. (B–E) Scatter plots of log_2_ fold-change comparisons of the top metabolic pathway proteins of (B) YRP/YCL vs. YCR/YCL, (C) ORP/OCL vs. OCR/OCL, (D) OCR/OCL vs. YCL/OCL, and (E) ORP/OCL vs. YCL/OCL are shown. (D,E) If changes with aging are reversed, those proteins are located in the top right or bottom left quadrants of the scatter plots. The number of significantly changed proteins that mapped to each pathway is listed in parenthesis in panel (B). All heatmap proteins and their log_2_ fold-changes are listed in Table S3 (Supporting information).

This analysis revealed many contrasting changes between CR and RP (Fig.5A). The most prominent example of this is the mt dysfunction pathway, where YRP/YCL and ORP/OCL comparisons showed a decrease in relative protein abundances, while YCR/YCL and OCR/OCL showed increases (Fig.5A mt dysfunction pathway, filled circles in Fig.5B,C in the lower right quadrants). Overall, this inverse correlation between CR and RP was stronger in old liver (Fig.5C, Spearman’s ρ = −0.54, *P* < 0.0001) than in young liver (Fig.5B, Spearman’s ρ = −0.14, not significant). This was recapitulated in the assay of aconitase activity, with a similar trend in citrate synthase activity (Fig. S7). These apparent changes in mt protein content and activity by CR vs. RP are not likely to be explained by changes in total mt mass per cell as mtDNA copy number did not alter with treatments (S8A). Nor can they be explained by increased mt biogenesis, as mRNA levels of mt biogenesis markers Nrf1, Nrf2, and mt transcription factor A (TFAM) did not change with the treatments (Fig. S8B–D).

In general, OCR restored protein abundances to young levels (significant positive correlation, Spearman’s ρ = 0.31, *P* < 0.0001, Fig.5D). Conversely, RP reduced most protein abundances (blue colors in ORP/OCL, Fig.5A, significant negative correlation, Spearman’s ρ = −0.21, *P* = 0.004 in Fig5E), amplifying many of the changes that occurred with aging. Notable exceptions to this pattern were eIF2, eIF4 and p70S6K, and Rho GDI signaling pathways. These pathway components were almost all decreased by both CR and RP, and are comprised of many ribosomal and protein translation proteins regulated by mTORC1, as noted above. With the exception of these pathways, the striking difference between CR and RP in old mice appears to create two very different metabolic profiles, suggesting that important parts of their mechanisms of action are distinct.

### Analysis of CR and Rapa effects on protein translation: polysome profiles and eEF2

**Figure 6:**
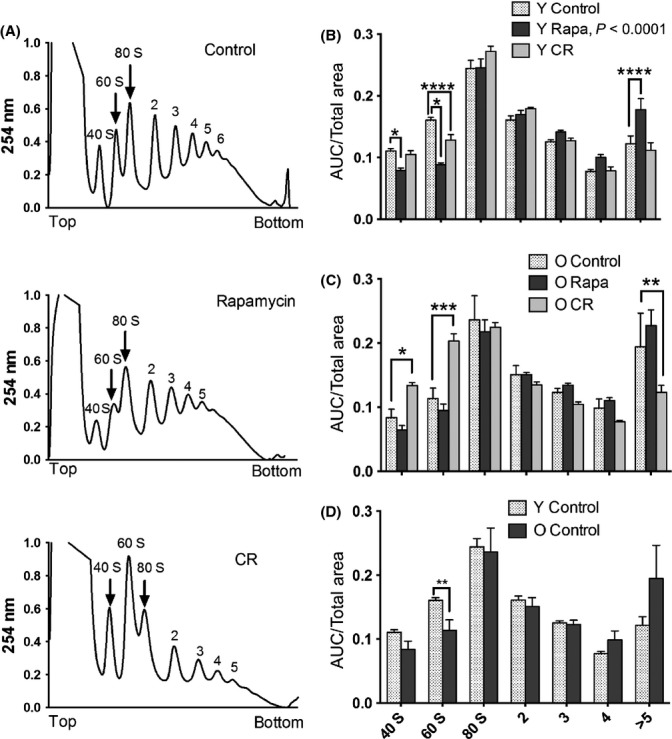
Subacute CR decreases global protein translation while RP increases ribosome loading in the liver. (A) Representative polysome profiles of young control, RP, and CR groups. The ‘top’ and ‘bottom’ of the gradient are indicated on the *x*-axis. The peaks are labeled with the name of the ribosome subunit or number of ribosomes that they represent. (B, C) Area under the curve (AUC) measurements shown relative to the total AUC of the single subunits, monosome, and polysome peaks of control, CR, and RP livers of (B) young and (C) old mice. (D) Quantified relative polysome area of young and old livers. *n* = 5 per group, *P* < 0.05 anova. *P < 0.05, **P < 0.01, ***P < 0.001, and ****P < 0.0001.

As initiation is usually the rate-limiting step of mRNA translation (Gingras *et al*., 2001), we wished to gain insight into changes in global protein translation by examining polysome profiles of CR- and RP-treated livers. Figure6A shows representative profiles of YCL, YCR, and YRP liver polysome gradients. We quantified the areas under the curve of free subunits (40S, 60S), monosome (80S), and polysome peaks (Fig.6A); the ratios of these areas to the total area under the ribosome peaks are plotted in Fig.6B (young livers) and 6C (old livers). As expected, old CR showed a significant increase in free 40S and 60S subunits and a decrease in ribosome loading (5 ribosomes or higher, Fig.6C, *P* < 0.001) compared to control, indicating decreased translation. This was confirmed by a significant linear trend of decreased peak ratios vs. ribosome loading when the areas were analyzed as a percent of control values (Fig. S10B, *P* < 0.01). Surprisingly, young RP livers showed an increase in polysomes consisting of 5 or more ribosomes and a decrease in 40S and 60S subunits (Fig.6B, *P* < 0.0001) and a significant linear trend of increased loading when the peak ratios were analyzed as a percent of control (Fig. S10A, *P* < 0.0001). No significant differences were observed in individual old RP peaks (Fig.6C), but trend analysis showed a significantly increased ribosome recruitment (Fig. S10B, *P* < 0.0001), just as was seen in young RP livers. This is contrary to the expected outcome of decreased translational activity by RP (Beretta *et al*., 1996) and the net increase in protein half-lives seen in RP (Fig.1C–F), but it would be consistent with a reduction in elongation rates that would slow ribosome translocation on messages. In agreement with this, rapamycin significantly increased the proportion of phosphorylated eukaryotic elongation factor 2 (eEF2) in both young and old mice (Fig.4C, D), which is consistent with reduced translation elongation rates (Kaul *et al*., 2011).

The comparison of YCL to OCL polysome profiles (Fig.6D) showed significantly lower levels of 60S ribosomal subunits in OCL; this was confirmed by trend analysis (Fig. S10C), which showed significantly (*P* < 0.001) increased polysome loading in OCL livers compared to YCL. This result is consistent with the overall increase in protein synthesis rates (decreased half-lives) observed with aging (Fig.1C).

## Discussion

**Figure 7:**
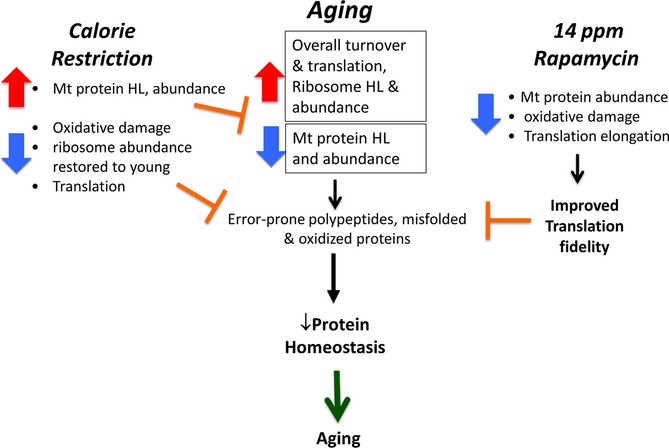
A summary model of the effects observed in this study. With aging, we observed an overall increase in turnover and translation with a concomitant decrease in mt protein HLs and abundance. In combination, these factors can result in increased ROS and the formation of error-prone polypeptides and oxidized proteins, thereby disrupting protein homeostasis. CR reversed these changes effectively recapitulating a more youthful proteome, while RP changes were more subtle but indicated decreased oxidative damage and changes that improved translational fidelity. Short-term CR and RP can both restore protein homeostasis to promote more healthy aging.

In this study, we sought to investigate the hepatic proteome dynamics associated with two well-established longevity interventions, CR and RP, in young and old mice. To measure dynamic changes, we utilized whole-body stable isotope labeling and a new software tool to calculate protein turnover rates and relative protein abundances *in vivo*. While both CR and RP are lifespan-extending interventions with a mechanistic link in the mTOR pathway, our results provide insight into distinct differences (especially in protein abundances), as well as similarities (especially in protein HL changes) after subacute treatment. In particular, the highly opposing effects of these treatments on mt protein abundances (Fig.5A) suggest that these interventions have a different effect on cellular energetics in liver. The effects of RP on protein half-lives, although positively correlated in many pathways, are generally much smaller than those of CR (Fig.1C–F). While this might suggest that the ITP dose of RP is suboptimal as a CR mimetic, it is notable that the effects on altered protein abundance were qualitatively different, rather than just quantitatively different. We would not expect that a higher dose of RP would reverse the *direction* of abundance changes seen in Fig.5A and D. Furthermore, RP was equally effective as CR in slowing turnover of ribosomal proteins in the eIF2, eIF4, and p70S6K pathways in young mice (Fig.3A), in accordance with the hypothesis that RP has a subset of specific overlapping targets with CR that are sufficient to promote health. These data are consistent with a recent study that suggests that 20 weeks of RP treatment inhibited pS6 but not 4EBP1, and thus, there is little inhibitory effect on cap-dependent translation by RP (Fang *et al*., 2013). This is in agreement with our observations (Fig. S1B,C) and another report that RP differentially inhibits S6Ks vs. 4EBP1 (Fang *et al*., 2013). One may therefore speculate that the beneficial effects of RP track less with inhibition of cap-dependent initiation of translation and more closely with inhibition of ribosomal protein synthesis, reducing translation elongation and improving translational fidelity (see summary Fig.7 and discussion below).

Our approach to define proteome dynamics with CR and RP *in vivo* used novel software that is able to deconvolute complex isotopomer distributions and calculate amino acid precursor pool enrichment levels. This is key to accurate calculation of protein turnover rates; other heavy isotope labeling approaches require complex calculations to indirectly estimate and compensate for changes in precursor pool enrichment (Price *et al*., 2010, 2012; Kim *et al*., 2012). With this novel platform, we were able to determine the precursor pool-adjusted turnover rates of over 900 proteins in mouse liver. Previous reports on the effect of CR on protein turnover suggest an increase in protein synthesis and degradation (Lewis *et al*., 1985; Goldspink *et al*., 1987; Merry *et al*., 1987). In contrast, we observed a large global increase in HLs (decreased synthesis) following 10 weeks of CR, a reversal from the slightly lower HLs seen in old control livers (Fig.1C OCL/YCL). Consistent with our data, Price *et al*. reported prolonged HLs in over 80% of liver proteins with chronic CR (Price *et al*., 2012). We believe an important part of this apparent contradiction with previously published turnover data relates to differences between acute, subacute, and chronic CR treatments.

The global decline in protein synthesis rates observed with subacute CR, and to a lesser extent with RP, is consistent with the hypothesis that with longer treatment times, CR is switching cells to an energy conservation state, decreasing the energetically expensive process of protein synthesis. After subacute or chronic treatment, these longer lived proteins may have higher quality and less damage; this is supported by our observation of lower levels of protein carbonyls (Fig.4A) and a recent report that showed increased protein translation fidelity in RP-treated liver MEFs (Conn & Qian, 2013). Consistent with this interpretation, acute RP treatment (1–2 weeks) caused insulin resistance and lower glucose tolerance (Lamming *et al*., 2012), while a longer treatment (20-week) reversed the insulin resistance with better lipid profiles and increased oxygen consumption (Fang *et al*., 2013). Our results are also disparate to two recent reports using metabolic labeling that showed no change in overall protein synthesis in the liver, heart, or skeletal muscle with CR and RP (Drake *et al*., 2013; Miller *et al*., 2013). We and Price *et al*. show substantially reduced global turnover with CR, and we observed a reduction in turnover after RP. A plausible explanation for the difference from Miller *et al*. is that they measured ‘bulk’ protein synthesis that can be dominated by the synthesis rates of a few highly abundant structural proteins, while our and Price’s measurements of large numbers of individual proteins are not skewed by the most abundant proteins.

Whereas there is an abundance of evidence that acute CR and RP treatments increase autophagy (Hansen *et al*., 2008; Alvers *et al*., 2009), our data are contrary to this: LC3-II/I levels are unchanged and beclin-1 only decreased with OCR, while p62 only increased with ORP (Fig.4B). Measurement of autophagic flux will be needed to conclusively determine the status of autophagy; however, this disparity may be due to the majority of prior studies reporting changes after acute CR and RP treatments. In order for protein turnover rates to have decreased after 10 weeks of CR and RP, as we observe, elevated rates of autophagy cannot be maintained.

An unanticipated finding of this study was the increased polysome loading observed with young and old RP treatments (Fig.6B,C; Fig. S10A,B). This might seem to be inconsistent with reduced protein turnover and a previous report of a reduction in cap-dependent translation by RP (Beretta *et al*., 1996). This dichotomy is resolved by noting that RP treatment reduces eEF2 phosphorylation (Fig.4C,D), which will lead to a reduced elongation rate of ribosomes; this can result in increased polysome loading, even if initiation is decreased. This condition has been shown to enhance translation fidelity (Conn & Qian, 2013), keeping proteins in good quality with longer half-lives (Hipkiss, 2007). Thus, our results are consistent with the hypothesis that reduced rates of translational elongation and reduced rates of protein synthesis after 10 weeks of RP facilitate production of error-free polypeptides that are longer lived.

In agreement with our observation of differential mt protein abundance (Fig.5C), Zid and colleagues demonstrated preferential translation of mt proteins after CR in Drosophila (Zid *et al*., 2009) and CR has also been shown to increase mt gene expression and respiratory capacity in multiple murine tissues (Dani *et al*., 2010; Cerqueira *et al*., 2012). In contrast, acute RP has been shown to suppress mt biogenesis (Cunningham *et al*., 2007) and decrease mt respiration in cultured cells (Schieke *et al*., 2006; Ramanathan & Schreiber, 2009). Surprisingly, carbonylation, an indication of oxidative protein damage, was significantly lower in RP compared to CR in both young and older livers (Fig.4A). We believe this is best explained by our observation of differential mt respiratory dynamics, as indicated by increased protein abundances (Fig.5A) and higher levels of aconitase activity in CR compared to RP (Fig. S7A). Increased mt respiration with CR may be producing more carbonyl-inducing ROS, while decreased mt activity in RP results in less carbonylation. Interestingly, these differences in mt occurred without a change in mtDNA copy number and mt biogenesis (Fig. S8). The 10-week treatments also made no impact on the elevated mitochondrial deletion frequency seen in old mice (Fig. S9).

Consistent with differences we observe in treatments, two recent reports have highlighted the distinctive effects of RP and CR in the liver after a 6-month treatment (Fok *et al*., 2014; Yu *et al*., 2014). CR had a larger and mostly opposite effect on the transcriptome compared to RP. Genes associated with mt function and antioxidants were among the top upregulated with CR, while most transcripts changed with RP were downregulated. No significant change was observed in the liver metabolome by RP, but CR had a larger effect changing metabolites associated with energy status. Compared to CR, RP showed no protective effect on fat mass, insulin sensitivity, redox status, or the fatty acid oxidation in liver. These results track with our observations of distinctive differences in mt protein abundance (Fig.5A,B), polysome loading (Fig. S10 A,B), and ∼40% lower effect on protein half-lives (Fig.3A,C) by RP compared to CR.

A cautionary note is that the effects of CR and RP on the proteome appear to be different depending on the tissue examined. We have recently reported that short-term CR and RP in the heart produced similar proteome effects, both treatments restoring proteins involved in mitochondrial function, electron transport chain, fatty acid oxidation, and TCA cycle into youthful levels. Furthermore, both treatments reversed age-dependent cardiac hypertrophy and diastolic dysfunction to the same extent (Dai *et al*., 2014). The magnitude of change in protein HLs by OCR in the heart was similar to liver (∼45% longer than OCL), but ORP induced a bigger change in the heart (27% longer compared to only 15% in the liver). Overall, the changes in proteome turnover and abundance were highly similar in both treatments, including effects on mt proteins, in contrast to our observations in the liver. Therefore, these treatments, especially RP, appear to have much different effects on the proteome and physiology depending on the tissue type, with heart and liver perhaps lying at extremes, considering their disparate function and energetics.

In summary, we investigated the effect of CR and RP in parallel on young and aged mouse protein turnover, abundance, and polysome loading. Our findings provide compelling evidence supporting the idea that CR’s beneficial effect on lifespan is mediated to a large extent by a restoration of a more youthful proteome: A majority of the metabolic pathways were significantly altered by aging, and this was reversed in OCR. The effects of RP in liver are complex and, in many cases, dissimilar to those of CR, but nevertheless indicate a large impact on proteome dynamics. Ribosomal and mt proteins in particular behaved very differently in OCR and ORP (Fig.3A,C-E, Fig.5A,C-E). Increased HLs after RP and decreased relative protein abundance in many key metabolic pathways in old mice, coupled with increased polysome loading (most likely due to a reduction in elongation rate), suggest a slowing of protein synthesis with production of higher quality, longer lived proteins after RP. Overall, our data provide the first comprehensive analysis of proteome dynamics with aging and subacute CR, and RP and underline a functional connection between protein homeostasis and longevity.

## Experimental procedures

Methods in detail are included in the supplement.

### Animals

C57BL/6 female mice were purchased at 3 and 25 months of age (Fig.1A) from the NIA Charles River colony. Female mice were used as a past study showed that the effects of RP in murine aging were larger in this gender (Harrison *et al*., 2009). Mice were housed at 20 °C with a 12-h light and dark cycle. All animals were handled according to the guidelines of the Institutional Animal Care Committee of the University of Washington and the National Institutes of Health. One week after arrival, all mice were started on a synthetic diet (Harlan Teklad diet #TD.99366) that was nutritionally similar to the NIH-31 standard for rodents. The use of this diet facilitated the subsequent substitution of heavy-labeled [5,5,5 – ^2^H_3_] leucine for light leucine, which enabled the protein turnover measurements. Mouse weights and food intake were recorded weekly. The young and old mice were individually housed after 3 weeks of acclimation to the synthetic chow and were randomly assigned to three groups: 1) an *ad libitum* synthetic food regimen (Control); 2) rapamycin-containing synthetic diet (RP); and 3) calorie restricted (CR), as detailed in the supplemental methods.

### Stable Isotope labeling

After 10 weeks of diet regimens, all mice received a synthetic diet (TD.09846, Harlan Teklad, Madison, WI, USA) with the light leucine fully replaced by 11 g/kg of deuterated [5,5,5 – ^2^H_3_] – L – leucine (Cambridge Isotope Laboratory, Tewksbury, MA, USA), with CR and RP cohort conditions continued as above. Three mice were euthanized for tissue collections and proteomics analysis at four time points: days 3, 7, 12, and 17 after switching to ^2^H_3_–leucine diet.

### Mass spectrometry and analysis

Whole liver tissue was homogenized, or mt fractions were isolated as previously described (Zhang *et al*., 2008). Both were processed, trypsin digested, and LC-MS/MS analysis performed with a Waters nanoAcquity UPLC and a Thermo Scientific LTQ Orbitrap Velos, as previously described (Hsieh *et al*., 2012). The raw data from MS/MS and extended supplementary files are available at https://chorusproject.org/pages/blog.html#/351.

The topograph software program (http://proteome.gs.washington.edu/software/topograph/)(http://proteome.gs.washington.edu/software/topograph/) was developed for the measurement of peptide isotopologue abundances from LC-MS/MS chromatograms and the calculation of peptide turnover rates (Hsieh *et al*., 2012). It allows the measurement of the proportion of the amino acid precursor pool that is labeled, which varied over time and condition (Fig. S12A). This information allows the correct calculation of percent of new synthesis for each peptide, which when plotted for 12 biological replicates over time (four time points) generated an exponential curve following first-order kinetics (Fig. S2A). Using a logarithmic transformation, the first-order protein turnover rate (slope) was determined by linear regression (Fig. S2B). Only peptides that uniquely mapped to a single protein were used for our measurements (see supplemental methods for details).

## Supporting Information

**Fig. S1** Immunoblot analysis of mTOR downstream targets. **Fig. S2** Topograph calculation of protein turnover rate and peak alignment for isotopomer abundance. **Fig. S3** Body weights of young (A) and old (B) mouse cohorts over the course of the experiment. **Fig. S4** Linear regression fits of peptide abundance (peptide isotopomer area) over the labeling period indicate no significant change in abundance. **Fig. S5** Liver total and mitochondrial protein turnover slope correlation. **Fig. S6** Heatmap of mitochondrial protein half-life log2 ratios. **Fig. S7** Mitochondrial aconitase and citrate synthase activity in liver. **Fig. S8** The ratios of mitochondrial DNA-to-nuclear DNA (mtDNA/nDNA) did not alter with treatment. **Fig. S9** Mitochondrial deletion frequency in mouse liver. **Fig. S10** Global protein translation increased with aging and linear trendanalysis of CR and RP effects on polysome loading. **Fig. S11** Rapamycin blood level measurements from n=11-17 mice from the two age groups. **Fig. S12** Precursor pool enrichment of 3H-leucine over time and days until initial appearance of label. **Fig. S13** Relative standard error of the half-life estimates in Liver and Heart.

**Table S1** (A) Shortest living pathways. (B) Longest living pathways. (C) Cellular compartment half-lives **Table S2** Figure 3A heatmap proteins and corresponding log2 ratios. **Table S3** Figure 5A heatmap proteins and corresponding log2 fold changes. **Table S4** Turnover rate constants and summary statistics for all proteins detected by mass spec in each group.
