# Supplementary figures and images for: Subacute calorie restriction and rapamycin discordantly alter mouse liver proteome homeostasis and reverse aging effects
Source: Aging Cell. 2015 Mar 23;14(4):547–57. doi: 10.1111/acel.12317 (PMC4531069; doi:10.1111/acel.12317)

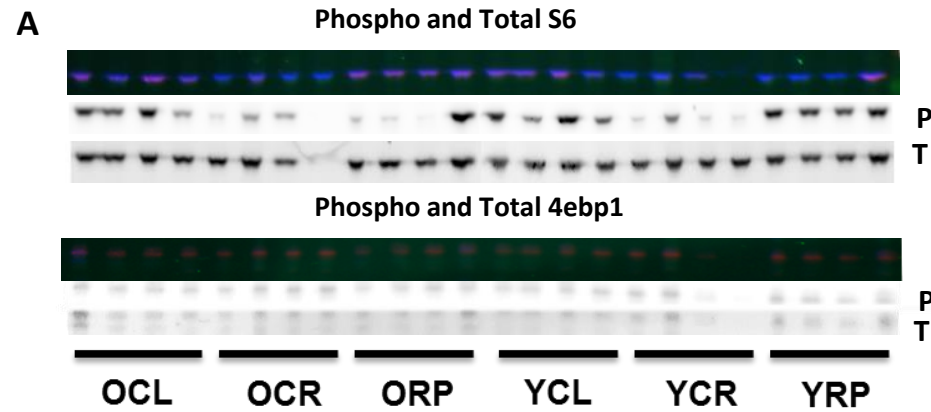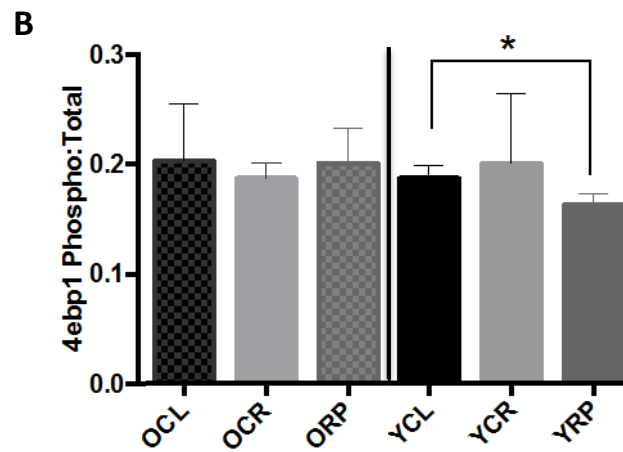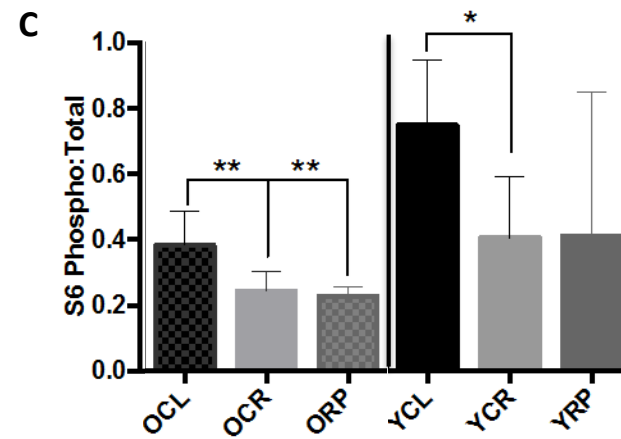

OCL vs OCR, Q8C196nProts=1, nPeps=48, Signif=6.84e-25

A

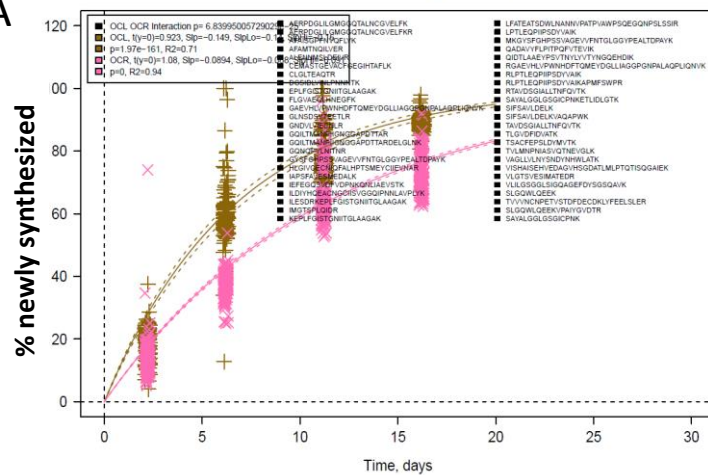

B

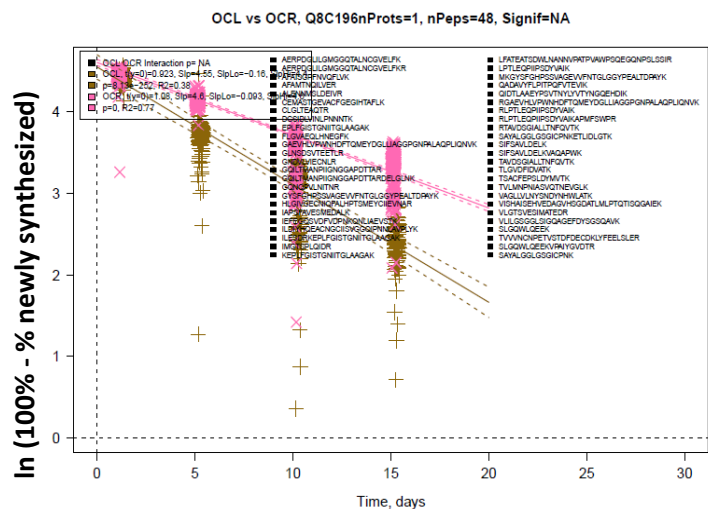

C

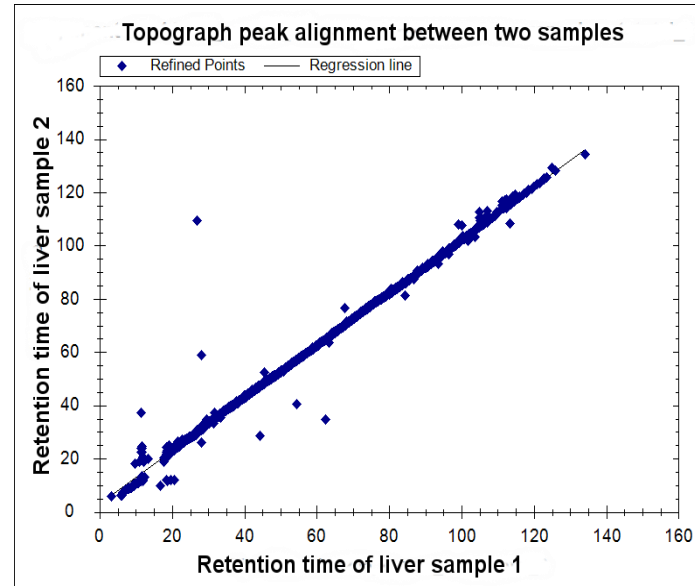

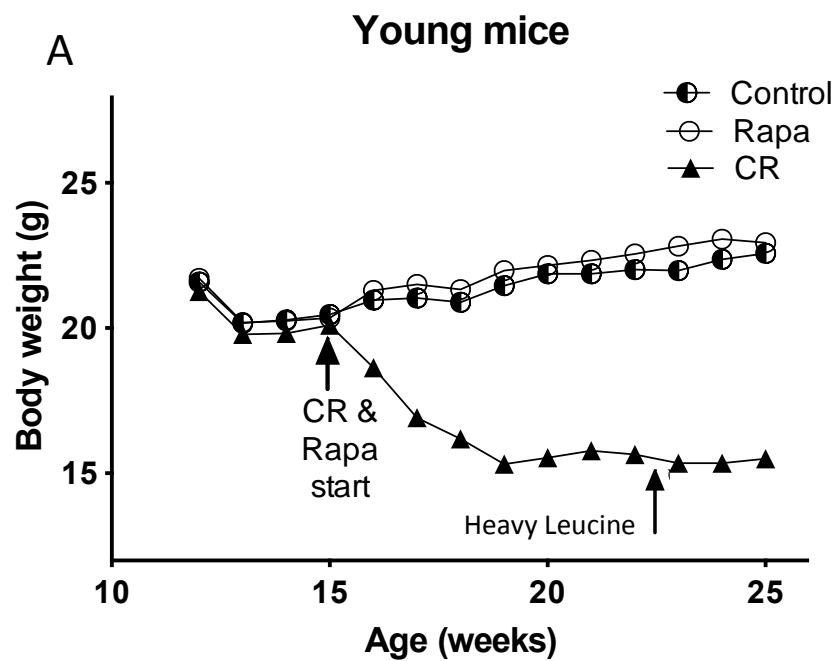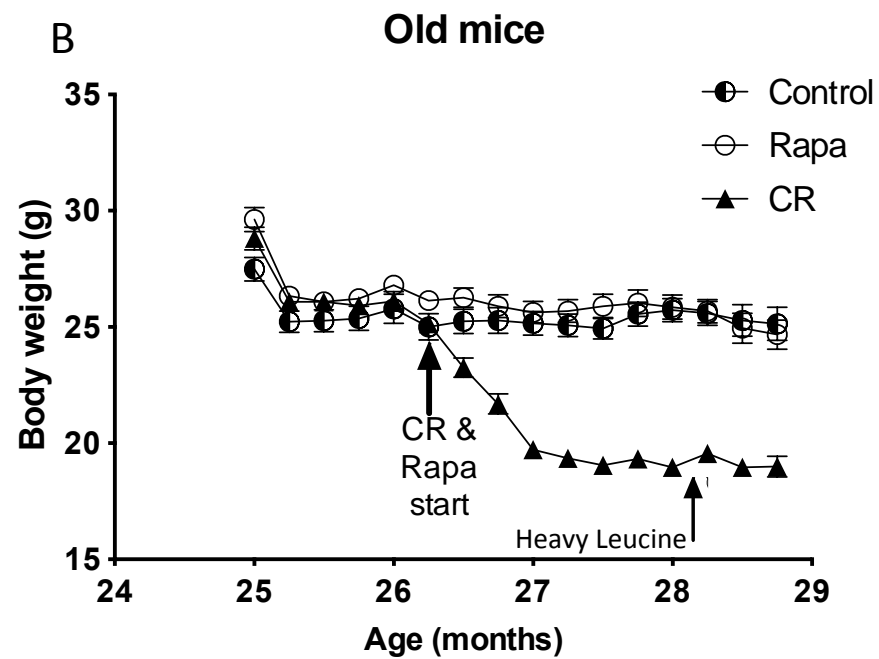

A

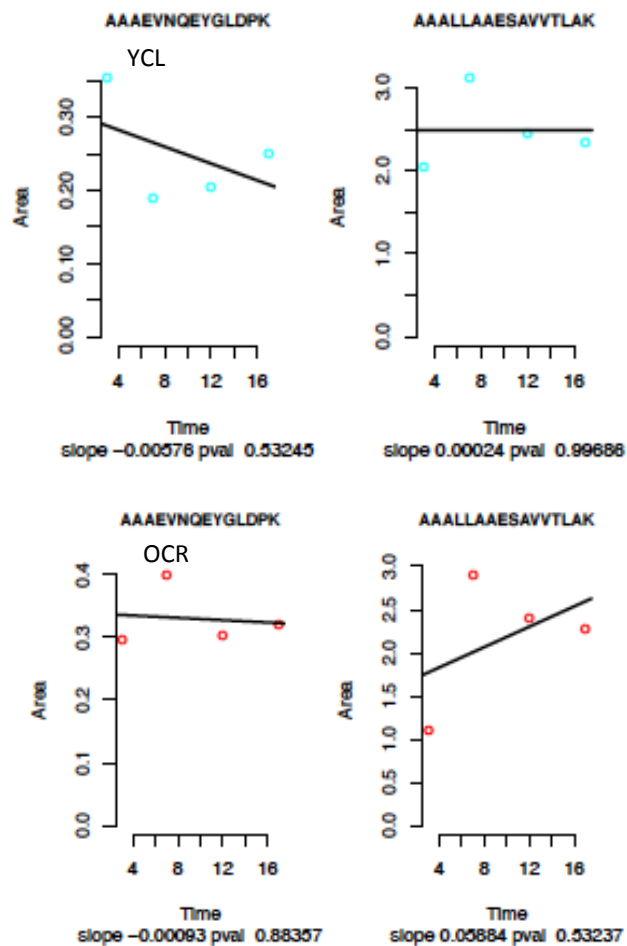

B

Regression slope histograms for each cohort

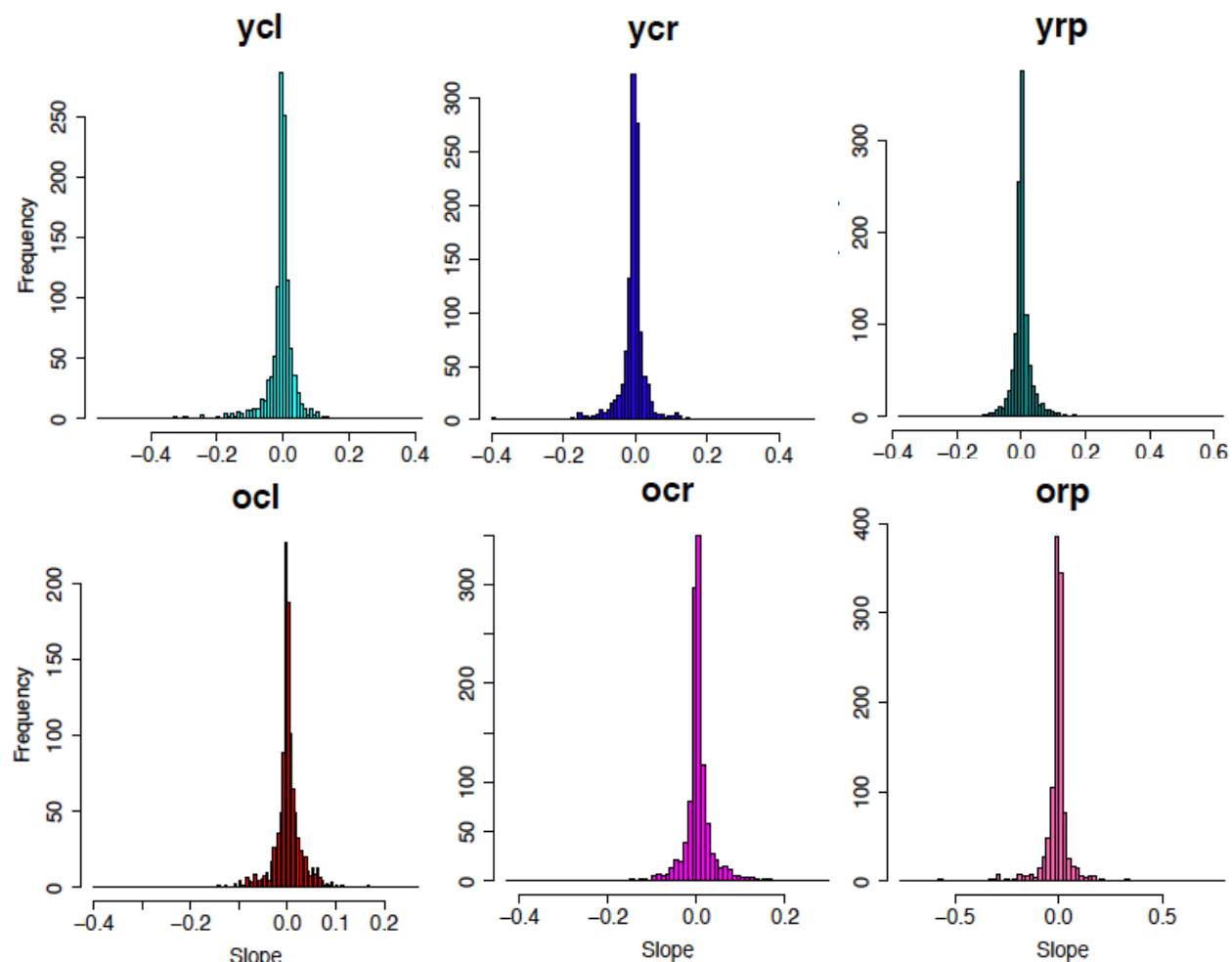

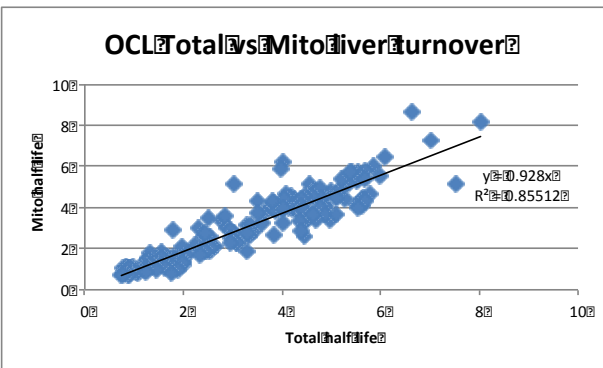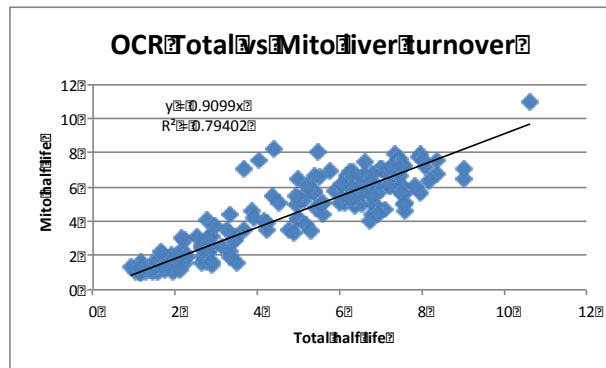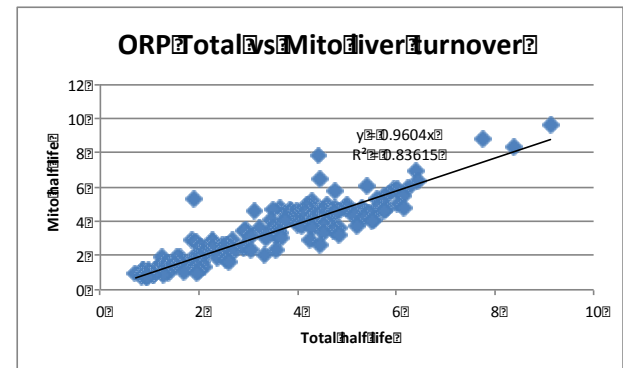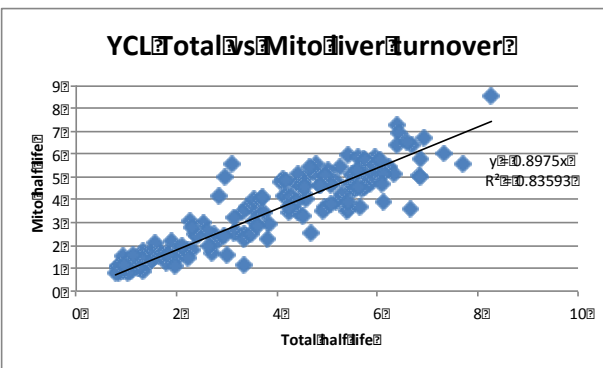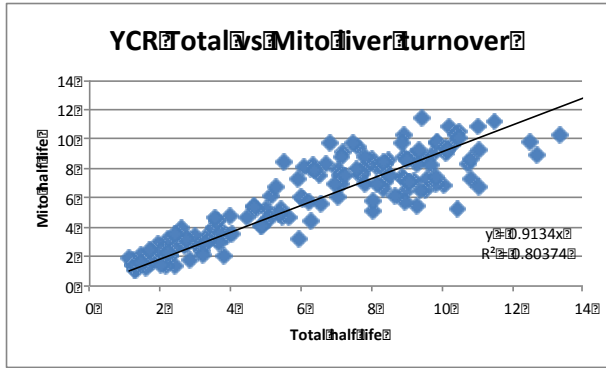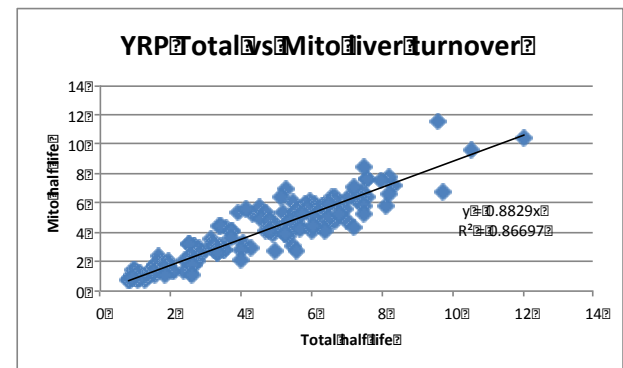



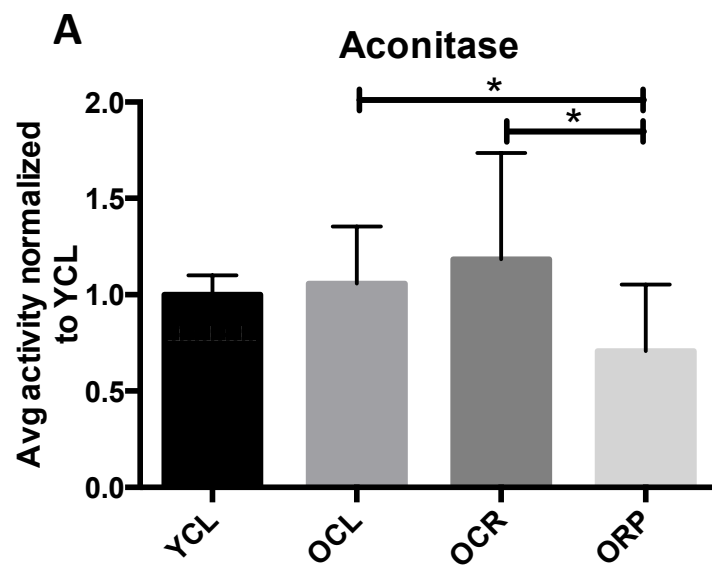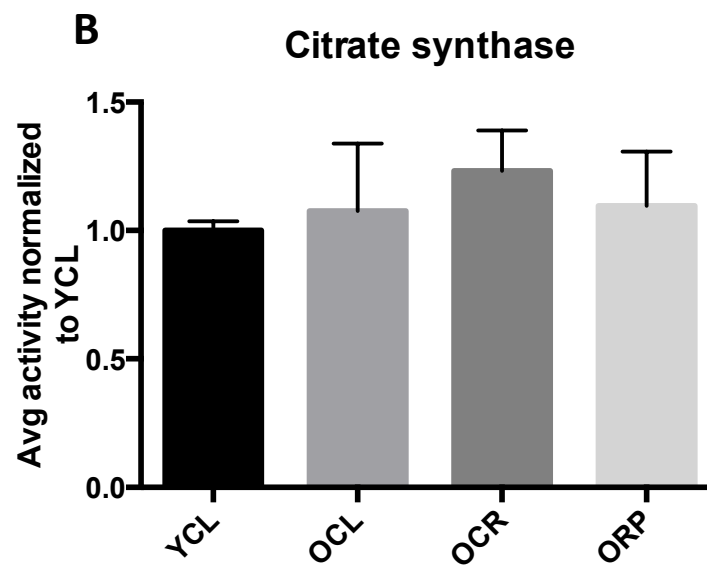

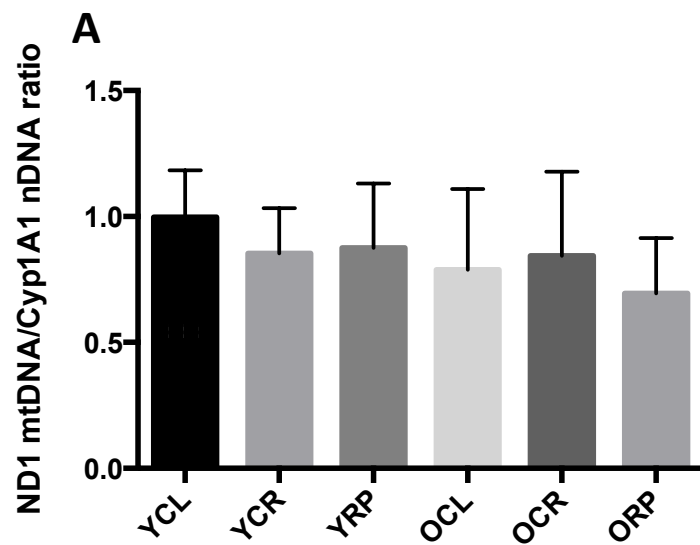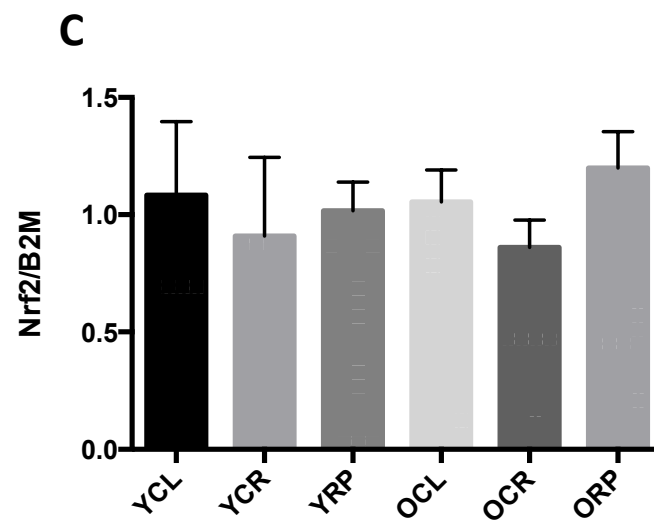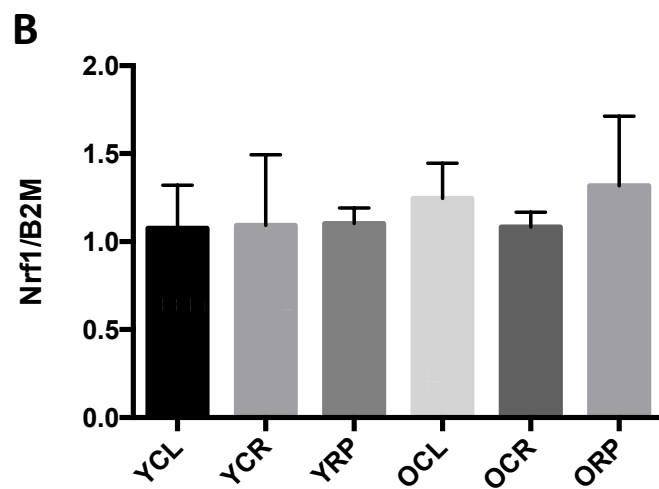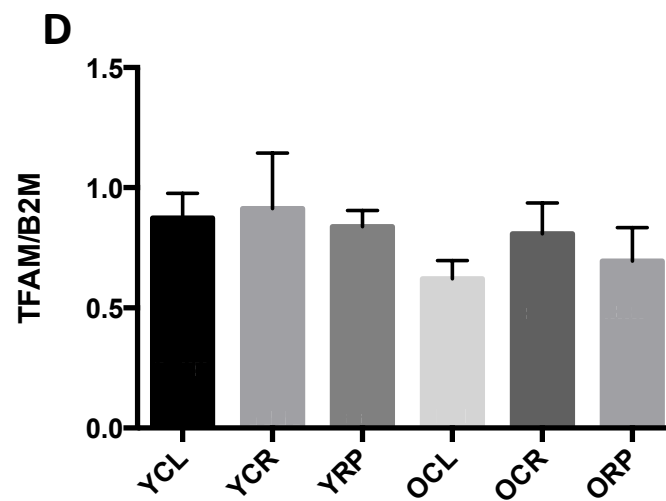

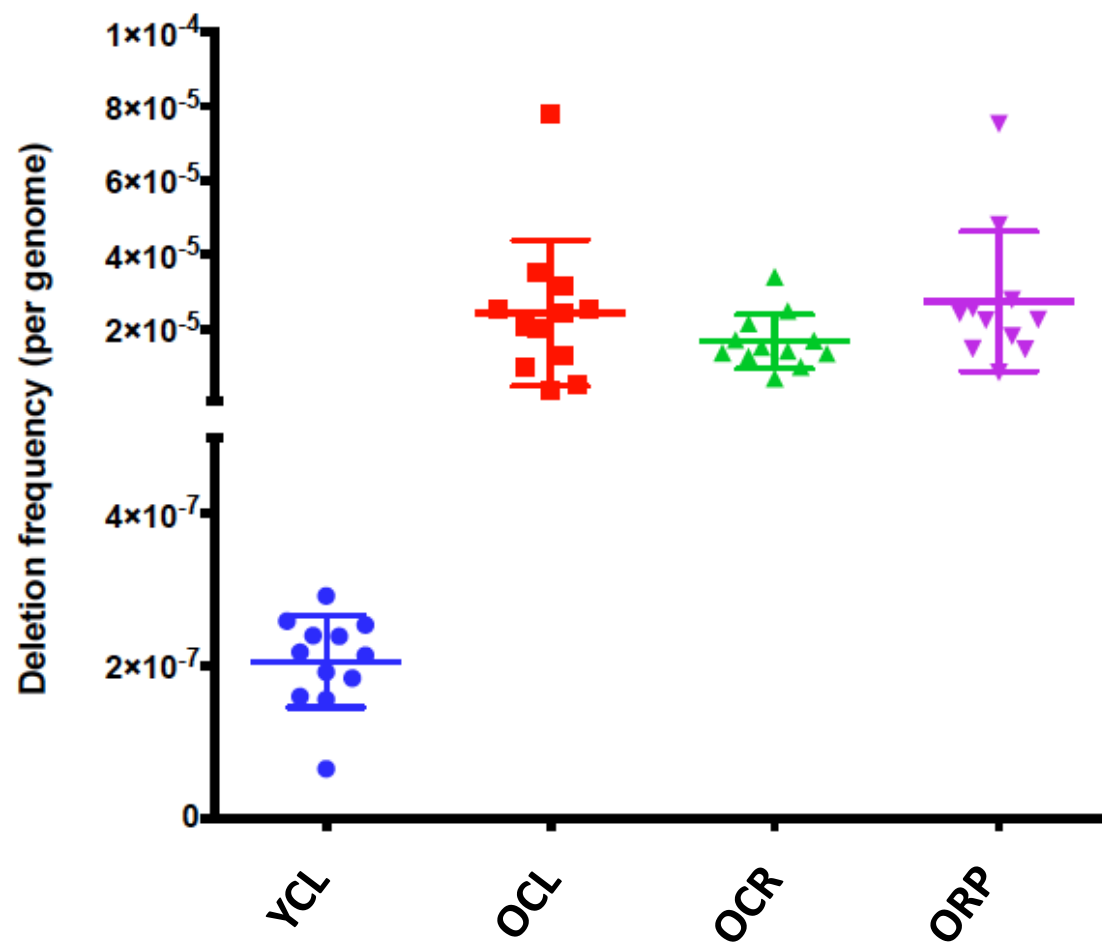

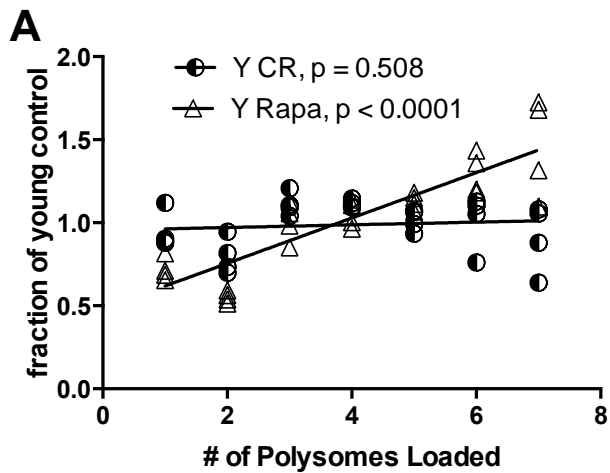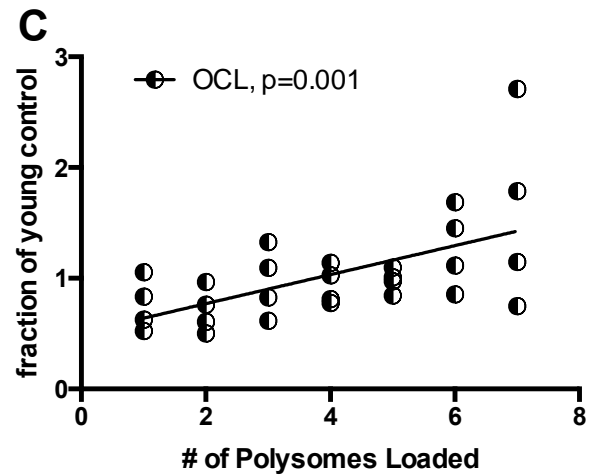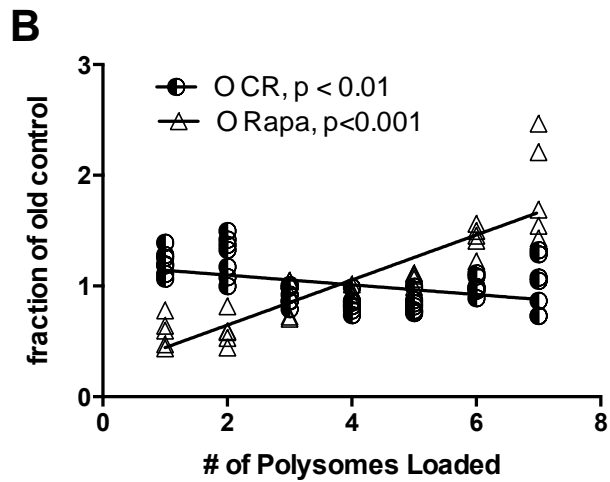

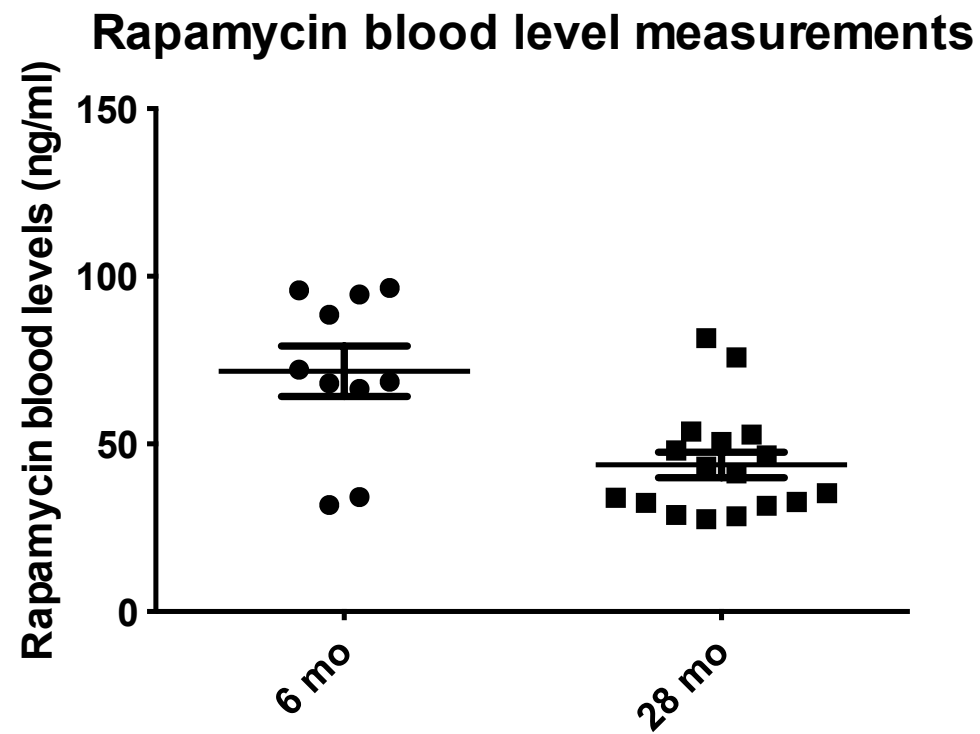

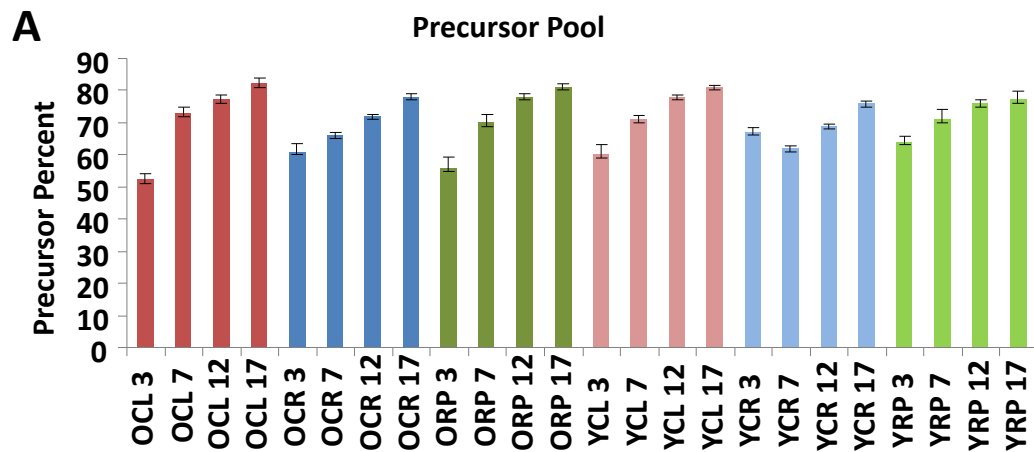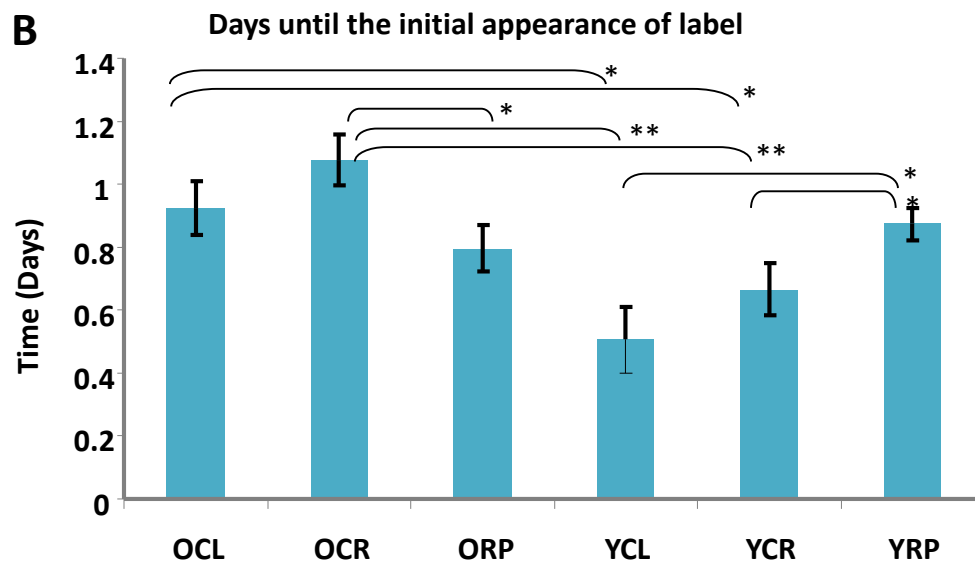

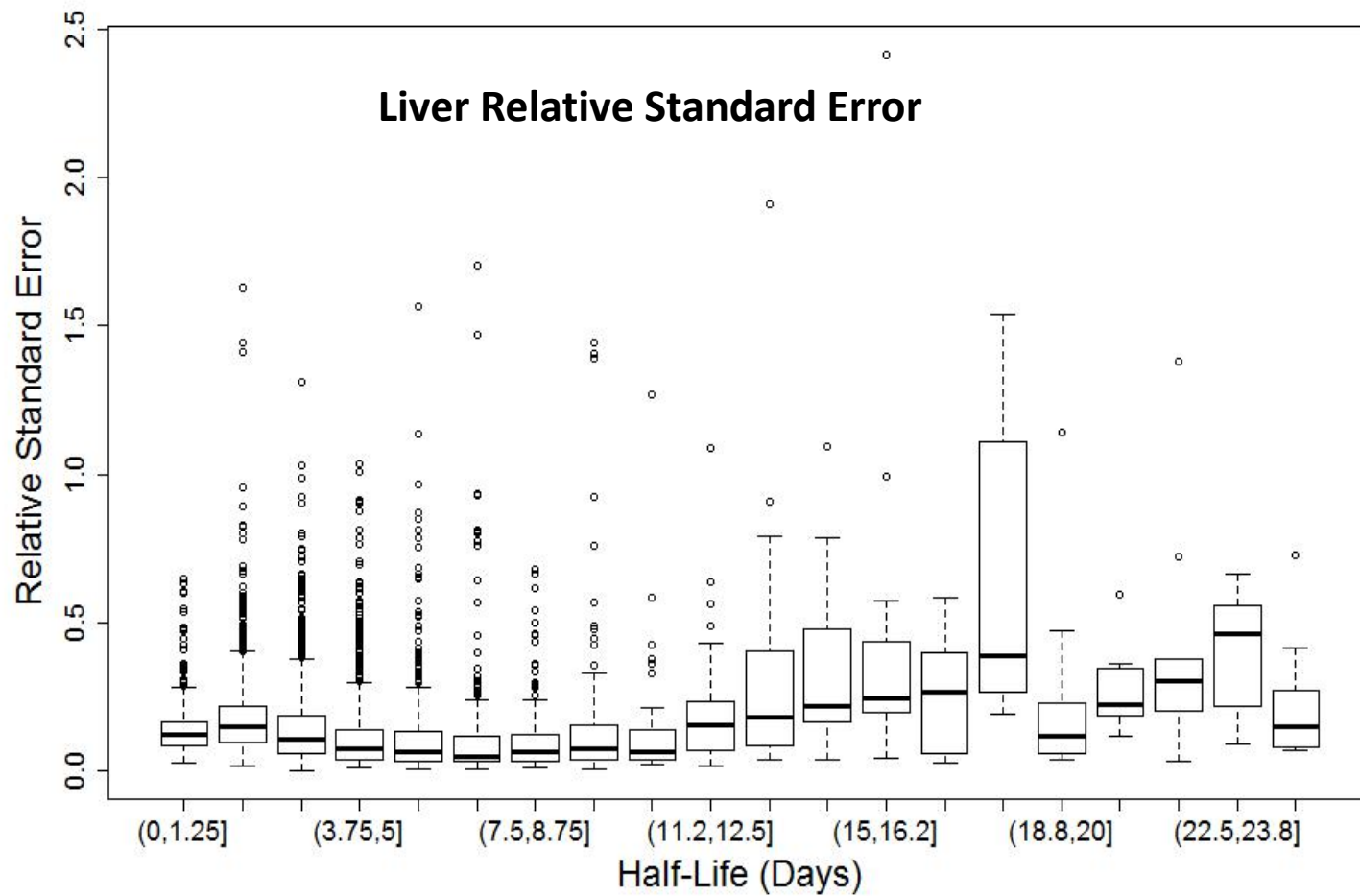

Supplement: Supplementary file 1 [file acel0014-0547-sd1.pdf]
