# Supplementary material for: Subacute calorie restriction and rapamycin discordantly alter mouse liver proteome homeostasis and reverse aging effects
Source: Aging Cell. 2015 Mar 23;14(4):547–57. doi: 10.1111/acel.12317 (PMC4531069; doi:10.1111/acel.12317)
Supplement: Supplementary file 2 [file acel0014-0547-sd2.pdf]

Table S1A. Shortest living pathways

	YCL	YCR	YRP	OCL	OCR	ORP
**Shortest living pathways**			**Avg. Half-life (days)**			
Acute Phase Response Signaling (n=10)	0.894	**2.202**	1.162	1.126	**3.251**	1.013
LXR/RXR Activation (n=16)	0.919	**1.502**	1.124	1.049	2.290	0.977
FXR/RXR Activation (n=8)	0.962	**1.312**	1.031	1.051	1.592	1.015
PPARα/RXRα Activation (n=10)	1.052	1.293	1.183	1.046	1.690	1.044
Estrogen Biosynthesis (n=12)	1.142	**1.424**	1.246	0.989	1.529	1.034
Stearate Biosynthesis (n=6)	1.165	1.615	1.209	1.103	1.761	1.228
Acetone Degradation (to MG) (n=10)	1.168	1.439	1.277	0.987	1.545	1.025
LPS/IL-1 med Inhibition of RXR Function (n=18)	1.188	**1.719**	1.293	1.141	**1.915**	1.259
Bile Acid Biosynthesis, Neutral Pathway (n=4)	1.206	1.814	1.385	1.184	1.958	1.629
Nicotine Degradation (n=14)	1.224	**1.429**	1.384	1.005	1.596	1.112
Fatty Acid β-oxidation (n=8)	1.227	**1.791**	1.248	1.217	**2.043**	1.316
Melatonin Degradation (n=12)	1.234	1.419	1.405	0.984	1.501	1.083
PXR/RXR Activation (n=9)	1.295	1.740	1.442	1.075	1.850	1.290
Fatty Acid Activation (n=3)	1.425	**2.169**	1.398	1.316	**2.339**	1.550
Zymosterol Biosynthesis (n=3)	1.524	2.127	1.233	1.153	1.532	1.261

$P \leq 0.05$ (bold) indicate significance compared to the CL.

Table S1B. Longest living pathways

	YCL	YCR	YRP	OCL	OCR	ORP
**Longest living pathways**			**Avg. half-life (days)**			
Primary Immunodeficiency Signaling (n=5)	11.898	**18.269**	12.499	3.675	**9.273**	**6.346**
Ketolysis and Ketogenesis (n=5)	8.487	8.964	9.252	9.218	7.794	7.791
Acetyl-CoA Biosynthesis I (Pyruvate Dehydrogenase Complex, n=2)	6.817	9.989	4.651	5.395	7.066	4.188
eIF2 Signaling (n=41)	6.767	**8.874**	**9.296**	6.882	6.854	7.489
mTOR Signaling (n=21)	6.701	**8.903**	**9.428**	6.793	7.102	7.676
Mitochondrial Dysfunction (n=18)	6.609	**10.048**	7.037	5.384	**7.212**	5.324
Reg of eIF4 and p70S6K Signaling (n=20)	6.591	**8.926**	**9.465**	6.842	6.740	7.796
Superpathway of Cholesterol Biosyn (n=4)	6.413	9.869	6.683	6.256	8.209	6.074
Fatty Acid β-oxidation (n=7)	6.089	**10.276**	6.661	5.634	**7.557**	4.129
Mevalonate Pathway (n=3)	5.778	**9.460**	5.815	4.975	7.165	4.349
Superpathway of Geranylgeranyldiphosphate Biosynthesis (via Mevalonate, n=3)	5.778	**9.460**	5.815	4.975	**7.165**	4.349
Branched-chain amino acid degradation (BCAA) (n=5)	5.774	**9.557**	6.181	5.160	**7.256**	**4.149**
Glutaryl-CoA Degradation (n=5)	5.766	**9.761**	6.031	5.126	**7.175**	**3.934**
Tryptophan Degradation III (Eukaryotic, n=5)	5.766	**9.761**	6.031	5.126	**7.175**	**3.934**
Tyrosine Degradation (n=2)	5.551	7.718	5.859	4.662	4.738	3.370

$P \leq 0.05$ (bold) indicate significance compared to the CL.

Table S1C. Cellular compartment half-lives

	YCL	YCR	YRP	OCL	OCR	ORP
**Compartment**			**Avg. Half-life (days)**			
Peroxisome (n=26)	1.90	2.93	1.88	1.86	2.22	1.80
Endoplasmic Reticulum (n=107)	2.42	**3.76**	2.98	2.33	3.07	2.50
Lysosome (n=15)	2.62	4.38	3.26	2.71	3.35	2.82
Extracellular Space (n=42)	2.67	4.81	2.52	1.82	2.68	2.32
Plasma Membrane (n=62)	3.93	**6.21**	3.87	2.70	3.46	3.13
Cytoplasm (n=674)	3.96	6.04	4.39	3.43	4.61	4.10
Mitochondrion (n=108)	4.16	**7.11**	4.75	3.70	**5.45**	3.83
Nucleus (n=51)	4.41	6.66	4.76	3.70	4.67	3.85
Ribosome (n=45)	6.04	**8.04**	**8.41**	6.10	6.28	6.70

$p \leq 0.05$ (bold) indicate significance compared to the CL.

Comparisons

Canonical Pathways	Protein	**OCR/OCL**	**ORP/OCL**	**YCL/OCL**	**YCR/YCL**	**YRP/YCL**
chondrial Dysfunction and electron transport	Cox5b	2.66711293	1.58115525	2.62353048	0.31560741	-1.1621456
	Ndufs4	-0.0787726	-0.1321896	-0.3283468	1.00693193	0.31047186
	Hsd17b10	0.23989721	-0.5712862	0.08558021	0.71981803	0.21982563
	Atp5o	0.19447254	-0.2570855	-0.1422051	0.48152353	0.0256311
	Atp5f1	0.05437355	0.18234666	0.62370445	0.79386907	0.18922593
	Gpd2	0.35406662	0.07517009	0.13389501	0.43640484	0.19641615
	Atp5a1	0.53905721	-0.073388	0.26225878	0.4276019	0.11194735
	Atp5b	0.41159165	-0.0553546	0.23712972	0.47060394	0.15548651
	Ndufs7	0.47128171	0.0226348	-0.0170767	0.5905079	0.28614005
	Atp5i	0.36214967	0.04303685	0.14054606	0.735213	0.32815847
	Atp5j2	0.52792069	0.13694234	0.2423094	0.62387639	0.28148255
	Atp5c1	0.44498527	0.02637437	0.20846041	0.68009528	0.21035518
	Atp5d	0.43964885	0.11570617	0.25256703	0.67259829	0.23894357
	Atp5l	0.43234674	0.11791221	0.21497281	0.69988623	0.22907521
	Xdh	0.30229157	0.10665428	-0.3838818	1.22503439	0.50302804
	Ndufb7	0.63289286	0.17131953	-0.3880792	1.49762895	0.48298184
	Ndufs3	0.72355859	0.30551512	-0.0206939	0.66650812	0.52514338
	Mtnd4	0.43003914	0.41099095	-0.2003915	0.95315764	0.34696681
	Ndufs8	0.50409256	0.16424798	-0.1752945	0.92633316	0.35085392
	Uqcrc1	0.49676634	-0.113918	0.04989037	0.71077654	0.10204756
	Uqcrc2	0.5567538	-0.0489939	0.02830702	0.77522855	0.06790964
	Prdx5	0.35957492	0.04463135	0.19770125	0.69631341	0.03815595
	Aifm1	0.40818417	-0.0919753	0.17965763	0.80874694	0.12934063
	Cyc1	0.50814818	0.02709135	0.0361524	0.91220412	0.28176841
	Ndufa8	0.60026728	-0.0124163	0.05609334	0.90960135	0.21509039
	Mtco2	0.53728154	-0.0289794	0.09269738	0.82945883	0.15650558
	Cox7a2	0.58400671	-0.0276201	0.10918933	0.86826726	0.1672678
	Cox4i1	0.55308564	0.05645396	0.15653767	0.72234213	0.14794923
	Txnrd2	0.48663015	0.01515653	0.14556465	0.75453513	0.11583277
	Uqcrh	0.52452999	0.02299207	0.11194902	0.8003394	0.13792815
	Atp5j	0.54319509	0.06342229	0.19335046	0.83157671	0.2238639
	Ndufa2	0.58755535	0.03581802	0.11738379	0.81593716	0.17266918
	Ndufb9	0.60142835	0.04470539	0.11122209	0.80205454	0.18331361
	Cyb5a	0.59731453	0.0604224	0.10720115	0.90289554	0.41448379
	Ndufv2	0.63754833	0.07573109	0.0838592	1.03988306	0.31681404
	Ndufa3	0.68907347	-0.0671434	0.16766071	0.82697607	0.10873401
	Ndufa10	0.789427	0.10564643	0.1988848	0.83103574	0.10212735
	Ndufs2	0.82386309	0.0342489	0.19998518	0.72511379	0.18074206

Mitoc	Sdhb	0.61461071	0.09148089	-0.0931304	0.90340387	0.08723484
	Ndufb10	0.6920985	0.11947095	0.06289219	0.79241573	0.11600481
	Ndufa5	0.68204325	-0.1594953	0.02856066	0.80115645	0.27361314
	Cyb5r3	0.65542625	-0.1145494	-0.0746201	1.00433373	0.13571828
	Sdha	1.01668639	-0.0511382	0.32328573	0.94563725	0.07642552
	Ndufa12	1.29621586	-0.0443185	0.56384684	0.96894201	0.18901219
	Ndufa9	0.91792757	0.35258181	0.14193436	1.2293196	0.11884525
	Cpt1a	0.91251152	0.3191011	0.11825067	0.76539081	0.07397312
	Ndufs5	0.83028963	0.29818292	0.2860967	0.87390951	0.11679638
	Prdx3	-0.9451873	-1.4788082	0.11523118	0.23393986	-0.1008817
	Ndufs6	1.07400633	-0.4635532	0.03625481	1.24985044	0.06656584
	Maob	0.82418132	-0.4394837	0.04508677	0.98042936	0.0122228
	Ndufb4	0.60541333	-0.5248463	0.10102889	0.73046425	0.12004457
	Uqcrb	0.47988541	-0.9019238	-0.1215842	0.61125416	-0.3510922
	Ndufa4	0.94924285	-0.3777577	0.53675882	0.57991041	-0.4941695
	Cox6b1	0.60154674	-0.3251983	0.08838842	0.48898878	-0.3937814
	Uqcrfs1	0.73868578	0.32006599	0.66655738	0.05239059	-0.1271417
	Ndufv1	0.758367	-0.2934406	0.11718221	0.60700104	0.03841611
	Ndufs1	0.64671971	-0.104495	0.16771213	0.58299507	0.09691619
	Cox6a1	0.74061951	-0.0932272	0.14054386	0.71402248	0.03467071
	Ndufa13	0.66479053	-0.1280972	0.08364453	0.65842054	0.00252625
	Ndufb8	0.67191272	0.00771232	0.19982066	0.71887232	-0.2423608
	Cycs	0.73425454	0.06355378	0.14925738	0.59674514	-0.0523916
	Cat	0.52370124	0.06714458	0.13649342	0.5562019	-0.1067881
	Rps2	0.025904	0.16692625	-0.0637678	0.57220968	0.49587859
	Rps16	0.02956494	0.16047354	-0.0255819	0.62661957	0.52026901
	Rpl31	0.11186182	0.16988746	-0.0938186	0.60765503	0.55817712
	Rpl7a	0.05653191	0.12607005	-0.132648	0.61832133	0.47577773
	Rpsa	0.06332635	0.20185257	-0.0034633	0.46643939	0.50575084
	Rps3a	0.05622026	0.22657023	-0.0026124	0.43519195	0.52437182
	Rpl22	-0.0141974	0.22333863	-0.0489554	0.41824256	0.56888595
	Rpl18a	-0.0144207	0.17388742	-0.1109073	0.47408169	0.51409696
	Rps18	0.04615437	0.15703095	-0.0252362	0.47184467	0.42234595
	Rpl4	0.01541395	0.11055832	-0.0250983	0.45054436	0.40782756
	Rps14	0.01086537	0.20648846	-0.0448226	0.47568677	0.41556036
	Rpl27	0.03275302	0.19647733	-0.0591432	0.44048633	0.36439455
	Rpl24	0.04807906	0.07804188	-0.0621226	0.52552544	0.3419812
	Rpl6	0.0354463	0.15342927	0.06625916	0.47625524	0.40261769
	Rps23	0.07500323	0.05675199	0.01462189	0.43718096	0.38344755
	Rplp2	0.20560032	0.17469639	-0.0072128	0.46457178	0.37678295
	Rpl19	0.15652631	0.05631588	-0.0726165	0.53192798	0.4475856

EIF2 Signaling	Rpl11	0.18608892	0.15285794	-0.0606438	0.61124458	0.41845055
	Rpl18	0.14765162	0.09435923	-0.0643575	0.59129279	0.37135705
	Rpl17	0.15139801	0.10957084	-0.0347053	0.615013	0.40445555
	Rpl23	-0.0294013	0.16925744	-0.0533864	0.20466064	0.30539134
	Rps3	-0.0458204	0.10592339	-0.0122551	0.20939182	0.44691432
	Rps15	0.08384705	0.12339766	-0.039371	0.32282476	0.46970817
	Rps10	0.05636581	0.08970779	-0.1085747	0.34989731	0.31578502
	Rps8	-0.1196617	0.02468098	-0.1072222	0.37475468	0.63473814
	Rpl3	-0.021063	-0.1344885	-0.1017409	0.49051144	0.5576714
	Rps5	-0.1827172	0.38173642	-0.1046974	0.27355484	0.6278103
	Rps19	-0.1083051	0.18153484	-0.111896	0.35266686	0.47072411
	Rpl12	-0.1386602	0.21377268	-0.0782537	0.29509356	0.59365665
	Rpl14	-0.0435892	0.16393304	-0.0650495	0.32663561	0.5803902
	Rpl8	-0.70169	0.10994735	-0.0082869	0.5923798	0.45204541
	Rpl30	-0.044794	0.15438118	-0.2613997	0.63124929	0.67725777
	Rps12	-0.019435	0.29904746	-0.1075608	0.70319573	0.75404193
	Rps6	-0.039799	0.29510829	-0.1030913	0.58503405	0.56259132
	Rps24	-0.0415941	0.25768207	-0.0956556	0.56754852	0.70175081
	Rps4x	-0.1451373	0.21056872	-0.0654706	0.58850124	0.67968277
	Rpl22l1	-0.420328	0.11379605	0.06573735	-1.5101128	-0.5982018
	Eif2s1	0.86663276	-0.3274842	0.10970412	1.25388318	0.54028761
	Rpl27a	0.36091252	-0.2495613	0.04940441	0.78767953	-0.8673415
	Rpl13	-0.0837748	0.17305481	0.32502584	-0.390743	0.00663194
	Rps26	-0.136342	0.24953633	-0.0193772	-0.260356	0.48230093
	Pabpc1	-0.4271186	-0.0888192	0.20589579	-0.4113162	0.59753091
	Rpl7	0.39551293	-0.040642	0.2940996	0.44186998	0.3944686
	Rplp0	-0.040142	-0.2082924	-0.0329687	0.27795845	0.36739161
	Rplp1	0.06647442	-0.5148149	-0.0596696	0.28717562	0.31472758
	Rps15a	-0.0422416	-0.0174934	-0.0007557	0.2492216	0.09093094
	Rpl9	0.17286191	-0.1918332	0.12538896	0.0956043	0.14538862
BCAA	Acadm	1.16836625	-0.1238838	0.82102527	0.09148468	0.29295764
	Abat	0.9794764	-0.2721885	-0.1418136	1.22206664	0.66598877
	Hibadh	0.81114318	-0.3912836	-0.2268163	0.18799187	0.83279919
	Dld	0.52744809	-0.0799067	0.34009751	0.8563888	-0.1550641
	Hadha	0.62811381	-0.0691577	0.33285223	0.7800026	0.05940517
	Acat1	0.68084577	-0.0682348	0.27490855	0.77847623	-0.0219048
	Sds	0.81697452	-0.0797196	0.05770259	1.04629251	0.00818509
	Hibch	0.67820727	0.14099506	0.30342446	0.76634399	0.1666492
	Acads	0.66271941	0.03814458	0.11479679	0.9079897	0.28605643
	Mccc2	0.27460212	0.44481361	-0.309359	0.86403847	0.58054117
	Dbt	0.419736	0.2180892	-0.0364223	1.00513133	-0.3149946

	Acadsb	0.4634967	-0.2339255	-0.3847575	1.06327297	0.17498883
	Bckdhb	0.53012426	-0.0993689	0.01161662	0.90809366	0.15835954
	Hmgcl	0.41116142	0.09052859	-0.0044019	0.85624777	0.16054035
	Ehhadh	0.44625233	-0.0139232	-0.0542034	0.79238931	0.10526217
	Mccc1	-0.586576	-0.3178074	0.1904506	0.70769716	0.59011166
	Aldh6a1	0.36103791	-0.130301	-0.0534215	0.51035572	-0.0961815
	Echs1	0.62447406	-0.4274016	0.08450947	0.78037571	0.22494722
	Hsd17b10	0.23989721	-0.5712862	0.08558021	0.71981803	0.21982563
	Hadhb	0.29851846	-0.4141473	0.06754758	0.57782504	-0.0092558
Fatty Acid β-oxidation	Hsd17b10	0.23989721	-0.5712862	0.08558021	0.71981803	0.21982563
	Eci1	0.12792071	-0.5371793	0.00150569	0.8123275	0.30422839
	Hsd17b8	0.59876707	-0.8329527	0.12014013	0.93399368	0.06393802
	Echs1	0.62447406	-0.4274016	0.08450947	0.78037571	0.22494722
	Acaa2	0.54400019	-0.2941021	0.16676297	0.64702007	-0.0566321
	Hadhb	0.29851846	-0.4141473	0.06754758	0.57782504	-0.0092558
	Acadm	1.16836625	-0.1238838	0.82102527	0.09148468	0.29295764
	Acaa1b	0.42238297	0.06642385	0.09194762	0.3503211	0.00783129
	Hsd17b4	0.15631929	0.02245419	-0.0758848	0.43529506	0.09501188
	Eci2	0.96004152	0.04054516	0.56064798	0.80168849	-0.101241
	Sds	0.81697452	-0.0797196	0.05770259	1.04629251	0.00818509
	Hadh	0.53874789	-0.0007232	0.19515919	0.80535086	0.10877227
	Hadha	0.62811381	-0.0691577	0.33285223	0.7800026	0.05940517
	Acsl1	0.68926196	0.30272098	0.13919103	1.01280886	0.41469853
	Acsl5	0.63688509	-0.0345611	-0.096886	0.93194224	0.24016806
	Slc27a5	0.59717416	-0.0220227	-0.1134569	0.91017768	0.17663322
	Slc27a2	0.58527596	-0.0236417	-0.1310831	0.6438544	0.28425697
	Ehhadh	0.44625233	-0.0139232	-0.0542034	0.79238931	0.10526217
Gluconeogenesis	Pecr	0.38297323	0.43107381	0.50880204	2.16325546	-0.0373858
	Aldh2	1.18504089	-0.0889127	0.23208829	1.18573902	-0.0595114
	Aldob	1.02229332	-0.0495277	0.25965289	0.91981837	0.0363255
	Adh5	0.90616335	-0.4812057	0.17624961	1.0412218	0.58800348
	Acsl1	0.68926196	0.30272098	0.13919103	1.01280886	0.41469853
	Aldh1b1	0.71015076	0.13262631	0.07739299	1.34188546	0.5239206
	Pgk1	0.13807214	-0.0244011	-0.3109528	0.79127336	0.69072163
	Pklr	0.30141261	-0.07963	0.03231508	0.83251588	0.28987274
	Aldh4a1	0.16470427	-0.0443146	-0.0242634	0.9544955	0.3118812
	Hsd17b10	0.23989721	-0.5712862	0.08558021	0.71981803	0.21982563
	Aldh1a1	0.08380617	-0.3849699	0.15192993	0.64585292	0.19690445
	Mdh2	0.36299284	-0.1627006	0.24373618	0.3975898	0.25322808
	Eno1	0.3949641	-0.1150406	0.19532879	0.48040555	0.27795718
	Fbp1	0.53264535	-0.290448	0.32812834	0.83504192	0.13783926

Glycolysis and G	Mdh1	0.53391523	-0.2613065	0.08351761	0.8483325	0.2513038
	Tpi1	0.43579884	-0.1286768	0.1804371	0.76314194	0.16737668
	Gapdh	0.80417867	-0.3337031	0.29679372	0.07820782	0.02742225
	Akr1a1	0.78156873	0.72472683	0.29129839	0.20599932	-0.066689
	Pkm	0.88513734	0.26309111	0.38726661	0.0798828	-0.0154025
	Agl	0.05706838	0.121586	0.77377434	0.36356287	0.02814266
	Adh1	0.43501624	-0.0346183	0.20058306	0.64791522	0.00300465
	Dhrs4	0.55706093	-0.0192048	0.17581789	0.65880036	0.04057293
	Me1	0.73205089	0.22795012	0.31954404	0.8575496	0.10264778
	Gpi	0.528042	0.09630288	0.24826739	0.85399568	0.13354845
	Aldh3a2	0.63043709	0.087697	0.4372669	0.71685142	0.03358944
	Pygl	0.43218101	0.06053097	0.43181731	0.80657746	-0.1212346
	Pgam1	-1.0039461	-1.1932733	-0.2780986	0.00505649	-0.4915774
	Aldh1a7	1.17075352	0.464924	1.67364786	0.37698218	-1.1341536
Nicotine Degradation	Cyp3a44	0.54586304	0.31160313	1.39960263	1.21275813	3.22961387
	Ugt1a2	-0.0339525	0.89133366	-0.4714489	0.24565925	0.23489518
	Ugt1a9	-0.3111283	-0.0634462	-0.7869802	0.55108915	0.67713658
	Ugt1a6	0.32144946	-0.1302243	-0.4142914	0.34791413	0.69782043
	Cyp51a1	0.00477061	-0.2638659	-0.3966843	0.47051188	-0.0135173
	Cyp2c37	0.06686521	-0.0397622	-0.5223683	0.62024055	0.30687009
	Cyp2f2	0.35381182	-0.2029507	0.46128759	0.17446153	-0.9081891
	Cyp2a4	0.61831091	0.34050813	0.42712651	1.64179312	0.60918206
	Cyp1a2	0.01009457	0.14843414	-0.0303565	0.30524169	-0.090248
	Cyp2c29	0.34370556	-0.0588797	0.02003424	0.50115965	0.06096738
	Fmo5	0.32685201	0.00234338	0.1563941	0.32846637	0.07685504
	Cyp2e1	0.70073766	0.28964872	0.05610049	0.88100493	-0.0270627
	Cyp2c40	0.53670049	-0.0152772	-0.0986735	0.78576218	-0.1064382
	Cyp2d10	0.35075943	0.21962814	0.1527185	0.75065459	-0.0863294
	Ugt2a3	0.38078858	-0.1305193	-0.24026	0.64301344	0.27153211
	Ugt1a1	0.46122223	0.00209304	-0.079887	0.53864214	0.41381992
	Inmt	0.36579287	-0.1643049	0.11934274	0.67160439	0.168453
	Por	0.54990189	-0.0593997	-0.0688964	0.855203	0.23591646
	Ugt2b17	0.76077718	0.22543568	-0.1574788	0.91899605	0.54430977
	Fmo1	0.3859376	0.23106254	0.00087664	1.08132611	0.40494885
dation	Cyp3a44	0.54586304	0.31160313	1.39960263	1.21275813	3.22961387
	Sult1a1	0.27779144	-0.4739511	-0.0320057	0.54155163	-0.0581422
	Cyp2c29	0.34370556	-0.0588797	0.02003424	0.50115965	0.06096738
	Ugt2a3	0.38078858	-0.1305193	-0.24026	0.64301344	0.27153211
	Ugt1a1	0.46122223	0.00209304	-0.079887	0.53864214	0.41381992
	Cyp1a2	0.01009457	0.14843414	-0.0303565	0.30524169	-0.090248
	Cyp51a1	0.00477061	-0.2638659	-0.3966843	0.47051188	-0.0135173

Melatonin Degra	Cyp2c37	0.06686521	-0.0397622	-0.5223683	0.62024055	0.30687009
	Ugt1a2	-0.0339525	0.89133366	-0.4714489	0.24565925	0.23489518
	Ugt1a6	0.32144946	-0.1302243	-0.4142914	0.34791413	0.69782043
	Ugt1a9	-0.3111283	-0.0634462	-0.7869802	0.55108915	0.67713658
	Cyp2f2	0.35381182	-0.2029507	0.46128759	0.17446153	-0.9081891
	Cyp2a4	0.61831091	0.34050813	0.42712651	1.64179312	0.60918206
	Maob	0.82418132	-0.4394837	0.04508677	0.98042936	0.0122228
	Ugt2b17	0.76077718	0.22543568	-0.1574788	0.91899605	0.54430977
	Cyp2c40	0.53670049	-0.0152772	-0.0986735	0.78576218	-0.1064382
	Por	0.54990189	-0.0593997	-0.0688964	0.855203	0.23591646
	Cyp2e1	0.70073766	0.28964872	0.05610049	0.88100493	-0.0270627
Estrogen Biosynthesis	Cyp2d10	0.35075943	0.21962814	0.1527185	0.75065459	-0.0863294
	Por	0.54990189	-0.0593997	-0.0688964	0.855203	0.23591646
	Akr1c6	0.62079758	0.0722894	0.10089791	1.01366316	0.36050602
	Cyp2e1	0.70073766	0.28964872	0.05610049	0.88100493	-0.0270627
	Hsd17b13	0.6146066	0.19158752	0.00408373	0.63137902	0.13739092
	Cyp2c40	0.53670049	-0.0152772	-0.0986735	0.78576218	-0.1064382
	Cyp2d10	0.35075943	0.21962814	0.1527185	0.75065459	-0.0863294
	Cyp1a2	0.01009457	0.14843414	-0.0303565	0.30524169	-0.090248
	Cyp2c29	0.34370556	-0.0588797	0.02003424	0.50115965	0.06096738
	Hsd17b4	0.15631929	0.02245419	-0.0758848	0.43529506	0.09501188
	Cyp51a1	0.00477061	-0.2638659	-0.3966843	0.47051188	-0.0135173
	Cyp2c37	0.06686521	-0.0397622	-0.5223683	0.62024055	0.30687009
	Cyp2f2	0.35381182	-0.2029507	0.46128759	0.17446153	-0.9081891
	Hsd17b10	0.23989721	-0.5712862	0.08558021	0.71981803	0.21982563
	Hsd17b8	0.59876707	-0.8329527	0.12014013	0.93399368	0.06393802
	Cyp3a44	0.54586304	0.31160313	1.39960263	1.21275813	3.22961387
	Hsd17b2	1.40180384	0.39081167	0.49500034	1.18809919	0.84595504
	Cyp2a4	0.61831091	0.34050813	0.42712651	1.64179312	0.60918206
Glutaryl-CoA Degradation	Hsd17b12	0.82898142	0.16645098	0.00182922	1.17447125	0.8151486
	Hsd17b8	0.59876707	-0.8329527	0.12014013	0.93399368	0.06393802
	Hsd17b10	0.23989721	-0.5712862	0.08558021	0.71981803	0.21982563
	Acat2	0.42986085	-0.4310873	0.19860352	0.58035465	-0.0436768
	Hadhb	0.29851846	-0.4141473	0.06754758	0.57782504	-0.0092558
	Gcdh	0.79212577	-0.1702838	0.20343917	1.05099783	-0.0037052
	Hadh	0.53874789	-0.0007232	0.19515919	0.80535086	0.10877227
	Hadha	0.62811381	-0.0691577	0.33285223	0.7800026	0.05940517
	Acat1	0.68084577	-0.0682348	0.27490855	0.77847623	-0.0219048
	Ehhadh	0.44625233	-0.0139232	-0.0542034	0.79238931	0.10526217
	Hsd17b4	0.15631929	0.02245419	-0.0758848	0.43529506	0.09501188
	Hsd17b10	0.23989721	-0.5712862	0.08558021	0.71981803	0.21982563

Tryptophan Degradation	Acat2	0.42986085	-0.4310873	0.19860352	0.58035465	-0.0436768
	Hadhb	0.29851846	-0.4141473	0.06754758	0.57782504	-0.0092558
	Kmo	0.54839471	-0.4344869	0.26370063	0.82566726	-0.248331
	Hsd17b8	0.59876707	-0.8329527	0.12014013	0.93399368	0.06393802
	Ehhadh	0.44625233	-0.0139232	-0.0542034	0.79238931	0.10526217
	Hsd17b4	0.15631929	0.02245419	-0.0758848	0.43529506	0.09501188
	Haao	0.87028976	0.19794902	0.55036576	0.64810475	0.1083742
	Gcdh	0.79212577	-0.1702838	0.20343917	1.05099783	-0.0037052
	Hadh	0.53874789	-0.0007232	0.19515919	0.80535086	0.10877227
	Hadha	0.62811381	-0.0691577	0.33285223	0.7800026	0.05940517
	Acat1	0.68084577	-0.0682348	0.27490855	0.77847623	-0.0219048
LPS/IL-1 Mediated Inhibition of RXR Function	Fabp5	-0.1800653	0.67710845	0.70216779	0.14433881	-0.4019312
	Aldh1a1	0.08380617	-0.3849699	0.15192993	0.64585292	0.19690445
	Sult1a1	0.27779144	-0.4739511	-0.0320057	0.54155163	-0.0581422
	Fabp1	0.22860028	-0.2260648	0.4936006	0.62004392	0.21041152
	Gstp1	0.50680946	-0.4430297	0.23067303	0.52208144	0.28528261
	Gstm1	0.42200388	-0.174705	0.18821091	0.64754366	0.1946458
	Cyp3a11	0.43556598	0.22382802	-0.4015452	0.47802012	0.10786821
	Aldh6a1	0.36103791	-0.130301	-0.0534215	0.51035572	-0.0961815
	Acox1	0.43032248	0.02917219	-0.0064539	0.5775751	-0.0444644
	Cyp2c29	0.34370556	-0.0588797	0.02003424	0.50115965	0.06096738
	Scarb1	0.0196927	0.0746384	0.06629296	0.1497472	-0.1183196
	Cyp4a10	0.24094635	-0.0882384	0.05032839	0.28872067	-0.0246162
	Fmo5	0.32685201	0.00234338	0.1563941	0.32846637	0.07685504
	Apoe	2.37187728	0.49577673	1.03006591	1.73417075	-0.4895671
	Aldh3a2	0.63043709	0.087697	0.4372669	0.71685142	0.03358944
	Aldh8a1	0.83710916	-0.0659388	0.42801347	0.58092763	0.02518517
	Slco1b2	0.73382705	0.10875977	0.28180526	0.76181829	-0.2925342
	Hmgcs2	0.7390597	-0.1716354	0.14655202	0.70974273	-0.0922155
	Gsta3	0.40703244	0.11598574	0.0429769	0.89547027	-0.1189361
	Abcb11	0.42714374	0.26865928	0.29804048	0.62571325	-0.1487017
	Cyp2a12	0.62666739	0.18290791	0.21805751	0.55359447	-0.046801
	Cat	0.52370124	0.06714458	0.13649342	0.5562019	-0.1067881
	Maob	0.82418132	-0.4394837	0.04508677	0.98042936	0.0122228
	Aldh1l1	0.8769339	0.11829569	0.48480215	0.9788333	0.27938446
	Slc10a1	0.91349452	0.07692732	0.04425267	0.92963848	-0.0643525
	Cpt1a	0.91251152	0.3191011	0.11825067	0.76539081	0.07397312
	Slc27a2	0.58527596	-0.0236417	-0.1310831	0.6438544	0.28425697
	Gstt1	0.46447197	0.17012454	0.05391968	0.76991653	0.2798069
	Gstm2	0.49281219	0.06426905	0.00558161	0.62449445	0.24353827
	Aldh4a1	0.16470427	-0.0443146	-0.0242634	0.9544955	0.3118812

	Cpt2	0.4959683	-0.0362797	-0.0101775	0.94250928	0.33969091
	Acsl5	0.63688509	-0.0345611	-0.096886	0.93194224	0.24016806
	Slc27a5	0.59717416	-0.0220227	-0.1134569	0.91017768	0.17663322
	Papss2	1.12559626	0.07317715	-0.2653388	1.42599356	0.94141116
	Cyp2a4	0.61831091	0.34050813	0.42712651	1.64179312	0.60918206
	Aldh1b1	0.71015076	0.13262631	0.07739299	1.34188546	0.5239206
	Acsl1	0.68926196	0.30272098	0.13919103	1.01280886	0.41469853
Serotonin Degradation	Fmo1	0.3859376	0.23106254	0.00087664	1.08132611	0.40494885
	Aldh1a7	1.17075352	0.464924	1.67364786	0.37698218	-1.1341536
	Pecr	0.38297323	0.43107381	0.50880204	2.16325546	-0.0373858
	Ugt2b17	0.76077718	0.22543568	-0.1574788	0.91899605	0.54430977
	Aldh1b1	0.71015076	0.13262631	0.07739299	1.34188546	0.5239206
	Adh5	0.90616335	-0.4812057	0.17624961	1.0412218	0.58800348
	Maob	0.82418132	-0.4394837	0.04508677	0.98042936	0.0122228
	Aldh2	1.18504089	-0.0889127	0.23208829	1.18573902	-0.0595114
	Akr1a1	0.78156873	0.72472683	0.29129839	0.20599932	-0.066689
	Ugt1a2	-0.0339525	0.89133366	-0.4714489	0.24565925	0.23489518
	Ugt1a9	-0.3111283	-0.0634462	-0.7869802	0.55108915	0.67713658
	Ugt1a6	0.32144946	-0.1302243	-0.4142914	0.34791413	0.69782043
	Aldh4a1	0.16470427	-0.0443146	-0.0242634	0.9544955	0.3118812
	Ugt2a3	0.38078858	-0.1305193	-0.24026	0.64301344	0.27153211
	Ugt1a1	0.46122223	0.00209304	-0.079887	0.53864214	0.41381992
	Aldh3a2	0.63043709	0.087697	0.4372669	0.71685142	0.03358944
	Adh1	0.43501624	-0.0346183	0.20058306	0.64791522	0.00300465
	Dhrs4	0.55706093	-0.0192048	0.17581789	0.65880036	0.04057293
	Sult1a1	0.27779144	-0.4739511	-0.0320057	0.54155163	-0.0581422
	Hsd17b10	0.23989721	-0.5712862	0.08558021	0.71981803	0.21982563
	Aldh1a1	0.08380617	-0.3849699	0.15192993	0.64585292	0.19690445
Noradrenaline and Adrenaline Degradation	Aldh1a7	1.17075352	0.464924	1.67364786	0.37698218	-1.1341536
	Akr1a1	0.78156873	0.72472683	0.29129839	0.20599932	-0.066689
	Hsd17b10	0.23989721	-0.5712862	0.08558021	0.71981803	0.21982563
	Aldh1a1	0.08380617	-0.3849699	0.15192993	0.64585292	0.19690445
	Aldh3a2	0.63043709	0.087697	0.4372669	0.71685142	0.03358944
	Adh1	0.43501624	-0.0346183	0.20058306	0.64791522	0.00300465
	Dhrs4	0.55706093	-0.0192048	0.17581789	0.65880036	0.04057293
	Comt	0.38433812	0.32239398	0.16833807	0.68834248	0.29959073
	Aldh4a1	0.16470427	-0.0443146	-0.0242634	0.9544955	0.3118812
	Pecr	0.38297323	0.43107381	0.50880204	2.16325546	-0.0373858
	Maob	0.82418132	-0.4394837	0.04508677	0.98042936	0.0122228
	Aldh2	1.18504089	-0.0889127	0.23208829	1.18573902	-0.0595114
	Adh5	0.90616335	-0.4812057	0.17624961	1.0412218	0.58800348

	Aldh1b1	0.71015076	0.13262631	0.07739299	1.34188546	0.5239206
Ethanol Degradation	Aldh1a7	1.17075352	0.464924	1.67364786	0.37698218	-1.1341536
	Aldh4a1	0.16470427	-0.0443146	-0.0242634	0.9544955	0.3118812
	Hsd17b10	0.23989721	-0.5712862	0.08558021	0.71981803	0.21982563
	Aldh1a1	0.08380617	-0.3849699	0.15192993	0.64585292	0.19690445
	Akr1a1	0.78156873	0.72472683	0.29129839	0.20599932	-0.066689
	Aldh3a2	0.63043709	0.087697	0.4372669	0.71685142	0.03358944
	Cat	0.52370124	0.06714458	0.13649342	0.5562019	-0.1067881
	Adh1	0.43501624	-0.0346183	0.20058306	0.64791522	0.00300465
	Dhrs4	0.55706093	-0.0192048	0.17581789	0.65880036	0.04057293
	Pecr	0.38297323	0.43107381	0.50880204	2.16325546	-0.0373858
	Acsl1	0.68926196	0.30272098	0.13919103	1.01280886	0.41469853
	Aldh1b1	0.71015076	0.13262631	0.07739299	1.34188546	0.5239206
	Aldh2	1.18504089	-0.0889127	0.23208829	1.18573902	-0.0595114
Ketogenesis	Adh5	0.90616335	-0.4812057	0.17624961	1.0412218	0.58800348
	Bdh1	0.45416173	-0.2733213	0.13125537	0.73198209	0.13796066
	Acat2	0.42986085	-0.4310873	0.19860352	0.58035465	-0.0436768
	Hadhb	0.29851846	-0.4141473	0.06754758	0.57782504	-0.0092558
	Hmgcl	0.41116142	0.09052859	-0.0044019	0.85624777	0.16054035
	Hmgcs2	0.7390597	-0.1716354	0.14655202	0.70974273	-0.0922155
	Hadha	0.62811381	-0.0691577	0.33285223	0.7800026	0.05940517
	Acat1	0.68084577	-0.0682348	0.27490855	0.77847623	-0.0219048
LXR/RXR Activation	Apoh	1.47539416	0.43854214	0.54980067	2.30328647	-0.1264202
	Apoe	2.37187728	0.49577673	1.03006591	1.73417075	-0.4895671
	Ttr	2.23547043	0.06634594	0.75617594	2.05735948	-0.6569957
	Ugt1a2	-0.0339525	0.89133366	-0.4714489	0.24565925	0.23489518
	Apoa1	0.26118002	-0.0162907	0.13869594	0.13997709	-0.2826245
	Kng1	0.46960512	0.20564682	0.35003326	0.01970731	-0.3778936
	Fasn	-0.2210999	0.00071761	-0.1029532	-0.2062429	0.06974327
	Cyp51a1	0.00477061	-0.2638659	-0.3966843	0.47051188	-0.0135173
	Pcyox1	0.22607646	0.16436271	0.09469804	0.46204135	0.07899344
	Pon3	0.16511487	0.12701099	-0.0410528	0.12488366	0.23786766
	Echs1	0.62447406	-0.4274016	0.08450947	0.78037571	0.22494722
	Acaca	0.51882954	0.05382479	-0.1086835	0.96454255	0.39242775
	Pon1	0.56612786	0.08197759	-0.0036575	0.79047282	-0.0377859
	Hadh	0.53874789	-0.0007232	0.19515919	0.80535086	0.10877227
	Ahsg	0.79573775	0.6998419	0.41688778	1.07153247	-0.0068016
	Vtn	0.55020862	0.4125198	0.045599	1.67600242	-0.0644826
	Apoa2	0.67619586	0.9068998	0.26243019	1.73415859	0.02054886
	Apob	1.69962818	0.9257344	1.49131734	0	-0.4077038
	Serpina1d	0.3508946	-0.1064447	0.53323165	0.67904715	-1.1278259

	Alb	0.96418471	0.1585167	0.76032829	0.59161058	-0.2340469
	Cd36	0.88500718	0.04560506	0.626649	0.78153772	-0.0200801
	Tf	0.99618873	0.01424651	0.4286424	1.11858928	-0.1892165
	C3	0.97015065	0.16221772	0.54875305	0.89347408	-0.4065637
	Gc	1.1540105	0.26368379	0.61573531	1.00703676	-0.3618747
TCA Cycle	Mdh1	0.53391523	-0.2613065	0.08351761	0.8483325	0.2513038
	Sdhb	0.61461071	0.09148089	-0.0931304	0.90340387	0.08723484
	Cs	0.59645196	0.20426127	0.19754225	0.90369099	0.24134069
	Sucla2	0.60156198	0.14197488	0.14683396	1.05956669	0.3290605
	Mdh2	0.36299284	-0.1627006	0.24373618	0.3975898	0.25322808
	Fh	0.66594442	0.10130746	0.34457025	0.38445325	0.13992372
	Aco2	0.57051867	0.14764069	0.03381056	0.43888606	0.27624492
	Dlst	0.49605696	0.03489704	0.36514566	0.66520076	-0.0151967
	Dld	0.52744809	-0.0799067	0.34009751	0.8563888	-0.1550641
	Sdha	1.01668639	-0.0511382	0.32328573	0.94563725	0.07642552
	Aco1	0.9365025	-0.0728164	0.73897444	0.63613603	-0.0165435
Acetone Degradation I (to Methylglyoxal)	Cyp2c54	3.50597712	3.95856413	3.57212099	-1.9882706	-3.5044136
	Cyp2d9	1.89826708	2.4496485	-0.9442768	0.34515626	-0.0358218
	Cyp2a4	0.61831091	0.34050813	0.42712651	1.64179312	0.60918206
	Cyp2f2	0.35381182	-0.2029507	0.46128759	0.17446153	-0.9081891
	Cyp2e1	0.70073766	0.28964872	0.05610049	0.88100493	-0.0270627
	Cyp2c29	0.34370556	-0.0588797	0.02003424	0.50115965	0.06096738
	Cyp2c40	0.53670049	-0.0152772	-0.0986735	0.78576218	-0.1064382
	Por	0.54990189	-0.0593997	-0.0688964	0.855203	0.23591646
	Cyp3a11	0.43556598	0.22382802	-0.4015452	0.47802012	0.10786821
	Cyp1a2	0.01009457	0.14843414	-0.0303565	0.30524169	-0.090248
	Cyp51a1	0.00477061	-0.2638659	-0.3966843	0.47051188	-0.0135173
f eIF4 and p70S6K Signaling	Eif2s1	0.86663276	-0.3274842	0.10970412	1.25388318	0.54028761
	Itgb1	0.49223744	-0.1889491	0.6277311	1.33689644	-0.5707726
	Rps7	0.15559219	-0.2243614	-0.2020813	-0.3674061	0.09385874
	Rps26	-0.136342	0.24953633	-0.0193772	-0.260356	0.48230093
	Pabpc1	-0.4271186	-0.0888192	0.20589579	-0.4113162	0.59753091
	Rps12	-0.019435	0.29904746	-0.1075608	0.70319573	0.75404193
	Rps6	-0.039799	0.29510829	-0.1030913	0.58503405	0.56259132
	Rps24	-0.0415941	0.25768207	-0.0956556	0.56754852	0.70175081
	Rps4x	-0.1451373	0.21056872	-0.0654706	0.58850124	0.67968277
	Rps10	0.05636581	0.08970779	-0.1085747	0.34989731	0.31578502
	Rps23	0.07500323	0.05675199	0.01462189	0.43718096	0.38344755
	Rps15	0.08384705	0.12339766	-0.039371	0.32282476	0.46970817
	Rps2	0.025904	0.16692625	-0.0637678	0.57220968	0.49587859
	Rps16	0.02956494	0.16047354	-0.0255819	0.62661957	0.52026901

Regulation of	Rpsa	0.06332635	0.20185257	-0.0034633	0.46643939	0.50575084
	Rps3a	0.05622026	0.22657023	-0.0026124	0.43519195	0.52437182
	Rps18	0.04615437	0.15703095	-0.0252362	0.47184467	0.42234595
	Rps14	0.01086537	0.20648846	-0.0448226	0.47568677	0.41556036
	Rps15a	-0.0422416	-0.0174934	-0.0007557	0.2492216	0.09093094
	Rps5	-0.1827172	0.38173642	-0.1046974	0.27355484	0.6278103
	Rps8	-0.1196617	0.02468098	-0.1072222	0.37475468	0.63473814
	Rps19	-0.1083051	0.18153484	-0.111896	0.35266686	0.47072411
	Rps3	-0.0458204	0.10592339	-0.0122551	0.20939182	0.44691432

Canonical Pathway	Protein	Comparisons				
		OCR/OCL	ORP/OCL	YCL/OCL	YCR/YCL	YRP/YCL
Mitochondrial Dysfunction and ETC proteins	Cat	-0.9431831	1.00626421	-0.5802898	-0.718466	0.9338827
	Prdx5	-0.162192	0.39015903	-0.6095418	-0.099245	0.46049685
	Cyb5r3	-0.220262	0.21694766	-0.346214	-0.1307192	0.11687195
	Cyb5a	-0.1920695	0.12950765	-0.0223432	0.0266798	0.15943263
	Gpd2	-0.1734433	0.11852287	-0.0603059	-0.2735884	0.23835533
	Ndufb7	0.28396888	-0.1430223	-0.2265212	0.56928144	-0.1040808
	Uqcrfs1	0.41423726	-0.4773852	0.19005957	0.20351153	-0.4642351
	Ndufs3	0.41661848	-0.4571055	0.05814706	0.24745126	-0.3236551
	Ndufb10	0.40619248	-0.4443507	0.12624234	0.18731659	-0.3361303
	Ndufs2	0.43644297	-0.3541534	-0.0118008	0.34345645	-0.2475638
	Hsd17b10	0.53927889	-0.4027925	0.23814975	0.32493052	-0.2466067
	Ndufs5	0.30390932	-0.5606832	-0.0163584	0.31898357	-0.1463675
	Ndufa8	0.27334166	-0.4840133	0.03061853	0.36755675	-0.1437332
	Ndufa2	0.33144503	-0.455728	0.00476119	0.31951979	-0.1418241
	Uqcrq	0.24848196	-0.3807613	0.18403012	0.25977926	-0.1878328
	Ndufb9	0.2638446	-0.4190427	0.17628008	0.30268923	-0.24348
	Sdha	0.2829107	-0.4319632	0.06104314	0.28967533	-0.2064378
	Ndufs6	0.35312496	-0.4269774	0.08946668	0.2668503	-0.1725609
	Ndufv2	0.43866916	-0.7097538	0.03286713	0.13076546	-0.7103589
	Ndufb8	0.33048557	-0.6672257	-0.1913861	0.26349077	-0.6640923
	Ndufa9	0.13928368	-0.5373211	-0.1467541	0.33113745	-0.2936577
	Ndufa12	0.09062392	-0.5068104	-0.0431829	0.17620951	-0.3724998
	Atp5j2	0.16406395	-0.6675933	0.18871711	0.11705119	-0.3955292
	Ndufa3	0.36423716	-0.5765463	-0.0013189	0.27237796	-0.4810818
	Ndufs1	0.32301231	-0.4992966	0.04610713	0.29446827	-0.3052336
	Ndufv1	0.28793761	-0.5699507	0.09699533	0.24147832	-0.3417534
	Sdhb	0.24826714	-0.5896165	0.02161893	0.25898436	-0.327451
	Mtco2	-0.1300972	-0.3913286	0.07562188	-0.0300932	-0.3812873
	Cox7a2	-0.1038674	-0.4426759	0.0290307	0.0085891	-0.3196796
	Cox6b1	-0.1574477	-0.3315589	0.27273406	-0.127029	-0.2641811
	Cpt1a	-0.0235439	-0.1593926	0.2798281	-0.1505503	-0.2262584
	Cox5a	0.10940385	-0.3770183	0.24691648	-0.3046517	-0.3402734
	Cox4i1	0.00158374	-0.3608804	0.20713473	-0.0782663	-0.3413479
	Cox6a1	0.01548033	-0.2463432	0.0907679	-0.1855565	-0.3524077
	Atp5d	0.12292725	-0.7628673	0.08524638	-0.1091109	0.01434041
	Maob	0.04083214	-0.3857748	0.17247869	0.29514906	-0.2396124
	Atp5b	0.21844719	-0.4689914	0.12868865	0.13375486	-0.1574161
	Atp5i	0.07846249	-0.4533587	0.15358034	0.19643733	-0.1062759

	Atp5c1	0.16480937	-0.4860901	0.11527703	-0.0219404	-0.1493502
	Atp5j	0.06015378	-0.4190995	0.25777338	0.03859863	-0.0699359
	Atp5a1	0.24833166	-0.4297523	0.25511588	0.08127304	-0.1723401
	Uqcrc1	0.2316552	-0.369837	0.29680177	0.16473894	-0.1963122
	Uqcrc2	0.29150412	-0.3391582	0.29940511	0.12464427	-0.1468413
	Cyc1	0.31022204	-0.3067711	0.35232076	0.21888268	-0.2101935
	Uqcrb	0.28748433	-0.4291687	0.34527231	0.17174589	-0.2748763
	Uqcrh	0.32294343	-0.4035471	0.35214645	0.16291683	-0.2630016
	Ndufs8	0.22247284	-0.1893686	0.01084322	0.21215989	-0.107006
	Ndufa5	0.40111319	-0.0700823	0.36143903	-0.0297153	-0.2885434
	Aifm1	0.27607752	-0.255653	0.25215838	-0.0031965	-0.170939
	Atp5o	0.19313571	-0.1883264	0.2966934	0.15121832	-0.1057077
EIF2 Signaling	Rps12	-0.3299647	-0.1064485	-0.3641305	-0.4499684	-0.0509527
	Rps4x	-0.1600334	-0.0449563	-0.46074	-0.3160467	-0.1050387
	Rpl30	-0.2988538	0.09889674	-0.1781394	-0.4162799	-0.0206526
	Rpl6	-0.268921	0.09302997	-0.2371787	-0.4070014	-0.0243474
	Rps3	-0.2474867	0.01183199	-0.2762479	-0.379998	-0.0038961
	Rpl22	-0.1508626	-0.0095587	-0.2738727	-0.377248	-0.0234394
	Rplp1	-0.1023087	0.01950826	-0.1940048	-0.3469743	-0.0309136
	Rpl3	-0.1746371	0.00121151	-0.1865685	-0.3177737	-0.0988798
	Rplp0	-0.1089524	-0.1695383	-0.3147948	-0.1407729	-0.0000827
	Rps18	-0.1866898	-0.102027	-0.2813934	-0.1658174	0.0746648
	Rpl4	-0.2193947	-0.118349	-0.2029292	-0.2571636	-0.0106111
	Rpl19	-0.1621602	-0.0817715	-0.2360433	-0.2935144	0.01691231
	Rps19	-0.1185381	-0.1732693	-0.2847395	-0.2999869	-0.0287007
	Rps2	-0.179169	-0.1544513	-0.3738153	-0.2614945	-0.0408781
	Rpl27	-0.308662	-0.084588	-0.3765564	-0.1356954	0.08866304
	Rpl10a	-0.2590482	-0.0397329	-0.3565338	-0.2085631	0.08787813
	Rpl11	-0.1626585	0.04397762	-0.3348003	-0.2382129	0.12557613
	Rplp2	-0.117257	-0.0197051	-0.3591312	-0.2464535	0.09001602
	Eif2s1	-0.126785	0.02868102	-0.2201111	-0.3134605	0.12301174
	Rpl18a	-0.2199123	0.0625173	-0.2604013	-0.2332425	0.09735973
	Rpl12	-0.230769	0.0207561	-0.286516	-0.2642796	0.07373367
	Rpl17	-0.076357	-0.0212078	-0.2266812	-0.2627568	-0.1873746
	Rpsa	-0.0975288	-0.1024347	-0.2793948	-0.3271214	-0.1192065
	Rpl24	-0.1614612	-0.0861462	-0.2432079	-0.3110931	-0.1117014
	Rps5	-0.0170275	-0.1009369	-0.1680448	-0.4136438	-0.2567181
	Rpl18	-0.0079584	0.05347297	-0.158656	-0.3252478	-0.1545672
	Rpl9	0.05302447	0.01687501	-0.1605109	-0.3122651	-0.2378376
	Rpl7	0.02075792	-0.3928687	-0.3568599	0.00747957	-0.0664525
	Rps15	-0.0731302	-0.3583325	-0.2501028	-0.3199671	-0.3366073

	Rpl23	-0.0405379	-0.1869407	-0.1640749	-0.2084366	-0.3229659
	Rpl27a	0.11086891	-0.437019	0.08266757	-0.7110645	-0.4465384
	Rps7	0.2680256	-0.1714912	-0.0960343	-0.6480752	-0.3356969
	Rpl14	0.09722213	0.23969961	0.01079149	-0.4693959	-0.3354577
	Rps11	0.03532111	0.24445133	-0.0389111	-0.495037	-0.1432719
	Rps8	-0.1289496	0.15371301	0.05031729	-0.3018826	-0.0032755
	Rps24	-0.204768	0.08303373	-0.0876548	-0.5437842	-0.3205926
	Rpl15	-0.2024625	-0.068817	0.01517313	-0.5039017	-0.1413596
	Rps26	-0.1960041	0.04216666	-0.0210384	-0.4662993	-0.1321122
	Rps3a	-0.0655772	0.05183138	-0.0280296	-0.5415142	-0.1039854
	Rpl8	0.00895175	0.00541338	-0.0381535	-0.4165212	-0.1848636
LPS-1 mediated inhibition of RXR function	Cyp3a16	-1.102931	0.27718637	-1.3774815	-1.1604426	-1.1346799
	Cyp2a4	-0.8375981	-0.0780198	-1.8351868	-1.4498792	-0.4872766
	Cat	-0.9431831	1.00626421	-0.5802898	-0.718466	0.9338827
	Fabp5	-0.4478974	0.81489146	-1.2718018	-0.4317198	0.94905823
	Gstp1	0.01438003	0.46931159	-0.9636578	0.62515102	0.73148653
	Gstm1	0.17830721	0.35080265	-0.4019336	0.61242517	0.45904585
	Fmo5	0.4472248	-0.0810201	-0.3600339	0.64103533	0.34505009
	Gstm2	-0.4639091	0.00056478	-1.1321115	-0.2459041	0.26427693
	Acsl5	-0.0865226	0.33161294	-0.1735398	0.03030639	0.23109853
	Aldh1l1	0.05588816	0.00269037	-0.1582852	0.2359507	0.27341608
	Aldh7a1	-0.279811	0.00277116	-0.5336894	0.1249223	0.51253931
	Aldh8a1	-0.2444507	-0.1066792	-0.2789349	-0.1855506	0.17461651
	Aldh4a1	-0.5021872	0.05206391	-0.3625751	-0.1999038	0.17185741
	Cyp4a14	0.93369558	-0.1748836	0.98965685	0.77816212	-0.5066569
	Cyp4a10	1.63204815	0.48920373	1.37358548	1.03705426	-0.5264025
	Fmo3	1.68715265	-0.0480365	0.73085235	1.05488581	0.01457076
	Fabp1	-0.8069125	0.5404609	0.50468962	-1.2220462	0.54263058
	Aldh3a2	-0.2928949	0.26225027	0.14789831	-0.1422882	-0.0980015
	Cyp2a12	-0.0700625	0.0154031	0.00842597	-0.1678433	-0.128659
	Fmo1	-0.3563692	0.22026129	0.40002765	-0.2076448	0.22900204
	Acox1	-0.3589628	0.43103951	0.19586672	-0.3697839	0.17377151
	Slc27a2	-0.4079817	0.41312205	0.3598053	-0.5097146	0.15192386
	Maob	0.04083214	-0.3857748	0.17247869	0.29514906	-0.2396124
	Cpt2	0.1562912	-0.4170633	0.27466347	0.19588079	-0.5999812
	Abcb11	0.05234127	-0.4202805	0.38084932	-0.3172429	-0.162572
	Cpt1a	-0.0235439	-0.1593926	0.2798281	-0.1505503	-0.2262584
	Slc10a1	0.32567578	-0.4490268	0.83555049	-0.0120079	-0.1398674
	Hmgcs2	0.27729717	0.02501758	0.65903181	0.06678549	-0.2944871
	Acsl1	-0.0162175	-0.0148536	0.57634402	-0.0887483	-0.0650987
	Cyp2c29	-0.4042005	-0.2705702	-0.1759605	-0.2196545	-0.4264451

	Sult2a1	-0.2282704	-0.4565025	-0.23736	0.02220625	-0.9776695
	Gstt1	-0.4242678	-0.1356127	0.7715633	-0.1358809	-0.5058186
	Slco1b2	-0.4080587	-0.0712267	0.73210377	-0.7845984	-0.2627216
	Papss2	-0.3260839	0.03736082	0.38195435	-0.7893297	-0.5098744
Glycolysis and Gluconeogenesis	Agl	-1.887167	0.71612891	1.12953127	-1.8438737	0.47546851
	Pygl	-1.2234931	0.42550776	0.78345995	-1.5160454	0.77788352
	Hsd17b10	0.53927889	-0.4027925	0.23814975	0.32493052	-0.2466067
	Acsl1	-0.0162175	-0.0148536	0.57634402	-0.0887483	-0.0650987
	Adh1	-0.3106024	0.40850325	0.07901143	-0.4443368	0.39300418
	Pklr	-0.1199004	0.37243528	-0.0170187	-0.3418783	0.87939775
	Dhrs4	-0.3443946	0.39915887	-0.0731095	-0.2778277	0.02380903
	Aldh3a2	-0.2928949	0.26225027	0.14789831	-0.1422882	-0.0980015
	Fbp1	-0.3811603	0.12281748	-0.1436946	-0.3657564	0.27721139
	Aldh4a1	-0.5021872	0.05206391	-0.3625751	-0.1999038	0.17185741
	Pkm	-0.3735588	0.25204499	-1.4927163	-0.5074049	-0.1951295
	Aldh2	-0.3227039	0.43290204	-0.6657573	0.06831833	0.75689042
	Me1	-0.230212	0.23262657	-0.2938878	-0.111241	0.55660934
	Aldh7a1	-0.279811	0.00277116	-0.5336894	0.1249223	0.51253931
	Aldh1a7	0.18740476	-0.2789855	-0.7117303	0.3495208	0.19636396
	Aldob	0.34003219	0.04466669	-0.0576509	0.33577835	0.47840624
	Mdh2	0.01120011	0.00545257	-0.1994727	0.1112211	0.00738848
	Akr1a1	0.00383992	0.15981215	-0.3026326	0.1327345	0.24255949
	Gapdh	0.09394361	0.14649993	-0.3253093	0.14409657	0.1778036
	Gpi	0.255497	0.25920157	-0.4070002	-0.0051089	0.55772372
	Pgk1	0.05728043	0.15713093	-0.5319037	0.11131054	0.25364895
	Tpi1	0.24579729	0.11849148	-0.522068	0.21134907	0.35435431
Fatty acid beta oxidation	Acaa1b	-0.7300709	0.63555076	0.96813671	-0.4261808	0.12287927
	Acsl5	-0.0865226	0.33161294	-0.1735398	0.03030639	0.23109853
	Acaa1a	-0.3842085	0.75652636	-0.1424276	-0.3627895	0.27403413
	Hsd17b4	-0.4351782	0.51208458	0.12222804	-0.3339597	0.22971315
	Slc27a2	-0.1980519	0.35750382	0.19397412	-0.2099506	0.12331986
	Sds	0.21057082	-0.9576634	0.26519457	0.29836934	-1.0947514
	Ehhadh	-0.014627	0.42074931	0.74582866	0.13728425	-0.1969669
	Echs1	0.26516761	-0.0026913	0.46680424	0.23849083	0.1678463
	Eci2	0.36595025	0.16798578	0.45842385	-0.2096689	0.00623222
	Acsl1	-0.0162175	-0.0148536	0.57634402	-0.0887483	-0.0650987
	Hsd17b10	0.53927889	-0.4027925	0.23814975	0.32493052	-0.2466067
	Hadha	0.09639062	-0.182346	0.12436669	0.0072056	-0.0515677
	Acaa2	-0.0971577	-0.0605315	0.12503319	-0.0274744	0.13907311
	Cyp3a16	-1.102931	0.27718637	-1.3774815	-1.1604426	-1.1346799
	Cyp2a4	-0.8375981	-0.0780198	-1.8351868	-1.4498792	-0.4872766

Nicotine Degradation	Fmo5	0.4472248	-0.0810201	-0.3600339	0.64103533	0.34505009
	Por	0.37029396	0.01725351	-0.4327861	0.46877442	-0.0971298
	Fmo3	1.68715265	-0.0480365	0.73085235	1.05488581	0.01457076
	Cyp2e1	0.60368426	0.15325427	1.00510129	0.10299988	-0.1208269
	Ugt1a6	0.36098603	0.12363457	0.64169704	-0.3656878	0.04071635
	Cyp1a2	-0.8232849	-0.4805444	0.87293836	-0.6562819	-0.4571128
	Cyp2f2	-1.202152	0.41906194	0.73135352	-1.2771843	0.27726412
	Cyp2c37	-0.9281103	0.5745283	-0.5564151	0.21801817	-0.6655353
	Cyp2c29	-0.4042005	-0.2705702	-0.1759605	-0.2196545	-0.4264451
	Ugt2a3	-0.3454645	-0.2532005	-0.2164032	-0.2690317	-0.0812816
	Cyp2c40	0.21073278	-0.2101141	0.00216194	-0.0930175	0.11949171
	Ugt2b17	-0.1984498	-0.0627267	0.02659585	-0.0938558	-0.0110966
	Cyp2d10	-0.1727724	-0.0598146	0.18262568	-0.3678752	-0.0856006
	Ugt1a5	-0.2484413	0.44227751	-0.596234	-0.3915193	0.563396
	Fmo1	-0.3563692	0.22026129	0.40002765	-0.2076448	0.22900204
	Inmt	-0.3400145	0.21877342	0.5095961	-0.3391242	0.66511523
Estrogen Biosynthesis	Cyp3a16	-1.102931	0.27718637	-1.3774815	-1.1604426	-1.1346799
	Cyp2a4	-0.8375981	-0.0780198	-1.8351868	-1.4498792	-0.4872766
	Cyp2e1	0.60368426	0.15325427	1.00510129	0.10299988	-0.1208269
	Por	0.37029396	0.01725351	-0.4327861	0.46877442	-0.0971298
	Hsd17b10	0.53927889	-0.4027925	0.23814975	0.32493052	-0.2466067
	Cyp2c40	0.21073278	-0.2101141	0.00216194	-0.0930175	0.11949171
	Cyp1a2	-0.8232849	-0.4805444	0.87293836	-0.6562819	-0.4571128
	Cyp2f2	-1.202152	0.41906194	0.73135352	-1.2771843	0.27726412
	Hsd17b2	-0.3778441	0.60284676	0.27546099	-0.8301142	0.53058906
	Hsd17b4	-0.4351782	0.51208458	0.12222804	-0.3339597	0.22971315
	Hsd17b12	-0.397593	0.29756758	-0.0744194	-0.2861344	0.12843947
	Akr1c6	-0.1981164	0.16682835	0.15400531	-0.5148179	0.31648334
	Cyp2d10	-0.1727724	-0.0598146	0.18262568	-0.3678752	-0.0856006
	Cyp2c29	-0.4042005	-0.2705702	-0.1759605	-0.2196545	-0.4264451
	Cyp2c37	-0.9281103	0.5745283	-0.5564151	0.21801817	-0.6655353
phan Degradation	Ehhadh	-0.014627	0.42074931	0.74582866	0.13728425	-0.1969669
	Hsd17b10	0.53927889	-0.4027925	0.23814975	0.32493052	-0.2466067
	Maob	0.04083214	-0.3857748	0.17247869	0.29514906	-0.2396124
	Kmo	0.14496847	-0.4512313	0.13791522	0.18137206	-0.1334052
	Hadha	0.09639062	-0.182346	0.12436669	0.0072056	-0.0515677
	Gcdh	0.0406886	-0.1755351	0.04511907	0.22278524	-0.070361
	Aldh1a7	0.18740476	-0.2789855	-0.7117303	0.3495208	0.19636396
	Aldh2	-0.3227039	0.43290204	-0.6657573	0.06831833	0.75689042
	Akr1a1	0.00383992	0.15981215	-0.3026326	0.1327345	0.24255949
	Aldh7a1	-0.279811	0.00277116	-0.5336894	0.1249223	0.51253931

Trypto	Aldh4a1	-0.0974922	0.26523128	-0.2233887	-0.369291	0.09923737
	Hsd17b4	-0.4351782	0.51208458	0.12222804	-0.3339597	0.22971315
	Aldh3a2	-0.2928949	0.26225027	0.14789831	-0.1422882	-0.0980015
	Haao	-0.4107189	0.06994864	-0.2157843	-0.6007605	0.62450498
	Acat1	-0.3422159	0.21674905	0.11582544	-0.1081783	0.64478549
	Acat2	-0.4272646	0.12817404	-0.0658715	-0.2642023	0.33735632
Xenobiotic Metabolism Signaling	Ugt1a6	0.36098603	0.12363457	0.64169704	-0.3656878	0.04071635
	Fmo1	-0.3563692	0.22026129	0.40002765	-0.2076448	0.22900204
	Aldh3a2	-0.2928949	0.26225027	0.14789831	-0.1422882	-0.0980015
	Ugt2b17	-0.1984498	-0.0627267	0.02659585	-0.0938558	-0.0110966
	Hsp90ab1	-0.3826215	-0.0724046	0.19501024	-0.6208933	-0.6924612
	Cyp1a2	-0.8232849	-0.4805444	0.87293836	-0.6562819	-0.4571128
	Gstt1	-0.4242678	-0.1356127	0.7715633	-0.1358809	-0.5058186
	Maob	0.04083214	-0.3857748	0.17247869	0.29514906	-0.2396124
	Sult2a1	-0.2282704	-0.4565025	-0.23736	0.02220625	-0.9776695
	Cyp3a16	-1.102931	0.27718637	-1.3774815	-1.1604426	-1.1346799
	Ftl1	-0.2077937	-0.1981661	-1.3487348	0.05906406	0.10089016
	Hmox1	-0.4474136	-0.1979515	-1.1301941	-0.4540261	-0.1407735
	Gstm2	-0.4639091	0.00056478	-1.1321115	-0.2459041	0.26427693
	Cat	-0.9431831	1.00626421	-0.5802898	-0.718466	0.9338827
	Hsp90b1	-0.2042569	0.44991379	-0.8556221	0.14015511	0.16027294
	Ugt1a5	-0.2484413	0.44227751	-0.596234	-0.3915193	0.563396
	Aldh8a1	-0.2444507	-0.1066792	-0.2789349	-0.1855506	0.17461651
	Esd	-0.3020188	0.13236928	-0.3507887	-0.1588329	0.36545493
	Aldh4a1	-0.5021872	0.05206391	-0.3625751	-0.1999038	0.17185741
	Aldh1l1	0.05588816	0.00269037	-0.1582852	0.2359507	0.27341608
	Aldh7a1	-0.279811	0.00277116	-0.5336894	0.1249223	0.51253931
	Fmo3	1.68715265	-0.0480365	0.73085235	1.05488581	0.01457076
	Ces2e	0.02658297	1.03095587	-0.726079	0.64559307	0.56483613
	Ces1	0.07184036	0.61385672	-0.7095115	0.95982831	0.64177552
	Gstp1	0.01438003	0.46931159	-0.9636578	0.62515102	0.73148653
	Fmo5	0.4472248	-0.0810201	-0.3600339	0.64103533	0.34505009
	Ces1d	0.10427038	0.37567992	-0.3204995	0.57097188	0.40197451
	Gstm1	0.17830721	0.35080265	-0.4019336	0.61242517	0.45904585
	Cyp2c29	-0.4042005	-0.2705702	-0.1759605	-0.2196545	-0.4264451
Glutaryl-CoA Degradation	Hsd17b4	-0.4351782	0.51208458	0.12222804	-0.3339597	0.22971315
	Acat1	-0.3422159	0.21674905	0.11582544	-0.1081783	0.64478549
	Acat2	-0.4272646	0.12817404	-0.0658715	-0.2642023	0.33735632
	Ehhadh	-0.014627	0.42074931	0.74582866	0.13728425	-0.1969669
	Hsd17b10	0.53927889	-0.4027925	0.23814975	0.32493052	-0.2466067
	Hadha	0.09639062	-0.182346	0.12436669	0.0072056	-0.0515677

	Gcdh	0.0406886	-0.1755351	0.04511907	0.22278524	-0.070361
Acetone Degradation I (to Methylglyoxal)	Cyp3a44	-0.1204293	0.13461319	-2.0895633	-0.279066	0.15682355
	Cyp2a4	-0.8375981	-0.0780198	-1.8351868	-1.4498792	-0.4872766
	Cyp1a2	-0.8232849	-0.4805444	0.87293836	-0.6562819	-0.4571128
	Cyp2f2	-1.202152	0.41906194	0.73135352	-1.2771843	0.27726412
	Cyp2c37	-0.9281103	0.5745283	-0.5564151	0.21801817	-0.6655353
	Cyp2e1	0.60368426	0.15325427	1.00510129	0.10299988	-0.1208269
	Por	0.37029396	0.01725351	-0.4327861	0.46877442	-0.0971298
	Cyp2c29	-0.4042005	-0.2705702	-0.1759605	-0.2196545	-0.4264451
	Cyp2c40	0.21073278	-0.2101141	0.00216194	-0.0930175	0.11949171
	Cyp2d10	-0.1727724	-0.0598146	0.18262568	-0.3678752	-0.0856006
Citrulline Metabolism	Ass1	0.7801242	-0.5213852	0.30110321	0.16742057	-0.7976003
	Arg1	0.36954406	-0.3520099	-0.3539101	0.08459715	-0.2499676
	Asl	-0.0252881	-0.3784814	-0.4854439	0.15121012	0.07838398
	Prodh	0.24109514	-0.7152287	-0.0816464	0.4829929	-0.4987594
	Cps1	0.12205013	-0.4791511	0.04969291	0.16108125	-0.4727652
	Otc	-0.1086926	-0.2965107	-0.1498862	0.03734197	-0.2578096
Phenylalanine Degradation	Got1	0.27265475	-0.2936543	-0.4033937	0.23079311	0.09808711
	Maob	0.04083214	-0.3857748	0.17247869	0.29514906	-0.2396124
	Hpd	-0.1188957	-0.3191538	0.05716188	0.04496455	-0.2512194
	Aldh2	-0.3227039	0.43290204	-0.6657573	0.06831833	0.75689042
	Got2	-0.0952933	0.07232592	-0.0814407	-0.0473548	0.22137014
	Slc27a2	-0.1980519	0.35750382	0.19397412	-0.2099506	0.12331986
	Aldh3a2	-0.2928949	0.26225027	0.14789831	-0.1422882	-0.0980015
Urea Cycle	Ass1	0.7801242	-0.5213852	0.30110321	0.16742057	-0.7976003
	Cps1	0.12205013	-0.4791511	0.04969291	0.16108125	-0.4727652
	Otc	-0.1086926	-0.2965107	-0.1498862	0.03734197	-0.2578096
	Arg1	0.36954406	-0.3520099	-0.3539101	0.08459715	-0.2499676
	Asl	-0.0252881	-0.3784814	-0.4854439	0.15121012	0.07838398
BCAA	Bckdha	-0.5210587	-0.830439	-1.0724261	0.83779778	-0.1311156
	Sds	0.21057082	-0.9576634	0.26519457	0.29836934	-1.0947514
	Dbt	-0.2862593	-0.643156	-0.337589	0.48721004	-0.8546886
	Acat1	-0.3422159	0.21674905	0.11582544	-0.1081783	0.64478549
	Acads	-0.6273041	0.17553923	-0.3934166	0.28698247	0.52412617
	Abat	-0.2911824	-0.0668308	-0.3538086	0.3537467	0.16117766
	Dld	-0.4075801	0.14615975	-0.2242712	-0.3619122	-0.4807592
	Mccc2	-0.1564779	-0.1958688	0.31134271	-1.0533514	-0.4674247
	Ehhadh	-0.014627	0.42074931	0.74582866	0.13728425	-0.1969669
	Hsd17b10	0.53927889	-0.4027925	0.23814975	0.32493052	-0.2466067
	Hadha	0.09639062	-0.182346	0.12436669	0.0072056	-0.0515677
	Echs1	0.26516761	-0.0026913	0.46680424	0.23849083	0.1678463

Melatonin Degradation	Cyp3a44	-0.1204293	0.13461319	-2.0895633	-0.279066	0.15682355
	Cyp2a4	-0.8375981	-0.0780198	-1.8351868	-1.4498792	-0.4872766
	Cyp1a2	-0.8232849	-0.4805444	0.87293836	-0.6562819	-0.4571128
	Cyp2f2	-1.202152	0.41906194	0.73135352	-1.2771843	0.27726412
	Cyp2e1	0.60368426	0.15325427	1.00510129	0.10299988	-0.1208269
	Ugt1a6	0.36098603	0.12363457	0.64169704	-0.3656878	0.04071635
	Ugt1a5	-0.2484413	0.44227751	-0.596234	-0.3915193	0.563396
	Cyp2c37	-0.9281103	0.5745283	-0.5564151	0.21801817	-0.6655353
	Sult1a1	0.28863803	-0.7268405	0.18494659	0.42055801	-1.2072908
	Por	0.37029396	0.01725351	-0.4327861	0.46877442	-0.0971298
	Cyp2c29	-0.4042005	-0.2705702	-0.1759605	-0.2196545	-0.4264451
	Ugt2a3	-0.3454645	-0.2532005	-0.2164032	-0.2690317	-0.0812816
	Maob	0.04083214	-0.3857748	0.17247869	0.29514906	-0.2396124
	Cyp2c40	0.21073278	-0.2101141	0.00216194	-0.0930175	0.11949171
	Ugt2b17	-0.1984498	-0.0627267	0.02659585	-0.0938558	-0.0110966
	Cyp2d10	-0.1727724	-0.0598146	0.18262568	-0.3678752	-0.0856006
RhoGDI Signaling	Rdx	-0.3426938	-0.1063879	0.4209095	-0.4788342	-0.4493783
	Cdh2	0.16610859	0.1539241	0.80194097	-0.5742769	-0.2835879
	Cdc42	-0.5313047	-0.297118	-0.6356495	-0.1533166	0.05440939
	Cfl1	-0.3008211	0.15364051	-0.8167489	-0.3415562	0.02907421
	Arpc5	-0.2874374	-0.0268195	-0.7745607	-0.3973764	-0.0291554
	Gnai2	-0.5560721	0.04850586	-0.7537604	-0.4288366	-0.065455
	Myl12b	-0.5324276	-0.0042447	-0.7124085	-0.5711255	-0.2794192
	Arpc1b	-0.8015407	-0.0379637	-0.5785866	-0.9675864	-0.2137178
	Myl6	-0.5703314	-0.191289	-1.2205716	-0.5187777	0.00636956
	Arhgdib	-0.8833344	-0.1535128	-1.0955959	-0.772158	-0.1222924
	Gnb2	-0.2153542	-0.032023	-0.1838678	-0.326833	-0.3958418
	Actr3	-0.2250836	0.02043143	-0.4341256	-0.5303612	-0.1211116
	Gdi2	-0.1584591	-0.1518914	-0.3675416	-0.2875706	-0.1182102
	Gnb2l1	-0.1213487	-0.0034755	-0.2478216	-0.3337735	-0.0574355
	Itgb1	-0.3514562	-0.6001948	-0.2954969	-0.2832753	-0.6702488
	Arhgdia	-0.1793329	-0.1235898	-0.8778933	-0.249765	-0.4408983
	Msn	-0.0943648	-0.1268315	-0.7175562	-0.5846238	-0.6509967
	Actr2	-0.3542033	-0.0315609	-0.6494078	-0.7291648	-0.4323933
drenaline and e Degradation318	Maob	0.04083214	-0.3857748	0.17247869	0.29514906	-0.2396124
	Hsd17b10	0.53927889	-0.4027925	0.23814975	0.32493052	-0.2466067
	Dhrs4	-0.3443946	0.39915887	-0.0731095	-0.2778277	0.02380903
	Aldh3a2	-0.2928949	0.26225027	0.14789831	-0.1422882	-0.0980015
	Adh1	-0.3106024	0.40850325	0.07901143	-0.4443368	0.39300418
	Comt	-0.0460227	0.1004424	-0.215643	-0.3981089	0.2595244
	Aldh1a7	0.18740476	-0.2789855	-0.7117303	0.3495208	0.19636396

Nora[illegible] Adrenalin[illegible]	Aldh2	-0.3227039	0.43290204	-0.6657573	0.06831833	0.75689042
	Aldh4a1	-0.5021872	0.05206391	-0.3625751	-0.1999038	0.17185741
	Akr1a1	0.00383992	0.15981215	-0.3026326	0.1327345	0.24255949
	Aldh7a1	-0.279811	0.00277116	-0.5336894	0.1249223	0.51253931
Regulation of eIF4 and p70S6K Signaling	Rps18	-0.1866898	-0.102027	-0.2813934	-0.1658174	0.0746648
	Eif2s1	-0.126785	0.02868102	-0.2201111	-0.3134605	0.12301174
	Rps12	-0.3299647	-0.1064485	-0.3641305	-0.4499684	-0.0509527
	Rps3	-0.2474867	0.01183199	-0.2762479	-0.379998	-0.0038961
	Rps4x	-0.1600334	-0.0449563	-0.46074	-0.3160467	-0.1050387
	Rpsa	-0.0975288	-0.1024347	-0.2793948	-0.3271214	-0.1192065
	Rps19	-0.1185381	-0.1732693	-0.2847395	-0.2999869	-0.0287007
	Rps2	-0.179169	-0.1544513	-0.3738153	-0.2614945	-0.0408781
	Rps24	-0.204768	0.08303373	-0.0876548	-0.5437842	-0.3205926
	Rps26	-0.1960041	0.04216666	-0.0210384	-0.4662993	-0.1321122
	Rps3a	-0.0655772	0.05183138	-0.0280296	-0.5415142	-0.1039854
	Rps8	-0.1289496	0.15371301	0.05031729	-0.3018826	-0.0032755
	Rps11	0.03532111	0.24445133	-0.0389111	-0.495037	-0.1432719
	Rps7	0.2680256	-0.1714912	-0.0960343	-0.6480752	-0.3356969
	Rps15	-0.0731302	-0.3583325	-0.2501028	-0.3199671	-0.3366073
	Rps5	-0.0170275	-0.1009369	-0.1680448	-0.4136438	-0.2567181

Table S4: Turnover rate constants and summary statistics for all proteins detected by MS in each group.
For each identified protein, the slope of the natural log-transformed regression between percent newly synthesized peptide vs. time gives the turnover rate. The proportion of each peptide that is newly synthesized is back calculated in Topograph (1) based on the distribution and intensity of single-labeled and multiple-labeled peaks (representing copies of the peptide which have incorporated one or more heavy leucines) compared with the intensity of the unlabeled peptide peak. In contrast to previous metabolic labeling analysis tools, Topograph significantly improves half-life estimates by correcting to label enrichment in the amino acid precursor pool (1). The standard error (SE) and the coefficient of determination (r^2) for each slope estimate are also reported.

		YCL			YCR			YRP		
UniProt ID	geneid	slope	SE	r2	slope	SE	r2	slope	SE	r2
Q61838	A2m Pzp	-0.4042974	0.08712073	0.57373913	-0.3915483	0.06213604	0.72581957	-0.4294311	0.10243588	0.52344768
Q99PG0	Aadac Aada	-0.4985068	0.0292579	0.73438298	-0.2854829	0.02108685	0.63358465	-0.3423912	0.03164414	0.52718355
Q8BGQ7	Aars	-0.2875302	0.03757267	0.84186983	-0.2100025	0.03929849	0.93454638	-0.3110339	0.05450084	0.84443609
Q99K67	Aass Lorsdh	-0.1371654	0.0053392	0.88471686	-0.0749322	0.0025991	0.90034356	-0.1311304	0.01524179	0.48371952
P61922	Abat Gabat	-0.2971995	0.04566647	0.61069674	-0.1274	0.00689823	0.92664755	-0.187312	0.02476726	0.72221251
Q9QY30	Abcb11 Bsep	-0.4979318	0.05339319	0.60408335	-0.3227092	0.0437387	0.49292174	-0.551993	0.05816394	0.62086146
Q61102	Abcb7 Abc7	-0.1672382	0.00528196	0.97757176	-0.088931	0.00346697	0.96764563	-0.1370308	0.01367547	0.83389274
Q9CXJ4	Abcb8	-0.188917	0.11526929	0.17123839	-0.1083478	0.02613319	0.58888883	-0.1398065	0.02300417	0.75477823
Q8VI47	Abcc2	-0.4352898	0.1042251	0.63560368	-0.2727775	0.01898901	0.96267852	-0.262659	0.0443998	0.921045
P55096	Abcd3 Pmp7	-0.5155076	0.02294083	0.76862914	-0.3561951	0.01999885	0.69078268	-0.4387653	0.02766481	0.62333461
Q99LR1	Abhd12	-0.1024909	0.01996632	0.63723991	-0.1140305	0.07561258	0.13165973	-0.189588	0.06953375	0.33137588
Q8VCR7	Abhd14b Cib	-0.1460857	0.00315928	0.98844278	-0.0908719	0.00208512	0.98649579	-0.1350932	0.01247948	0.86684957
Q8R2Y0	Abhd6	-0.170063	0.03280564	0.84312933	-0.1810623	0.02774493	0.91414128	-0.2321309	0.30396311	0.36837142
Q921H8	Acaa1a Acaa	-0.6224707	0.06729286	0.76695353	-0.4406836	0.05586227	0.72168245	-0.6794785	0.07443135	0.76220386
Q8VCH0	Acaa1b Acaa	-0.7023045	0.05403394	0.75777606	-0.5508943	0.03914513	0.78265412	-0.6985026	0.06480203	0.69082092
Q8BWT1	Acaa2	-0.1184006	0.00605031	0.6603209	-0.0756104	0.00542778	0.48996591	-0.1231407	0.01082337	0.40905629
Q5SWU9	Acaca Acac G	-0.7702948	0.06044722	0.72693586	-0.3947305	0.03156974	0.70315222	-0.5868464	0.05770039	0.62524328
E9Q4Z2	Acacb	-0.4540214	0.14688828	0.65644829	-0.481392	0.14138943	0.5368686	-0.4181097	0.1826435	0.36799935
Q80XL6	Acad11	-0.5427307	0.04554408	0.71717938	-0.4200467	0.03174414	0.75767307	-0.6292504	0.05287028	0.71667427
Q8JZN5	Acad9	-0.162146	0.00545774	0.98001439	-0.0951483	0.00497362	0.92893039	-0.1671808	0.01298525	0.91701575
P51174	Acadl	-0.1060648	0.00507511	0.9417813	-0.0669066	0.00339002	0.93293795	-0.1083939	0.01386472	0.69360151
P45952	Acadm	-0.196226	0.02337481	0.79654587	-0.1841691	0.06704565	0.26433442	-0.1601649	0.02304372	0.751202
Q07417	Acads	-0.1381413	0.00360547	0.98589645	-0.0736192	0.00267461	0.96682151	-0.1132953	0.00963754	0.83152281
Q9DBL1	Acadsb	-0.4157387	0.10218624	0.62338353	-0.1989497	0.01217564	0.95357057	-0.3682506	0.10018809	0.62807968
P50544	Acadvl Vlcad	-0.3959391	0.02476597	0.66631154	-0.1879391	0.0154729	0.52778472	-0.3575729	0.03386684	0.52465133
Q8QZT1	Acat1	-0.1229942	0.00498559	0.83530194	-0.0717034	0.00183523	0.92655587	-0.1248759	0.01409904	0.38941876
Q8CAY6	Acat2	-0.1532386	0.0038387	0.98333908	-0.1024859	0.00240342	0.98430153	-0.1579487	0.01338558	0.83758226
Q91V92	Acly	-0.5805581	0.04356118	0.70033922	-0.3754667	0.03960911	0.5228595	-0.5396914	0.05445503	0.56377855
P28271	Aco1 Ireb1 Ir	-0.1828429	0.00417599	0.9922363	-0.1176473	0.00705732	0.95203801	-0.1849516	0.03834576	0.62430079
Q99KI0	Aco2	-0.2259414	0.03160804	0.36472644	-0.1666779	0.0354555	0.18872634	-0.1865683	0.03254344	0.27649711
Q9QYR7	Acot3 Pte1a	-0.423047	0.06606637	0.57748302	-0.410425	0.05942227	0.58387176	-0.4346593	0.07444467	0.5081256
Q8BWN8	Acot4 Pte1b	-0.3107572	0.06932969	0.45567259	-0.3408615	0.08554312	0.37030105	-0.2837059	0.06998218	0.39664106
Q9R0H0	Acox1 Acox F	-0.782637	0.02864644	0.74165688	-0.5244366	0.0184738	0.76540666	-0.8071337	0.02867288	0.75798903
Q9QXD1	Acox2	-0.3844669	0.07468664	0.5463839	-0.2235348	0.0560655	0.38869942	-0.2576714	0.05868365	0.4454664
Q8BP40	Acp6 Acpl1 L	-0.4964571	0.08856698	0.7073455	-0.0954221	0.00629322	0.94260102	-0.2492676	0.07583593	0.45387165
Q8VCW8	Acsf2	-0.244613	0.03488484	0.53346242	-0.1186878	0.023683	0.35820042	-0.1772594	0.03385926	0.38926652
P41216	Acsl1 Acsl2 F	-0.3700856	0.02544611	0.63803625	-0.1834072	0.0095649	0.75393779	-0.2776294	0.02162275	0.58489677
Q8JZR0	Acsl5 Facl5	-0.5283094	0.02481531	0.72144814	-0.2769145	0.01531628	0.64872413	-0.4472914	0.02793261	0.60702962
Q91VA0	Acsm1 Bucs1	-0.2276948	0.04919964	0.29172838	-0.1853506	0.04296045	0.3122475	-0.1618316	0.0234762	0.47748878
P57780	Actn4	-0.3984394	0.02825735	0.72345607	-0.2442288	0.0266407	0.55275875	-0.4684659	0.04377883	0.6139532
P61161	Actr2 Arp2	-0.2752972	0.04078774	0.61102998	-0.0777432	0.00625621	0.88028703	-0.1305497	0.04884303	0.20923358
Q99JY9	Actr3 Arp3	-0.2041296	0.03040845	0.45034814	-0.120247	0.02481791	0.28463752	-0.1848186	0.03559009	0.32899856
Q60936	Adck3 Cabc1	-1.1850055	0.36440925	0.77899821	-0.3751542	0.027345	0.97412266	-0.4159996	0.08690734	0.85137031
P00329	Adh1 Adh-1	-0.1522068	0.00606143	0.89751576	-0.0971387	0.00462637	0.86466974	-0.1518901	0.02072806	0.43061515
P28474	Adh5 Adh-2	-0.2154348	0.02610434	0.68034927	-0.1046832	0.00846395	0.79271442	-0.1433208	0.01057091	0.85172856
P55264	Adk	-0.2002286	0.05504329	0.35540259	-0.1421233	0.01349806	0.86031709	-0.192169	0.03355706	0.63316466
Q8VDL4	Adpgk	-0.5520251	0.24250322	0.56435666	-0.8829184	0.36054453	0.85707857	-0.6895304	0.23960437	0.80547887
Q9ESW4	Agk Mulk	NA	NA	NA	-0.1164382	0.00878983	0.99433367	NA	NA	NA
F8VPN4	Agl	-0.0360116	0.0012471	0.92975306	-0.0279897	0.00224056	0.72904414	-0.0353159	0.0024471	0.77060418
A2AS89	Agmat	-0.1295068	0.00187735	0.99001412	-0.0723083	0.00186533	0.96842116	-0.1131156	0.00797341	0.81396087
O35083	Agpat1	-0.1851208	0.02897641	0.87183636	-0.2205649	0.02037199	0.93611307	-0.163967	0.0616972	0.63843063
Q8K3K7	Agpat2	-0.9186727	0.12761683	0.7873019	-0.7018671	0.10337081	0.74235677	-0.8892337	0.15590779	0.65677998
O35423	Agxt Agxt1	-0.1481498	0.0502673	0.25042285	-0.1741694	0.03534923	0.45566856	-0.1968439	0.05233831	0.44003812
P50247	Ahcy	-0.2267741	0.01675185	0.65388952	-0.1686568	0.01760916	0.50477076	-0.2027286	0.02250864	0.4768418
Q8BK64	Ahsa1	-0.5280285	0.08051677	0.78184706	-0.6192379	0.0768605	0.84397258	-0.4890249	0.14591622	0.55515888
P29699	Ahsg Fetua	-0.6860926	0.08950821	0.68514704	-0.326452	0.07669745	0.54705504	-0.6893349	0.0915528	0.66938798
O70200	Aif1 Iba1	NA	NA	NA	-0.8998782	0.22674052	0.94030222	NA	NA	NA

Q9Z0X1	Aifm1 Aif Pd	-0.1249967	0.00160647	0.9755082	-0.0713578	0.00141426	0.94052007	-0.1142782	0.01092969	0.42484708
Q9R0Y5	Ak1	-0.1730547	0.15644597	0.09253124	-0.1916145	0.09483415	0.21393991	-0.1035851	0.02062992	0.65978858
Q9WTP6	Ak2	-0.1444582	0.01394218	0.86329484	-0.1594083	0.07973018	0.13785341	-0.2203136	0.09560625	0.2380163
Q9WTP7	Ak3 Ak3l Ak3	-0.2988167	0.03494981	0.62963107	-0.120499	0.00446979	0.94536675	-0.3108314	0.05174238	0.48060614
Q9JII6	Akr1a1 Akr1a	-0.1954544	0.03670711	0.42730074	-0.1694469	0.05501562	0.18789789	-0.2047014	0.0511037	0.30247778
Q91WT7	Akr1c14 mC	-0.3504542	0.05174278	0.77918799	-0.1290287	0.00432424	0.98560888	-0.4507616	0.09171158	0.65013455
P70694	Akr1c6 Hsd1	-0.2870607	0.02555533	0.58638949	-0.1421775	0.01151522	0.63138814	-0.2235891	0.02468107	0.4825597
Q8VCX1	Akr1d1	-0.1743486	0.0082754	0.96731123	-0.0929905	0.00849488	0.85696829	-0.1471044	0.01761113	0.7440581
Q8CG76	Akr7a2 Afar	-0.111445	0.0187147	0.76324406	-0.1055761	0.020458	0.67198269	-0.1656423	0.02985644	0.73671517
P10518	Alad Lv	-0.2026867	0.00451136	0.99458002	-0.1296262	0.00277564	0.9954359	-0.1858203	0.02845181	0.81008323
P07724	Alb Alb-1 Alb	-0.2257114	0.00259805	0.97067776	-0.1497825	0.00389592	0.85931019	-0.2654659	0.01662359	0.52042406
Q61490	Alcam	-0.6592165	0.08798532	0.84879472	-0.6880864	0.30177584	0.72218237	-0.5766259	0.18069879	0.71797315
P24549	Aldh1a1 Ahd	-0.1820973	0.00941988	0.92568625	-0.1163811	0.00560213	0.93298405	-0.1588654	0.01423838	0.79046402
O35945	Aldh1a7 Aldh	-0.1484177	0.04148004	0.31376662	-0.1142885	0.03439768	0.26899587	-0.3257616	0.07253903	0.41869752
Q9CZS1	Aldh1b1 Aldh	-0.1563919	0.02784954	0.48119315	-0.0616974	0.00849949	0.56846511	-0.1087674	0.03768403	0.19680077
Q8R0Y6	Aldh1l1 Fthf	-0.2451073	0.00944093	0.74973169	-0.124365	0.00204159	0.94115738	-0.2019543	0.01244782	0.54136034
P47738	Aldh2 Ahd-1	-0.4638306	0.0241745	0.77155176	-0.2038996	0.00806867	0.84965413	-0.4833637	0.03596033	0.62587839
P47740	Aldh3a2 Ahd	-0.2749869	0.01680656	0.79507653	-0.1673086	0.00478358	0.94295818	-0.2686584	0.02685127	0.59197813
Q8CHT0	Aldh4a1	-0.1308938	0.00901427	0.837206	-0.0675441	0.00228239	0.95216238	-0.1054469	0.00706922	0.8444005
Q8BWF0	Aldh5a1	-0.1109823	0.01142497	0.94021645	-0.1320229	0.12753751	0.11812449	-0.1419345	0.12963572	0.11753844
Q9EQ20	Aldh6a1	-0.166433	0.00693977	0.90983283	-0.1168441	0.01596705	0.47579158	-0.1779069	0.02184577	0.5558199
Q9DBF1	Aldh7a1 Ald7	-0.1178334	0.02316488	0.37030226	-0.0652836	0.0033587	0.89781431	-0.0938274	0.00763707	0.77828262
Q8BH00	Aldh8a1 Rald	-0.2272993	0.02037805	0.66032334	-0.1519573	0.02495426	0.36325183	-0.2233658	0.03415685	0.39682976
Q9JLJ2	Aldh9a1	-0.1797037	0.00675426	0.95032749	-0.1431813	0.02376696	0.46955244	-0.2194995	0.0322311	0.54321056
P05064	Aldoa Aldo1	-0.1866254	0.04171232	0.27414796	-0.1258036	0.02466897	0.3514095	-0.2054328	0.04697843	0.29821722
Q91Y97	Aldob Aldo2	-0.4309616	0.01664617	0.81514537	-0.2277958	0.0122514	0.69044357	-0.420246	0.02622902	0.63430607
O09174	Amacr Macr1	-0.5501945	0.08799715	0.69693003	-0.2938908	0.10652367	0.33662554	-0.2461344	0.06496719	0.48898696
Q9DBA8	Amdhd1	-0.1495072	0.01040621	0.89583959	-0.1327605	0.01460195	0.76073016	-0.2103462	0.03894716	0.5287187
Q8CFA2	Amt	-0.2040284	0.01355615	0.98264801	NA	NA	NA	-0.1834314	0.0110882	0.98559435
P00688	Amy2 Amy2a	-0.2119709	0.06045978	0.33869562	-0.0881295	0.01852535	0.53086124	-0.2171613	0.06215118	0.33717413
Q02357	Ank1 Ank-1	-0.018839	0.00512487	0.57470252	-0.0215677	0.00274415	0.8261379	-0.0372983	0.0171088	0.26771634
Q6P9J9	Ano6 Tmem1	-0.3805351	0.14619243	0.45856222	-0.4302153	0.16407098	0.69621981	-0.3442511	0.16977413	0.2913616
P97449	Anpep Lap-1	-0.7245156	0.06568671	0.75256397	-0.4782856	0.05809642	0.61182919	-0.7624337	0.06989819	0.7531326
P10107	Anxa1 Anx1	-0.1776728	0.04841628	0.31711103	-0.0614913	0.0346153	0.0923908	-0.1144587	0.04364036	0.19722325
P07356	Anxa2 Anx2	-0.0844553	0.01410811	0.49885643	-0.0516782	0.00887477	0.47154591	-0.1227599	0.03137298	0.29269085
P97429	Anxa4 Anx4	-0.3492931	0.08637895	0.46254425	-0.2008724	0.04695902	0.5041044	-0.1918072	0.05501212	0.40311632
P48036	Anxa5 Anx5	-0.1258353	0.00344019	0.98818275	-0.069765	0.00437847	0.94773804	-0.1290748	0.01512665	0.81071386
P14824	Anxa6 Anx6	-0.1718714	0.02094661	0.57383513	-0.0976414	0.00459824	0.89660057	-0.1631501	0.02395108	0.49152922
O70423	Aoc3 Vap1	-0.421314	0.20494192	0.34566755	-0.3856328	0.21063184	0.40133871	-0.518062	0.24027429	0.43656076
O35643	Ap1b1 Adtb1	-0.1783378	0.00309168	0.99610818	-0.1252685	0.00467408	0.98762504	-0.1564568	0.02780942	0.79824619
P17426	Ap2a1 Adtaa	-0.1755597	0.00892581	0.98472741	-0.1122364	0.01716775	0.95529789	-0.1376641	0.01734252	0.91305746
P17427	Ap2a2 Adtab	-0.163046	0.0093538	0.92117379	-0.1108662	0.00426145	0.97129888	-0.1577941	0.01455855	0.84834797
Q9DBG3	Ap2b1 Clapb	-0.166377	0.00419883	0.99682562	NA	NA	NA	-0.1697417	0.00495387	0.99660456
P12246	Apcs Ptx2 Sa	-0.2244871	0.09071618	0.20328487	-0.3563994	0.11087675	0.30997662	-0.3325201	0.07439318	0.48754006
P28352	Apex1 Ape A	-0.1407879	0.0203446	0.78649541	-0.0312848	0.00576726	0.69358206	-0.1080502	0.08735278	0.10530074
Q9D7N9	Apmap	-0.5771904	0.07908281	0.81614531	-0.2859975	0.02629184	0.94414599	-0.3389198	0.09143042	0.57878447
Q00623	Apoa1	-0.129653	0.0303472	0.6033417	-0.1176645	0.01796529	0.81095171	-0.1577107	0.02444777	0.76196757
P09813	Apoa2	-0.5740685	0.12055558	0.54409433	-0.172556	0.0136174	0.90938577	-0.5659498	0.12326002	0.56852354
E9Q414	Apob	-0.2222582	0.09006262	0.35635347	NA	NA	NA	-0.2948416	0.05273665	0.77643847
P33622	Apoc3	-0.7185977	0.11689824	0.77453543	-0.6607835	0.09947187	0.75915373	-0.8990726	0.14007542	0.77442339
P08226	Apoe	-0.4831992	0.11124526	0.59204699	-0.1452409	0.00580251	0.97814329	-0.678423	0.1269082	0.68732969
Q01339	Apoh B2gp1	-0.6679191	0.11975841	0.7387508	-0.1353211	0.0225369	0.76622169	-0.7290884	0.2014189	0.6209011
Q78IK4	Apool Fam12	-0.19692	0.01493682	0.92055315	-0.0797186	0.01265132	0.70020402	-0.1338457	0.02278321	0.64494437
Q02013	Aqp1	-0.1562812	0.00614628	0.98028903	-0.1017112	0.00273469	0.98998083	-0.1412665	0.01467858	0.87691861
Q5XJY5	Arcn1 Copd	NA	NA	NA	NA	NA	NA	NA	NA	NA
P62331	Arf6	-0.0953455	0.00277118	0.97850841	-0.0587576	0.00186043	0.97554959	-0.0984075	0.00999515	0.81502383
A2A5R2	Arfgef2 Arfge	-0.5879837	0.17404582	0.58790696	-0.5372493	0.17053682	0.49810924	-0.1643371	0.14909195	0.09193839
Q61176	Arg1	-0.1629128	0.00179523	0.98140901	-0.0966075	0.00142483	0.96555505	-0.1634406	0.01477961	0.43320875
Q99PT1	Arhgdia C872	-0.1186615	0.00426095	0.98351395	-0.0672876	0.00443227	0.94273372	-0.1120693	0.01956879	0.71614389
Q61599	Arhgdib Gdid	-0.6125942	0.08721117	0.76686452	-0.2419931	0.05569351	0.46183651	-0.5722624	0.13529505	0.4984769
Q8R5J9	Arl6ip5 Aip5	-0.3987389	0.08918632	0.66653905	-0.1532065	0.03229173	0.69240049	-0.3149774	0.10407361	0.53378917
Q9WV32	Arpc1b	-0.1453384	0.00401264	0.99093586	-0.1012365	0.00587517	0.95497173	-0.1460653	0.01353354	0.90660416
Q9CVB6	Arpc2	-0.18239	0.01603738	0.88383271	-0.1623621	0.07322092	0.25992433	-0.5581802	0.10631099	0.67954404
Q9CPW4	Arpc5	-0.1843274	0.00577109	0.98741713	-0.0919274	0.00312331	0.98409605	-0.1680075	0.01828484	0.86656508
Q9WV54	Asah1 Asah	-0.1160921	0.00792151	0.95127959	-0.0666869	0.00867983	0.86770191	-0.085539	0.01706321	0.75853282
P34927	Asgr1 Asgr-1	-0.507168	0.08538444	0.67483674	-0.3170568	0.07742946	0.62640906	-0.4578929	0.1428366	0.46131777

P24721	Asgr2 Asgr-2	-0.4829368	0.20527717	0.47983342	-0.360496	0.0823027	0.95045944	-0.4036194	0.08409322	0.74224144
Q91YI0	Asl	-0.1937184	0.03510833	0.38322401	-0.1326115	0.02591671	0.35776542	-0.2031463	0.02954621	0.52366782
Q8BSY0	Asph	-0.3458099	0.05743113	0.57316318	-0.214481	0.03553767	0.55674453	-0.1810184	0.01587686	0.82801655
P16460	Ass1 Ass	-0.2336153	0.0117469	0.6993856	-0.1962129	0.01629449	0.44615708	-0.2611474	0.02122428	0.48932251
Q925I1	Atad3 Atad3a	-0.1500056	0.00351809	0.99398588	-0.1180613	0.02061584	0.76632988	-0.2174683	0.02997214	0.86808459
Q6PA06	Atl2 Arl6ip2	-0.9175672	0.07843891	0.91324073	-0.5467317	0.07541575	0.7896516	-0.8687306	0.12827333	0.77916206
Q91YH5	Atl3	-0.4990824	0.0566057	0.74936463	-0.2505412	0.02926714	0.71647033	-0.4709033	0.06200893	0.68111748
O08997	Atox1	-0.0464085	0.05065426	0.1734495	-0.1208555	0.02091409	0.82670252	-0.1774726	0.02095734	0.93482092
Q9QZW0	Atp11c	-0.2240221	0.02192031	0.88931002	-0.2407166	0.05920715	0.54142946	-0.4040066	0.10671151	0.52439434
Q8VDN2	Atp1a1	-0.1932987	0.00661211	0.93953579	-0.1915781	0.02707215	0.49058529	-0.1732846	0.01597999	0.69748942
P14094	Atp1b1 Atp4	-0.5466889	0.17470803	0.44932978	-0.2482574	0.06280019	0.54588729	-0.6373686	0.13052092	0.66523748
Q8R429	Atp2a1	-0.6806534	0.11929817	0.94211707	-0.6231933	0.119369	0.87202463	NA	NA	NA
O55143	Atp2a2	-0.4802917	0.04458502	0.82862839	-0.2917055	0.0390189	0.75639679	-0.3989726	0.05479534	0.73616618
Q03265	Atp5a1	-0.1155436	0.00109481	0.98045802	-0.0859063	0.00693823	0.39480323	-0.1069169	0.00591228	0.60112221
P56480	Atp5b	-0.1108996	0.00069541	0.98914855	-0.0800321	0.00350787	0.64699661	-0.099569	0.0028267	0.82237116
Q91VR2	Atp5c1	-0.0992601	0.00078314	0.99559977	-0.0619506	0.00235941	0.90306809	-0.0857929	0.0046142	0.83361806
Q9D3D9	Atp5d	-0.102815	0.00136447	0.99579087	-0.0645036	0.00163761	0.98601822	-0.0871219	0.00822462	0.83607492
Q9CQQ7	Atp5f1	-0.1057501	0.00410104	0.93398194	-0.0609962	0.0039159	0.83772295	-0.0927509	0.00780503	0.75834669
Q9DCX2	Atp5h	-0.0988949	0.00165718	0.97348484	-0.0620935	0.00261685	0.84662468	-0.0848003	0.00507102	0.74642557
Q06185	Atp5i Atp5k	-0.1065382	0.00309577	0.98667033	-0.0640006	0.00182753	0.98712192	-0.0848633	0.0086584	0.87280219
P97450	Atp5j	-0.1070903	0.00112974	0.99700418	-0.0601756	0.00109291	0.99052469	-0.0916981	0.00707319	0.86158822
P56135	Atp5j2	-0.1011174	0.00170618	0.99631245	-0.0656176	0.00368074	0.95780741	-0.0831938	0.0095106	0.85477866
Q9CPQ8	Atp5l	-0.1029234	0.00136962	0.99770323	-0.0633618	0.00160834	0.9910602	-0.0878123	0.00978374	0.86104672
Q9DB20	Atp5o D12W	-0.2830152	0.04985464	0.29776678	-0.2027014	0.04409669	0.21532741	-0.2780315	0.05529577	0.25988049
P50516	Atp6v1a Atp6	-0.1211123	0.01575617	0.81965661	-0.0731496	0.01984992	0.49238947	-0.1356169	0.08887659	0.15189973
P62814	Atp6v1b2 Atp	-0.8581148	0.11830787	0.88256889	-0.3357145	0.06319362	0.6684223	-1.1275948	0.16109973	0.84480357
P01887	B2m	-0.6088209	0.16074816	0.58923057	NA	NA	NA	-1.0693019	0.17189769	0.79464226
Q91X34	Baat	-0.3850512	0.12128694	0.45649224	-0.2128014	0.09934165	0.33768326	-0.4712532	0.08669029	0.59637298
O54962	Banf1 Baf Bc	-0.2506833	0.09289476	0.47651858	-0.2511952	0.13561048	0.32893117	-0.1655013	0.00793239	0.98640398
Q61335	Bcap31 Bap3	-0.275842	0.00376718	0.99758118	-0.1542118	0.00326519	0.99376276	-0.2256855	0.02455297	0.86665136
Q03311	Bche	-0.7040482	0.11447681	0.70273593	-0.5924486	0.08338684	0.71622545	-0.6894254	0.0869974	0.74940436
Q6P3A8	Bckdhb	-0.1326463	0.00568344	0.98552597	-0.0706857	0.00263907	0.98489838	-0.1188568	0.01944624	0.82362316
Q80XN0	Bdh1 Bdh	-0.110568	0.00074782	0.99431442	-0.0665703	0.00137577	0.94624926	-0.1004845	0.00532993	0.74446542
O35490	Bhmt	-0.230445	0.00372692	0.97203348	-0.1431962	0.0084623	0.70992428	-0.2093929	0.01289336	0.70948179
D3Z6Q9	Bin2	NA	NA	NA	NA	NA	NA	NA	NA	NA
Q9CY64	Blvra	NA	NA	NA	NA	NA	NA	NA	NA	NA
Q923D2	Blvrb	-0.2410577	0.06231229	0.37446235	-0.2867151	0.07330234	0.39946351	-0.1811591	0.03808623	0.47506313
Q8R164	Bphl	-0.1537805	0.00857906	0.93865197	-0.0746062	0.00542596	0.90867954	-0.0931462	0.01070087	0.77497986
P18572	Bsg	-0.2575726	0.01348893	0.96557414	-0.187613	0.01047294	0.95819824	-0.2457742	0.02186146	0.90673683
Q64152	Btf3	-0.7507965	0.07087752	0.77274125	-0.4217913	0.05777559	0.64761968	-0.8344596	0.08758367	0.75787858
Q9CQC6	Bzw1	-0.7683026	0.12150066	0.7546519	-0.6568234	0.08931016	0.80622271	-0.7731737	0.12782987	0.75300436
P98086	C1qa	-0.3411177	0.10549663	0.48730319	-0.1116402	0.07651141	0.14072671	-0.232036	0.14285218	0.16871168
P14106	C1qb	-0.4345463	0.11431738	0.6161937	-0.2730241	0.15435705	0.3848873	-0.4525322	0.20247402	0.33312372
P01027	C3	-0.5331045	0.03527647	0.57471243	-0.2869788	0.02432274	0.50037988	-0.7066433	0.03923508	0.66419455
P00920	Ca2 Car2	-0.0204544	0.00101151	0.90485019	-0.0212005	0.0023402	0.64586507	-0.0293884	0.00532877	0.41429492
P16015	Ca3 Car3	-0.1413501	0.00203125	0.97759144	-0.0873648	0.00197118	0.94378678	-0.1253334	0.01116493	0.53619955
P62204	Calm1 Calm	-0.1496608	0.00231036	0.99360677	-0.0820134	0.00168572	0.98789649	-0.1472054	0.01120182	0.86479122
P14211	Calr	-0.250962	0.00711917	0.95761653	-0.186042	0.01484678	0.73025879	-0.2088963	0.02211971	0.61855135
P51437	Camp Cnlp C	-0.9828009	0.1421448	0.92278669	-0.6461553	0.19360097	0.78782578	-0.8091913	0.18381446	0.76358893
P35564	Canx	-0.5642064	0.04136081	0.77185931	-0.4033577	0.03815419	0.65449104	-0.5074267	0.05873705	0.57572057
P40124	Cap1 Cap	-0.2774876	0.02032733	0.77856587	-0.1647879	0.01806561	0.61539681	-0.268567	0.04811194	0.41458351
P24452	Capg Mbh1	-0.4983114	0.10901156	0.61647034	-0.3790361	0.09412239	0.51949528	-0.6304742	0.12861505	0.60029827
P24270	Cat Cas-1 Cas	-0.7651792	0.04054342	0.73564272	-0.5203909	0.02902493	0.69964261	-0.8239666	0.03768957	0.79400071
Q91WT9	Cbs	-0.4000334	0.05227475	0.78541064	-0.2482586	0.01164935	0.97635204	-0.3029007	0.03186495	0.84956687
Q71RI9	Ccbl2 Kat3	-0.2833983	0.09352865	0.43346303	-0.0676056	0.00304275	0.95919689	-0.1242249	0.0125406	0.89103317
Q9D024	Ccdc47 Asp4	-0.4056516	0.06011427	0.85056675	-0.1959317	0.01302026	0.97839695	-0.3661885	0.09131544	0.69672411
P80314	Cct2 Cctb	-0.1043167	0.01765154	0.592706	-0.1043555	0.01295977	0.73815722	-0.1401137	0.01973174	0.74786131
P80318	Cct3 Cctg	-0.2294525	0.00532771	0.99624026	-0.1443424	0.00761299	0.98628191	-0.1872362	0.01952413	0.92927001
P80316	Cct5 Ccte Kia	-0.5995596	0.09763013	0.6315742	-0.2551408	0.0735576	0.33390838	-0.4886523	0.14368482	0.34457221
P80317	Cct6a Cct6 C	-0.3684355	0.03659918	0.76575456	-0.2991231	0.0542625	0.49501433	-0.3257228	0.08027597	0.3970617
P80313	Cct7 Ccth	-0.2393657	0.00762012	0.97432692	-0.1632077	0.00845324	0.93714872	-0.2279917	0.01524675	0.91790024
P42932	Cct8 Cctq	-0.5534366	0.0676277	0.84804539	-0.2598155	0.0708718	0.54990894	-0.4910167	0.12021036	0.60266333
Q08857	Cd36	-0.4566872	0.04673419	0.78599463	-0.2656761	0.02987089	0.74553715	-0.463088	0.05163087	0.76291339
Q61735	Cd47	-0.1605509	0.00688815	0.97663027	-0.1004295	0.00577929	0.95872707	-0.1457608	0.01527253	0.8751053
P35762	Cd81 Tapa1	-0.935374	0.12548395	0.76572434	-0.5948745	0.07281493	0.80663165	-0.7620904	0.14850269	0.59400633
P40240	Cd9	-0.6353087	0.17766234	0.7189006	-0.2063388	0.00234027	0.99974279	-0.4416772	0.71947252	0.27371044

P60766	Cdc42	-0.1004949	0.01380376	0.80303673	-0.0714633	0.0106257	0.76364359	-0.0806824	0.01896303	0.60136391
P15116	Cdh2	-0.4547621	0.09765494	0.73051296	-0.1829572	0.02598417	0.94294098	-0.3785312	0.12178526	0.51770603
Q91X79	Cela1 Ela1	-0.3735707	0.1271443	0.41840185	-0.3036031	0.08753006	0.48064191	-0.2992347	0.16669983	0.19862944
P05208	Cela2a Ela-2	-0.2470437	0.11648722	0.24314944	-0.4000072	0.15066493	0.43920822	-0.0946312	0.0606635	0.19571481
Q9CQ52	Cela3b Ela3 E	-0.6358551	0.12934971	0.6681868	-0.752168	0.13805029	0.71213516	-0.6129129	0.21077304	0.43462305
Q9CXS4	Cenpv Prr6	-0.0323209	0.00100786	0.9744177	-0.0164174	0.00059762	0.96299525	-0.0281809	0.0022824	0.85430109
Q8VCC2	Ces1 Ces1g	-0.454859	0.03221349	0.71879575	-0.1993204	0.01800519	0.56854272	-0.2683895	0.02772611	0.53635585
P23953	Ces1c Es1	-0.2634532	0.03193739	0.86084207	NA	NA	NA	-0.3559051	0.04231307	0.8549829
Q8VCT4	Ces1d Ces1 C	-0.3756203	0.0193375	0.72936875	-0.2027915	0.01135356	0.69501084	-0.266704	0.01929557	0.57021162
Q64176	Ces1e Es22	-0.3473876	0.03854975	0.93119703	-0.3205861	0.02023743	0.98046456	-0.3096384	0.05488187	0.86424525
Q63880	Ces3a Es31	-0.4162218	0.03627113	0.81445093	-0.2154759	0.04669397	0.54192541	-0.309071	0.02723395	0.82142159
Q8VCU1	Ces3b Gm47	-0.2874656	0.06877908	0.5927874	-0.1379176	0.01031484	0.93221333	-0.2151557	0.02709315	0.82909301
P18760	Cfl1	-0.3727353	0.0413912	0.68092239	-0.2862123	0.05686479	0.47499857	-0.3647637	0.04489944	0.65999865
Q9D1L0	Chchd2	-0.6640738	0.10104326	0.7970235	-0.1504277	0.01635287	0.88496013	-0.5417445	0.16722137	0.53835635
Q9CRB9	Chchd3	-0.1263882	0.00187115	0.99130895	-0.1177995	0.02281926	0.38819362	-0.1029558	0.00811897	0.8170779
Q8BJ64	Chdh	-0.141789	0.00586034	0.94970655	-0.0828146	0.00373701	0.92468376	-0.1508017	0.03622765	0.35854118
O35744	Chi3l3 Ym1	-0.1432219	0.05227919	0.19491347	-0.0398565	0.00518372	0.59048044	-0.0952238	0.03779265	0.15734313
Q91WS0	Cisd1 D10Ert	-0.1589646	0.00185625	0.99633192	-0.0805335	0.00175957	0.9863451	-0.1410616	0.0111148	0.85643635
Q9CQB5	Cisd2 Cdgsh2	-0.7593063	0.0869149	0.8841537	-0.4994315	0.09904132	0.7841398	-0.5459916	0.15878472	0.66337012
Q8BMK4	Ckap4	-0.450367	0.1006795	0.487932	-0.2072513	0.06728076	0.34518867	-0.3016977	0.10900797	0.27693357
P07310	Ckm Ckmm	-0.1353479	0.03387209	0.61489348	-0.1265852	0.01783669	0.90969188	-0.1347559	0.04264847	0.62461596
Q6P8J7	Ckmt2	-0.122114	0.07924042	0.15446354	-0.0335372	0.01991658	0.20494213	-0.0700275	0.09835833	0.03752838
P70194	Clec4f Clecsf	-0.2124302	0.09454778	0.23983716	-0.0770745	0.05854427	0.08783231	-0.1562326	0.09583296	0.11234153
Q68FD5	Cltc	-0.1546649	0.00236793	0.96233014	-0.0994015	0.00263047	0.89249788	-0.156508	0.01214825	0.51389584
Q8R1G2	Cmbl	-0.1997505	0.00857632	0.99268026	-0.1490835	0.00521093	0.99513689	-0.1776174	0.02490731	0.83566983
Q8CHQ9	Cml2	-0.8750869	0.10208305	0.84968385	-0.732496	0.06294781	0.91240456	-0.8866374	0.14331322	0.7613146
Q9QXT0	Cnpy2 Msap	-0.0725356	0.01860797	0.68461564	-0.0428726	0.00962273	0.73929386	-0.1311982	0.02848089	0.77957521
Q9DBL7	Coasy Ukr1	-0.2834337	0.03321023	0.76001213	-0.1138035	0.00507306	0.96546672	-0.2627326	0.03766418	0.6886493
Q80X19	Col14a1	-0.1302981	0.05421911	0.65812956	-0.1084583	0.01107449	0.96967038	-0.1123937	0.05420781	0.68248581
O88587	Comt Comt1	-0.3465148	0.02950417	0.85708586	-0.2150355	0.00686534	0.98002121	-0.2815374	0.02877465	0.82009895
Q8CIE6	Copa	-0.5481203	0.05481383	0.69928691	-0.3951444	0.05211606	0.59579982	-0.5022151	0.06837372	0.60652577
O89079	Cope Cope1	-0.2857096	0.06015243	0.84939879	-0.1324015	0.09961912	0.46899528	-0.39619	0.06177291	0.95363388
Q9QZE5	Copg1 Copg	-0.6168327	0.11198794	0.70003485	-0.3159584	0.07477136	0.56052523	-0.5482819	0.15446191	0.49218428
O89053	Coro1a Coro	NA	NA	NA	NA	NA	NA	NA	NA	NA
Q9WUM4	Coro1c	-0.2824589	0.0655934	0.45737206	-0.0651342	0.00735911	0.75807299	-0.1617887	0.06174713	0.27610123
Q9CQI6	Cotl1 Clp	-0.1272758	0.01281091	0.93377691	-0.1136287	0.00589959	0.98147972	-0.3634726	0.20003578	0.39770847
P19783	Cox4i1 Cox4	-0.1397661	0.00184876	0.99773058	-0.084714	0.00331576	0.97900254	-0.1261435	0.01416881	0.85909624
P12787	Cox5a	-0.1381642	0.00247773	0.98293	-0.1108496	0.01910195	0.37976025	-0.1858688	0.03796876	0.31546555
P19536	Cox5b	-0.0773045	0.01372349	0.70937249	-0.0621152	0.0051812	0.91123852	-0.1729998	0.07928389	0.26806972
P43024	Cox6a1 Cox6	-0.1644122	0.0105078	0.86258832	-0.1002287	0.0051418	0.90691552	-0.1605082	0.01285187	0.81247799
P56391	Cox6b1 Cox6	-0.154327	0.00456265	0.97694406	-0.1099618	0.00971831	0.81531836	-0.2027599	0.05897203	0.30450873
P48771	Cox7a2 Cox7	-0.1596106	0.00182481	0.99830365	-0.0874354	0.0016967	0.99475581	-0.1421377	0.01615307	0.85624213
Q8BT60	Cpne3 Kiaa0	-0.2124545	0.02746168	0.83298973	-0.1123555	0.01794431	0.7808959	-0.3091648	0.0906387	0.53777817
P36552	Cpox Cpo	-0.3026504	0.05419692	0.53595582	-0.100792	0.00452454	0.94478861	-0.1447503	0.01195903	0.84438323
Q8C196	Cps1	-0.1286241	0.00165877	0.90230755	-0.0784548	0.00244401	0.60279893	-0.1249682	0.00422097	0.58493102
Q6NVF9	Cpsf6	-0.2223536	0.03452023	0.89244942	-0.1034322	0.02796684	0.77373099	-0.146463	0.05553513	0.49840103
P97742	Cpt1a Cpt-1 C	-0.4587512	0.02872026	0.8226605	-0.2698806	0.02933327	0.58927697	-0.435822	0.05150227	0.57009335
P52825	Cpt2 Cpt-2	-0.1545587	0.01707083	0.48227482	-0.0804211	0.00192797	0.94565097	-0.122134	0.00830956	0.72243713
Q9DC50	Crot Cot	-0.2905877	0.04738268	0.6417057	-0.3238362	0.08592194	0.44108144	-0.7006052	0.09567772	0.68201389
P47199	Cryz	-0.1404208	0.02911441	0.36198659	-0.0857805	0.003957	0.90732582	-0.0994667	0.00871628	0.7431869
Q9CZU6	Cs	-0.1417396	0.00254218	0.99138928	-0.0757623	0.00380418	0.9296873	-0.1199058	0.00949501	0.8552078
Q8R311	Ctage5 Mea6	-0.2033603	0.07945166	0.52196137	-0.2701744	0.02959299	0.93284911	-0.3021191	0.04700574	0.7749012
Q8VCN5	Cth	-0.1843983	0.00537079	0.98413751	-0.1211168	0.00229461	0.99181225	-0.1656056	0.01917831	0.79693038
P26231	Ctnna1 Catna	-0.2702505	0.0159573	0.89963086	-0.2121169	0.00946045	0.9526265	-0.340167	0.04077495	0.69878947
P30999	Ctnnd1 Catns	-0.4803464	0.04544624	0.91036174	-0.2840206	0.01942383	0.95106997	-0.4226226	0.07895232	0.72259604
Q9CR35	Ctrb1	-0.3253825	0.10350991	0.28328845	-0.239254	0.08177533	0.24768515	-0.205611	0.09250173	0.17071958
P16675	Ctsa Ppgb	-0.2143183	0.0840448	0.33342726	-0.2800555	0.11543725	0.29597557	-0.1170919	0.02424691	0.64207697
P10605	Ctsb	-0.298969	0.05893956	0.44569472	-0.2298101	0.06340071	0.2472519	-0.4875482	0.07627047	0.54583255
P18242	Ctsd	-0.5752707	0.09390374	0.60994645	-0.4109717	0.09268312	0.43059581	-0.5009202	0.10464312	0.48843419
P49935	Ctsh	-0.5281609	0.08509272	0.73346253	-0.600369	0.08324051	0.85250641	-0.6271244	0.15993157	0.6577664
P56395	Cyb5a Cyb5	-0.3384855	0.01684439	0.88012283	-0.1810262	0.00217643	0.99154393	-0.2539616	0.0291702	0.579504
Q9CQX2	Cyb5b Cyb5m	-0.2430972	0.00652414	0.99072354	-0.1249047	0.00387241	0.98765889	-0.2256175	0.02803475	0.83283353
Q9DCN2	Cyb5r3 Dia1	-0.2888042	0.01730429	0.74369065	-0.143969	0.00234287	0.97545899	-0.2628743	0.02275397	0.58418951
Q61093	Cybb Cgd	-0.1285078	0.03227934	0.46823099	-0.0566178	0.0135506	0.5731794	-0.2099856	0.10087432	0.23636187
Q9D0M3	Cyc1	-0.1175221	0.00113635	0.9936825	-0.062448	0.00123815	0.97247544	-0.0966715	0.00512663	0.84144897
P62897	Cycs	-0.1427463	0.00426677	0.97644515	-0.0943901	0.0079165	0.83057107	-0.1480254	0.01762872	0.73823854

Q7TMB8	Cyfip1 Kiaa0(	-0.3298363	0.12107048	0.55297232	-0.2730306	0.21521261	0.28692218	-0.395868	0.12478833	0.58976973
P27786	Cyp17a1 Cyp	-0.4976796	0.05580077	0.75366233	-0.4197217	0.03321409	0.96963995	-0.4628292	0.05889434	0.7286397
P00186	Cyp1a2 Cyp1	-1.0002421	0.11970558	0.84303359	-0.8095026	0.08035439	0.87877592	-1.0648109	0.1254758	0.84708643
Q9DBG1	Cyp27a1	-0.5875782	0.05100632	0.77286465	-0.3745193	0.0603813	0.49659154	-0.5724696	0.06449947	0.6923774
P56593	Cyp2a12	-0.7288298	0.03921489	0.75186223	-0.4965667	0.02781398	0.74170085	-0.7528607	0.04357097	0.72543639
B2RXZ2	Cyp2a22 mC(	-1.0030606	0.10435326	0.88505054	-0.5480067	0.07633849	0.78636684	-0.9095995	0.11242251	0.85613901
P15392	Cyp2a4 Cyp2	-0.6573691	0.11976107	0.55661616	-0.2106591	0.05585346	0.38214008	-0.4309503	0.08375132	0.56968091
P20852	Cyp2a5 Cyp2	-0.8164748	0.09301548	0.77011595	-0.4897448	0.04935412	0.77859926	-0.6115677	0.10931853	0.6348656
P12790	Cyp2b9 Cyp2	-0.6119532	0.08164972	0.6753751	-0.3566734	0.0478238	0.65730295	-0.6397105	0.09106691	0.64634361
Q64458	Cyp2c29	-0.7348837	0.04031954	0.79817636	-0.5192237	0.03431077	0.75828344	-0.704475	0.04672556	0.74452355
P56654	Cyp2c37	-0.7533684	0.05445744	0.83071573	-0.4901132	0.04198843	0.76011028	-0.6090182	0.06867891	0.6741962
P56657	Cyp2c40	-0.8376206	0.05128679	0.77150279	-0.485858	0.03625812	0.70261319	-0.9017547	0.05090259	0.80093506
Q6XVG2	Cyp2c54	-0.0307022	0.0193138	0.16274808	-0.1218143	0.02391378	0.64954254	-0.3484199	0.12976738	0.35672213
Q5GLZ0	Cyp2c66 mC(	-0.8641407	0.15463164	0.88646047	NA	NA	NA	-0.9742549	0.25684413	0.82746903
Q569X9	Cyp2c67 Cyp	-0.9998802	0.12088858	0.84031624	-0.6579267	0.07848232	0.83388055	-1.0425784	0.1363307	0.81813875
E9PXC3	Cyp2c69	-0.8621197	0.10998041	0.83661808	-0.4047716	0.05968978	0.79305142	-0.8850363	0.12550806	0.79274768
Q91W64	Cyp2c70 Cyp	-0.5285519	0.0711373	0.5621416	-0.3615906	0.05063957	0.58614221	-0.5401841	0.08235644	0.58120434
P24456	Cyp2d10 Cyp	-0.5286488	0.04057674	0.72618973	-0.3141939	0.02215343	0.78527956	-0.5612483	0.05441742	0.65511651
Q8CIM7	Cyp2d26 Cyp	-0.7784155	0.02766662	0.89791391	-0.4554158	0.02735346	0.7467666	-0.7889213	0.05038941	0.74027986
P11714	Cyp2d9 Cyp2	-0.2517485	0.03909709	0.83825802	-0.1981822	0.03602769	0.83452437	-0.2580776	0.06295782	0.70592639
Q05421	Cyp2e1 Cyp2	-0.8702631	0.06987455	0.77127891	-0.4725433	0.05099291	0.734758	-0.8867419	0.0816147	0.73757827
P33267	Cyp2f2 Cyp2f	-0.2005411	0.03201398	0.50153288	-0.1776991	0.03104638	0.52199119	-0.3763532	0.06618664	0.45971566
Q64459	Cyp3a11 Cyp	-0.412424	0.04683065	0.76368218	-0.2961048	0.02277058	0.93373818	-0.3827122	0.08845956	0.52404703
Q64464	Cyp3a13 Cyp	-0.3983147	0.04202936	0.56911478	-0.2849907	0.02665706	0.64104942	-0.3289703	0.03820898	0.53280457
Q64481	Cyp3a16 Cyp	-0.5630989	0.11680302	0.60775436	-0.2476688	0.07629589	0.56844378	-0.4095671	0.14793343	0.4893104
O09158	Cyp3a25	-0.8269206	0.14747812	0.74080608	-0.58619	0.07505316	0.81333646	-1.1208285	0.14637059	0.81852917
Q9JMA7	Cyp3a41a Cy	-0.8703665	0.09362155	0.75530389	-0.4841909	0.06966876	0.67742436	-0.9525861	0.10981858	0.74318807
Q9EQW4	Cyp3a44 cyp	-0.2482549	0.10595192	0.1997111	-0.1071079	0.0855993	0.06938312	-0.0264659	0.01494544	0.14836692
O88833	Cyp4a10 Cyp	-0.5249412	0.07359735	0.67050689	-0.4297314	0.04731248	0.73990531	-0.5339749	0.10080271	0.57195813
O35728	Cyp4a14	-0.5253311	0.06412273	0.58814738	-0.3565486	0.0354065	0.64835525	-0.6247094	0.08451386	0.55960082
Q8K0C4	Cyp51a1 Cyp	-0.6641962	0.08633804	0.62837841	-0.479356	0.05835084	0.66498276	-0.6704486	0.08953125	0.63667987
Q9D172	D10Jhu81e	NA	NA	NA	-0.065467	0.00931606	0.90806002	-0.1505618	0.01907116	0.93969268
Q99LF4	D10Wsu52e	-0.2456654	0.02099518	0.93831997	-0.222888	0.00965274	0.98340027	-0.4742007	0.15469987	0.65268211
P61804	Dad1	-0.1467896	0.00237489	0.99660872	-0.088628	0.00276499	0.98750523	-0.1116549	0.0132413	0.84542956
Q8VC30	Dak	-0.1881951	0.00545385	0.89816868	-0.1339956	0.01081433	0.52844138	-0.1747313	0.008696	0.75785575
P31786	Dbi	-0.1915208	0.00713444	0.94865916	-0.1174386	0.00566906	0.93060693	-0.2043091	0.029475	0.57855275
P53395	Dbt	-0.1944247	0.00724179	0.99174456	-0.0968672	0.00701294	0.9549525	-0.2418656	0.12182512	0.79763719
O54734	Ddost	-0.1776206	0.03388062	0.27351805	-0.1400397	0.02908501	0.2291172	-0.1327275	0.02939522	0.23600249
Q80WW9	Ddrgk1	-0.6709928	0.14290618	0.62906103	-0.5294268	0.1498638	0.50980615	-0.8538149	0.16671756	0.70452328
O35215	Ddt	-0.1472784	0.00495075	0.94249108	-0.1361858	0.02731021	0.31529818	-0.1273147	0.00786019	0.83724601
Q91VR5	Ddx1	-0.2679072	0.01146688	0.9784891	-0.1883679	0.00668296	0.99003074	-0.2554651	0.02668161	0.89286321
Q62167	Ddx3x D1Pas	-0.385911	0.0495759	0.85834588	-0.4053978	0.07400809	0.81084017	-0.4513935	0.05666884	0.82995111
Q61656	Ddx5 Tnz2	-0.5119745	0.16297319	0.52302207	-0.6282697	0.11018345	0.74720308	-0.8492804	0.1643571	0.68992938
Q9CQ62	Decr1	-0.1180846	0.00252855	0.97583829	-0.0691864	0.00255192	0.9303826	-0.1069013	0.00734124	0.80611674
Q9WV68	Decr2 Pdcr	-0.6507684	0.04192508	0.84262315	-0.2973723	0.02753285	0.70847926	-0.4668953	0.04974168	0.65212115
Q8BNI4	Derl2 Der2 Fl	-0.629134	0.07492581	0.84432177	-0.4043348	0.06253456	0.74913245	-0.7047095	0.11477708	0.74357595
P31001	Des	-0.0764579	0.00842117	0.91153646	-0.0342885	0.01128484	0.75474657	NA	NA	NA
O88455	Dhcr7	-0.3334429	0.06248162	0.51333714	-0.2098362	0.040211	0.48427445	-0.3282188	0.07206861	0.43445114
Q9DBB8	Dhdh	-0.1406872	0.00567701	0.98714124	-0.0760167	0.01041492	0.88386171	-0.1265211	0.0215213	0.7585666
Q99L04	Dhrs1 D14er	-0.253052	0.00946484	0.95972151	-0.1492648	0.00541687	0.95714157	-0.2124226	0.02033677	0.79577425
Q99LB2	Dhrs4 D14Uc	-0.1786248	0.0044235	0.97547279	-0.1131418	0.00227392	0.98253757	-0.1736713	0.01169515	0.84646004
Q8BMF4	Dlat	-0.1012496	0.01283751	0.75670574	-0.0668719	0.00639106	0.83267617	-0.1788955	0.05094808	0.39354152
O08749	Dld	-0.1280386	0.01237202	0.72316516	-0.07072	0.00480177	0.82503575	-0.1425673	0.03606988	0.28600837
Q9D2G2	Dlst	-0.0925472	0.00632966	0.94267567	-0.0583604	0.00483374	0.91237438	-0.0935272	0.0117811	0.82900075
Q9DBT9	Dmgdh	-0.1396122	0.00334132	0.93719371	-0.0803654	0.00184745	0.93618001	-0.1249138	0.00508973	0.83618518
Q99M87	Dnaja3 Tid1	-0.2572727	0.03182967	0.85589194	-0.2728868	0.06820256	0.59272805	-0.4224674	0.08566905	0.72988058
Q91YW3	Dnajc3 P58ip	-0.341254	0.05246473	0.8580347	-0.273585	0.04732558	0.94353303	-0.3719578	0.05817035	0.81959187
Q8K1M6	Dnm1l Drp1	-0.186417	0.01875759	0.80450963	-0.1641703	0.01792874	0.77032077	-0.1429556	0.02875839	0.65526419
P31428	Dpep1 Mbd1	NA	NA	NA	NA	NA	NA	NA	NA	NA
P28843	Dpp4 Cd26	-0.0853185	0.00756036	0.73042812	-0.0457294	0.00526353	0.64249543	-0.0784153	0.03747108	0.08868773
Q9EQF5	Dpys	-0.4871949	0.07791466	0.60060913	-0.2145387	0.05514931	0.35084824	-0.3086106	0.06962332	0.42119368
Q9JHU4	Dync1h1 Dhc	-0.1440536	0.02292463	0.63191793	-0.0902059	0.01733933	0.57505423	-0.0556294	0.0238468	0.24248749
P97425	Ear2 Rnase2	-0.7171876	0.17461263	0.77137598	-0.6770362	0.14677713	0.80971832	-0.5757595	0.19809451	0.62818858
Q923L7	Ear6	-0.7569296	0.14894333	0.81147903	-0.4373385	0.34041178	0.24818142	-0.2877179	0.23768641	0.17309435
P70245	Ebp Msi	-0.0833368	0.00796118	0.8939441	-0.0624716	0.00471248	0.92621415	-0.0746164	0.00840481	0.85841228
O35459	Ech1	-0.6678673	0.041628	0.77670516	-0.3311734	0.03784816	0.51885097	-0.6101149	0.05131934	0.65941755

Q9D9V3	Echdc1	-0.1399163	0.03797632	0.87158156	NA	NA	NA	-0.0747976	0.01172043	0.9313934
Q3TLP5	Echdc2 D4Ert	-0.1522566	0.01006058	0.97034364	-0.0729431	0.00500713	0.95499998	-0.0939816	0.04310012	0.48742971
Q9D7J9	Echdc3	-0.1829402	0.01409459	0.96560942	-0.0805393	0.00542803	0.97779324	-0.1324935	0.02232225	0.81494343
Q8BH95	Echs1	-0.1215128	0.0033743	0.93107459	-0.0707466	0.00164523	0.94723628	-0.1039696	0.00533767	0.80484085
P42125	Eci1 Dci	-0.0880175	0.00658061	0.90858224	-0.0501227	0.0033141	0.92704792	-0.0712832	0.00718795	0.83100645
Q9WUR2	Eci2 Peci	-0.1865132	0.01355525	0.72447928	-0.1069984	0.01393783	0.42115487	-0.200072	0.02268334	0.52996058
P10126	Eef1a1 Eef1a	-0.2945959	0.01909333	0.70419633	-0.3792336	0.0300082	0.68336906	-0.1910042	0.01344368	0.6599747
O70251	Eef1b Eef1b2	-0.0747725	0.0689056	0.28187344	-0.0418832	0.02439978	0.37079312	-0.177598	0.03575264	0.89159951
P57776	Eef1d	-0.3023913	0.07737275	0.56002612	-0.17522	0.01191351	0.96005608	-0.1772262	0.02144415	0.84010384
Q9D8N0	Eef1g	-0.0986151	0.00389385	0.98013448	-0.0676802	0.00740942	0.85631629	-0.0644269	0.0095557	0.77761736
P58252	Eef2	-0.630124	0.03593809	0.74722126	-0.5037517	0.05994232	0.60557752	-0.4065888	0.03469188	0.53580606
Q9WVK4	Ehd1 Past1	-0.3289224	0.0643794	0.5787435	-0.2773367	0.05187012	0.62709285	-0.2613335	0.10047869	0.36049805
Q9QXY6	Ehd3 Ehd2	-0.3334402	0.04411492	0.58818121	-0.1209735	0.01227859	0.69799297	-0.504173	0.08085439	0.49291542
Q9DBM2	Ehhadh	-0.5018283	0.02026657	0.7653303	-0.2897492	0.01577411	0.62091072	-0.4665177	0.02652028	0.62584131
Q6ZWX6	Eif2s1 Eif2a	-0.3591364	0.04615395	0.8232451	-0.1505924	0.00618402	0.97693623	-0.2469543	0.08149923	0.41393307
Q9WUK2	Eif4h Wbscr1	-0.8396419	0.06860403	0.94332212	-0.4771847	0.09685502	0.82919586	-0.8303259	0.1311797	0.85126917
P63242	Eif5a	-0.1938793	0.02192904	0.78036683	-0.1359107	0.0124451	0.8752426	-0.2011584	0.02858938	0.70215736
O55135	Eif6 Itgb4bp	-0.2380407	0.0237626	0.94358219	-0.1842222	0.01786164	0.97257151	-0.3189865	0.03974735	0.94152586
Q3UP87	Elane Ela2	-0.1478551	0.02652145	0.88597424	NA	NA	NA	-0.2345966	0.18818112	0.27981698
Q9JLJ4	Elovl2 Ssc2	-1.0137291	0.12243688	0.84059226	-0.6589285	0.07601085	0.8429602	-1.0102956	0.1413952	0.79704545
Q9CRD2	Emc2 Kiaa01	-0.1810912	0.00613186	0.98531386	-0.113711	0.00475036	0.97614977	-0.1382614	0.01800637	0.81934144
P17182	Eno1 Eno-1	-0.1794479	0.01936445	0.48827278	-0.1286239	0.01719457	0.35651504	-0.1480011	0.01574047	0.48467347
P21550	Eno3 Eno-3	NA	NA	NA	NA	NA	NA	NA	NA	NA
P16406	Enpep	-0.5292561	0.09441955	0.61104725	-0.2280046	0.04607017	0.52681379	-0.5293987	0.06991993	0.69633514
Q9WUZ9	Entpd5 Cd39	-0.363983	0.07421616	0.39396697	-0.2371903	0.03574106	0.48375009	-0.3510863	0.07174741	0.38655288
Q9D379	Ephx1	-0.3239779	0.02425237	0.67994211	-0.243624	0.0242616	0.52020483	-0.3012796	0.03348438	0.46807611
P34914	Ephx2 Eph2	-0.294406	0.01341879	0.71274417	-0.1864722	0.01081719	0.59059233	-0.2799781	0.01850316	0.54649429
Q8R0W0	Eppk1	NA	NA	NA	NA	NA	NA	NA	NA	NA
P49290	Epx Eper	-0.2948999	0.15894107	0.27667406	-0.2157344	0.0681116	0.37112066	-0.2567447	0.23223142	0.16923432
Q9EQH2	Erap1 Appils	-0.5493428	0.12891236	0.602112	-0.2109764	0.08590471	0.37623271	-0.509772	0.16157892	0.49883869
P57759	Erp29	-0.1331419	0.01870495	0.8941164	-0.1244635	0.01634073	0.9508319	-0.1192522	0.04701332	0.68200609
Q9D1Q6	Erp44 Kiaa05	-0.0563588	0.00881965	0.56844861	-0.0554184	0.00566483	0.72118584	-0.0808774	0.01039631	0.62701906
Q9R0P3	Esd Es10 Sid4	-0.1412118	0.00334837	0.97106338	-0.0866242	0.00174978	0.97844164	-0.1194161	0.00750845	0.82947709
Q8BWY3	Etf1	-0.2769341	0.03523196	0.87285341	-0.2154283	0.01399004	0.97132553	-0.3600161	0.09112956	0.63425359
Q99LC5	Etfa	-0.2323208	0.0257155	0.41723184	-0.0811345	0.01112992	0.30692215	-0.1406528	0.01744255	0.37151638
Q9DCW4	Etfb	-0.1289665	0.00225646	0.99331025	-0.0680076	0.0015187	0.98817296	-0.1096688	0.01009407	0.83103488
Q921G7	Etfdh	-0.1194955	0.00121702	0.98578699	-0.0701536	0.00109946	0.96469491	-0.1030751	0.00432119	0.80480497
P26040	Ezr Vil2	-0.1506035	0.06399946	0.24570313	-0.0747042	0.01673115	0.52551692	-0.2400143	0.07152528	0.46414815
O08914	Faah Faah1	-0.3224845	0.01955756	0.82169135	-0.1921931	0.00607805	0.95056457	-0.2901724	0.02171562	0.74535929
P12710	Fabp1 Fabpl	-0.1860362	0.01678077	0.60275706	-0.1210446	0.01135482	0.56922427	-0.1607893	0.0157253	0.56043535
P04117	Fabp4 Ap2	-0.0371466	0.00716792	0.56119035	-0.0356089	0.0037726	0.77409275	-0.0475903	0.00686964	0.65749661
Q05816	Fabp5 Fabpe	-0.1746012	0.09389629	0.23916399	-0.1579781	0.090164	0.23488449	-0.2306962	0.13653411	0.19218852
P35505	Fah	-0.1883162	0.02061471	0.51687534	-0.1350712	0.01889139	0.39591475	-0.1610572	0.01505628	0.58552077
Q9CR98	Fam136a	-0.1310487	0.00627614	0.99090896	-0.0869059	0.03772689	0.63883092	-0.2454229	0.08074414	0.7548748
Q921M7	Fam49b	-0.1831609	0.02545351	0.91194227	-0.1546507	0.03615816	0.78534571	-0.3524111	0.06093783	0.84788768
Q8BSE0	Fam82a1 Far	-0.4459243	0.07128239	0.60082485	-0.4328824	0.06359136	0.65879369	-0.3213935	0.06512887	0.50363565
Q3UJU9	Fam82a2 Far	-0.1619922	0.02984297	0.59567254	-0.1334262	0.05837723	0.22493651	-0.1390893	0.01563489	0.80639962
Q9DCV4	Fam82b	-0.0295739	0.01434673	0.26150402	-0.0453777	0.010317	0.61716963	-0.0179263	0.01048596	0.19584822
P19096	Fasn	-0.5826297	0.01490093	0.76409753	-0.6721682	0.02156969	0.79461743	-0.555134	0.01833673	0.66198191
Q9QXD6	Fbp1 Fbp	-0.2324214	0.01324709	0.74928815	-0.1302877	0.00568306	0.83348764	-0.211243	0.01853081	0.56267585
Q920E5	Fdps	-0.2595267	0.01275417	0.94091668	-0.2066319	0.01615614	0.86285182	-0.2639319	0.020028	0.88756209
P46656	Fdx1	-0.2689525	0.00644826	0.99713412	NA	NA	NA	-0.2571918	0.01006123	0.99542996
P22315	Fech	-0.0537809	0.02271747	0.31835609	-0.024038	0.01223211	0.25984859	-0.0508314	0.01855108	0.38486834
Q8K1B8	Fermt3 Kind3	NA	NA	NA	NA	NA	NA	NA	NA	NA
Q8K0E8	Fgb	-0.7823528	0.06598249	0.91535792	-0.3419371	0.08410063	0.55978169	-0.7631385	0.13186567	0.7362183
Q8VCM7	Fgg	-0.7379537	0.04080269	0.89347177	-0.3109728	0.04118186	0.58771702	-0.7575684	0.06201582	0.76830875
P97807	Fh Fh1	-0.1148967	0.00908285	0.79209857	-0.0880189	0.00451742	0.88157125	-0.1042765	0.007226	0.82885315
P97447	Fhl1	-0.3216498	0.05920062	0.6942605	-0.2213254	0.08002037	0.35334878	-0.2210136	0.02747257	0.83273316
Q9D1M7	Fkbp11	-0.1462453	0.02028662	0.72210282	-0.060124	0.01374701	0.47667992	-0.0965672	0.01747261	0.64244584
P45878	Fkbp2 Fkbp1	-0.2719385	0.03238823	0.85453912	-0.1960366	0.09275342	0.28880763	-0.2053791	0.02683611	0.82995539
O35465	Fkbp8 Fkbp3	-0.3291931	0.04734529	0.94157127	-0.3017398	0.01800963	0.98249966	-0.334534	0.05205737	0.85506272
Q8BTM8	Flna Fln Fln1	-0.1927109	0.00604449	0.98833216	-0.1187241	0.00763795	0.96408839	-0.2224716	0.03540419	0.78211655
P50285	Fmo1 Fmo-1	-0.5304785	0.02690438	0.80196677	-0.2507011	0.01492199	0.75418559	-0.4006508	0.03410526	0.61062512
P97501	Fmo3	-0.7232204	0.03994481	0.86082252	-0.4706285	0.04192087	0.70006059	-0.8230459	0.05625021	0.81998659
P97872	Fmo5	-0.4585517	0.0221409	0.79294906	-0.3651826	0.02217962	0.73647676	-0.434763	0.03366973	0.62981799
P11276	Fn1	-0.3689833	0.05307649	0.47695208	-0.2718209	0.04575045	0.43419447	-0.4532069	0.06310335	0.50283098

Q91XD4	Ftcd	-0.2711879	0.02346933	0.77393641	-0.1406001	0.01181503	0.78406823	-0.1966554	0.02722552	0.53691604
P29391	Ftl1 Ftl Ftl-1	-0.1784	0.00904122	0.93067932	-0.1472299	0.00768159	0.91301292	-0.2389672	0.05005542	0.42370169
P97855	G3bp1 G3bp	-0.8627833	0.06354876	0.93411956	-0.5374121	0.07691203	0.77715242	-0.7876039	0.11365055	0.80008525
P35576	G6pc G6pt	-0.3008611	0.03972837	0.83906275	-0.1450737	0.01340316	0.90708882	-0.1860331	0.03096904	0.76637911
Q8K157	Galm	-0.0564221	0.03603783	0.25935422	-0.0129114	0.00185064	0.78921576	-0.0450517	0.01219983	0.66080102
O35969	Gamt	-0.6693676	0.10488701	0.91056923	-0.4852944	0.17652875	0.71584251	-0.7252378	0.16020826	0.71922254
Q8BHN3	Ganab G2an	-0.5137892	0.08796937	0.45414831	-0.2609156	0.04949219	0.41610164	-0.3406956	0.06461837	0.41615442
P16858	Gapdh Gapd	-0.1916378	0.01900601	0.55657026	-0.1815257	0.03138763	0.28238052	-0.1880296	0.02776774	0.36433972
Q9Z0E6	Gbp2	-0.3682664	0.1391103	0.36869305	-0.3016157	0.11296208	0.33741067	-0.1004915	0.14800257	0.04022507
P21614	Gc	-0.6043588	0.05142579	0.6636408	-0.3007091	0.03376724	0.56523378	-0.7766583	0.05840546	0.72225493
O88986	Gcat Kbl	-0.2089045	0.04337482	0.72046545	-0.1117137	0.00954979	0.90718875	-0.4539412	0.15194083	0.59801321
Q60759	Gcdh	-0.2573096	0.01573551	0.68669195	-0.1241864	0.00243866	0.95156435	-0.2579713	0.02178248	0.53480968
O09172	Gclm Glclr	-0.2712749	0.03142279	0.85147905	-0.1921865	0.00621652	0.98556351	-0.3167579	0.07668243	0.6080291
Q61598	Gdi2 Gdi3	-0.4536238	0.03281215	0.70754512	-0.207216	0.01453608	0.7100063	-0.3117286	0.03340539	0.53397208
Q9QYC7	Ggcx	-0.2601222	0.03143851	0.90723444	-0.2181191	0.03234488	0.9009418	-0.2674813	0.04976745	0.85244896
Q99JY3	Gimap4 Ian1	-0.2397359	0.0867426	0.2667183	-0.0895561	0.0153098	0.5877544	-0.6464906	0.13564592	0.58672252
Q00977	Gjb2 Cxn-26	-0.5738507	0.10061517	0.84427321	NA	NA	NA	-0.4356256	0.10204184	0.78471584
Q91W43	Gldc	-0.3152141	0.0239281	0.85260746	-0.1424704	0.00424932	0.97232108	-0.334193	0.05060588	0.59245042
Q9CPU0	Glo1	-0.1975551	0.00592102	0.98845703	-0.1238217	0.00281212	0.99283068	-0.1661047	0.02041745	0.83582789
P26443	Glud1 Glud	-0.154856	0.0036629	0.94202402	-0.1068289	0.00212527	0.95755458	-0.1168179	0.00477616	0.85068696
P15105	Glul Glns	-0.2652855	0.01349394	0.83387271	-0.1658853	0.01192562	0.71532924	-0.2001175	0.01080749	0.81855627
Q91XE0	Glyat	-0.1131699	0.01830829	0.76099923	-0.1205501	0.01567364	0.80862687	-0.1129549	0.02690328	0.57554974
Q5FW57	Gm4952	-0.1427354	0.01077227	0.94104067	-0.1189303	0.08950971	0.63838938	-0.1036119	0.01581484	0.79600547
P08752	Gnai2 Gnai-2	-0.4522367	0.05292582	0.62934989	-0.3680304	0.05871207	0.48334886	-0.4610375	0.06722675	0.52825653
P62874	Gnb1	-0.4814869	0.09187227	0.54425021	-0.2744751	0.11161616	0.28731494	-0.7861815	0.16725472	0.59562998
P62880	Gnb2	-0.2301647	0.02212473	0.73509671	-0.1403847	0.00496323	0.95923445	-0.193546	0.03575505	0.48591876
P68040	Gnb2l1 Gnb2	-0.1193388	0.00308534	0.93093115	-0.0956319	0.00227287	0.9495799	-0.0822009	0.0042773	0.77863502
Q9QXF8	Gnmt	-0.2144032	0.00870051	0.85739669	-0.1118268	0.00219065	0.96020367	-0.2043665	0.01687547	0.58509317
P05201	Got1	-0.1404877	0.0041545	0.95650374	-0.072901	0.00327349	0.89854289	-0.1177729	0.00780223	0.82004827
P05202	Got2 Got-2	-0.1087417	0.00286198	0.93280101	-0.0639756	0.00189406	0.9062666	-0.0946171	0.00449593	0.80249866
Q61586	Gpam Gpat1	-0.8254946	0.07860835	0.79178417	-0.6759356	0.07421563	0.72795245	-0.7451881	0.09075769	0.69204332
P13707	Gpd1 Gdc-1 (	-0.2035111	0.00653568	0.9748644	-0.1238431	0.00253833	0.98878455	-0.1803064	0.01497044	0.85299473
Q3ULJ0	Gpd1l Kiaa00	-0.2034523	0.01486315	0.96398649	-0.1286503	0.00447245	0.99638741	-0.1567464	0.04239392	0.69497647
Q64521	Gpd2 Gdm1	-0.1495994	0.01625138	0.54762287	-0.11055	0.01625601	0.39110852	-0.1305577	0.00876727	0.76007384
P06745	Gpi Gpi1	-0.1690467	0.00275906	0.9941737	-0.0935252	0.00243648	0.98463017	-0.1541007	0.0143287	0.84018994
Q8QZR5	Gpt Gpt1	-0.6795785	0.09416915	0.80024253	-0.4068376	0.06892109	0.71337785	-0.5987039	0.110304	0.69383389
P11352	Gpx1	-0.1458484	0.00192731	0.99289142	-0.0859873	0.0024467	0.96560112	-0.1355165	0.01019389	0.81543654
Q91Z53	Grhpr Glxr	-0.1004312	0.00557216	0.96152201	-0.0482791	0.00797912	0.72337858	-0.0951998	0.0136442	0.78924458
P28798	Grn	-0.4637782	0.05780542	0.54380333	-0.219885	0.03651583	0.38880607	-0.5060278	0.06896088	0.4992801
P30115	Gsta3 Gstyc	-0.1507668	0.00822683	0.94115191	-0.081048	0.00424465	0.9579597	-0.1637228	0.02147278	0.76357966
Q9DCM2	Gstk1	-0.2787378	0.0405494	0.54155931	-0.1741777	0.03193583	0.4146014	-0.3254593	0.06296897	0.40042636
P10649	Gstm1	-0.1462361	0.00371815	0.97417934	-0.0933522	0.00144393	0.99052091	-0.1277792	0.00825404	0.86004236
P15626	Gstm2	-0.1302342	0.00843132	0.94832938	-0.084476	0.00560415	0.94196187	-0.110005	0.01658966	0.7718073
P19639	Gstm3	NA	NA	NA	NA	NA	NA	NA	NA	NA
P19157	Gstp1 Gstpib	-0.1333162	0.00577061	0.95184861	-0.092837	0.00635106	0.88049762	-0.1093968	0.00998691	0.81631456
Q64471	Gstt1	-0.1485842	0.00428203	0.99258069	-0.0871374	0.00361321	0.98476122	-0.122389	0.01862407	0.84370487
Q9WVL0	Gstz1 Maai	-0.1235628	0.00302366	0.96198102	-0.0801083	0.00225635	0.94596614	-0.1160885	0.00670367	0.82186099
P58710	Gulo	-0.4677218	0.03978977	0.75847515	-0.311713	0.03447221	0.62053877	-0.3804015	0.05370497	0.5626396
Q80SU7	Gvin1	-0.367318	0.20378334	0.21306209	-0.0922147	0.01609504	0.80404563	-0.0779636	0.03798277	0.29642799
Q8VCB3	Gys2	-0.1569252	0.00421428	0.92835916	-0.0910936	0.01761776	0.23301278	-0.1896395	0.01575027	0.57534824
P14434	H2-Aa	-0.2192809	0.03136978	0.87469309	-0.1815618	0.02231754	0.95663769	-0.2229789	0.02517101	0.94010118
P01899	H2-D1	-0.9806527	0.12560323	0.82422394	-0.3308955	0.05651849	0.71000585	-1.0073898	0.13442264	0.81203821
P01901	H2-K1 H2-K	-0.6998475	0.07905664	0.89698561	-0.2274208	0.03421638	0.84667453	-0.8352601	0.13578924	0.80784283
Q9QZQ8	H2afy	-0.0784226	0.00167644	0.98293139	-0.0396443	0.00189066	0.91091362	-0.0818858	0.0053599	0.85682899
Q8CFX1	H6pd	-0.6769512	0.07399521	0.86555879	-0.5247263	0.08422949	0.74908045	-0.611876	0.12196094	0.69588156
Q78JT3	Haao	-0.1938395	0.00705628	0.94966194	-0.1236925	0.00637749	0.89741681	-0.1798119	0.01596979	0.77408224
Q9QXE0	Hacl1 Hpcl Pl	-0.5339794	0.05961523	0.58040829	-0.400535	0.05741497	0.4520119	-0.5532532	0.06284508	0.56776761
Q61425	Hadh Hadhsc	-0.1307243	0.00199681	0.97608665	-0.0748034	0.00136799	0.96669958	-0.1212307	0.00567586	0.81727199
Q8BMS1	Hadha	-0.1241425	0.00150002	0.96546553	-0.0722964	0.00132368	0.92181976	-0.1191346	0.00776069	0.50498667
Q99JY0	Hadhb	-0.113367	0.0037898	0.94607906	-0.0759528	0.00291394	0.92510914	-0.1140966	0.00708062	0.84124872
Q99KB8	Hagh Glo2	-0.2494381	0.15636417	0.16370668	-0.5427917	0.13212673	0.54658223	-0.2205358	0.10642692	0.26352953
P35492	Hal Hsd Huth	-0.4627713	0.07780045	0.56717505	-0.192027	0.0456773	0.42409507	-0.5838332	0.09649341	0.56662069
Q9WU19	Hao1 Gox1 H	-0.1983306	0.02782926	0.86392189	-0.1494288	0.01759012	0.91157789	-0.4301991	0.12455014	0.54400941
P01942	Hba Hba-a1	-0.0196097	0.00098712	0.94492919	-0.0136598	0.00096272	0.90554127	-0.0171788	0.0025914	0.66639299
P02088	Hbb-b1	-0.019357	0.00105659	0.86809085	-0.0161484	0.00385301	0.2525012	-0.018286	0.00166332	0.7200062
Q8VDJ3	Hdlbp	-0.7442067	0.04905638	0.7517493	-0.7355079	0.04284055	0.80368495	-0.7532759	0.05528256	0.71778252

Q9R257	Hebp1 Hbp	-0.7362686	0.16690611	0.59949894	-0.6367939	0.09680444	0.75555256	-0.9688613	0.08999916	0.89913889
O09173	Hgd Aku Hgo	-0.211213	0.01513756	0.72729006	-0.1264852	0.00619017	0.84429257	-0.2251539	0.02345694	0.55125616
Q99L13	Hibadh	-0.4201811	0.14903826	0.41947476	-0.3688462	0.12215202	0.34910247	-0.235906	0.08083986	0.39579359
Q8QZS1	Hibch	-0.1626216	0.00353406	0.99436469	-0.0956061	0.00390626	0.98036107	-0.1448811	0.01681667	0.87092818
P70349	Hint1 Hint Pk	-0.1591713	0.00273766	0.99616905	-0.0982139	0.00224017	0.99174461	-0.1473267	0.01375392	0.85793194
P43275	Hist1h1a H1a	-0.2449329	0.08601489	0.38413791	-0.7804136	0.14168302	0.71657933	-0.3765746	0.10842206	0.50131568
P43276	Hist1h1b H1f	-0.0277714	0.00756784	0.50881095	-0.044047	0.01756308	0.32606593	-0.0397927	0.01544984	0.3560075
P43274	Hist1h1e H1f	-0.040584	0.00117087	0.9780205	-0.0241612	0.00120993	0.93220539	-0.0406537	0.00349196	0.83388565
P68433	Hist1h3a H3a	-0.0077835	0.00185432	0.61564066	-0.0047364	0.00086144	0.75143709	-0.0090129	0.0017959	0.73673557
P84228	Hist1h3b H3-	-0.0077532	0.00104386	0.84654756	-0.0042144	0.0011171	0.64017151	-0.0099969	0.00244508	0.67633184
P62806	Hist1h4a; His	-0.0122652	0.00159776	0.5218202	-0.0059659	0.00052715	0.69202682	-0.0118344	0.00091334	0.75324295
P30681	Hmgb2 Hmg2	-0.490476	0.13172897	0.66448584	-0.4558606	0.17126057	0.50302288	-0.7724899	0.12426638	0.82848688
P38060	Hmgcl	-0.1516987	0.0042687	0.97604158	-0.0837964	0.00555129	0.87015836	-0.1357233	0.00955749	0.86304928
P54869	Hmgcs2	-0.2035991	0.00253886	0.98003636	-0.1244864	0.00907724	0.6124753	-0.2170378	0.01626434	0.58562569
P14901	Hmox1	-0.4794293	0.1155107	0.45064724	-0.4429608	0.07635749	0.56414803	-0.5611484	0.12059588	0.48490123
O70252	Hmox2	-0.155317	0.03285447	0.73639593	-0.1212134	0.00565085	0.9913816	-0.1161772	0.02549195	0.80597564
O88569	Hnrnpa2b1 H	-0.235051	0.01739655	0.98916323	-0.1859004	0.03544553	0.87304267	-0.378002	0.0601838	0.88750992
Q99020	Hnrnpab Cbf	-0.1657113	0.0996071	0.23519661	-0.1543846	0.09863437	0.21396804	-0.3627366	0.15114364	0.32431445
Q60668	Hnrnpd Auf1	-0.1394954	0.00498982	0.98363826	-0.0853528	0.00418204	0.96748295	-0.1290611	0.01946143	0.77184426
Q9Z2X1	Hnrnpf Hnrp	-0.038803	0.0092466	0.5753066	-0.048255	0.00780206	0.73207293	-0.0451753	0.0092356	0.64794557
P61979	Hnrnpk Hnrp	-0.5797459	0.05219746	0.82043176	-0.2081656	0.01003571	0.93889814	-0.5876316	0.07708742	0.69087726
Q8R081	Hnrnpl Hnrpl	-0.5893228	0.17730253	0.8467177	-0.2947474	0.41590004	0.33433298	-0.4261045	0.20759091	0.6781064
Q8VEK3	Hnrnpu Hnrp	-0.5235613	0.05352	0.75532371	-0.2382493	0.04768756	0.44603769	-0.4233348	0.05434064	0.69209779
Q9DCU9	Hoga1 Dhdps	-0.0988113	0.00466464	0.97184454	-0.0536693	0.0028186	0.96282165	-0.0929427	0.01165042	0.8303815
Q61646	Hp	NA	NA	NA	NA	NA	NA	NA	NA	NA
P49429	Hpd	-0.6736302	0.03625138	0.7878156	-0.4083412	0.02898354	0.66276252	-0.6866383	0.0470628	0.69822526
Q91X72	Hpx Hpxn	-0.4361031	0.09837413	0.58398266	-0.3154743	0.10077248	0.47116417	-0.6268061	0.15490008	0.55743647
Q9ESB3	Hrg	-0.2751975	0.20411481	0.23251811	-0.1334895	0.11617856	0.39762792	-0.8054725	0.52277306	0.5427494
P52760	Hrsp12 Hrp1	-0.1742578	0.00381888	0.98719866	-0.0993235	0.00224119	0.98743102	-0.1414437	0.01244218	0.83251006
P50172	Hsd11b1 Hsd	-0.5356856	0.03940789	0.77384982	-0.2887441	0.03016038	0.651628	-0.4564815	0.04841985	0.6446161
O08756	Hsd17b10 Er	-0.118829	0.00370218	0.94670191	-0.0721499	0.00357009	0.87377679	-0.1020348	0.00782828	0.76911456
Q9EQ06	Hsd17b11 Dh	-0.3381382	0.01873756	0.96161325	-0.2706241	0.00940221	0.99043591	-0.3285755	0.02863761	0.93600807
O70503	Hsd17b12 Ki	-0.3295783	0.03767259	0.58186373	-0.1460184	0.00702007	0.87999491	-0.1873158	0.01704262	0.6990786
Q8VCR2	Hsd17b13 Sc	-0.6512905	0.04984924	0.69474915	-0.4204465	0.03585151	0.63223945	-0.5921285	0.05321805	0.62273286
P51658	Hsd17b2 Edh	-0.4846239	0.05138472	0.87248539	-0.2126921	0.00751832	0.98401602	-0.2696168	0.03133819	0.85060819
P51660	Hsd17b4 Edh	-0.74403	0.02337929	0.8296181	-0.5502418	0.01852973	0.8032424	-0.6966089	0.02766671	0.75836211
Q9R092	Hsd17b6 Gm	-0.2850036	0.0596323	0.45829012	-0.256278	0.04821216	0.50227471	-0.1919117	0.01636719	0.84096385
P50171	Hsd17b8 H2-	-0.1224385	0.0022229	0.99573332	-0.0640852	0.0022128	0.98473722	-0.1171307	0.01715651	0.79525855
P11499	Hsp90ab1 Hs	-0.5506831	0.03383382	0.73809723	-0.5907616	0.03776544	0.74672019	-0.5745878	0.05156108	0.61421449
P08113	Hsp90b1 Grp	-0.4213715	0.02512059	0.69581961	-0.2182597	0.01555427	0.61168199	-0.2877881	0.02736356	0.48384066
P20029	Hspa5 Grp78	-0.4930403	0.02040693	0.79773862	-0.3600358	0.02021386	0.65921612	-0.3539704	0.02805761	0.52500254
P63017	Hspa8 Hsc70	-0.7166474	0.05063933	0.79387893	-0.6946032	0.05770843	0.73215485	-0.6009045	0.06234283	0.64114355
P38647	Hspa9 Grp75	-0.5091775	0.04097418	0.72018209	-0.2455658	0.02926948	0.57046469	-0.4319218	0.05222909	0.54541422
P63038	Hspd1 Hsp60	-0.1208032	0.00314662	0.93408975	-0.0743575	0.00241246	0.90047488	-0.1153316	0.00539425	0.82346332
Q64433	Hspe1	-0.1234795	0.00102063	0.99815877	-0.0664067	0.00157151	0.98401874	-0.1107033	0.00865611	0.85831197
Q9JKR6	Hyou1 Grp17	-0.4351123	0.04246056	0.57067585	-0.2955955	0.03463325	0.48940713	-0.3527779	0.04713882	0.41794254
Q9DB29	Iah1	-0.2027473	0.00778159	0.98120981	-0.1196825	0.00348238	0.98828611	-0.1829495	0.02116106	0.86166494
P03975	Iap	-0.0510988	0.01966705	0.49093164	-0.0355671	0.0053769	0.79910663	-0.084721	0.02502217	0.74133308
O88844	Idh1	-0.1429055	0.00525811	0.91456692	-0.0856537	0.00334757	0.89968221	-0.1358252	0.0080511	0.81876254
P54071	Idh2	-0.1258454	0.00570651	0.91703316	-0.0685598	0.00319736	0.89839507	-0.1177697	0.03086253	0.25297211
Q9ESY9	Ifi30 Gilt Ip30	-0.115103	0.03405167	0.69560562	-0.1224066	0.03410758	0.72035436	-0.1690287	0.0729773	0.51759326
P01867	Igh-3	-0.0972044	0.00320602	0.98605539	-0.0612697	0.00476436	0.95385848	-0.1087182	0.01808667	0.83770596
Q9QZ85	Iigp1 Irga6	-0.6470162	0.0401897	0.75303592	-0.3206905	0.03117729	0.56038691	-0.6111872	0.05215377	0.65293244
Q8BU33	Ilvbl	-0.3483835	0.02914677	0.94073792	-0.2527689	0.01071394	0.99641967	-0.3423153	0.04230231	0.94243144
Q8CAQ8	Immt	-0.1557212	0.01819294	0.4811638	-0.0836811	0.01441003	0.29395095	-0.1086544	0.01548988	0.38987593
P40936	Inmt Temt	-0.1432755	0.00193041	0.99620231	-0.0899496	0.00171347	0.99136621	-0.1274861	0.01155351	0.83534346
Q9JKF1	Iqgap1	-0.0746409	0.02325408	0.46195106	-0.0493184	0.02758776	0.38993498	-0.2053541	0.05544235	0.63165939
Q3UQ44	Iqgap2	-0.4103371	0.02790768	0.75016419	-0.2417176	0.01776762	0.73130971	-0.436433	0.03857339	0.66669203
Q60766	Irgm1 Ifi1 Iig	-0.2660028	0.0167193	0.91011263	-0.1511684	0.00911546	0.91362714	-0.262164	0.04804802	0.54355507
P85094	Isoc2a Isoc2	-0.1251634	0.00622258	0.93311643	-0.068143	0.00253031	0.96539163	-0.1297447	0.0099714	0.87584308
P05555	Itgam	-0.2009482	0.03991337	0.66099295	-0.2941803	0.08908353	0.52164939	-0.276559	0.07531792	0.5091148
P09055	Itgb1	-0.3667745	0.08035667	0.6345155	-0.1451955	0.00770082	0.96211037	-0.5447771	0.11218757	0.64461687
P11835	Itgb2	-0.8007298	0.12674983	0.78393067	-0.6160406	0.13168762	0.64585164	-0.6785419	0.12532075	0.67679515
P97328	Khk	-0.2114795	0.03185269	0.62014774	-0.1033677	0.00298247	0.97801668	-0.1478537	0.01238141	0.84579021
Q91WN4	Kmo	-0.1904753	0.00292292	0.98721414	-0.1074702	0.00565846	0.8594325	-0.2262526	0.03960255	0.37672639
O08677	Kng1 Kng	-0.8625164	0.16128695	0.87729325	-0.8508145	0.05253611	0.97763474	-1.1207912	0.11117754	0.9531082

P70168	Kpnb1 Impnb	-0.2492402	0.01158883	0.95459687	-0.1815212	0.00421417	0.99092058	-0.2426005	0.02222472	0.83820428
P05784	Krt18 Kerd Kr	-0.0845157	0.00105316	0.98021304	-0.0561772	0.00092624	0.96485252	-0.077564	0.00329808	0.81686421
P19001	Krt19 Krt1-19	-0.0807698	0.02250768	0.68216387	-0.0910609	0.0394316	0.51611519	-0.1163425	0.01903428	0.86162271
P11679	Krt8 Krt2-8	-0.0979025	0.00193095	0.96835761	-0.0624225	0.00221624	0.90322441	-0.0899483	0.0053426	0.77562072
Q9EP89	Lactb Lact1	-0.1331406	0.00796942	0.93626404	-0.0620048	0.00315891	0.94831147	-0.098315	0.01631218	0.68120505
Q9CPY7	Lap3 Lapep	-0.1152497	0.0238942	0.49221992	-0.0880064	0.04031607	0.16009008	-0.1433263	0.041855	0.32822386
Q3U9G9	Lbr	-0.5592618	0.07742496	0.80053918	-0.4603701	0.10142598	0.61312173	-0.436005	0.11930561	0.52673052
P11672	Lcn2	-0.0238247	0.01315223	0.20154125	-0.0224824	0.00856201	0.36491143	-0.0347975	0.01638922	0.25748062
Q61233	Lcp1 Pls2	-0.3402412	0.04731323	0.42141913	-0.2013855	0.03165091	0.36313758	-0.3500408	0.04660077	0.44630036
P06151	Ldha Ldh-1 Lc	-0.239364	0.02598618	0.53752467	-0.176103	0.02225904	0.49056479	-0.1896319	0.01741305	0.59717367
Q7TNG8	Ldhd	-0.3525366	0.05013127	0.80472821	-0.1560117	0.0065795	0.97910316	-0.2483642	0.03026619	0.84874906
Q9Z2I0	Letm1	-0.332992	0.04564203	0.58992648	-0.2031041	0.03637222	0.44429841	-0.2842613	0.05872362	0.40101337
P16045	Lgals1 Gbp	-0.354288	0.08472225	0.57359026	-0.2211106	0.14571216	0.20372634	-0.2750776	0.10071785	0.48251192
O08573	Lgals9	-0.8955819	0.07747583	0.83190351	-0.5739175	0.05959981	0.7617632	-0.8602345	0.08732124	0.78234523
O89017	Lgmn Prsc1	-0.2667341	0.02752263	0.8951623	-0.1291931	0.00609982	0.98247861	-0.169251	0.01362467	0.94489207
Q9D7I5	Lhpp	-0.3082389	0.05824918	0.68294538	-0.2177503	0.09087889	0.36471843	-0.2034622	0.11906048	0.18343415
Q9D0F3	Lman1 Ergic5	-0.532997	0.04617863	0.61613059	-0.4051601	0.03779342	0.56356863	-0.4378105	0.05157476	0.46472539
Q9DBH5	Lman2	-0.6893129	0.05815368	0.83880666	-0.501036	0.05435383	0.75886902	-0.6408078	0.07908453	0.73230932
P48678	Lmna Lmn1	-0.0863435	0.03070074	0.16861584	-0.0837049	0.02450526	0.21740551	-0.0635403	0.00826844	0.62126945
Q9DBN5	Lonp2	-0.4319227	0.14678181	0.46406551	-0.2215164	0.03404214	0.84108897	-0.4153895	0.1202162	0.52047718
Q91YX5	Lpgat1 Fam3	-0.214027	0.05208286	0.43425545	-0.3407936	0.11624037	0.30058815	-0.271661	0.0845989	0.37755343
Q91ZX7	Lrp1 A2mr	-0.3870314	0.07422813	0.4754018	-0.246552	0.05262813	0.4224916	-0.3529037	0.10163568	0.30098677
Q922Q8	Lrrc59	-0.0950291	0.00861008	0.81856598	-0.061261	0.00867413	0.66612692	-0.0782971	0.01006604	0.69943082
P08071	Ltf	-0.2764931	0.069786	0.29233275	-0.2430249	0.0847608	0.19020383	-0.1830333	0.06495361	0.18492058
P25911	Lyn	-0.7729459	0.13055567	0.59357518	-0.5278801	0.08035789	0.64260854	-0.6072355	0.09528641	0.6486286
P08905	Lyz2 Lyz Lyzs	-0.4406098	0.10564759	0.63495106	-0.4536963	0.11454192	0.6107318	-0.5107172	0.15160246	0.48605417
P24668	M6pr 46mpr	-0.6839526	0.07711886	0.91827757	-0.0669737	0.03901803	0.59565794	-0.5201887	0.22267868	0.438073
Q922B1	Macrod1 Lrp	-0.3018183	0.05209136	0.72085423	-0.1159519	0.00502257	0.97440452	-0.2237792	0.07762611	0.38997032
P27046	Man2a1 Mar	-0.6828739	0.05984789	0.68094854	-0.4698119	0.05085635	0.58316537	-0.6812148	0.0650224	0.65039002
Q9CXI5	Manf Armet	-0.6199091	0.09242967	0.63370703	-0.4870109	0.07534779	0.61638847	-0.5085844	0.08750547	0.56506962
Q8BW75	Maob	-0.212028	0.01144206	0.74424683	-0.1074619	0.00321609	0.89284138	-0.2102393	0.02167189	0.45004932
Q9CW42	Marc1 Mosc1	-0.2428121	0.0094853	0.85070698	-0.1899919	0.01345937	0.65490072	-0.2468607	0.01625999	0.67694182
Q922Q1	Marc2 Mg87	-0.451221	0.02667493	0.8057081	-0.1989739	0.00472217	0.96311265	-0.3918803	0.03841432	0.60131469
Q91X83	Mat1a	-0.4548515	0.07787291	0.63042796	-0.2449997	0.02721689	0.70442888	-0.2966679	0.05620956	0.58208073
P04247	Mb	-0.2603841	0.07250349	0.76328093	-0.1967938	0.02426007	0.9563966	-0.3025246	0.0499462	0.85944319
P41317	Mbl2	-0.5670451	0.08069893	0.5586945	-0.2086842	0.04245498	0.37079295	-0.6137629	0.08935461	0.54118455
Q99MR8	Mccc1 Mcca	-0.1497274	0.01242854	0.86836723	-0.0916776	0.01023269	0.80860026	-0.0994626	0.01143015	0.81665405
Q3ULD5	Mccc2	-0.4133709	0.0705008	0.52584077	-0.2271111	0.05327316	0.3622245	-0.2764265	0.05832056	0.39093883
P14152	Mdh1 Mor2	-0.1707354	0.0099603	0.89356341	-0.0948309	0.00376882	0.94619825	-0.1434411	0.01209973	0.80061364
P08249	Mdh2 Mor1	-0.1333812	0.0048011	0.90821434	-0.1012531	0.01754476	0.28152378	-0.1119091	0.00609818	0.80804679
P06801	Me1 Mod-1 I	-0.3023394	0.02454227	0.47460753	-0.1668578	0.01867294	0.32880016	-0.2815754	0.02220131	0.47610519
Q9DCS3	Mecr Nrbf1	-0.130264	0.01601624	0.94297909	-0.0707639	0.03012337	0.73398754	-0.1574933	0.02922098	0.85315378
Q9DD20	Mettl7b	-0.5641812	0.03729195	0.77882103	-0.4111727	0.03573134	0.65743043	-0.5697832	0.04696013	0.69371109
Q91VS7	Mgst1	-0.2850941	0.05489382	0.64262754	-0.14806	0.01124494	0.92036738	-0.2737854	0.08069013	0.41845191
Q80UM7	Mogs Gcs1	-0.1049453	0.00280807	0.99077835	-0.0661504	0.00361453	0.96541137	-0.0784728	0.00941378	0.84240132
P63030	Mpc1 Brp44l	-0.2157423	0.00823144	0.98848823	-0.1431072	0.01287269	0.92514427	-0.2218881	0.02192786	0.91102747
P11247	Mpo	-0.2735702	0.04793366	0.89062938	NA	NA	NA	-0.3709414	0.03070203	0.97332877
Q99J99	Mpst	-0.2736075	0.10318428	0.43859437	-0.1276277	0.00649773	0.96740248	-0.2408698	0.03309278	0.80296563
P19258	Mpv17	-0.1981776	0.00558061	0.99213274	-0.1129829	0.00501981	0.9902264	-0.1702713	0.02381347	0.86469457
Q61830	Mrc1	-0.4397155	0.08157586	0.50924512	-0.3835846	0.08616013	0.52406481	-0.42114	0.10669309	0.45055688
Q99N96	Mrpl1	-0.1414412	0.00516383	0.98296764	-0.083002	0.00393181	0.973779	-0.1090929	0.01288894	0.87751175
Q9EQI8	Mrpl46	-0.1673044	0.02646884	0.83316865	-0.1033888	0.01990122	0.7499239	-0.1199215	0.01982388	0.82060585
Q61733	Mrps31 Imog	-0.2023155	0.01194427	0.97287266	-0.1363261	0.00469442	0.98828113	-0.1765615	0.03439003	0.79016082
P26041	Msn	-0.2679719	0.05069684	0.37786868	-0.224035	0.05834197	0.24680957	-0.2805406	0.04510198	0.45151211
P30204	Msr1 Scvr	NA	NA	NA	NA	NA	NA	NA	NA	NA
Q791V5	Mtch2	-0.1588992	0.00206891	0.99561162	-0.088727	0.00342476	0.95995431	-0.1406572	0.01159849	0.84488909
P00397	Mtco1 COI m	-0.1640096	0.00166134	0.99866788	-0.094726	0.00285155	0.98747218	-0.1399446	0.01600715	0.85464071
P00405	Mtco2 COII m	-0.1487397	0.00174926	0.99449805	-0.0837018	0.0013767	0.99062036	-0.1334486	0.00865078	0.85918913
Q922D8	Mthfd1	-0.1710021	0.00394165	0.91050357	-0.1107313	0.00175344	0.9529273	-0.1778848	0.01156296	0.56528814
P03911	Mtnd4 mt-Nd	-0.1570062	0.00270661	0.99762821	-0.0810938	0.00738462	0.96018858	-0.1234438	0.02961575	0.68471333
O08601	Mttp Mtp	-0.3058978	0.02939089	0.56032516	-0.2896513	0.02884194	0.52296023	-0.2689691	0.02931206	0.48614316
O88441	Mtx2 MNCb-	-0.2709192	0.02238967	0.7982711	-0.138207	0.00407838	0.97124438	-0.2282424	0.03241548	0.58618012
P16332	Mut	-0.3794843	0.05343695	0.72635018	-0.3252749	0.06505409	0.58140201	-0.3381588	0.0287896	0.87338996
Q9EQK5	Mvp	-0.3058055	0.04109809	0.62655541	-0.1834976	0.0302801	0.58548353	-0.2407765	0.02833937	0.6672372
O08638	Myh11	NA	NA	NA	NA	NA	NA	-0.110295	0.1877478	0.2565682
Q8VDD5	Myh9	-0.4032463	0.01471793	0.68574925	-0.2410983	0.01168407	0.55747243	-0.3981155	0.01850922	0.57212055

Q3THE2	Myl12b Mrlc	-0.7451396	0.05575079	0.85621011	-0.5558165	0.05865367	0.7433758	-0.7714271	0.07298089	0.78280733
Q642K0	Myl6 mCG_1	-0.1324493	0.03121953	0.78259866	-0.1205019	0.0330821	0.65462582	-0.2788453	0.1216851	0.46671966
Q60605	Myl6 Myln	-0.5238339	0.04406546	0.77511547	-0.2601673	0.01997205	0.7978292	-0.5965343	0.0651866	0.68786988
P46735	Myo1b	-0.2833364	0.08503943	0.52609001	-0.1707201	0.00602999	0.98525003	-0.308882	0.08809642	0.52776132
Q9QWR8	Naga	-0.0383349	0.04251565	0.21321791	NA	NA	NA	NA	NA	NA
Q99KQ4	Nampt Pbef1	-0.6646962	0.10139368	0.81123481	-0.514785	0.11232702	0.6176819	-0.2441637	0.03929407	0.76289623
Q9DB05	Napa Snapa	-0.8537833	0.11991689	0.80858631	-0.5435783	0.10062487	0.67579012	-0.8921193	0.14000125	0.77188584
Q8BLF1	Nceh1 Aadac	-0.2449883	0.01879528	0.89941769	-0.2048836	0.00847979	0.99150777	-0.2303755	0.01842822	0.92869063
P09405	Ncl Nuc	-0.4002843	0.06048309	0.58555877	-0.3276126	0.05280998	0.54600142	-0.2148349	0.06650788	0.26459961
P57716	Ncstn	-0.1247667	0.03362031	0.57933514	-0.2291704	0.10491199	0.32302664	-0.1288955	0.04156972	0.57867823
Q9QYG0	Ndrg2 Kiaa12	-0.9491684	0.04582735	0.88819412	-0.5205455	0.03622302	0.78667746	-0.915677	0.06115236	0.80301681
Q99LC3	Ndufa10	-0.1982776	0.00427261	0.9693911	-0.1114569	0.00230223	0.97301544	-0.184727	0.01009309	0.83540118
Q9D8B4	Ndufa11	-0.4138695	0.06118475	0.8061852	-0.3830098	0.14617614	0.53363334	-0.3904865	0.04967832	0.89823262
Q7TMF3	Ndufa12	-0.2179726	0.00527037	0.99245718	-0.111358	0.00362163	0.98540814	-0.1912069	0.0232083	0.83926147
Q9ERS2	Ndufa13 Grir	-0.1706974	0.00699396	0.95663852	-0.108149	0.01187982	0.74746419	-0.1703987	0.02970564	0.55860746
Q9CQ75	Ndufa2	-0.1452123	0.00209046	0.99731309	-0.0824863	0.00188889	0.99271214	-0.1288323	0.01433511	0.86136231
Q9CQ91	Ndufa3	-0.1562046	0.00173349	0.99840154	-0.088054	0.00216313	0.99162198	-0.1448644	0.016297	0.85871824
Q62425	Ndufa4	-0.2930907	0.01117544	0.98145032	-0.1960793	0.00885587	0.97416693	-0.4128211	0.09052038	0.61536703
Q9CPP6	Ndufa5	-0.180179	0.00685491	0.97875007	-0.1034028	0.00418962	0.96667386	-0.1490522	0.02500228	0.78041304
Q9DCJ5	Ndufa8	-0.1451761	0.00351884	0.99242037	-0.0772819	0.00204551	0.99028735	-0.1250681	0.01414405	0.85743888
Q9DC69	Ndufa9	-0.2640292	0.02116272	0.69286132	-0.1126134	0.00441907	0.90269791	-0.243151	0.03292339	0.45247977
Q9DCS9	Ndufb10	-0.1642321	0.00472381	0.971859	-0.0948238	0.00368066	0.94719706	-0.1515434	0.01156094	0.8348117
O09111	Ndufb11 Np1	-0.4029859	0.10455972	0.55314341	-0.4071941	0.10841964	0.52039173	-0.6285486	0.14195511	0.64058629
Q9CQC7	Ndufb4	-0.1854337	0.00485199	0.98649219	-0.1117628	0.00316105	0.9811626	-0.1706286	0.01504012	0.87136663
Q9CR61	Ndufb7	-0.1986422	0.03930689	0.51553421	-0.0703461	0.00354108	0.95176623	-0.142128	0.01448293	0.84252572
Q9D6J5	Ndufb8	-0.1371716	0.00449508	0.98623203	-0.0833416	0.00283071	0.98410591	-0.1622639	0.08163845	0.23306196
Q9CQJ8	Ndufb9	-0.143794	0.00282819	0.9949962	-0.0824704	0.00239897	0.98829241	-0.1266362	0.01458781	0.8528731
Q9CQ54	Ndufc2	-0.143823	0.00212245	0.99717684	-0.0796737	0.00205806	0.99074504	-0.1285126	0.01287716	0.8845453
Q91VD9	Ndufs1	-0.1549313	0.00370569	0.94537589	-0.1034285	0.0089634	0.54313334	-0.1448654	0.01305545	0.55430469
Q91WD5	Ndufs2	-0.1957036	0.00700908	0.89137971	-0.1183907	0.00943881	0.59295388	-0.1726593	0.02067602	0.42075938
Q9DCT2	Ndufs3	-0.2020897	0.01798605	0.6062345	-0.1273225	0.01580532	0.42167937	-0.1404301	0.00778858	0.80053695
Q9CXZ1	Ndufs4	-0.3181732	0.01297733	0.98524865	-0.158324	0.00853839	0.97173768	-0.2565678	0.02296381	0.93275017
Q99LY9	Ndufs5	-0.159592	0.00366638	0.98595016	-0.087084	0.00347715	0.95726718	-0.1471811	0.01347631	0.82672389
P52503	Ndufs6 Ip13	-0.3266575	0.02987955	0.8157236	-0.1373568	0.00219049	0.99267866	-0.311928	0.0414025	0.67765704
Q9DC70	Ndufs7	-0.1869133	0.00987344	0.93723521	-0.1241308	0.0063244	0.94365929	-0.1532864	0.0158562	0.8094518
Q8K3J1	Ndufs8	-0.1116863	0.00234271	0.99431272	-0.0587687	0.00291697	0.96665931	-0.0875754	0.01100423	0.82969856
Q91YT0	Ndufv1	-0.2020655	0.01059398	0.80167593	-0.1326682	0.01071711	0.60751974	-0.1967559	0.01855354	0.57243439
Q9D6J6	Ndufv2	-0.1654349	0.00350784	0.98800639	-0.080462	0.00504786	0.90073682	-0.1328179	0.01290057	0.80302641
O70131	Ninj1	-0.288523	0.02687987	0.9275446	-0.2133257	0.03197361	0.86411638	-0.2736909	0.03506626	0.89693369
O55125	Nipsnap1	-0.1252293	0.00337601	0.9496188	-0.0711578	0.00331438	0.85845594	-0.1320722	0.01867794	0.43477962
Q9CQE1	Nipsnap3b N	-0.1574232	0.00343345	0.99385406	-0.095543	0.00203356	0.9936977	-0.1340196	0.01630673	0.83860262
Q6GQT9	Nomo1	-0.1638203	0.01808474	0.83682808	-0.1218609	0.01678663	0.76709964	-0.1038522	0.00949412	0.87559698
Q99K48	Nono	NA	NA	NA	-0.1311117	0.04880835	0.78298536	-0.092406	0.03318584	0.79494423
Q61937	Npm1	-0.4328883	0.07191231	0.51592059	-0.3051254	0.05802715	0.52516572	-0.3733472	0.07055746	0.46665636
Q9R1J0	Nsdhl	-0.5893443	0.13111074	0.66893037	-0.4218089	0.08647536	0.82634585	-0.5977919	0.08449245	0.80662919
P11928	Oas1a Oias1	-0.3820478	0.14472722	0.3489723	-0.3971981	0.1610116	0.37832406	-0.4799743	0.11697846	0.62735827
P29758	Oat	-0.6356118	0.08511527	0.61439275	-0.3289581	0.05158614	0.51044675	-0.6669709	0.08124767	0.65816795
Q9CZ30	Ola1 Gtpbp9	-0.3595231	0.0800358	0.69155183	-0.286546	0.08138547	0.52984188	-0.354885	0.10966608	0.48770548
P58281	Opa1	-0.3059766	0.12510916	0.35223006	-0.1181021	0.00384943	0.98534471	-0.1269694	0.02468046	0.7064022
Q78XF5	Ostc	-0.081972	0.00146674	0.99585512	-0.0460749	0.0018373	0.97822306	-0.0577571	0.00729676	0.82816572
P11725	Otc	-0.1070383	0.00157804	0.98227966	-0.0529508	0.0019893	0.89063593	-0.1124873	0.01391986	0.44942882
Q9D0K2	Oxct1 Oxct S	-0.0160141	0.01546699	0.07617932	-0.1190188	0.10107895	0.07974403	-0.0445109	0.10759069	0.01299452
P09103	P4hb Pdia1	-0.3805659	0.01872295	0.68612637	-0.2386181	0.01456253	0.57679302	-0.236858	0.0162777	0.53639631
P50580	Pa2g4 Ebp1 F	-0.1669789	0.00275444	0.99458728	-0.1011148	0.00181961	0.99356495	-0.1521271	0.01643273	0.81853354
P29341	Pabpc1 Pabp	-0.2724635	0.02019192	0.95289924	-0.3623488	0.13669751	0.63723401	-0.1800668	0.03292472	0.81035181
P16331	Pah	-0.6747501	0.09453467	0.65360298	-0.4081671	0.06271337	0.6288597	-0.7711651	0.09560431	0.72241916
O88428	Papss2 Atpsk	-0.5156983	0.04876629	0.73172517	-0.1919238	0.01369618	0.83785816	-0.2685361	0.03854155	0.56748058
Q6TCG2	Paqr9	-0.7048533	0.12334618	0.73127144	-0.5142251	0.10658381	0.62443171	-0.6751736	0.14126651	0.67496937
Q9DCG6	Pbld1 Mawb	-0.1103351	0.0047815	0.97796038	-0.0761067	0.00358893	0.97190356	-0.1056185	0.01209487	0.85435236
Q05920	Pc Pcx	-0.1984546	0.01736343	0.4165147	-0.1185948	0.01628466	0.21732966	-0.2112647	0.02210421	0.32013096
P61458	Pcbd1 Dcoh I	-0.1333695	0.00413731	0.98764435	-0.0908902	0.00618412	0.93913373	-0.2485236	0.07838253	0.43608263
Q99MN9	Pccb	-0.1042138	0.00384651	0.98259791	-0.0475043	0.00398499	0.91618601	-0.0917312	0.02195251	0.57322301
Q9Z2V4	Pck1 Pepck	-0.0443262	0.01489237	0.59620965	-0.0289815	0.02113846	0.38521239	-0.0799144	0.05311826	0.24433816
Q9CQF9	Pcyox1 Kiaa0	-0.5354209	0.04917821	0.60620787	-0.3886933	0.03932438	0.56571843	-0.5068926	0.05788831	0.50887463
Q9WU78	Pdcd6ip Aip1	-0.8719936	0.11737139	0.8214161	-0.7777831	0.09059554	0.85006821	-0.6221991	0.18379377	0.58890735
P35486	Pdha1 Pdha-	-0.1021174	0.01740647	0.72583893	-0.0721039	0.0072857	0.86719001	-0.1276935	0.01212331	0.85378077

Q9D051	Pdhb	-0.2370287	0.06674653	0.27055607	-0.2297484	0.05794077	0.3099782	-0.289238	0.07022464	0.29780368
D3Z6P0	Pdia2 Pdip	-0.0100986	0.00398478	0.3306779	-0.0114845	0.00357964	0.42370352	-0.0119845	0.00458778	0.34422506
P27773	Pdia3 Erp Erp	-0.2972361	0.01109298	0.85577558	-0.1797983	0.00870593	0.77335428	-0.2121516	0.01381972	0.67590615
P08003	Pdia4 Cai Erp	-0.3725678	0.05462454	0.63275053	-0.2560982	0.04837274	0.50026103	-0.233278	0.04099545	0.54530119
Q921X9	Pdia5 Pdir	-0.1010943	0.00223717	0.96869078	-0.0631007	0.00147871	0.96758734	-0.0820828	0.00828419	0.63267466
Q922R8	Pdia6 Txndc7	-0.3991246	0.03315806	0.61422714	-0.1880553	0.01513703	0.62653865	-0.2238202	0.02290826	0.50922459
Q9JIL4	Pdzk1 Cap70	-0.6380044	0.05167775	0.84951458	-0.4027938	0.05328837	0.67110984	-0.6433858	0.08071824	0.70176628
P70296	Pebp1 Pbp Pe	-0.1678018	0.01094752	0.84529184	-0.0875794	0.002645	0.96057331	-0.1319602	0.01020672	0.77690271
Q99MZ7	Pecr	-0.517447	0.06223054	0.84173219	-0.1155211	0.01436145	0.82211655	-0.5310313	0.12321813	0.58826082
P62962	Pfn1	-0.2147474	0.00729285	0.96763695	-0.1701528	0.02620261	0.55362189	-0.2033209	0.01422196	0.85737244
Q9DBJ1	Pgam1	-0.2049093	0.01552152	0.93058634	-0.2041924	0.05764911	0.47260698	-0.2880986	0.11480982	0.32631541
Q9DCD0	Pgd	-0.1539466	0.02237835	0.78449818	-0.0631912	0.00954681	0.75783737	-0.3570521	0.1176713	0.41460123
P09411	Pgk1 Pgk-1	-0.2014274	0.02908363	0.48962242	-0.1163916	0.01760941	0.44268301	-0.1247931	0.00730289	0.8342886
Q9CQ60	Pgls	-0.1829434	0.02552299	0.82365357	-0.1095129	0.01844518	0.79661231	-0.1345511	0.01883001	0.85014782
Q9D0F9	Pgm1 Pgm2	-0.1390549	0.05141397	0.23359252	-0.1179572	0.0455404	0.19329183	-0.1566313	0.0445808	0.32193065
O55022	Pgrmc1 Pgrm	-0.6496444	0.04395465	0.84197026	-0.5015177	0.04490485	0.76649058	-0.6282624	0.07061559	0.65877541
Q80UU9	Pgrmc2	-0.642408	0.06622272	0.88690336	-0.3498521	0.0716656	0.70441559	-0.4727653	0.08133591	0.73790657
P67778	Phb	-0.1117716	0.00090953	0.99283399	-0.0680192	0.00145966	0.9513691	-0.0982214	0.00438099	0.82584489
O35129	Phb2 Bap Bca	-0.111128	0.00155054	0.99227304	-0.06611	0.00134412	0.98533673	-0.0966217	0.00680113	0.85221524
Q7M6Y3	Picalm Calm	-0.3206468	0.02748892	0.96455471	NA	NA	NA	-0.3673304	0.03034172	0.94214677
Q9D826	Pipox Pso	-0.3473614	0.02130583	0.95680453	-0.2784738	0.01450976	0.98135021	-0.3760435	0.04577644	0.83847452
P53810	Pitpna Pitpn	-0.1246313	0.01788458	0.82924083	-0.0849303	0.01133616	0.83613831	-0.0776585	0.0132385	0.8309639
P53811	Pitpnb	-0.2314037	0.03608054	0.82047887	-0.1982239	0.03178336	0.7954871	-0.2813847	0.06603295	0.81948227
P53657	Pklr	-0.2307993	0.0208374	0.58230959	-0.1296052	0.02105954	0.30574902	-0.1887879	0.00828561	0.84120741
P52480	Pkm Pk3 Pkm	-0.2755494	0.0507137	0.40154119	-0.2607068	0.05269831	0.34728042	-0.278507	0.05982314	0.33512427
Q99P27	Pla2g12b Pla	-0.6199684	0.2626315	0.6500413	-0.4192062	0.20260028	0.46128214	-0.1859957	0.04943477	0.66912467
Q8VCI0	Plbd1	-0.1076324	0.08149982	0.11829171	-0.138086	0.07198061	0.20815251	-0.2930967	0.100555	0.39523627
Q8BG07	Pld4	-0.0779235	0.02384405	0.34811229	-0.0819999	0.01705596	0.52396006	-0.07972	0.01873516	0.45145178
Q9QXS1	Plec Plec1	-0.2645656	0.01696782	0.9681423	-0.1676706	0.00441533	0.99242984	-0.21478	0.02274568	0.91766505
P20918	Plg	-0.4997998	0.14543953	0.62784536	-0.3644763	0.24649615	0.42155769	-0.4120284	0.16412863	0.51227855
P43883	Plin2 Adfp Ad	-0.4801802	0.06942845	0.799444	-0.3615356	0.14325715	0.38908821	-0.7931291	0.15165408	0.69505535
Q99K51	Pls3	-0.26101	0.00770222	0.9905121	-0.1596426	0.00527324	0.98814045	-0.3794459	0.0618765	0.77368695
Q8C165	Pm20d1	-0.4182195	0.03934131	0.85606985	-0.2955038	0.04582152	0.70984732	-0.3198141	0.05071324	0.68841804
Q6P8U6	Pnlip	-0.309753	0.06416181	0.34627449	-0.2684775	0.06837955	0.24698435	-0.0969848	0.04420889	0.11510124
P23492	Pnp Np Pnp1	-0.2653462	0.0992214	0.35489609	-0.0883432	0.01251939	0.78054458	-0.1200168	0.0126662	0.89085404
P52430	Pon1 Pon	-0.8159866	0.04495608	0.83521353	-0.4717664	0.02585227	0.85384975	-0.8376406	0.05802506	0.77935142
Q62086	Pon2	-0.1594429	0.03629104	0.76286843	-0.180275	0.01527996	0.96532493	-0.1378102	0.03266702	0.71770643
Q62087	Pon3	-0.2287958	0.01754407	0.90912636	-0.2098236	0.00640062	0.99444776	-0.1940183	0.0325521	0.78033779
P37040	Por	-0.4516619	0.02309528	0.73910774	-0.2496731	0.01453789	0.68282982	-0.3835266	0.02815039	0.58625338
Q9D819	Ppa1 Pp Pyp	-0.1556361	0.00988534	0.95750887	-0.1030102	0.00666913	0.97546748	-0.1452925	0.01776991	0.88134821
P17742	Ppia	-0.2179606	0.00536158	0.98392486	-0.1318423	0.00470198	0.96442705	-0.2362327	0.0447578	0.50781595
P24369	Ppib	-0.611366	0.05506011	0.87882257	-0.175582	0.01927355	0.85565828	-0.2523372	0.07325628	0.44165552
P35700	Prdx1 Msp23	-0.193248	0.00250641	0.99083277	-0.1231874	0.00156281	0.99075149	-0.1861413	0.00998539	0.86335418
P20108	Prdx3 Aop1 M	-0.0795077	0.02266595	0.48626107	-0.0676062	0.01437046	0.61253746	-0.0852663	0.01746957	0.64695673
O08807	Prdx4	-0.415069	0.04764188	0.73051966	-0.2056833	0.03287008	0.59187283	-0.3345988	0.06059924	0.52126088
P99029	Prdx5 Prdx6	-0.1514402	0.00453634	0.97891924	-0.0934609	0.00211783	0.98782651	-0.1474874	0.01358243	0.81933303
O08709	Prdx6 Aop2 L	-0.171091	0.00481499	0.95175613	-0.1017905	0.00179142	0.98086053	-0.1542552	0.0083245	0.85549515
Q9JK53	Prelp	-0.1126943	0.03779795	0.81633346	-0.0349688	0.00690717	0.92761673	NA	NA	NA
Q9WU79	Prodh Pro1	-0.6119337	0.06340107	0.69960276	-0.3473509	0.04817616	0.55311711	-0.5063293	0.06989187	0.57368806
P07146	Prss2 Try2	-0.0728613	0.03076226	0.4448818	-0.0534799	0.02479246	0.36774388	-0.0521431	0.04133957	0.2095874
Q61096	Prtn3	NA	NA	NA	NA	NA	NA	NA	NA	NA
Q61207	Psap Sgp1	-0.7233849	0.04334238	0.91462998	-0.3439452	0.04611801	0.66515516	-0.7312794	0.07711871	0.7757037
P49722	Psma2 Lmpc3	-0.2098987	0.00468781	0.99503683	-0.1407261	0.00467752	0.99779528	-0.1780183	0.03123255	0.84410459
Q9R1P1	Psmb3	-0.4369643	0.06581201	0.77226569	-0.1263089	0.00557185	0.97347931	-0.3319228	0.10309367	0.44363542
O35593	Psmd14 Pad1	-0.2798636	0.0386551	0.85346273	-0.1713466	0.00773902	0.99190622	-0.2332249	0.03443639	0.8514902
Q8VDM4	Psmd2	-0.4568407	0.0625869	0.76905254	-0.1571991	0.00665855	0.97379304	-0.4482815	0.09823904	0.59796136
P97371	Psme1	-0.6585553	0.22622207	0.62892898	-0.581426	0.35012959	0.57962001	-0.9877842	0.35308558	0.79646748
P97372	Psme2 Pa28b	-0.2230067	0.00542111	0.98889688	-0.1408126	0.00345387	0.99223953	-0.1721213	0.02062448	0.82279378
P17225	Ptbp1 Ptb	-0.2439323	0.01130659	0.95291254	-0.1535063	0.00500871	0.97813169	-0.3144772	0.07574721	0.46289023
Q60866	Pter	-0.1927634	0.00523968	0.99631932	-0.1363954	0.0121614	0.94728359	-0.2125804	0.03352355	0.83406331
Q8BWM0	Ptges2 Gbf1	-0.2822605	0.03046498	0.87735297	-0.1155081	0.02619399	0.6183888	-0.2231523	0.03262453	0.79586909
P22437	Ptgs1 Cox-1 C	-0.488039	0.07221965	0.70618509	-0.3214879	0.12903014	0.27953667	-0.6681617	0.12498401	0.61356371
Q8K2C9	ptplad1 hacd	-0.2031528	0.01676553	0.92444694	-0.1451877	0.00853668	0.95699005	-0.1656304	0.01837048	0.87136943
Q66GT5	Ptpmt1 Plip	-0.2082841	0.00899289	0.98169941	-0.1132832	0.00578535	0.97211082	-0.1862477	0.01809592	0.89824504
P06800	Ptprc Ly-5	-0.0595284	0.00925544	0.76088445	-0.0607168	0.01386547	0.59596728	-0.0445671	0.02101399	0.25705472
P32848	Pvalb Pva	-0.6990062	0.09113663	0.98328512	-0.4651508	0.67045918	0.19398059	NA	NA	NA

P42925	Pxmp2 Pmp2	-0.3790529	0.02790958	0.95843209	-0.2659406	0.01136582	0.98915949	-0.2775513	0.0339984	0.89282652
Q9EPB4	Pycard Asc	-0.0990327	0.01404631	0.98027955	-0.0414809	0.01108804	0.73677918	-0.0761987	0.00052403	0.99990542
Q9ET01	Pygl	-0.1043298	0.0101992	0.46374011	-0.0596492	0.01067849	0.22908892	-0.1134759	0.01105184	0.46355631
Q8BVI4	Qdpr Dhpr	-0.2004041	0.02223552	0.61899074	-0.103039	0.00947207	0.69882207	-0.2197728	0.03769144	0.41462494
Q8R404	Qil1	-0.619404	0.07028071	0.85662924	-0.3637032	0.05662591	0.77466394	-0.6565761	0.10275239	0.7585019
Q91X91	Qprt	-0.2670306	0.07016192	0.527015	-0.0876072	0.00965441	0.8546864	-0.1055977	0.01665426	0.75565297
P61027	Rab10	-0.2434356	0.01198488	0.9717363	-0.1996248	0.01135239	0.96564764	-0.221542	0.02930932	0.86391426
Q91V41	Rab14	-0.2637218	0.01191269	0.97609973	-0.1643832	0.00631805	0.98258188	-0.2619481	0.02877783	0.87349
P35292	Rab17	-0.1993544	0.01399299	0.98067348	NA	NA	NA	-0.2480452	0.12591187	0.56400827
P62821	Rab1A Rab1	-0.29507	0.01032054	0.98434527	-0.2079275	0.00512248	0.9921717	-0.2957237	0.03899922	0.83941377
P51150	Rab7a Rab7	-0.7841697	0.08106346	0.77607701	-0.618347	0.06250017	0.7837956	-0.7441156	0.08561657	0.74393813
Q99JI6	Rap1b	-0.1854608	0.00905829	0.97442993	-0.1016617	0.01459746	0.77600754	-0.1723679	0.01499793	0.91671526
Q8BU31	Rap2c	-0.3252858	0.06330765	0.6700579	-0.3673217	0.10502791	0.48477327	-0.3252789	0.07281821	0.64463487
O89086	Rbm3	-0.1920182	0.02302367	0.95865274	-0.2193943	0.02343032	0.96691619	-0.2456902	0.06849473	0.92788392
Q9QYF1	Rdh11 Arsdr1	-0.1354151	0.03603289	0.93387683	-0.9120142	0.31197385	0.89524493	-0.5845104	0.15649462	0.87461103
O88451	Rdh7 Crad2	-0.4967051	0.03808607	0.80960043	-0.2963486	0.03378479	0.6636249	-0.4597089	0.06196051	0.59160552
P26043	Rdx	-0.252725	0.01552265	0.87174112	-0.1170431	0.01400729	0.63576957	-0.1756943	0.02347865	0.58946313
Q9JM62	Reep6 Dp1l1	-0.2897726	0.02824918	0.89003657	-0.2304089	0.01772986	0.92344864	-0.1997697	0.025136	0.86332003
Q64FW2	Retsat Ppsig	-0.3068227	0.03458964	0.90771006	-0.4225165	0.13047342	0.72388666	-0.4666314	0.09341083	0.757243
Q64374	Rgn Smp30	-0.1344244	0.00202884	0.98607354	-0.0838822	0.00258038	0.93954202	-0.1297193	0.01078809	0.69987984
Q9CXW4	Rpl11	-0.122486	0.00198075	0.9966119	-0.0801832	0.00151396	0.99503379	-0.0916474	0.01013295	0.86287346
P35979	Rpl12	-0.0926812	0.00127988	0.9922419	-0.075537	0.0011349	0.99016533	-0.0614162	0.00420747	0.8386281
P47963	Rpl13	-0.045007	0.00712212	0.75441015	-0.0590072	0.00897026	0.75554963	-0.0448006	0.00860951	0.67562904
Q9CR57	Rpl14	-0.1056766	0.00223723	0.99420725	-0.0842658	0.00510229	0.9511778	-0.0706747	0.00860984	0.83827034
Q9CZM2	Rpl15	-0.0752236	0.01057707	0.65197135	-0.0743514	0.00708177	0.79743739	-0.0621723	0.00912847	0.63208832
Q9CPR4	Rpl17	-0.1147911	0.00175783	0.99696081	-0.0749499	0.00152739	0.99421948	-0.0867271	0.01046957	0.84072583
P35980	Rpl18	-0.1187793	0.00154753	0.99779818	-0.0788395	0.00140982	0.99586017	-0.0918229	0.0109682	0.8435358
P62717	Rpl18a	-0.1209797	0.00269629	0.99358414	-0.0870963	0.0042926	0.96711142	-0.0847137	0.01038872	0.83646596
P84099	Rpl19	-0.1726207	0.00430435	0.99198179	-0.1193896	0.0030008	0.99123314	-0.1265774	0.01401173	0.86258981
P67984	Rpl22	-0.0979433	0.00146472	0.99399782	-0.0732944	0.00133563	0.99046185	-0.0660272	0.00611126	0.82360887
Q9D7S7	Rpl22l1	-0.310391	0.02512887	0.9682683	-0.8840939	0.24480607	0.76528916	-0.4698788	0.27410838	0.37015879
P62830	Rpl23	-0.1143042	0.00600052	0.93074554	-0.0991866	0.00702241	0.87692086	-0.0924975	0.00873666	0.80588203
Q8BP67	Rpl24	-0.1378845	0.00248189	0.99580576	-0.0957892	0.00235173	0.99163205	-0.108785	0.0125821	0.85185769
P61358	Rpl27	-0.1196782	0.00212924	0.99590194	-0.0881892	0.0021723	0.99157709	-0.0929654	0.01040706	0.8599093
P14115	Rpl27a	-0.1371236	0.00472471	0.98826723	-0.0794323	0.00384745	0.98611866	-0.2501543	0.09787565	0.44950246
P27659	Rpl3	-0.0924938	0.00131051	0.98089935	-0.0658346	0.00149959	0.95255429	-0.0628401	0.00307444	0.81156759
P62889	Rpl30	-0.1206283	0.00244667	0.97787432	-0.0778797	0.00275851	0.93655075	-0.0754352	0.00479746	0.81802765
P62900	Rpl31	-0.1224706	0.0025039	0.99500909	-0.0803728	0.00192666	0.9942865	-0.0831771	0.01007342	0.83986099
Q9D8E6	Rpl4	-0.1123964	0.00143231	0.99563453	-0.0822479	0.00142246	0.99198878	-0.0847196	0.00688511	0.8486609
P47962	Rpl5	-0.0930898	0.00280005	0.98837503	-0.0771072	0.00322813	0.97604964	-0.0605364	0.0078308	0.82133408
P47911	Rpl6	-0.1086179	0.00620421	0.89751098	-0.078079	0.00186572	0.98040711	-0.0821678	0.00740434	0.78866305
P14148	Rpl7	-0.1154737	0.00160214	0.9921692	-0.0850094	0.00203422	0.97542417	-0.0878489	0.0059898	0.83990844
P12970	Rpl7a Surf-3	-0.1315468	0.00243233	0.99085342	-0.0856933	0.00178649	0.98840147	-0.0945926	0.00810131	0.83469436
P62918	Rpl8	-0.0951956	0.00332102	0.95579627	-0.0631383	0.00345904	0.93020203	-0.0695885	0.00669795	0.75514575
P51410	Rpl9	-0.1078866	0.00316821	0.93925164	-0.100969	0.0208006	0.24151304	-0.0975442	0.01015111	0.56187567
P14869	Rplp0 Arbp	-0.0921982	0.00178884	0.97468303	-0.0760411	0.00235778	0.93525971	-0.0714705	0.00636289	0.64645606
P47955	Rplp1	-0.1140417	0.00181605	0.99319968	-0.0934577	0.00361243	0.95984613	-0.0916899	0.0083777	0.82732716
P99027	Rplp2	-0.1446211	0.00194909	0.99386231	-0.1048049	0.00299893	0.97680603	-0.1113803	0.00716071	0.87678427
Q91YQ5	Rpn1	-0.125579	0.0012052	0.98861789	-0.0729972	0.00100458	0.97633197	-0.1045028	0.00415408	0.8383802
Q9DBG6	Rpn2	-0.1414437	0.00151418	0.98587722	-0.1064821	0.01152953	0.40368069	-0.105643	0.00438859	0.83201037
P63325	Rps10	-0.1039237	0.00389804	0.9833975	-0.0815427	0.00606294	0.96789484	-0.0834938	0.01234879	0.8060479
P62281	Rps11	-0.5460278	0.07993239	0.79544594	-0.516246	0.11089001	0.64363646	-0.4770311	0.12834846	0.51517439
P63323	Rps12	-0.0851464	0.00179317	0.99470599	-0.0522978	0.00327743	0.97697841	-0.0504867	0.00633816	0.82995313
Q6ZWZ6	Rps12 LOC10	-0.0875427	0.00183122	0.99434386	-0.0609365	0.00219605	0.9909906	-0.0561617	0.0065339	0.85037055
P62264	Rps14	-0.1230062	0.0026602	0.98752941	-0.0884567	0.00289458	0.97089023	-0.0922212	0.00800597	0.84145978
P62843	Rps15 Rig	-0.1239423	0.00332511	0.98093753	-0.0990922	0.00313096	0.97186294	-0.0895	0.00762343	0.83619534
P62245	Rps15a	-0.1084037	0.00343139	0.98714197	-0.0912055	0.00355647	0.97915628	-0.1017821	0.01152488	0.85713579
P14131	Rps16	-0.1124942	0.00320525	0.98955649	-0.0728616	0.00541566	0.9282076	-0.0784356	0.00926007	0.8466007
P63276	Rps17	-0.1254824	0.0018152	0.99438177	-0.089425	0.00199361	0.98869801	-0.0952526	0.00779098	0.84700398
P62270	Rps18	-0.1038158	0.00169586	0.99654305	-0.0748556	0.00153144	0.99500245	-0.0774684	0.00874611	0.85785327
Q9CZX8	Rps19	-0.1015551	0.00218004	0.98771095	-0.0795314	0.00263653	0.96911405	-0.0732824	0.04162177	0.10298928
P25444	Rps2 Llrep3 F	-0.1182558	0.00238798	0.9768957	-0.0795371	0.00214765	0.97166256	-0.0838587	0.00520072	0.83066948
P62267	Rps23	-0.1039837	0.00246569	0.98504568	-0.0767999	0.0021488	0.98231319	-0.0797143	0.00681746	0.84021473
P62849	Rps24	-0.0986713	0.00347588	0.984124	-0.0665797	0.00385062	0.95526668	-0.0606657	0.00725529	0.84321479
P62855	Rps26	-0.117139	0.00364235	0.97455918	-0.1403061	0.03152556	0.40582774	-0.0838522	0.00800881	0.80237265
P62858	Rps28	-0.12163	0.00276472	0.99485977	-0.0877687	0.00985685	0.94067899	-0.0889404	0.01476668	0.81931965

P62908	Rps3	-0.1182834	0.00213373	0.98398991	-0.1023035	0.00360773	0.94366897	-0.0867739	0.00546102	0.82921834
P97351	Rps3a	-0.1039342	0.00208546	0.98532199	-0.0768692	0.00198332	0.97914188	-0.0722614	0.00579032	0.79973611
P62702	Rps4x Rps4	-0.0990436	0.00360648	0.97416678	-0.0658673	0.00287375	0.96687156	-0.0618331	0.00831984	0.75421539
P97461	Rps5	-0.1095609	0.00486203	0.949512	-0.0906374	0.00618547	0.89571101	-0.0709032	0.00624778	0.82668931
P62754	Rps6	-0.114425	0.00336543	0.98887947	-0.0762795	0.00499155	0.94344156	-0.0774754	0.00973711	0.82964076
P62082	Rps7	-0.1552138	0.01857903	0.57304762	-0.2002307	0.0402978	0.40680899	-0.1454374	0.02459998	0.43176804
P62242	Rps8	-0.0968681	0.00149368	0.9870915	-0.0747082	0.0014631	0.9778719	-0.0623886	0.00378896	0.83135357
P14206	Rpsa Lamr1 F	-0.0952755	0.00144172	0.99385549	-0.0689555	0.00169046	0.98753642	-0.067102	0.00540796	0.85552205
Q99PL5	Rrbp1	-0.7248926	0.0498601	0.77889873	-0.4346856	0.03791055	0.67953877	-0.721509	0.05637707	0.72540449
Q01730	Rsu1 Rsp1	-0.2249819	0.0099663	0.9826455	-0.1334236	0.00477475	0.98735527	-0.2184436	0.03227405	0.83579996
P50543	S100a11 S10	-0.4097857	0.11182171	0.72869656	-0.2034936	0.02950858	0.95964157	-0.2590646	0.05654705	0.83993093
P27005	S100a8 Caga	-0.3029676	0.08038468	0.41529265	-0.2095977	0.02109636	0.82457492	-0.3770537	0.06724765	0.58830645
Q60710	Samhd1 Mg1	-0.1154138	0.02730132	0.51248847	-0.0760101	0.0092477	0.93108939	-0.0968401	0.02061056	0.64785133
Q8BGH2	Samm50	-0.1260278	0.00657565	0.98922789	-0.0714058	0.00517324	0.96456071	-0.1177964	0.0157215	0.90344451
Q9CQC9	Sar1b Sara1b	-0.3275182	0.01468368	0.95765233	-0.2553126	0.01184137	0.97076512	-0.3120924	0.02507371	0.9063932
Q99LB7	Sardh	-0.2241846	0.02337725	0.42192679	-0.0893496	0.0144373	0.22623138	-0.1523607	0.02098142	0.29176997
Q61009	Scarb1 Srb1	-0.3207562	0.0247785	0.95443451	-0.2891323	0.00391674	0.99981653	-0.3481712	0.02504455	0.94153973
P32020	Scp2 Scp-2	-0.661636	0.03746349	0.74457198	-0.543894	0.03486996	0.68864167	-0.64438	0.0394372	0.71388527
Q8K2B3	Sdha	-0.1809549	0.00307443	0.95506271	-0.0939518	0.00133015	0.96611114	-0.1716184	0.00959944	0.66640354
Q9CQA3	Sdhb	-0.1601553	0.00288696	0.97497248	-0.0856228	0.00298507	0.90736216	-0.1507582	0.02062281	0.40657313
Q8VBT2	Sds	-0.5032803	0.03317188	0.72116608	-0.2436938	0.02436866	0.50762912	-0.500433	0.04393513	0.59584509
Q99J08	Sec14l2	-0.3804437	0.03339234	0.65955937	-0.2456315	0.03000511	0.48911023	-0.338606	0.04791182	0.46269627
O08547	Sec22b Sec22	-0.6200625	0.11714387	0.58348652	-0.8043235	0.14462505	0.65906391	-0.6766639	0.12729823	0.5855385
Q01405	Sec23a Sec23	-0.1922108	0.01091367	0.97180277	-0.1382161	0.00677834	0.9788129	-0.1765365	0.03081302	0.8913772
Q3UPL0	Sec31a Sec31	-0.3379143	0.06149851	0.71558213	-0.2128715	0.00675827	0.98903421	-0.251038	0.03164513	0.86288419
P61620	Sec61a1 Sec6	-0.2427993	0.00341141	0.9960673	-0.158755	0.0047871	0.98566041	-0.1968438	0.02272599	0.80650069
P17563	Selenbp1 Lps	-0.157697	0.00516735	0.98623384	-0.0986843	0.00251921	0.99159939	-0.1459681	0.01947793	0.82394467
Q9CY58	Serbp1 Pairb	-0.223901	0.01887092	0.92145322	-0.158172	0.00689394	0.98135758	-0.1998726	0.03283313	0.75539162
Q00897	Serpina1d Dc	-0.3770636	0.0621056	0.75440565	-0.2355055	0.01502927	0.95340574	-0.823994	0.08992976	0.86591577
P07759	Serpina3k M	-0.160871	0.07027606	0.28728521	-0.0392112	0.01170779	0.46318343	-0.0877218	0.02582327	0.49022189
Q99JR1	Sfxn1 F	-0.137424	0.00225373	0.98490102	-0.0662304	0.00202824	0.94756901	-0.1243821	0.00733689	0.85180943
Q925N2	Sfxn2	-0.1116407	0.01484134	0.8131772	-0.2235003	0.06885089	0.4294445	-0.1078989	0.02788896	0.53518609
Q91V61	Sfxn3	-0.1655106	0.00708253	0.98201774	-0.0925691	0.00933182	0.91620191	-0.1893585	0.01722934	0.92354176
Q8R0X7	Sgpl1	-0.3280085	0.01373596	0.9810747	-0.2238776	0.0092499	0.97992632	-0.4097256	0.12387093	0.5224623
P50431	Shmt1 Shmt	-0.1194316	0.04572393	0.22877344	-0.0148288	0.00636917	0.22196789	-0.0728912	0.01374522	0.47566071
Q62230	Siglec1 Sa Sn	-0.2629152	0.25895856	0.20489647	-0.3967425	0.13761142	0.62440062	-0.1200321	0.04487205	0.78155318
O55242	Sigmar1 Oprs	-0.6567954	0.08005098	0.83814203	-0.3941306	0.0760838	0.65715393	-0.6338995	0.11112417	0.71453962
O08705	Slc10a1 Ntcp	-0.9626211	0.08398213	0.90996129	-0.5053663	0.07033716	0.78666007	-1.0065316	0.11512722	0.85464492
P53986	Slc16a1 Mct1	-0.2050559	0.00565716	0.99020239	-0.1324177	0.00333028	0.99122257	-0.1935169	0.01803027	0.89859192
Q9QZD8	Slc25a10 Dic	-0.2769274	0.02142485	0.72302668	-0.1333974	0.00273314	0.97383657	-0.2101723	0.01375418	0.81217205
Q9CR62	Slc25a11	-0.1389729	0.0033616	0.98444791	-0.0778291	0.00162351	0.98753834	-0.1177506	0.00939871	0.85322914
Q8BH59	Slc25a12 Ara	-0.1009841	0.01292361	0.67798336	-0.0537031	0.00580381	0.72793597	-0.0736868	0.01370651	0.50792476
Q9QXX4	Slc25a13 Ara	-0.1369561	0.00939004	0.54724334	-0.0822701	0.00851102	0.34171391	-0.1295971	0.01148917	0.42663009
Q9WVD5	Slc25a15 Orr	-0.1905472	0.0051446	0.95409758	-0.1107915	0.00313185	0.95207095	-0.1948815	0.0178628	0.66116035
Q9Z2Z6	Slc25a20 Cac	-0.1211893	0.0042193	0.95265523	-0.0688356	0.00184663	0.96998311	-0.1068723	0.00764956	0.82992487
Q9D6M3	Slc25a22 Gc1	-0.25412	0.01585561	0.82364387	-0.13988	0.0062736	0.89713779	-0.2589857	0.04405537	0.39468992
Q8VEM8	Slc25a3	-0.2000549	0.0301455	0.57917229	-0.0958101	0.0020472	0.98782295	-0.1440793	0.01027917	0.87137749
Q8R0Y8	Slc25a42	-0.1503257	0.00454074	0.9882778	-0.0870012	0.00485493	0.9816588	-0.1414797	0.01519644	0.87839132
P51881	Slc25a5 Ant2	-0.1357681	0.00203941	0.99083365	-0.0859179	0.00383543	0.91938566	-0.1545089	0.0310127	0.37710308
O35488	Slc27a2 Acsv	-0.4996673	0.02422182	0.74195697	-0.3197874	0.01937749	0.64180535	-0.410309	0.03028393	0.56211296
Q4LDG0	Slc27a5 Acsb	-0.554852	0.02476622	0.77706228	-0.2952476	0.01351218	0.77076188	-0.4909138	0.03105164	0.64262146
P14246	Slc2a2 Glut-2	-0.2168143	0.03554409	0.7718241	-0.240355	0.01151962	0.98417513	-0.2239795	0.04233228	0.71790906
Q75N73	Slc39a14 Fad	-0.8860547	0.10320106	0.90209812	-0.4457379	0.06904052	0.80650994	-0.7755113	0.16249153	0.67434388
P10852	Slc3a2 Mdu1	-0.2685175	0.11215242	0.32326838	-0.1472689	0.08278185	0.14278648	-0.1455363	0.06845238	0.21004753
P04919	Slc4a1 Ae1	-0.0159343	0.00150087	0.69699672	-0.0103804	0.00084667	0.74666176	-0.014383	0.00157678	0.62464221
Q9JJL3	Slco1b2 Oatp	-0.8023103	0.07221942	0.82049963	-0.4731646	0.05034446	0.75283936	-0.9826615	0.0980472	0.78814742
Q78PY7	Snd1	-0.2700411	0.01841072	0.62880535	-0.19666	0.01866405	0.46255564	-0.2747672	0.02463795	0.50074784
P62315	Snrpd1	-0.0771905	0.00442747	0.97124229	-0.0409885	0.00179144	0.97942019	-0.0758738	0.00685376	0.93872247
P08228	Sod1	-0.1460394	0.00282985	0.97976644	-0.0917038	0.00136487	0.987099	-0.1141504	0.00792879	0.79331912
Q64442	Sord Sdh1	-0.1856891	0.01761796	0.55243176	-0.104419	0.00438151	0.85799592	-0.1605451	0.01419237	0.57135776
Q9CYN2	Spcs2 Spc25	-0.8535496	0.0733976	0.91230227	-0.3736591	0.08010222	0.62600864	-0.4838343	0.10270841	0.64903275
Q64105	Spr	-0.1282293	0.00982273	0.91417043	-0.0872673	0.00838125	0.87845791	-0.1509163	0.00961667	0.93542905
P08032	Spta1 Spna1	-0.0213907	0.00317983	0.68303122	-0.0232545	0.00323892	0.76313169	-0.0322688	0.02323681	0.0967696
P16546	Sptan1 Spna2	-0.1860657	0.00291705	0.98213683	-0.1315385	0.0034144	0.95618964	-0.2099564	0.02036454	0.63537248
Q9R112	Sqrdl	-0.3413142	0.07076176	0.37364942	-0.2595673	0.05907525	0.32553023	-0.4084853	0.07282989	0.44648012
P47758	Srprb	-0.2378515	0.0081758	0.98487232	-0.1378597	0.00444856	0.98563165	-0.1964301	0.02403885	0.83703385

Q62093	Srsf2 Pr264 S	-0.2762365	0.06469458	0.58375589	-0.1122478	0.08793545	0.11137847	-0.2627827	0.06822882	0.5528058
Q9CYR0	Ssbp1	-0.2203022	0.08526458	0.33928828	-0.1848334	0.10495927	0.18134036	-0.1268474	0.01218553	0.89288179
Q9CY50	Ssr1	-0.3659608	0.08431922	0.59167247	-0.2531494	0.062024	0.54335578	-0.2317511	0.09327352	0.32197866
Q9DCF9	Ssr3	-0.1329781	0.01196143	0.96865031	-0.1239559	0.02742766	0.83623186	-0.1875843	0.02501422	0.93359518
Q62186	Ssr4	-0.1546446	0.00301534	0.98465142	-0.0986816	0.00298193	0.96221981	-0.1280599	0.00991495	0.80271289
Q9JMD3	Stard10 Pctp	-0.2319921	0.0197774	0.77915781	-0.1625378	0.02071601	0.58317435	-0.2229639	0.0425258	0.40731383
P42225	Stat1	-0.5638894	0.30244354	0.33181601	-0.1504917	0.07363122	0.31700998	NA	NA	NA
Q923B6	Steap4 Tiarp	-0.6037454	0.06159443	0.72743419	-0.3659086	0.04789174	0.61205604	-0.6547412	0.08624351	0.63590235
P54116	Stom Epb7.2	-0.1787372	0.01019804	0.95939815	-0.1463501	0.00877113	0.95212105	-0.1745303	0.01829943	0.88345367
Q99JB2	Stoml2 Slp2	-0.1451216	0.00512117	0.92939962	-0.0933223	0.00467702	0.86903469	-0.1351739	0.00806109	0.82900369
P50427	Sts	-0.4509869	0.076672	0.59043154	-0.2840922	0.05649307	0.52369919	-0.3042579	0.06081627	0.50029015
P46978	Stt3a Itm1	-0.132096	0.02476868	0.739874	-0.0536293	0.01713816	0.52107491	-0.1070528	0.03719101	0.45311943
Q3TDQ1	Stt3b Simp	-0.1966123	0.00465598	0.99276249	-0.1470502	0.00877193	0.95578568	-0.1515072	0.01988433	0.82870766
Q9Z2I9	Sucla2	-0.1325678	0.00424133	0.98686804	-0.0636028	0.00673242	0.88148218	-0.1055312	0.01318345	0.84226542
Q9WUM5	Suclg1	-0.2355934	0.04430931	0.44682113	-0.1323677	0.03336804	0.30416361	-0.2396382	0.04731746	0.40940732
Q9Z2I8	Suclg2	-0.1795763	0.02533753	0.53306112	-0.0606137	0.00544288	0.71679216	-0.122522	0.01147809	0.69928221
P52840	Sult1a1 St1a1	-0.2656212	0.00762901	0.98138014	-0.1824902	0.00594982	0.97210005	-0.2765447	0.04776633	0.60373699
P52843	Sult2a1 Sta1	-0.1917554	0.01427064	0.93283541	-0.2369681	0.05668875	0.55518481	-0.1787979	0.08773683	0.24211468
Q8R086	Suox	-0.1497969	0.00381304	0.96018279	-0.1055162	0.00509656	0.87185541	-0.1322596	0.00818901	0.82586714
Q64310	Surf4 Surf-4	-0.2441296	0.01241597	0.97233509	-0.1806709	0.00499199	0.99242352	-0.2024346	0.02810387	0.85217903
Q9WVA4	Tagln2 Kiaa0	-0.3607732	0.08002642	0.49181638	-0.5486839	0.09371704	0.69560053	-0.4119268	0.10210941	0.47482867
Q93092	Taldo1 Tal Ta	-0.0324145	0.00820097	0.69057187	-0.0222467	0.00756836	0.81203443	-0.1298457	0.20356097	0.07525222
P21958	Tap1 Abcb2 I	-0.0984532	0.00985724	0.87693206	-0.0579944	0.00683104	0.82773929	-0.0667985	0.01289114	0.69112308
P36371	Tap2 Abcb3 I	-0.060039	0.02567983	0.28080343	-0.0138713	0.0072491	0.20731884	-0.0237296	0.01290279	0.20646171
Q9R233	Tapbp Tapa	-0.6305308	0.06511924	0.84650792	-0.2801669	0.01690808	0.97861458	-0.45129	0.04006274	0.90063228
Q9D0R2	Tars	-0.7123165	0.07157383	0.7857927	-0.5706528	0.11410037	0.51033636	-0.8073429	0.09063192	0.74612427
Q8QZR1	Tat	-0.1261961	0.03894765	0.63633158	-0.1021819	0.03519885	0.67812936	-0.1206208	0.03957159	0.60762066
Q9CY27	Tecr Gpsn2	-0.3980373	0.04518989	0.85648474	-0.2053547	0.00663293	0.98560434	-0.3167399	0.05080171	0.74938832
Q921I1	Tf Trf	-0.5983337	0.03660411	0.74180606	-0.2755589	0.02426155	0.59722354	-0.6821868	0.04943636	0.68393132
P82198	Tgfbi	-0.0666669	0.02827232	0.29957991	-0.1716738	0.13955314	0.11198687	-0.12818	0.03010557	0.58236789
Q9JLF6	Tgm1	-0.0776428	0.00681651	0.80714351	-0.0636998	0.00716122	0.72508069	-0.0739729	0.01116317	0.6562582
P21981	Tgm2	-0.256991	0.02741629	0.5673665	-0.2305412	0.03317434	0.4586582	-0.1700278	0.02310779	0.47433257
Q62264	Thrsp S14	-0.5575442	0.11023266	0.73975055	-0.2845729	0.0483509	0.89648033	-0.7430854	0.17098251	0.825232
P62075	Timm13 Tim1	-0.1822603	0.00361229	0.99491943	-0.092693	0.00203904	0.99327098	-0.1878933	0.02069167	0.86381412
Q9WV98	Timm9 Tim9	-0.1284195	0.0045837	0.9837078	-0.0614621	0.00237039	0.97960133	-0.1191379	0.01389229	0.84978883
P40142	Tkt	-0.1486234	0.05130147	0.18489555	-0.0831723	0.027578	0.17130641	-0.1152525	0.03017919	0.27735085
P26039	Tln1 Tln	-0.3581133	0.02294347	0.56838768	-0.2026288	0.01643048	0.45936204	-0.3877258	0.03079912	0.51878911
Q9D1D4	Tmed10 Tmp	-0.2925635	0.02528611	0.72412727	-0.2320472	0.02692107	0.57462109	-0.2654202	0.03811476	0.46856456
Q9CXE7	Tmed5	-0.2170039	0.03866516	0.88732045	-0.2411871	0.02579472	0.94590334	-0.2012895	0.03754673	0.87782805
Q91XE8	Tmem205	-0.4893605	0.0586942	0.84244947	-0.1931597	0.00400418	0.99401979	-0.5346392	0.09269021	0.71904089
Q5F285	Tmem256	-0.1633582	0.00222144	0.9976018	-0.0840251	0.00283327	0.98764755	-0.1608637	0.02472435	0.77913476
Q8VBT0	Tmx1 Txndc 1	-0.7756399	0.09300238	0.84253039	-0.6944075	0.09869702	0.81818714	-0.7102995	0.1044782	0.78048024
Q921Z5	Tnfaip8	-0.1132326	0.02157531	0.87319355	-0.0611015	0.06640029	0.17470741	-0.0737059	0.10630043	0.19379771
Q9CZW5	Tomm70a D1	-0.6330334	0.07256003	0.75275166	-0.423356	0.06772854	0.66143133	-0.7384625	0.12090158	0.62905025
P17751	Tpi1 Tpi	-0.1422767	0.00298542	0.97635635	-0.0838311	0.00216424	0.96278162	-0.1266917	0.00804776	0.82381845
O89023	Tpp1 Cln2	-0.2699594	0.12756258	0.4725006	-0.104161	0.02413808	0.69948608	-0.0948095	0.02741785	0.7051437
P63028	Tpt1 Trt	-0.2513038	0.03049072	0.95770487	-0.1382233	0.01257798	0.95266826	-0.0991182	0.01985123	0.80601708
Q9CQN1	Trap1 Hsp75	-0.8629803	0.05034495	0.92740489	-0.5986199	0.05433898	0.85851833	-0.801061	0.11826728	0.74142558
Q9DCK3	Tspan4 Tm4s	-0.2185065	0.02536648	0.96114027	-0.2772568	0.04637242	0.92257552	-0.2606228	0.04251478	0.90379753
P52196	Tst	-0.1201395	0.00541992	0.94974333	-0.0704102	0.00336936	0.94380731	-0.1113545	0.00973769	0.8341504
Q8BWP5	Ttpa	-0.4188892	0.04426086	0.73076437	-0.2299809	0.04662621	0.43190836	-0.2980558	0.07670748	0.36736598
P07309	Ttr	-0.5332889	0.12262142	0.59266031	-0.1281255	0.0173003	0.79665469	-0.8408877	0.127963	0.76861096
P68373	Tuba1c Tuba	-0.1991072	0.0091971	0.97301089	-0.1707885	0.02185823	0.81345815	-0.3253514	0.11057015	0.39976658
P68368	Tuba4a Tuba	-0.2001082	0.00527174	0.9910583	-0.1502227	0.00568931	0.98031472	-0.2232762	0.04085608	0.69672675
P99024	Tubb5	-0.2341839	0.0412845	0.58315667	-0.228882	0.04275693	0.60130642	-0.425345	0.09525473	0.5255577
Q8BFR5	Tufm	-0.133733	0.0054542	0.91339962	-0.0823923	0.00408166	0.87728045	-0.1152128	0.0083004	0.77792608
P10639	Txn Txn1	-0.1559838	0.00278814	0.99586369	-0.0912907	0.00383557	0.97588251	-0.1334602	0.01304015	0.8895931
Q91W90	Txndc5 Tlp46	-0.1566968	0.07558516	0.24845977	-0.1702706	0.07622716	0.29368279	-0.164121	0.08478824	0.22373099
Q9JLT4	Txnrd2 Trxr2	-0.1090995	0.00612785	0.96060346	-0.0646674	0.00269873	0.97619789	-0.1006824	0.0123396	0.8366303
Q02053	Uba1 Sbx Ub	-0.2443245	0.0233383	0.77951046	-0.1313193	0.00358651	0.98025805	-0.1843762	0.01561647	0.85311575
P68037	Ube2l3 Ubce	-0.2486226	0.01896531	0.95023628	-0.2056671	0.00902152	0.98297779	-0.2094125	0.06495041	0.83864994
Q8CCJ3	Ufl1 Kiaa077	-0.3129063	0.05047409	0.66917332	-0.2790997	0.05254398	0.70160037	-0.3120316	0.04870191	0.71954061
P61961	Ufm1	-0.6060452	0.071486	0.84683063	-0.1814729	0.00798685	0.97359812	-0.629997	0.12708816	0.65401138
O70475	Ugdh	-0.4654285	0.0737591	0.62392806	-0.312974	0.04776525	0.58866422	-0.439545	0.08889847	0.46612347
Q6P5E4	Uggt1 Gt Ugg	-0.0594341	0.02474472	0.49018949	-0.056534	0.02000481	0.61498146	-0.0566767	0.01244194	0.74775414
Q91ZJ5	Ugp2	-0.3898071	0.04451649	0.64609433	-0.2814	0.03427809	0.63944496	-0.4452022	0.07123171	0.53464948

Q63886	Ugt1a1 Ugt1	-0.3529098	0.02284149	0.74899154	-0.2429496	0.01879209	0.67904552	-0.2649058	0.02062951	0.67887235
P70691	Ugt1a2 Ugt1	-0.835092	0.097075	0.86047124	-0.7043419	0.14665781	0.62228691	-0.7096167	0.17620929	0.55506467
Q64435	Ugt1a6 Ugt1	-0.3470014	0.03770635	0.82471561	-0.2726457	0.05077035	0.62913505	-0.2139274	0.02488108	0.77877391
Q62452	Ugt1a9 Ugt1	-0.6251519	0.09486006	0.62552996	-0.4266691	0.0610134	0.62774027	-0.3909733	0.08386051	0.42013278
Q8BWQ1	Ugt2a3	-0.2970574	0.02338283	0.65769298	-0.1902278	0.01737132	0.56855155	-0.246094	0.02106325	0.61905766
Q8R084	Ugt2b1 mCG	-0.2447155	0.01480887	0.91921177	-0.273309	0.04569092	0.5609954	-0.2784789	0.0477561	0.62965542
P17717	Ugt2b17 Ugt	-0.4348065	0.03645012	0.7286177	-0.2299591	0.0273155	0.59123519	-0.2981552	0.03423408	0.57967806
Q8JZZ0	Ugt3a2	-0.2831674	0.04748813	0.56838866	-0.2348194	0.04236716	0.52315382	-0.3206781	0.0697366	0.4485153
P25688	Uox	-0.754772	0.02757223	0.87098358	-0.5498479	0.03440098	0.71466307	-0.6902845	0.04112211	0.72852569
Q9D855	Uqcrb	-0.2053596	0.00360883	0.99173084	-0.134434	0.00323143	0.98352015	-0.2619414	0.03986684	0.61522173
Q9CZ13	Uqcrc1	-0.1350085	0.00097386	0.99271694	-0.082489	0.0008944	0.98233051	-0.1257887	0.00459531	0.84733607
Q9DB77	Uqcrc2	-0.1482336	0.00124049	0.98994748	-0.0866123	0.00079646	0.98731	-0.1414177	0.00474805	0.8653801
Q9CR68	Uqcrfs1	-0.2529903	0.02382908	0.75286873	-0.243968	0.05100857	0.41686667	-0.2762978	0.04831457	0.47601174
P99028	Uqcrh	-0.1273613	0.00134156	0.99855967	-0.0731326	0.00127846	0.99573983	-0.1157489	0.01247225	0.86885631
Q9CQ69	Uqcrq	-0.2572856	0.00799957	0.98758858	-0.1554529	0.00499206	0.98576805	-0.6476725	0.11023975	0.72641434
Q8VC12	Uroc1	-0.2878163	0.03349398	0.63197827	-0.1159546	0.00284835	0.97470976	-0.1756837	0.01202922	0.85217643
Q78IK2	Usmg5 Dapit	-0.1555045	0.00287175	0.99558603	-0.0839885	0.00316513	0.98050509	-0.1354218	0.01568722	0.85146613
Q62465	Vat1 Vat-1	-0.535329	0.21439166	0.75712966	-0.2037596	0.12367544	0.31148219	-0.1166455	0.04441785	0.87335962
Q64727	Vcl	-0.134033	0.00209935	0.99052293	-0.0973372	0.0028951	0.96582367	-0.1505471	0.03288721	0.37449887
Q01853	Vcp	-0.3201897	0.02611092	0.6229904	-0.2090638	0.01994825	0.5441864	-0.3254668	0.03353906	0.51693419
Q60932	Vdac1 Vdac5	-0.2190026	0.01433928	0.66787115	-0.102654	0.00816577	0.55639467	-0.2105309	0.02149589	0.47038719
Q60930	Vdac2 Vdac6	-0.1765913	0.00266927	0.98758957	-0.0963988	0.00127836	0.98973091	-0.1626616	0.00870699	0.8638636
Q60931	Vdac3	-0.1922074	0.02104783	0.68135219	-0.0741041	0.00272404	0.94750617	-0.1271436	0.01056156	0.80997254
P20152	Vim	-0.1136413	0.02672428	0.53055201	-0.0606559	0.01343934	0.56007653	-0.2144688	0.06186463	0.44482085
Q9CRC0	Vkorc1	-0.2537059	0.00679933	0.99074922	-0.1406572	0.00376613	0.99006295	-0.1915676	0.02023087	0.87337258
P29788	Vtn	-0.923486	0.14208604	0.76467732	-0.2890036	0.05944071	0.64519188	-0.9656984	0.15713261	0.74394477
Q8CC88	Vwa8 Kiaa05	-0.552074	0.16423477	0.48497048	-0.6936782	0.09218045	0.80178097	-0.5044813	0.15847271	0.52963325
Q00519	Xdh	-0.3139103	0.05599509	0.72367807	-0.1342869	0.00939886	0.9358194	-0.2215027	0.03057224	0.83998277
Q3TNA1	Xylb	-0.1387146	0.00465561	0.98556761	-0.0945442	0.00399665	0.97559273	-0.1151562	0.01257037	0.86587217
Q9CQV8	Ywhab	-0.1201474	0.02068749	0.75407868	-0.2482963	0.07320678	0.48944227	-0.1972748	0.09231158	0.29337689
P62259	Ywhae	-0.1996983	0.01875279	0.87635331	-0.1313813	0.00566988	0.97992448	-0.173143	0.02051493	0.79827666
P61982	Ywhag	-0.2935141	0.0613806	0.65582797	-0.1208855	0.00537582	0.98060738	-0.2110431	0.02077967	0.90363479
P68254	Ywhaq	-0.4553935	0.09379154	0.70215676	-0.3433579	0.1552951	0.61970122	-0.3243809	0.12347975	0.46312738
P63101	Ywhaz	-0.1728147	0.00658457	0.95428236	-0.1519416	0.03464082	0.39072401	-0.1810176	0.01594155	0.81124704
Q8BGC4	Zadh2	-0.039157	0.01040234	0.56296386	-0.0172716	0.00368748	0.70910092	-0.0294327	0.01298412	0.36344025
Q80W54	Zmpste24 Fa	-0.6237163	0.06069987	0.8903732	-0.1871865	0.01027173	0.95954861	-0.5694565	0.14321048	0.54879036
Q91V76		-0.7814678	0.06989598	0.90579862	-0.3844731	0.08392388	0.63622584	-0.2601796	0.08463799	0.4405504
P01837		-0.210318	0.03744277	0.53886569	-0.2044817	0.04817887	0.40927079	-0.4412435	0.09919988	0.43212747
Q9CQE8		-0.2411243	0.00666816	0.99090625	-0.1500165	0.00403063	0.99141178	-0.2164452	0.02279776	0.88251285
Q9DCS2		-0.2444187	0.03098723	0.87362426	-0.133028	0.00416118	0.99513146	-0.2084728	0.03239366	0.77535244
P03987		-0.1211277	0.02171607	0.75676063	-0.086193	0.0175602	0.66752245	-0.2170671	0.10481688	0.28051422
P01864		-0.0313221	0.00963495	0.44841078	-0.1035409	0.09602071	0.09560073	-0.7065629	0.18701476	0.670962
P18525		-0.1047041	0.02929843	0.61485524	-0.1335821	0.04101701	0.77951575	-0.1055851	0.02992167	0.75686611
P04945		-0.0697284	0.03103734	0.45687559	-0.0254977	0.01545878	0.47557183	-0.0174581	0.00963208	0.31940615
Q3UNZ8		-0.1371918	0.02627406	0.81962899	-0.0816107	0.01102672	0.9820716	-0.0521053	0.04735404	0.37708974

Table S4: Turnover rate constants and summary statistics for all proteins detected by MS in each group.

For each identified protein, the slope of the natural log-transformed regression between percent newly synthesized peptide vs. time gives the turnover rate. The proportion of each peptide that is newly synthesized is back calculated in Topograph (1) based on the distribution and intensity of single-labeled and multiple-labeled peaks (representing copies of the peptide which have incorporated one or more heavy leucines) compared with the intensity of the unlabeled peptide peak. In contrast to previous metabolic labeling analysis tools, Topograph significantly improves half-life estimates by correcting to label enrichment in the amino acid precursor pool (1). The standard error (SE) and the coefficient of determination (r^2) for each slope estimate are also reported.

		OCL			OCR			ORP		
UniProt ID	geneid	slope	SE	r2	slope	SE	r2	slope	SE	r2
Q61838	A2m Pzp	-0.5749124	0.0751691	0.76469297	-0.6061351	0.0687	0.7955931	-0.5831656	0.07873711	0.72315997
Q99PG0	Aadac Aada	-0.4872762	0.03028678	0.73152287	-0.3032939	0.02404043	0.62133534	-0.3622952	0.02350373	0.70171538
Q8BGQ7	Aars	-0.2883768	0.03846234	0.87541773	-0.2478104	0.0214917	0.94998315	-0.295741	0.05091205	0.82819079
Q99K67	Aass Lorsdh	-0.1661867	0.01370729	0.64472198	-0.089572	0.00350739	0.87052718	-0.1735976	0.01481502	0.64370109
P61922	Abat Gabat	-0.2693754	0.03302914	0.73485132	-0.1366174	0.00765514	0.91654609	-0.3253081	0.04318219	0.70279337
Q9QY30	Abcb11 Bsep	-0.6121939	0.05821366	0.67602555	-0.4553087	0.05364763	0.59026321	-0.5081764	0.06016814	0.59279965
Q61102	Abcb7 Abc7	-0.1725987	0.00508585	0.98799027	-0.1176229	0.00487744	0.96195631	-0.1634245	0.00892615	0.95173221
Q9CXJ4	Abcb8	-0.270598	0.07857611	0.51880062	-0.1074377	0.00937327	0.90370111	-0.2402561	0.07826964	0.43983895
Q8VI47	Abcc2	-0.3359411	0.17761855	0.41706273	-0.4148803	0.11696852	0.67708572	NA	NA	NA
P55096	Abcd3 Pmp7(	-0.4618336	0.02274017	0.75203374	-0.3673662	0.01948942	0.70875986	-0.4471696	0.02062471	0.7630166
Q99LR1	Abhd12	-0.1230033	0.03470346	0.47294801	-0.1333388	0.07672136	0.15880276	-0.17561	0.11107813	0.12192687
Q8VCR7	Abhd14b Cib	-0.1618352	0.00724415	0.95594552	-0.1143853	0.00458549	0.96435526	-0.1620353	0.00668116	0.96711536
Q8R2Y0	Abhd6	-0.2075714	0.07093711	0.68158568	-0.2683693	0.06025151	0.76779696	-0.2801801	0.04536655	0.92708173
Q921H8	Acaa1a Acaa	-0.7916186	0.05514035	0.89570074	-0.577363	0.05358109	0.8287077	-0.6028117	0.05001661	0.84818073
Q8VCH0	Acaa1b Acaa	-0.7485218	0.06273206	0.75181351	-0.5585403	0.05443328	0.66516783	-0.71484	0.05589425	0.76947947
Q8BWT1	Acaa2	-0.132909	0.00217382	0.95527936	-0.0911578	0.00167118	0.93701545	-0.1629626	0.01015717	0.57792001
Q5SWU9	Acaca Acac G	-0.7143975	0.06193129	0.71515135	-0.4986051	0.0358963	0.77194195	-0.6882355	0.05052825	0.77133485
E9Q4Z2	Acacb	-0.2351031	0.08001779	0.74210504	-0.375108	0.1422513	0.49833181	-0.5482429	0.25175751	0.48676968
Q80XL6	Acad11	-0.6164081	0.04208758	0.80791014	-0.4167414	0.02798213	0.81604472	-0.6404526	0.03516311	0.85337289
Q8JZN5	Acad9	-0.1305595	0.01325426	0.82910379	-0.1775419	0.03734913	0.45560653	-0.1436783	0.01396991	0.86861339
P51174	Acadl	-0.2084955	0.05707213	0.34803849	-0.1142216	0.03759827	0.24141606	-0.2077794	0.05477982	0.34761778
P45952	Acadm	-0.3466648	0.11869215	0.41550429	-0.1542398	0.01729803	0.80711827	-0.3777483	0.10391747	0.48555519
Q07417	Acads	-0.1495825	0.00421522	0.98206315	-0.0944892	0.00401912	0.95342548	-0.1456794	0.00357874	0.98338334
Q9DBL1	Acadsb	-0.3184175	0.02592738	0.93202581	-0.2309247	0.01122755	0.96796559	-0.3744686	0.07043964	0.70194922
P50544	Acadvl Vlcad	-0.3644422	0.025119	0.64471703	-0.2293718	0.01860936	0.5294867	-0.3517488	0.02814224	0.58461817
Q8QZT1	Acat1	-0.1488129	0.00927106	0.70270976	-0.0928294	0.00203235	0.94560992	-0.1560203	0.01231578	0.58044964
Q8CAY6	Acat2	-0.1758546	0.00870549	0.94227089	-0.1305427	0.00430187	0.96946906	-0.2370958	0.03046525	0.69166573
Q91V92	Acly	-0.479602	0.04360749	0.63676455	-0.3636209	0.04185487	0.49177537	-0.4174212	0.04569405	0.5438272
P28271	Aco1 Ireb1 Ir	-0.3051628	0.05662791	0.69077337	-0.159447	0.00581515	0.98171882	-0.3209604	0.05499239	0.70872273
Q99KI0	Aco2	-0.231299	0.02979269	0.43277189	-0.1557509	0.03239522	0.19737211	-0.2087996	0.02628896	0.42889389
Q9QYR7	Acot3 Pte1a	-0.377408	0.05396819	0.63591077	-0.3287412	0.05300055	0.51659849	-0.3825742	0.04500548	0.72072731
Q8BWN8	Acot4 Pte1b	-0.1533515	0.020957	0.69952278	-0.2587617	0.07094244	0.33009408	-0.2759814	0.06999282	0.39313048
Q9R0H0	Acox1 Acox F	-0.7791437	0.0305739	0.72608312	-0.5781994	0.02083849	0.74609434	-0.7635472	0.02804459	0.73811695
Q9QXD1	Acox2	-0.3608598	0.07064438	0.59177061	-0.1726177	0.05071051	0.35557317	-0.3044662	0.06542665	0.48494614
Q8BP40	Acp6 Acpl1 L	-0.1918223	0.02608624	0.81838095	-0.1355325	0.01573132	0.84131674	-0.2584721	0.06321778	0.56253459
Q8VCW8	Acsf2	-0.2095436	0.02145384	0.68930162	-0.1307748	0.02174938	0.43478247	-0.2133586	0.03384206	0.47460933
P41216	Acsl1 Acsl2 F	-0.4075706	0.0301391	0.62870155	-0.2527635	0.01885282	0.60167763	-0.3304264	0.02486326	0.60982988
Q8JZR0	Acsl5 Facl5	-0.4939952	0.02425413	0.72165867	-0.3176883	0.01450892	0.72265661	-0.5059722	0.02034656	0.7873702
Q91VA0	Acsm1 Bucs1	-0.1703213	0.03001085	0.3736176	-0.1977281	0.04603385	0.26565239	-0.1603007	0.02707004	0.3727843
P57780	Actn4	-0.4207527	0.03372894	0.70218478	-0.3193622	0.02803926	0.64308378	-0.4214543	0.03427944	0.66837463
P61161	Actr2 Arp2	-0.1568723	0.00832862	0.93171717	-0.1065212	0.01023484	0.78881528	-0.31803	0.05682255	0.49467241
Q99JY9	Actr3 Arp3	-0.2402145	0.03202441	0.52454079	-0.1186683	0.00802488	0.78751896	-0.2648348	0.03197895	0.55495801
Q60936	Adck3 Cabc1	-0.5759418	0.12995586	0.8674975	-0.5026049	0.10848689	0.84291191	-0.3855786	0.25549803	0.31294705
P00329	Adh1 Adh-1	-0.1749104	0.0212243	0.518771	-0.1293786	0.01689027	0.45956482	-0.1791582	0.00468213	0.95312969
P28474	Adh5 Adh-2 /	-0.2434289	0.03595148	0.54034908	-0.1298942	0.00396378	0.96322519	-0.3398048	0.04221588	0.60670442
P55264	Adk	-0.3606055	0.06890495	0.64612812	-0.2393601	0.04818428	0.48694708	-0.1631328	0.01891597	0.77981583
Q8VDL4	Adpgk	-0.6206849	0.12539691	0.75384727	-0.7093473	0.14527744	0.85632617	-0.5221408	0.16517373	0.71414153
Q9ESW4	Agk Mulk	NA	NA	NA	NA	NA	NA	NA	NA	NA
F8VPN4	Agl	-0.0615703	0.0028221	0.89473458	-0.0591823	0.00439081	0.80861253	-0.056594	0.00212323	0.91973835
A2AS89	Agmat	-0.1412357	0.00345984	0.97713134	-0.0930038	0.00317656	0.94802089	-0.1290829	0.00527583	0.93590011
O35083	Agpat1	-0.1936329	0.03536857	0.88225782	-0.2156384	0.02327213	0.91476474	-0.2026223	0.04000082	0.83691484
Q8K3K7	Agpat2	-0.9362653	0.07787943	0.92333645	-0.5910584	0.10956013	0.72571453	-0.7563148	0.1017809	0.76459828
O35423	Agxt Agxt1	-0.1725252	0.01407004	0.83830843	-0.1064515	0.01013384	0.84656187	-0.2214065	0.04035452	0.53655893
P50247	Ahcy	-0.2825792	0.02250703	0.63655729	-0.1859779	0.00506507	0.93352677	-0.2911597	0.0219499	0.66159475
Q8BK64	Ahsa1	-0.6610936	0.08742944	0.82652837	-0.5365855	0.0808832	0.75866644	-0.5506607	0.11589585	0.65293086
P29699	Ahsg Fetua	-0.9159642	0.10741584	0.76772273	-0.5276398	0.08000766	0.66408246	-0.5639039	0.11162678	0.51534312
O70200	Aif1 Iba1	NA	NA	NA	NA	NA	NA	NA	NA	NA

Q9Z0X1	Aifm1 Aif Pdc	-0.1415732	0.0036545	0.91523044	-0.1066855	0.00889259	0.46439679	-0.1508927	0.01348193	0.4740143
Q9R0Y5	Ak1	-0.208703	0.08143296	0.39643992	-0.1379185	0.03684826	0.53862358	-0.146062	0.14696066	0.08240099
Q9WTP6	Ak2	-0.173611	0.08327587	0.20360774	-0.1681202	0.07728475	0.17063566	-0.3103648	0.10334891	0.3466182
Q9WTP7	Ak3 Ak3l Ak3	-0.3640368	0.04384294	0.63283602	-0.2065373	0.03100285	0.50215347	-0.4161091	0.06171262	0.53196599
Q9JII6	Akr1a1 Akr1a	-0.2391856	0.04717762	0.42343026	-0.1391424	0.03051124	0.33117747	-0.144734	0.01028301	0.83551765
Q91WT7	Akr1c14 mCC	-0.523257	0.10505931	0.67396832	-0.2813431	0.06167372	0.59781685	-0.5116523	0.08054225	0.75635112
P70694	Akr1c6 Hsd17	-0.3078556	0.03112225	0.5440595	-0.200202	0.01895807	0.55339193	-0.2928099	0.02371833	0.6339533
Q8VCX1	Akr1d1	-0.3205381	0.05155182	0.73414778	-0.2569376	0.06391153	0.51864542	-0.288658	0.03676451	0.76440432
Q8CG76	Akr7a2 Afar A	-0.1344081	0.02180593	0.730732	-0.0957986	0.01615544	0.73008086	-0.151117	0.02269896	0.75995132
P10518	Alad Lv	-0.2213399	0.00896397	0.98386324	-0.1856408	0.00788752	0.98226774	-0.2265116	0.01277973	0.96616952
P07724	Alb Alb-1 Alb	-0.3823272	0.01519963	0.74725701	-0.1959687	0.0024757	0.96222254	-0.3425444	0.01213468	0.77076016
Q61490	Alcam	NA	NA	NA	NA	NA	NA	-0.7269957	0.16335928	0.83196846
P24549	Aldh1a1 Ahd	-0.2023201	0.01653132	0.84250473	-0.1909021	0.03250801	0.51869451	-0.2641961	0.03731172	0.6416572
O35945	Aldh1a7 Aldh	-0.4734824	0.07598046	0.57248112	-0.2103158	0.02992826	0.58522644	-0.3430424	0.065938	0.47429289
Q9CZS1	Aldh1b1 Aldh	-0.1650107	0.05211198	0.23303061	-0.1008638	0.02409771	0.28948391	-0.1505177	0.02711177	0.44143583
Q8R0Y6	Aldh1l1 Fthfd	-0.3430017	0.01619256	0.68640304	-0.1867724	0.0079125	0.7042407	-0.315999	0.01385212	0.69908744
P47738	Aldh2 Ahd-1	-0.5447847	0.02546207	0.81198541	-0.2396029	0.00978006	0.8242258	-0.5794157	0.02221282	0.85434711
P47740	Aldh3a2 Ahd	-0.3723423	0.02935614	0.71539667	-0.2405259	0.02091037	0.6413206	-0.3503829	0.02458463	0.74643797
Q8CHT0	Aldh4a1	-0.1287109	0.00590832	0.92586424	-0.1148244	0.02446038	0.3337016	-0.1327258	0.00527067	0.93927113
Q8BWF0	Aldh5a1	-0.1416429	0.01023537	0.97455551	-0.0933097	0.01116242	0.86399171	-0.1139791	0.01000782	0.9284228
Q9EQ20	Aldh6a1	-0.1603828	0.00790692	0.88588291	-0.1248747	0.00716118	0.83981214	-0.1755425	0.00676631	0.92572935
Q9DBF1	Aldh7a1 Ald7	-0.1111172	0.00778957	0.82221256	-0.0904735	0.0071222	0.77817067	-0.1110881	0.00714981	0.84287984
Q8BH00	Aldh8a1 Rald	-0.3058039	0.03183195	0.61002312	-0.171178	0.0141793	0.66933444	-0.3201051	0.03610154	0.53989876
Q9JLJ2	Aldh9a1	-0.2786377	0.0331165	0.68869512	-0.1709013	0.02369135	0.55336651	-0.2219498	0.02267451	0.72689009
P05064	Aldoa Aldo1	-0.2892758	0.0485149	0.39700386	-0.3035317	0.03942016	0.50549342	-0.2836141	0.04412597	0.45242157
Q91Y97	Aldob Aldo2	-0.5159432	0.02398807	0.76135933	-0.2540159	0.00772626	0.86965917	-0.5339631	0.01982589	0.82487378
O09174	Amacr Macr1	-0.4286431	0.10208708	0.54029908	-0.2709448	0.06880856	0.47700631	-0.3401675	0.08208409	0.48825622
Q9DBA8	Amdhd1	-0.1732202	0.00338173	0.99205971	-0.154286	0.00808706	0.93332905	-0.2216013	0.03480301	0.60927262
Q8CFA2	Amt	NA	NA	NA	NA	NA	NA	-0.1833196	0.03270209	0.94016344
P00688	Amy2 Amy2a	-0.295672	0.09366214	0.29339734	-0.2197763	0.04993989	0.42689849	-0.2615162	0.08682528	0.29196795
Q02357	Ank1 Ank-1	-0.0326203	0.0070306	0.70518289	-0.0445526	0.02213851	0.25233389	-0.0277376	0.00482067	0.7506085
Q6P9J9	Ano6 Tmem1	-0.7186071	0.16661929	0.65035989	-0.1647941	0.10220505	0.22412402	-0.3277434	0.15717866	0.35211721
P97449	Anpep Lap-1	-0.7708473	0.06281191	0.80278196	-0.6833878	0.05927657	0.75129055	-0.803806	0.06619026	0.78663663
P10107	Anxa1 Anx1	-0.3380261	0.07098072	0.44750046	-0.2414217	0.06063643	0.32449088	-0.2873385	0.06049282	0.41351265
P07356	Anxa2 Anx2	-0.1335624	0.0341704	0.31645977	-0.0971323	0.01238002	0.61216423	-0.1014145	0.01386399	0.59119897
P97429	Anxa4 Anx4	-0.3343062	0.08064288	0.4621529	-0.2890971	0.06233158	0.49438687	-0.3717695	0.06959075	0.56469583
P48036	Anxa5 Anx5	-0.1548019	0.00642043	0.97321424	-0.1147877	0.00825634	0.91916034	-0.1562848	0.00594269	0.97463434
P14824	Anxa6 Anx6	-0.1535628	0.011381	0.78793276	-0.1405786	0.02193273	0.43666255	-0.1540777	0.01187892	0.76389253
O70423	Aoc3 Vap1	-0.159983	0.16564902	0.31804851	-0.2493295	0.07497174	0.64829855	-0.1273813	0.04520634	0.79878983
O35643	Ap1b1 Adtb1	-0.2111153	0.00785918	0.9917535	-0.1663586	0.00446115	0.9957038	-0.1842739	0.01195626	0.98752806
P17426	Ap2a1 Adtaa	-0.1833344	0.01073963	0.97654261	-0.1587371	0.01234033	0.95388097	-0.2042632	0.01243381	0.97471834
P17427	Ap2a2 Adtab	-0.1784543	0.00604417	0.97429382	-0.1393148	0.00397195	0.97774658	-0.1795734	0.00581731	0.97443456
Q9DBG3	Ap2b1 Clapb	NA	NA	NA	NA	NA	NA	-0.1986434	0.00726992	0.99202766
P12246	Apcs Ptx2 Sa	-0.3496334	0.08660925	0.42553657	-0.2221802	0.05685633	0.37919804	-0.2773678	0.08725167	0.31476251
P28352	Apex1 Ape A	-0.2676489	0.07698609	0.50179893	-0.0943995	0.01235952	0.80645859	-0.0972406	0.02170299	0.60695433
Q9D7N9	Apmap	-0.4052897	0.045506	0.86859692	-0.2919148	0.01030574	0.98405555	-0.3332081	0.01716384	0.9691421
Q00623	Apoa1	-0.1427362	0.03271655	0.63375028	-0.1190999	0.01922548	0.77722313	-0.1443571	0.03971345	0.52405499
P09813	Apoa2	-0.6885937	0.16424479	0.57484281	-0.4309311	0.09928661	0.57366437	-0.3672477	0.11129053	0.37693285
E9Q414	Apob	-0.624869	0.29923112	0.38384525	-0.1923756	0.02501652	0.93664389	-0.3289388	0.13195537	0.34116868
P33622	Apoc3	-0.9076977	0.19628356	0.64055996	-0.8809612	0.06966441	0.92481894	-0.8457384	0.14718196	0.76754418
P08226	Apoe	-0.9867495	0.1301244	0.82734752	-0.1906342	0.01178359	0.94922485	-0.6997828	0.11783206	0.74613665
Q01339	Apoh B2gp1	-0.9777557	0.11768342	0.86254944	-0.3516353	0.05084148	0.78630832	-0.7214663	0.19534631	0.63031812
Q78IK4	Apool Fam12	-0.2226264	0.02549697	0.8265373	-0.1912855	0.02146982	0.85927583	-0.2268123	0.02088698	0.88714894
Q02013	Aqp1	-0.2507358	0.02374546	0.90283336	-0.1378717	0.01223578	0.90068515	-0.2184906	0.01936283	0.90736065
Q5XJY5	Arcn1 Copd	NA	NA	NA	NA	NA	NA	-0.6334239	0.14119839	0.9096033
P62331	Arf6	-0.1194919	0.00646996	0.94200398	-0.0872553	0.00696701	0.85780864	-0.1244575	0.00549164	0.95713872
A2A5R2	Arfgef2 Arfge	-0.4761955	0.16292327	0.48697136	-0.1659056	0.11738005	0.15369746	-0.0026702	0.001494	0.26196199
Q61176	Arg1	-0.1911219	0.01026235	0.69529209	-0.119039	0.0015717	0.97056041	-0.1998218	0.01217058	0.62899421
Q99PT1	Arhgdia C872	-0.1256197	0.00860352	0.9467112	-0.1168866	0.00768435	0.94294423	-0.1344612	0.008251	0.95333331
Q61599	Arhgdib Gdid	-0.4004659	0.06065175	0.68551376	-0.3099352	0.06166044	0.48340732	-0.5548701	0.07803452	0.72685548
Q8R5J9	Arl6ip5 Aip5	-0.5046361	0.08532891	0.74454782	-0.4228632	0.08094208	0.73185307	-0.2653234	0.03856961	0.85539136
Q9WV32	Arpc1b	-0.1847817	0.00936528	0.97252009	-0.120379	0.00888204	0.92918068	-0.1807249	0.00856015	0.97166101
Q9CVB6	Arpc2	-0.2177912	0.06031353	0.40697666	-0.130867	0.01117558	0.87830357	-0.1763767	0.06985173	0.27274921
Q9CPW4	Arpc5	-0.3082027	0.05914622	0.69351061	-0.1417533	0.00598924	0.97561717	-0.1978585	0.00705399	0.98374502
Q9WV54	Asah1 Asah	-0.1296414	0.00960935	0.94300857	-0.097319	0.00480151	0.97623629	-0.0791808	0.01646617	0.65834895
P34927	Asgr1 Asgr-1	-0.4661969	0.05987686	0.80164153	-0.4726233	0.09296632	0.61763784	-0.3577722	0.09344819	0.49423233

P24721	Asgr2 Asgr-2	NA	NA	NA	NA	NA	NA	-0.407561	0.07299518	0.73917771
Q91YI0	Asl	-0.1895992	0.01516626	0.78818302	-0.1578784	0.01804928	0.54842364	-0.2320093	0.02733011	0.57622233
Q8BSY0	Asph	-0.3666894	0.05895157	0.60747758	-0.2354012	0.02903758	0.69383394	-0.2887674	0.03955125	0.66378599
P16460	Ass1 Ass	-0.2998399	0.02148933	0.55515672	-0.1658102	0.0102114	0.59162138	-0.3208248	0.02008669	0.60724115
Q925I1	Atad3 Atad3a	-0.1391921	0.01797377	0.85708627	-0.1191171	0.01015046	0.93865594	-0.2707327	0.03088477	0.9389059
Q6PA06	Atl2 Arl6ip2	-0.8411443	0.14096274	0.7479349	-0.8355921	0.10569085	0.81700514	-0.8257	0.12442554	0.80013731
Q91YH5	Atl3	-0.5140843	0.06125809	0.74583658	-0.4461041	0.04502421	0.77807778	-0.5243691	0.05039669	0.80038522
O08997	Atox1	-0.2461317	0.01097732	0.99015242	-0.0654432	0.00742879	0.92823443	-0.282811	0.01190077	0.99122394
Q9QZW0	Atp11c	-0.5209057	0.10313556	0.69870828	-0.4027122	0.0857548	0.6116855	-0.6556913	0.06809613	0.87702884
Q8VDN2	Atp1a1	-0.3179139	0.03269347	0.65411744	-0.2412274	0.02845385	0.56206865	-0.2234341	0.01959572	0.71430133
P14094	Atp1b1 Atp4	-0.4323531	0.08819062	0.77444344	-0.6597492	0.0892665	0.8198838	-0.6767914	0.12225927	0.81404759
Q8R429	Atp2a1	-0.4868652	0.20111255	0.74556521	-0.3224234	0.04654329	0.87270114	-0.266752	0.06564504	0.8919646
O55143	Atp2a2	-0.5200638	0.05876338	0.78857262	-0.4664526	0.04551713	0.82033829	-0.4724016	0.06122967	0.72129633
Q03265	Atp5a1	-0.1385778	0.00751977	0.62358228	-0.0953721	0.00155023	0.9408379	-0.1458094	0.00750372	0.6318535
P56480	Atp5b	-0.1307113	0.00185011	0.95157759	-0.0982679	0.00138196	0.94293492	-0.135824	0.00516238	0.71865675
Q91VR2	Atp5c1	-0.1146905	0.00217265	0.97720576	-0.0842507	0.00224871	0.94799833	-0.1126128	0.00319534	0.94520782
Q9D3D9	Atp5d	-0.1224861	0.00473999	0.96530578	-0.0903107	0.00387207	0.95271384	-0.1130461	0.00409801	0.96819202
Q9CQQ7	Atp5f1	-0.1629426	0.02750771	0.48008004	-0.1569158	0.03469006	0.30329943	-0.1435962	0.02517737	0.44239372
Q9DCX2	Atp5h	-0.1267202	0.01333275	0.50654469	-0.0824957	0.00180679	0.95291884	-0.1200174	0.01069198	0.57013664
Q06185	Atp5i Atp5k	-0.1174394	0.00427104	0.97927645	-0.0913684	0.00635698	0.92396504	-0.1139879	0.00449259	0.97722996
P97450	Atp5j	-0.1224487	0.00310935	0.9841355	-0.0840304	0.00349709	0.95217495	-0.1171824	0.00310243	0.9814262
P56135	Atp5j2	-0.1196102	0.00611324	0.96960631	-0.0829561	0.00523267	0.94723605	-0.1087788	0.00409401	0.98191877
Q9CPQ8	Atp5l	-0.1194613	0.00472313	0.9815874	-0.0885275	0.00503239	0.95671829	-0.110086	0.00388541	0.98406413
Q9DB20	Atp5o D12W	-0.2564495	0.05342273	0.27096272	-0.2241092	0.04636294	0.25025949	-0.306473	0.05647645	0.3185328
P50516	Atp6v1a Atp6	-0.5458726	0.08376586	0.77968186	-0.1817271	0.06513522	0.35732915	-0.1486454	0.07845816	0.21636906
P62814	Atp6v1b2 Atp	-0.9405004	0.09792509	0.86822572	-0.5916184	0.09559206	0.76144851	-0.5768039	0.11462193	0.61280963
P01887	B2m	-0.7689816	0.13286518	0.73624799	-0.4950587	0.18681482	0.77833158	-0.7278878	0.15055948	0.66075732
Q91X34	Baat	-0.2195596	0.05123689	0.53438037	-0.583623	0.12672242	0.7020937	-0.4170151	0.09358716	0.43299601
O54962	Banf1 Baf Bc	-0.1867611	0.02605025	0.91134491	-0.1344035	0.00341482	0.99294926	-0.1538889	0.00622773	0.99349167
Q61335	Bcap31 Bap3	-0.3760606	0.05745119	0.78120844	-0.1997938	0.00700038	0.98310313	-0.5815876	0.08763596	0.77209718
Q03311	Bche	-0.8426646	0.12923251	0.73920901	-0.6509339	0.08170262	0.77907301	-0.6296455	0.09719987	0.7366686
Q6P3A8	Bckdhb	-0.1337187	0.01003889	0.96728896	-0.0925995	0.00911462	0.91167199	-0.1432535	0.01251629	0.9632343
Q80XN0	Bdh1 Bdh	-0.1210992	0.00290749	0.93732401	-0.0883944	0.00212584	0.92857048	-0.1463589	0.0112032	0.5811609
O35490	Bhmt	-0.2693465	0.0147557	0.7638689	-0.1581988	0.00283121	0.96358258	-0.2752589	0.01415879	0.77775347
D3Z6Q9	Bin2	-0.0374346	0.04666919	0.243401	-0.0064223	0.01225124	0.08391517	-0.0094349	0.00727049	0.45711645
Q9CY64	Blvra	-0.0313008	0.04312204	0.1493902	-0.1805354	0.03363899	0.90566955	-0.0466526	0.03660783	0.35122055
Q923D2	Blvrb	-0.2546094	0.08159881	0.28859407	-0.2349539	0.06830338	0.31276242	-0.2296443	0.04914886	0.45642567
Q8R164	Bphl	-0.1356048	0.01534404	0.7802274	-0.0901594	0.00792127	0.83284939	-0.1172867	0.0110143	0.83136869
P18572	Bsg	-0.5885473	0.08519338	0.79908105	-0.238521	0.00490132	0.99412319	-0.4894606	0.0727122	0.77706437
Q64152	Btf3	-0.8502372	0.08465535	0.77670348	-0.6353548	0.06514437	0.7482724	-0.7732933	0.0673326	0.80475645
Q9CQC6	Bzw1	-0.9412262	0.10625567	0.87704885	-0.7491086	0.06175973	0.91310953	-0.8695687	0.13548923	0.78923415
P98086	C1qa	-0.3774914	0.12741222	0.42246408	-0.1493684	0.08179372	0.19237883	-0.2841843	0.17083954	0.17549785
P14106	C1qb	-0.6218864	0.22089215	0.49768103	-0.2923847	0.13295801	0.49165979	-0.1957708	0.13789173	0.18298212
P01027	C3	-0.7798364	0.04161427	0.70070279	-0.3980696	0.02577713	0.61073392	-0.696901	0.03864979	0.65400934
P00920	Ca2 Car2	-0.0297024	0.00263392	0.76072045	-0.0456221	0.01073455	0.28195326	-0.0282136	0.00190778	0.82935435
P16015	Ca3 Car3	-0.1889819	0.01785698	0.5333368	-0.1460724	0.01515721	0.4717453	-0.2079089	0.01731444	0.57863154
P62204	Calm1 Calm C	-0.1852407	0.00552546	0.97909265	-0.1133231	0.00367566	0.97039398	-0.1858015	0.00497069	0.98104223
P14211	Calr	-0.2628067	0.01874331	0.79402139	-0.2017912	0.00529569	0.96095236	-0.2275014	0.01460525	0.81520879
P51437	Camp Cnlp C	-0.8755169	0.23654001	0.69543111	-1.0948458	0.12501115	0.92745063	-0.6724507	0.23567356	0.67054875
P35564	Canx	-0.5794768	0.04915559	0.73153894	-0.4597567	0.03849518	0.70740027	-0.5175814	0.04270216	0.72760427
P40124	Cap1 Cap	-0.413088	0.04279025	0.64186244	-0.2390737	0.02349804	0.64489137	-0.3533309	0.03797769	0.62022906
P24452	Capg Mbh1	-0.5220258	0.11251261	0.53117563	-0.6386482	0.0989476	0.71019132	-0.4243147	0.08355601	0.57578096
P24270	Cat Cas-1 Cas	-0.841108	0.03849108	0.80050664	-0.5850621	0.02806983	0.75892444	-0.8028589	0.02550898	0.88477883
Q91WT9	Cbs	-0.3533379	0.02242262	0.94303461	-0.2743908	0.00716627	0.99054095	-0.3070293	0.01734582	0.93718348
Q71RI9	Ccbl2 Kat3	-0.1531784	0.01003654	0.93949926	-0.0903909	0.00510653	0.93161411	-0.1273121	0.00893157	0.93124989
Q9D024	Ccdc47 Asp4	-0.4238331	0.06177223	0.83950464	-0.2318227	0.01489594	0.96802573	-0.3051936	0.01491471	0.97667462
P80314	Cct2 Cctb	-0.1408093	0.02129397	0.68616095	-0.1233389	0.02104662	0.58863827	-0.1449952	0.02715224	0.60013871
P80318	Cct3 Cctg	-0.236303	0.01645537	0.9626546	-0.178392	0.01272518	0.97518948	-0.2125116	0.01586769	0.96243931
P80316	Cct5 Ccte Kia	-0.520867	0.10304942	0.58666611	-0.5983506	0.07068577	0.75701272	-0.5462955	0.12084703	0.51819946
P80317	Cct6a Cct6 Cc	-0.3319816	0.03899659	0.72856883	-0.377287	0.04491023	0.68139139	-0.3862778	0.05277239	0.69965238
P80313	Cct7 Ccth	-0.3128336	0.06813092	0.46765201	-0.2081757	0.01371394	0.89860713	-0.2395912	0.01653283	0.8974418
P42932	Cct8 Cctq	-0.7733238	0.11563613	0.83247552	-0.3722464	0.08668614	0.58651443	-0.5203177	0.1060278	0.66742684
Q08857	Cd36	-0.7051134	0.06610364	0.82580965	-0.3818082	0.04330741	0.7351644	-0.6831726	0.06868667	0.78559052
Q61735	Cd47	-0.2264961	0.01308449	0.96149475	-0.1506608	0.00977036	0.94439644	-0.1942431	0.01850702	0.90176697
P35762	Cd81 Tapa1	-0.9026206	0.11325169	0.77919949	-0.498087	0.07974293	0.70916758	-0.7564395	0.12357806	0.66352889
P40240	Cd9	-0.6607968	0.31174171	0.47330163	-0.5848927	0.12507509	0.78469995	-0.9485208	0.17374009	0.85634376

P60766	Cdc42	-0.0496125	0.0150684	0.52016378	-0.0903413	0.01035355	0.84468034	-0.0646059	0.01087122	0.73094532
P15116	Cdh2	-0.5109128	0.07743471	0.82868027	-0.4102221	0.14589574	0.72492029	-0.4616064	0.07488609	0.7916506
Q91X79	Cela1 Ela1	-0.5692181	0.19087966	0.44703586	-0.4138055	0.08299009	0.67446354	-0.3598414	0.16581168	0.29979507
P05208	Cela2a Ela-2	-0.3245166	0.10618923	0.45917434	-0.393104	0.18443812	0.33543502	-0.543587	0.25757355	0.42604882
Q9CQ52	Cela3b Ela3 E	-0.5780335	0.16048687	0.54114275	-0.6471958	0.11421205	0.74484193	-0.3071842	0.06338927	0.68100864
Q9CXS4	Cenpv Prr6	-0.0356431	0.00157108	0.95544832	-0.0250502	0.00116917	0.94058063	-0.0348782	0.0013245	0.96789677
Q8VCC2	Ces1 Ces1g	-0.3941463	0.03008147	0.67675662	-0.2167097	0.01190095	0.78465826	-0.3314469	0.02319226	0.69649479
P23953	Ces1c Es1	-0.4717632	0.09976464	0.7884433	-0.2485415	0.01573496	0.98035344	-0.3319662	0.07376553	0.66944989
Q8VCT4	Ces1d Ces1 C	-0.344756	0.02072114	0.66253368	-0.2479008	0.00629397	0.91398308	-0.3246578	0.01713581	0.7025191
Q64176	Ces1e Es22	-0.4116686	0.06270095	0.86029879	-0.5194142	0.07771243	0.83231839	-0.358544	0.06354286	0.86427208
Q63880	Ces3a Es31	-0.5296073	0.04415903	0.85192771	-0.3430354	0.04051101	0.76521332	-0.3822797	0.04781822	0.68787333
Q8VCU1	Ces3b Gm47	-0.202814	0.0150185	0.94311293	-0.1918544	0.0057474	0.988468	-0.1901505	0.01221207	0.94910879
P18760	Cfl1	-0.503977	0.05583155	0.69357027	-0.3071335	0.04480229	0.54020473	-0.5171172	0.04631699	0.76637151
Q9D1L0	Chchd2	-0.7552995	0.08437532	0.90922738	-0.1609584	0.02251014	0.7972849	-0.6377223	0.1124447	0.72829242
Q9CRB9	Chchd3	-0.1419903	0.00449099	0.96617107	-0.0987975	0.00327538	0.95788796	-0.1376198	0.00644382	0.93062871
Q8BJ64	Chdh	-0.1436317	0.00946113	0.88482366	-0.0849967	0.00641837	0.81427272	-0.1349574	0.01099963	0.82923387
O35744	Chi3l3 Ym1	-0.05499	0.01035646	0.43919306	-0.0557279	0.02735294	0.08620527	-0.0880381	0.04764144	0.07688538
Q91WS0	Cisd1 D10Ert	-0.1774914	0.04254755	0.41040878	-0.0980857	0.00259934	0.98004025	-0.1653246	0.00435083	0.98164364
Q9CQB5	Cisd2 Cdgsh2	-0.5706204	0.07285905	0.8598217	-0.8090024	0.11587263	0.87443019	-0.4140067	0.05253778	0.86129817
Q8BMK4	Ckap4	-0.527558	0.12404057	0.44023856	-0.2436481	0.08735571	0.23732479	-0.4319238	0.10313766	0.41228995
P07310	Ckm Ckmm	-0.1130032	0.02860992	0.69027723	-0.1029661	0.02810789	0.65718815	-0.1380703	0.11188276	0.13216367
Q6P8J7	Ckmt2	-0.0381974	0.01225265	0.44748056	-0.0620207	0.07852623	0.04265638	-0.1425497	0.1119788	0.11084022
P70194	Clec4f Clecsf	-0.3354701	0.1642985	0.19694201	-0.3126255	0.09658036	0.38131926	-0.4297938	0.12523954	0.31175184
Q68FD5	Cltc	-0.1920507	0.00770675	0.78607501	-0.1446721	0.00672676	0.71210825	-0.2031817	0.01189938	0.63167989
Q8R1G2	Cmbl	-0.1913259	0.00766254	0.98889683	-0.1950708	0.00859996	0.99037551	-0.2266304	0.00942605	0.98803553
Q8CHQ9	Cml2	-0.9311696	0.07076978	0.93517923	-0.7274479	0.0857722	0.83707686	-0.9502105	0.078404	0.91868911
Q9QXT0	Cnpy2 Msap	-0.1130036	0.01560419	0.85352693	-0.234776	0.10179605	0.43178089	-0.3118602	0.09010312	0.54503151
Q9DBL7	Coasy Ukr1	-0.3283634	0.06600725	0.56568649	-0.158621	0.01066259	0.92478291	-0.392377	0.06013174	0.68040603
Q80X19	Col14a1	NA	NA	NA	NA	NA	NA	-0.1478328	0.02464182	0.87802247
O88587	Comt Comt1	-0.3894006	0.04136319	0.80844105	-0.2983316	0.00889684	0.98424391	-0.3114199	0.01636962	0.9353876
Q8CIE6	Copa	-0.6157722	0.06837988	0.7045868	-0.5665701	0.06799059	0.67130704	-0.6153035	0.07919955	0.60142604
O89079	Cope Cope1	-0.7277664	0.02683751	0.99728762	-0.2053088	0.01959221	0.94819175	-0.2866232	0.2355897	0.22841466
Q9QZE5	Copg1 Copg	-0.5070622	0.12253003	0.58798665	-0.2736745	0.10658779	0.32014256	-0.3797032	0.09962774	0.52770883
O89053	Coro1a Coro	NA	NA	NA	NA	NA	NA	-0.230359	0.03222002	0.85028998
Q9WUM4	Coro1c	-0.2989679	0.07467765	0.41067318	-0.1667662	0.05177172	0.32048467	-0.3713902	0.09045811	0.44527268
Q9CQI6	Cotl1 Clp	-0.1928937	0.01287058	0.95737707	-0.1486221	0.01636131	0.88237128	-0.1711606	0.02986752	0.74909015
P19783	Cox4i1 Cox4	-0.1557846	0.00787404	0.97025504	-0.1061767	0.00674543	0.94651682	-0.1498063	0.00409603	0.99037481
P12787	Cox5a	-0.1859559	0.03146518	0.41615823	-0.1013151	0.00290097	0.95535456	-0.2148613	0.02420644	0.5933336
P19536	Cox5b	-0.4763941	0.13241905	0.51890127	-0.0750042	0.01169919	0.74592402	-0.1592176	0.08058128	0.24547466
P43024	Cox6a1 Cox6	-0.181235	0.01031208	0.89561623	-0.1084659	0.00817242	0.80747298	-0.1933331	0.02486748	0.62029232
P56391	Cox6b1 Cox6	-0.1640777	0.00458728	0.98083331	-0.1081349	0.0033918	0.97225978	-0.2055625	0.03208199	0.60326173
P48771	Cox7a2 Cox7	-0.1721595	0.00602569	0.98551244	-0.1148491	0.00555626	0.97047181	-0.1754872	0.00468095	0.99083524
Q8BT60	Cpne3 Kiaa0	-0.4606401	0.10075885	0.65517805	-0.2567836	0.07081954	0.50281203	-0.3093999	0.09227614	0.46375082
P36552	Cpox Cpo	-0.2415912	0.03785499	0.61965697	-0.1227314	0.00868111	0.87329386	-0.2383598	0.04877584	0.46935222
Q8C196	Cps1	-0.1491658	0.00391704	0.70976695	-0.0893688	0.00089077	0.93506526	-0.1577025	0.00465359	0.6450282
Q6NVF9	Cpsf6	-0.3718038	0.10494943	0.71511091	-0.406243	0.10563667	0.6216758	-0.198729	0.0487512	0.73471244
P97742	Cpt1a Cpt-1 C	-0.4979368	0.03725597	0.781307	-0.2645336	0.02780188	0.60544189	-0.3991308	0.03321392	0.72418314
P52825	Cpt2 Cpt-2	-0.1534722	0.01578995	0.55417623	-0.108825	0.01115626	0.5057197	-0.1573806	0.0194766	0.45250822
Q9DC50	Crot Cot	-0.3209276	0.07694063	0.49149865	-0.3413716	0.11062529	0.34598706	-0.5830157	0.08053025	0.71394871
P47199	Cryz	-0.1442756	0.02703368	0.40410601	-0.1140158	0.02992677	0.23985502	-0.1253929	0.01200025	0.71745025
Q9CZU6	Cs	-0.1625389	0.00565134	0.97066424	-0.1074997	0.0035618	0.96708812	-0.141081	0.00661423	0.93620939
Q8R311	Ctage5 Mea6	-0.2909481	0.03729697	0.88381089	-0.2878773	0.03085356	0.91584008	-0.2847518	0.03454267	0.9066106
Q8VCN5	Cth	-0.2142915	0.00900748	0.96917718	-0.1579087	0.00804205	0.94139882	-0.2089257	0.00514152	0.98803256
P26231	Ctnna1 Catna	-0.3015237	0.0245246	0.81199045	-0.2814397	0.02870628	0.78709598	-0.2781084	0.01707271	0.9014787
P30999	Ctnnd1 Catns	-0.4733499	0.05046924	0.87995826	-0.435262	0.05941595	0.79309995	-0.3624336	0.0386348	0.89796277
Q9CR35	Ctrb1	-0.3231343	0.10919577	0.30451675	-0.2995804	0.09673684	0.29427369	-0.3432679	0.10996763	0.32759513
P16675	Ctsa Ppgb	-0.3351792	0.12199047	0.38616489	-0.5862236	0.09948706	0.72758378	-0.4997374	0.13560819	0.53089041
P10605	Ctsb	-0.5227371	0.08774074	0.51075441	-0.3275163	0.04577832	0.54345367	-0.4287093	0.06793059	0.50525565
P18242	Ctsd	-0.41503	0.07730028	0.52577956	-0.2251785	0.07527255	0.2036247	-0.295265	0.05762921	0.48387632
P49935	Ctsh	-0.4898469	0.12809511	0.54927287	-0.321887	0.06284906	0.7045446	-0.5473192	0.09718	0.60166614
P56395	Cyb5a Cyb5	-0.3645951	0.02971406	0.74696849	-0.2409912	0.0156349	0.80106641	-0.3496406	0.0259435	0.77082677
Q9CQX2	Cyb5b Cyb5m	-0.3101364	0.09627395	0.46374437	-0.1618812	0.00734856	0.97195941	-0.2373779	0.00676772	0.99113802
Q9DCN2	Cyb5r3 Dia1	-0.2742462	0.01482672	0.79172921	-0.1741156	0.00268773	0.97672615	-0.296909	0.01887473	0.71839054
Q61093	Cybb Cgd	-0.2378313	0.06746642	0.34114614	-0.2070433	0.05575136	0.40813509	-0.1767324	0.08468493	0.18648081
Q9D0M3	Cyc1	-0.1205042	0.00283363	0.96582102	-0.0847295	0.00230557	0.94805392	-0.1182625	0.00238727	0.97342432
P62897	Cycs	-0.1583054	0.00610379	0.96416568	-0.0951619	0.004196	0.94662711	-0.1514831	0.00497284	0.97172596

Q7TMB8	Cyfip1 Kiaa0(	-0.3115339	0.13935472	0.41655316	-0.3090436	0.08342951	0.57844072	-0.3169797	0.07882319	0.61790748
P27786	Cyp17a1 Cyp	-0.5224719	0.04760258	0.81140481	-0.4709834	0.04091931	0.88627341	-0.4599059	0.05437439	0.76480661
P00186	Cyp1a2 Cyp1	-0.9794154	0.11345223	0.86131336	-0.9725864	0.08426212	0.91109702	-0.8836572	0.14713867	0.75035019
Q9DBG1	Cyp27a1	-0.7620044	0.0682603	0.80597265	-0.5368301	0.05010868	0.75621828	-0.6598525	0.06706291	0.75157582
P56593	Cyp2a12	-0.8477503	0.04272074	0.78010408	-0.5490631	0.02779506	0.75885801	-0.746805	0.04077718	0.73975167
B2RXZ2	Cyp2a22 mC(	-0.8631623	0.19764832	0.63421251	-0.5627872	0.1288937	0.59456699	-0.7161457	0.19179072	0.60771873
P15392	Cyp2a4 Cyp2	-0.8838675	0.14873866	0.73091765	-0.5757806	0.09470404	0.66049505	-0.6980459	0.0932578	0.70010138
P20852	Cyp2a5 Cyp2	-0.6140094	0.09954544	0.71722577	-0.5523641	0.06710446	0.76339639	-0.4315279	0.09770618	0.50657357
P12790	Cyp2b9 Cyp2	-0.6163384	0.07815994	0.72999236	-0.4148072	0.04895593	0.71228152	-0.3981393	0.05536639	0.68300252
Q64458	Cyp2c29	-0.7451599	0.04912281	0.74684242	-0.587197	0.03118202	0.81219229	-0.7762007	0.05352013	0.72445728
P56654	Cyp2c37	-0.5245162	0.07170763	0.59117869	-0.5007609	0.05195767	0.68356386	-0.5391734	0.06835178	0.62710602
P56657	Cyp2c40	-0.7822465	0.05639649	0.72767532	-0.5392383	0.03532536	0.74680897	-0.790574	0.05689363	0.72565584
Q6XVG2	Cyp2c54	-0.3651614	0.12872351	0.40141776	-0.0321426	0.01066904	0.39331788	-0.0234876	0.00754163	0.42729831
Q5GLZ0	Cyp2c66 mC(	NA	NA	NA	NA	NA	NA	NA	NA	NA
Q569X9	Cyp2c67 Cyp	-0.9098409	0.17432412	0.71234727	-0.8763238	0.07234471	0.93026009	-0.9476326	0.19098517	0.73229886
E9PXC3	Cyp2c69	-0.8857021	0.12220233	0.81404309	-0.6287978	0.07953362	0.8170074	-0.9390397	0.0920353	0.88898567
Q91W64	Cyp2c70 Cyp	-0.6466529	0.08855679	0.58388569	-0.4293258	0.04722424	0.63748615	-0.5793617	0.08234745	0.54695795
P24456	Cyp2d10 Cyp	-0.5876789	0.03625273	0.81919292	-0.4608409	0.02991944	0.78754761	-0.5046909	0.04353559	0.69133974
Q8CIM7	Cyp2d26 Cyp	-0.8586819	0.03572805	0.87436141	-0.572605	0.02965235	0.79188779	-0.7535092	0.03180588	0.85785416
P11714	Cyp2d9 Cyp2	-0.1308312	0.02344257	0.91214378	-0.0350975	0.00898541	0.68549563	-0.0239493	0.00972557	0.85843664
Q05421	Cyp2e1 Cyp2	-0.9047706	0.07992161	0.78548509	-0.5566669	0.0487594	0.77889182	-0.7401939	0.08534754	0.64720801
P33267	Cyp2f2 Cyp2f	-0.276099	0.06051698	0.42640506	-0.2160513	0.05340155	0.37742675	-0.3178038	0.05856137	0.47158316
Q64459	Cyp3a11 Cyp	-0.3122243	0.07272869	0.62622995	-0.2308598	0.06905051	0.4623204	-0.2673547	0.09218935	0.37528868
Q64464	Cyp3a13 Cyp	-0.5265863	0.04499429	0.66178609	-0.4310919	0.03452692	0.68406091	-0.4170825	0.04680035	0.52799743
Q64481	Cyp3a16 Cyp	-0.6405192	0.10509981	0.66156998	-0.4221998	0.0767773	0.6401294	-0.6351541	0.13841237	0.58399866
O09158	Cyp3a25	-0.9460322	0.1178251	0.87749571	-0.6943718	0.06594801	0.90232904	-0.7758973	0.15315771	0.74036747
Q9JMA7	Cyp3a41a Cy	-0.9103534	0.07712813	0.83264989	-0.598125	0.06093723	0.80727759	-0.8709727	0.08942271	0.73049272
Q9EQW4	Cyp3a44 cyp	-0.6549682	0.11471923	0.54695161	-0.4486411	0.05604957	0.66003855	-0.527738	0.11147772	0.45356257
O88833	Cyp4a10 Cyp	-0.5435769	0.11257301	0.56433311	-0.4599694	0.06059831	0.72367078	-0.5778611	0.10517565	0.60148847
O35728	Cyp4a14	-0.5116135	0.07061446	0.58007539	-0.4268123	0.04075733	0.68684147	-0.4111751	0.07024177	0.4676926
Q8K0C4	Cyp51a1 Cyp	-0.5045248	0.11327197	0.41470362	-0.5028592	0.07697855	0.5424098	-0.6057787	0.09537277	0.56548569
Q9D172	D10Jhu81e	NA	NA	NA	NA	NA	NA	NA	NA	NA
Q99LF4	D10Wsu52e	-0.3941066	0.06543108	0.78392082	-0.2013954	0.0265367	0.83964487	-0.2862472	0.02214781	0.9542962
P61804	Dad1	-0.1474696	0.00696437	0.97393431	-0.1065975	0.00258059	0.99186191	-0.1483333	0.00887538	0.95552827
Q8VC30	Dak	-0.215165	0.01455895	0.63973515	-0.1534164	0.00422911	0.9026044	-0.2209338	0.01159738	0.73775952
P31786	Dbi	-0.2798761	0.04850238	0.48753271	-0.1466049	0.00385821	0.97369947	-0.2546511	0.02920777	0.65521438
P53395	Dbt	-0.1895777	0.00798095	0.99296075	-0.1417209	0.00768674	0.97700655	-0.1629805	0.00798359	0.99522387
O54734	Ddost	-0.1321851	0.02546687	0.28987224	-0.1072952	0.01624694	0.36159542	-0.1333723	0.022	0.33486673
Q80WW9	Ddrgk1	-0.9750593	0.12821794	0.8281578	-0.6957253	0.11824874	0.71203162	-0.844398	0.15939436	0.68342046
O35215	Ddt	-0.160203	0.00409714	0.97140861	-0.1247479	0.00384614	0.95117552	-0.1842173	0.01715591	0.69332724
Q91VR5	Ddx1	-0.2874126	0.00771791	0.9914212	-0.2318059	0.00731988	0.98720298	-0.2533266	0.01443667	0.95949052
Q62167	Ddx3x D1Pas	-0.3950518	0.05786566	0.83815463	-0.3841654	0.04307618	0.88831272	-0.3270153	0.05438771	0.76671291
Q61656	Ddx5 Tnz2	-0.746189	0.1963447	0.67355409	-0.8482216	0.13902336	0.74116848	-0.6379712	0.23103747	0.48799853
Q9CQ62	Decr1	-0.1337176	0.00588093	0.91829391	-0.0945994	0.00327755	0.93701235	-0.1763725	0.02153656	0.59316156
Q9WV68	Decr2 Pdcr	-0.5507348	0.04065319	0.79958628	-0.456623	0.046749	0.66067642	-0.6161413	0.04889043	0.76791728
Q8BNI4	Derl2 Der2 Fl	-0.8607656	0.09679169	0.86825481	-0.534171	0.0759181	0.77955317	-0.6501988	0.08084322	0.83265803
P31001	Des	-0.088914	0.01454909	0.92564714	-0.061612	0.0148371	0.81170945	-0.0991658	0.00834548	0.98603311
O88455	Dhcr7	-0.3655158	0.0738729	0.49476302	-0.1621605	0.0162122	0.7752766	-0.3814274	0.06286146	0.57691886
Q9DBB8	Dhdh	-0.1733821	0.02660559	0.87620728	-0.1394352	0.01247415	0.93982528	-0.1879478	0.02036931	0.94452933
Q99L04	Dhrs1 D14er	-0.2826273	0.02647335	0.78078478	-0.2309972	0.02516861	0.70058752	-0.379419	0.03951784	0.74834223
Q99LB2	Dhrs4 D14Uc	-0.2017754	0.00900117	0.92969509	-0.1371438	0.00485158	0.94780991	-0.2044794	0.0055528	0.97065237
Q8BMF4	Dlat	-0.1366588	0.01788352	0.78492922	-0.0980037	0.00964502	0.79883532	-0.2623947	0.0581136	0.50479178
O08749	Dld	-0.1620767	0.01731174	0.70317253	-0.1124457	0.00802944	0.82017172	-0.1713069	0.01619496	0.74153295
Q9D2G2	Dlst	-0.1192019	0.00725238	0.95746941	-0.0845192	0.00576641	0.93881987	-0.1163531	0.00593358	0.96729749
Q9DBT9	Dmgdh	-0.1659993	0.01062446	0.69132092	-0.099646	0.00247294	0.92745535	-0.1516653	0.003422	0.94197545
Q99M87	Dnaja3 Tid1	-0.25049	0.02809347	0.89830573	-0.2399085	0.01389638	0.95513527	-0.3700631	0.11178398	0.52289039
Q91YW3	Dnajc3 P58ip	-0.3473102	0.04133722	0.88692265	-0.3283085	0.03742284	0.93899803	-0.3919021	0.0359581	0.91524446
Q8K1M6	Dnm1l Drp1	-0.123553	0.02461369	0.59712995	-0.1302276	0.02895828	0.52908789	-0.1019555	0.01989606	0.60702331
P31428	Dpep1 Mbd1	-0.0848038	0.01096777	0.96762987	-0.0674947	0.00973083	0.94130367	NA	NA	NA
P28843	Dpp4 Cd26	-0.1004396	0.00897566	0.77191612	-0.0524942	0.00795656	0.52112066	-0.0798331	0.02882845	0.1608755
Q9EQF5	Dpys	-0.4497137	0.05964093	0.69458913	-0.3496376	0.05561657	0.57677207	-0.4429135	0.06862288	0.60674732
Q9JHU4	Dync1h1 Dhc	-0.1452223	0.0244518	0.64991922	-0.0972246	0.02099263	0.47194232	-0.0704405	0.02303158	0.28044604
P97425	Ear2 Rnase2	-0.4042175	0.19125569	0.4267617	-0.6677506	0.19197233	0.70758613	-0.6706243	0.15687762	0.69551791
Q923L7	Ear6	-0.1747985	0.14185564	0.17824805	-0.853112	0.11919209	0.86493138	-0.4210395	0.17334062	0.37106546
P70245	Ebp Msi	-0.0686336	0.00457866	0.94930221	-0.0611139	0.00282911	0.97087203	-0.0616154	0.00518871	0.91559156
O35459	Ech1	-0.725708	0.03495646	0.87246751	-0.4296522	0.03340254	0.69972906	-0.6647513	0.0399825	0.79793655

Q9D9V3	Echdc1	-0.1927199	0.08159165	0.73611531	-0.0828907	0.00486098	0.99657276	NA	NA	NA
Q3TLP5	Echdc2 D4Ert	-0.1938471	0.00910496	0.98693594	-0.0922474	0.00618934	0.95692187	-0.1412318	0.01002298	0.9430066
Q9D7J9	Echdc3	-0.1596191	0.00746012	0.98071988	-0.086897	0.01082263	0.88960631	-0.1731911	0.01463139	0.94598737
Q8BH95	Echs1	-0.1288433	0.01371559	0.50355493	-0.083575	0.00237045	0.92348055	-0.1732697	0.02080137	0.4272847
P42125	Eci1 Dci	-0.0881094	0.00660968	0.92696866	-0.0806333	0.01285003	0.67451818	-0.1278584	0.01461484	0.81825355
Q9WUR2	Eci2 Peci	-0.2750942	0.03468459	0.52903846	-0.14141	0.015305	0.54247294	-0.2674707	0.02847186	0.56480342
P10126	Eef1a1 Eef1a	-0.2601106	0.01227116	0.81498577	-0.4594761	0.02916205	0.76091977	-0.2701976	0.01891838	0.64966314
O70251	Eef1b Eef1b2	-0.2128834	0.01541399	0.96460091	-0.2917049	0.02987584	0.97945216	-0.1724273	0.06015827	0.67254076
P57776	Eef1d	-0.1705281	0.01341745	0.93084747	-0.2741344	0.06406024	0.60412503	-0.3058305	0.05540162	0.70096453
Q9D8N0	Eef1g	-0.1057468	0.00905786	0.91908074	-0.0890089	0.00982859	0.85418696	-0.0809588	0.00605221	0.9322694
P58252	Eef2	-0.5846867	0.03244414	0.72855869	-0.7110637	0.04270771	0.82451296	-0.5622453	0.02930246	0.7316988
Q9WVK4	Ehd1 Past1	-0.4213775	0.05535372	0.74342334	-0.2626813	0.05008564	0.5340375	-0.445522	0.09575149	0.54602168
Q9QXY6	Ehd3 Ehd2	-0.4383143	0.05411277	0.63941229	-0.1513855	0.01376813	0.73316822	-0.4217611	0.06463806	0.50942371
Q9DBM2	Ehhadh	-0.483324	0.02083757	0.76312072	-0.3547341	0.01961407	0.62411205	-0.488011	0.02161809	0.73471531
Q6ZWX6	Eif2s1 Eif2a	-0.3875106	0.06693955	0.73633386	-0.2125207	0.01372735	0.94481206	-0.4862574	0.06706138	0.80175671
Q9WUK2	Eif4h Wbscr1	-0.8807328	0.14920027	0.77701317	-0.6451339	0.10303894	0.7656295	-0.8843195	0.07681428	0.92336429
P63242	Eif5a	-0.2179322	0.02672637	0.76876216	-0.1786101	0.02181748	0.75286378	-0.2120296	0.0238703	0.78978995
O55135	Eif6 Itgb4bp	-0.2697925	0.0314477	0.92462376	-0.2697003	0.0257786	0.95631556	-0.2291605	0.01236282	0.99421285
Q3UP87	Elane Ela2	-0.0673968	0.02538562	0.701451	-0.2423452	0.17553642	0.27599717	-0.2800417	0.22390518	0.23830299
Q9JLJ4	Elovl2 Ssc2	-1.0271684	0.11658157	0.86611463	-0.8207035	0.07507911	0.89512383	-0.8230178	0.12133196	0.77970463
Q9CRD2	Emc2 Kiaa01	-0.2161301	0.01394932	0.95239274	-0.1583709	0.00639477	0.97768352	-0.1907597	0.01673085	0.90908972
P17182	Eno1 Eno-1	-0.2054651	0.01989471	0.5536174	-0.1562579	0.01380687	0.55668297	-0.2225199	0.02170481	0.53055321
P21550	Eno3 Eno-3	NA	NA	NA	NA	NA	NA	-0.0093288	0.00472415	0.66098559
P16406	Enpep	-0.5551946	0.0879552	0.64426864	-0.4685544	0.06618612	0.71476304	-0.5137493	0.07458354	0.64600731
Q9WUZ9	Entpd5 Cd39	-0.4206734	0.06069188	0.56492532	-0.3433309	0.04450775	0.55350973	-0.394623	0.04860229	0.57363563
Q9D379	Ephx1	-0.3802447	0.03213925	0.65416752	-0.307552	0.03540665	0.45329389	-0.4067996	0.02729351	0.71858144
P34914	Ephx2 Eph2	-0.4298479	0.02585982	0.61495547	-0.2390382	0.01365282	0.60397368	-0.433863	0.02348952	0.64229177
Q8R0W0	Eppk1	-0.1724631	0.01705181	0.99031892	-0.239057	6.30E-05	0.99999993	NA	NA	NA
P49290	Epx Eper	-0.7124237	0.42986133	0.40712239	-0.1427762	0.02885999	0.68992071	-0.1858432	0.01684186	0.92410584
Q9EQH2	Erap1 Appils	-0.609563	0.13837439	0.65992803	-0.5716971	0.09829231	0.73815877	-0.6084604	0.11162908	0.71230265
P57759	Erp29	-0.2098103	0.02415404	0.92633748	-0.220472	0.0200001	0.9604801	-0.1661106	0.02137397	0.89613978
Q9D1Q6	Erp44 Kiaa05	-0.101188	0.01232776	0.65811494	-0.1014864	0.0114117	0.66411619	-0.0940053	0.01030172	0.68103206
Q9R0P3	Esd Es10 Sid4	-0.1523329	0.00387056	0.97055066	-0.111658	0.00270889	0.96919594	-0.1465776	0.00315259	0.97562878
Q8BWY3	Etf1	-0.4342249	0.07121412	0.80510603	-0.2880349	0.01789823	0.97368247	-0.3602669	0.0780249	0.65965095
Q99LC5	Etfa	-0.1531996	0.0114639	0.63421633	-0.1225558	0.01249016	0.44311234	-0.1936746	0.02028512	0.44870227
Q9DCW4	Etfb	-0.1438457	0.00433693	0.98126829	-0.0894476	0.00344776	0.96418715	-0.148048	0.00484237	0.97496704
Q921G7	Etfdh	-0.1578272	0.01282562	0.53998947	-0.0898256	0.00206316	0.92712315	-0.127434	0.00356336	0.90260711
P26040	Ezr Vil2	-0.1749622	0.02247389	0.7911453	-0.168254	0.02869001	0.66921518	-0.1540152	0.03145715	0.63129868
O08914	Faah Faah1	-0.3570825	0.02212948	0.81271785	-0.2754364	0.01453691	0.86715077	-0.3733617	0.02745987	0.76118754
P12710	Fabp1 Fabpl	-0.2619304	0.02802282	0.53153669	-0.2235478	0.02454725	0.4938502	-0.3063644	0.02816265	0.58776085
P04117	Fabp4 Ap2	-0.1002153	0.05668631	0.12954965	-0.1201015	0.03935339	0.24960985	-0.0553992	0.01059961	0.54289493
Q05816	Fabp5 Fabpe	-0.284067	0.07046432	0.57524908	-0.3218294	0.08203915	0.52363059	-0.1776605	0.00977383	0.96214424
P35505	Fah	-0.1956361	0.02165844	0.51125142	-0.126953	0.0020072	0.97632655	-0.2049251	0.01511506	0.68634547
Q9CR98	Fam136a	-0.1506778	0.01459083	0.95521497	-0.1120309	0.01024822	0.96761211	-0.2395745	0.04240679	0.91407987
Q921M7	Fam49b	-0.507017	0.13184266	0.64895048	-0.2701279	0.14793177	0.40007589	-0.1664709	0.03480082	0.76574671
Q8BSE0	Fam82a1 Far	-0.3244841	0.05036273	0.64347441	-0.499838	0.08974549	0.58505749	-0.446684	0.07674014	0.61735354
Q3UJU9	Fam82a2 Far	-0.1757048	0.01805492	0.84781439	-0.1866135	0.01574813	0.87532704	-0.1718543	0.01134827	0.92722283
Q9DCV4	Fam82b	-0.0286283	0.01177819	0.32990484	-0.0628114	0.01334012	0.61293472	-0.0094826	0.00892634	0.0798755
P19096	Fasn	-0.5425011	0.0176606	0.69148579	-0.6323512	0.02303086	0.71061476	-0.5422313	0.01733113	0.69975316
Q9QXD6	Fbp1 Fbp	-0.291778	0.0257655	0.5618673	-0.2017021	0.01519885	0.61554086	-0.3568504	0.03089632	0.56429946
Q920E5	Fdps	-0.2959538	0.04029018	0.69213879	-0.2445879	0.00935537	0.96335523	-0.3065774	0.03378874	0.75998343
P46656	Fdx1	-0.2357127	0.01445434	0.97079558	-0.1918995	0.01437559	0.95192185	-0.1987344	0.02385941	0.90835154
P22315	Fech	-0.0413012	0.01953319	0.28898063	-0.0150065	0.00688921	0.28336046	-0.0202168	0.0119357	0.22293897
Q8K1B8	Fermt3 Kind3	-0.2990913	0.17099708	0.43337623	-0.7419412	0.04131251	0.99383732	-0.3515459	0.18321188	0.55101719
Q8K0E8	Fgb	-0.7376629	0.08900427	0.840862	-0.2085286	0.00798989	0.97986076	-0.5622275	0.11148405	0.66174961
Q8VCM7	Fgg	-0.8740245	0.04543437	0.89590019	-0.3693842	0.0364515	0.70972307	-0.7070789	0.05344988	0.80275385
P97807	Fh Fh1	-0.1458927	0.00709367	0.91360421	-0.0919527	0.00687771	0.77130339	-0.1359994	0.011308	0.78336788
P97447	Fhl1	-0.2017132	0.03894819	0.70916536	-0.3590082	0.05882297	0.74128853	-0.569272	0.09587564	0.74605968
Q9D1M7	Fkbp11	-0.118535	0.01844269	0.66296976	-0.1157421	0.02463695	0.500798	-0.1164125	0.02585139	0.47963859
P45878	Fkbp2 Fkbp1	-0.228191	0.01523711	0.95324734	-0.3290228	0.05530832	0.74677792	-0.1983256	0.00800379	0.97926625
O35465	Fkbp8 Fkbp3	-0.3822383	0.05192966	0.90029879	-0.3332345	0.02504114	0.95677757	-0.3894606	0.05718244	0.88546903
Q8BTM8	Flna Fln Fln1	-0.2142423	0.007569	0.98767239	-0.1713256	0.00815202	0.97354999	-0.2370682	0.01529519	0.95621635
P50285	Fmo1 Fmo-1	-0.5308009	0.02998487	0.78663117	-0.4062121	0.02764692	0.7174953	-0.4522462	0.02491855	0.7835319
P97501	Fmo3	-0.6773073	0.05553684	0.7637799	-0.5513516	0.04106224	0.74719231	-0.6904902	0.05468768	0.7836961
P97872	Fmo5	-0.5110549	0.02402468	0.81752537	-0.4074509	0.02130824	0.77035304	-0.5102255	0.02469632	0.79509531
P11276	Fn1	-0.4651738	0.06119388	0.53611322	-0.2554829	0.03895928	0.45265064	-0.3391294	0.05116982	0.44855257

Q91XD4	Ftcd	-0.2824266	0.03333743	0.66595726	-0.2476655	0.02815307	0.65925273	-0.3928462	0.04569894	0.66040501
P29391	Ftl1 Ftl Ftl-1	-0.2665793	0.04155235	0.53342928	-0.1734022	0.00872363	0.91226191	-0.3180344	0.04014398	0.62912304
P97855	G3bp1 G3bp	-0.9060259	0.07475055	0.92448574	-0.7818598	0.06300145	0.91667299	-0.8756204	0.08728426	0.88560115
P35576	G6pc G6pt	-0.2557974	0.01647334	0.95259119	-0.2314999	0.00716927	0.98956041	-0.2044979	0.0072278	0.98523089
Q8K157	Galm	-0.0476453	0.0113259	0.66288048	-0.0441116	0.00609079	0.83987581	-0.0781947	0.01269798	0.86339267
O35969	Gamt	-0.6235444	0.10967364	0.84344171	-0.5770371	0.13745158	0.85453941	-0.5799559	0.0973882	0.8351512
Q8BHN3	Ganab G2an	-0.4394151	0.06364483	0.5200051	-0.484641	0.05738384	0.61848004	-0.466343	0.04532461	0.66637851
P16858	Gapdh Gapd	-0.23541	0.03047229	0.44644538	-0.1348165	0.01311171	0.54574215	-0.2966741	0.02692811	0.60274131
Q9Z0E6	Gbp2	-0.2850255	0.10582952	0.34128818	-0.0730786	0.0196309	0.51597251	-0.2238718	0.07803641	0.38766131
P21614	Gc	-0.9260825	0.0631181	0.79065203	-0.4161577	0.04112118	0.59747978	-0.7713881	0.05280913	0.75297081
O88986	Gcat Kbl	-0.2283428	0.01190304	0.97097696	-0.1588253	0.02383443	0.78725217	-0.1734586	0.02191232	0.92610498
Q60759	Gcdh	-0.2962766	0.01914333	0.67945979	-0.1710975	0.01238922	0.59466366	-0.3333941	0.02229342	0.66041302
O09172	Gclm Glclr	-0.3266967	0.08870415	0.55219806	-0.4033232	0.06209304	0.75085013	-0.2679357	0.03342519	0.86533069
Q61598	Gdi2 Gdi3	-0.4544652	0.03655609	0.67036	-0.2845869	0.02092031	0.68020808	-0.4604377	0.03568028	0.68663318
Q9QYC7	Ggcx	-0.2854133	0.03308417	0.8416704	-0.3073061	0.20352121	0.20212325	-0.2592636	0.04020774	0.76180893
Q99JY3	Gimap4 Ian1	-0.4166318	0.08325822	0.61014533	-0.4054984	0.1012271	0.43314729	-0.3767501	0.0959902	0.52388542
Q00977	Gjb2 Cxn-26	-0.8921745	0.1450583	0.8631015	-0.4202939	0.26030866	0.5658712	-0.5239371	0.16246407	0.67532986
Q91W43	Gldc	-0.3843554	0.0414597	0.77466038	-0.2035411	0.0272294	0.6217025	-0.4150094	0.0731614	0.65431328
Q9CPU0	Glo1	-0.2179292	0.01170038	0.96926698	-0.1755196	0.00841382	0.96883183	-0.2168577	0.01072967	0.97146156
P26443	Glud1 Glud	-0.1767301	0.00941935	0.77877576	-0.131572	0.00208897	0.97230405	-0.1574564	0.00872811	0.75257098
P15105	Glul Glns	-0.3649538	0.03687789	0.57972314	-0.2602907	0.01980607	0.67806679	-0.3252589	0.02348595	0.72706289
Q91XE0	Glyat	-0.0843174	0.01606177	0.69664773	-0.1131446	0.02227993	0.64814691	-0.0821089	0.01783805	0.61974761
Q5FW57	Gm4952	NA	NA	NA	NA	NA	NA	-0.1784986	0.03574639	0.80604339
P08752	Gnai2 Gnai-2	-0.5775254	0.05619479	0.69661184	-0.3274505	0.04646183	0.51918322	-0.6098247	0.06313701	0.67951396
P62874	Gnb1	-0.6518817	0.11499266	0.61639094	-0.565881	0.10759067	0.6193727	-0.4281168	0.10206906	0.48077301
P62880	Gnb2	-0.2387296	0.01389054	0.88868014	-0.2032883	0.01280279	0.87505424	-0.2277559	0.01629105	0.83011435
P68040	Gnb2l1 Gnb2	-0.1283998	0.01226188	0.5180755	-0.1233591	0.00376926	0.91227327	-0.1084382	0.00309968	0.91753253
Q9QXF8	Gnmt	-0.2606362	0.02263471	0.57252536	-0.1566524	0.01259404	0.58008259	-0.3071769	0.02283398	0.64180969
P05201	Got1	-0.1604924	0.00620385	0.93048288	-0.1152477	0.00492559	0.90420428	-0.1523889	0.00598727	0.92569434
P05202	Got2 Got-2	-0.1238406	0.00329775	0.93564356	-0.0802559	0.00242477	0.90649575	-0.1211075	0.00355859	0.91687829
Q61586	Gpam Gpat1	-0.8818587	0.08146397	0.83000857	-0.8641375	0.07090808	0.84137445	-0.7052495	0.08433024	0.74451484
P13707	Gpd1 Gdc-1 (	-0.2449219	0.01054673	0.95569631	-0.1812854	0.00582304	0.97094856	-0.2705571	0.02808331	0.78780412
Q3ULJ0	Gpd1l Kiaa00	-0.2108953	0.01108449	0.98637583	-0.1497414	0.01949534	0.89393286	-0.2215066	0.02638924	0.93373645
Q64521	Gpd2 Gdm1	-0.1641482	0.00668395	0.90406569	-0.1284256	0.00486091	0.89487452	-0.1558144	0.00829877	0.83630797
P06745	Gpi Gpi1	-0.2007903	0.01006395	0.94537597	-0.1392471	0.00517696	0.96659866	-0.1878247	0.00432901	0.98792949
Q8QZR5	Gpt Gpt1	-0.548756	0.0709804	0.8327987	-0.3537949	0.03030534	0.90684693	-0.5360708	0.08059061	0.78665202
P11352	Gpx1	-0.1933533	0.02895288	0.53994277	-0.1171415	0.00309718	0.97081785	-0.170831	0.00603045	0.95139189
Q91Z53	Grhpr Glxr	-0.0911619	0.01264374	0.81245503	-0.0872872	0.00747692	0.90684512	-0.0983784	0.01573104	0.76521081
P28798	Grn	-0.5316845	0.05215581	0.67079958	-0.2370839	0.02769722	0.5539457	-0.4971547	0.05310195	0.61444655
P30115	Gsta3 Gstyc	-0.1553256	0.00709951	0.96375806	-0.1171424	0.00660428	0.94022965	-0.143327	0.00557338	0.97782149
Q9DCM2	Gstk1	-0.3851906	0.05939601	0.52533729	-0.2736481	0.04127188	0.50553056	-0.3649709	0.05178926	0.55388744
P10649	Gstm1	-0.1666141	0.004321	0.97507881	-0.1243586	0.00463664	0.94483545	-0.1880629	0.03287888	0.4561952
P15626	Gstm2	-0.130739	0.00516141	0.98164055	-0.0929082	0.00399342	0.97478729	-0.1250427	0.00716656	0.95904673
P19639	Gstm3	-0.2135044	0.01953027	0.97551182	-0.2014771	0.00796183	0.99071728	-0.1941422	0.00953358	0.98808655
P19157	Gstp1 Gstpib	-0.1564309	0.00735249	0.94766203	-0.1100925	0.00538315	0.93725584	-0.212661	0.04267286	0.47912104
Q64471	Gstt1	-0.1542425	0.01080821	0.96220308	-0.1117851	0.01007001	0.93193555	-0.1370855	0.01299903	0.9407855
Q9WVL0	Gstz1 Maai	-0.1357211	0.00515236	0.92041152	-0.1005921	0.00240687	0.96146881	-0.1469409	0.00450718	0.93986885
P58710	Gulo	-0.4212664	0.04550947	0.67106819	-0.4007005	0.03873965	0.68586993	-0.4471754	0.04284962	0.70305052
Q80SU7	Gvin1	-0.2060718	0.12810082	0.20558101	-0.0952005	0.08886994	0.09446705	-0.1785485	0.10663179	0.18939445
Q8VCB3	Gys2	-0.2350246	0.02250562	0.5619795	-0.1468756	0.02155509	0.3672392	-0.2920832	0.02734988	0.53532446
P14434	H2-Aa	-0.6211218	0.33899082	0.35878277	-0.2245359	0.04083318	0.90974036	-0.4145931	0.15593518	0.43991424
P01899	H2-D1	-0.9963289	0.11990238	0.85193924	-0.4822902	0.07999917	0.72191932	-0.9999595	0.11761498	0.84756733
P01901	H2-K1 H2-K	-0.7895866	0.17569357	0.62729495	-0.2482515	0.03261944	0.87864132	-0.5449037	0.18615663	0.46144077
Q9QZQ8	H2afy	-0.089552	0.0041253	0.92719932	-0.0553599	0.00280398	0.90064674	-0.1084408	0.03131113	0.23991878
Q8CFX1	H6pd	-0.579938	0.09199869	0.79894418	-0.4525248	0.08423654	0.68943483	-0.6696965	0.07696068	0.85347381
Q78JT3	Haao	-0.2838696	0.04896237	0.46937331	-0.1552872	0.00575899	0.94293667	-0.2474744	0.0277874	0.66475776
Q9QXE0	Hacl1 Hpcl Pl	-0.4715105	0.0478923	0.62563368	-0.3194796	0.04257859	0.4840903	-0.538682	0.04004355	0.74176859
Q61425	Hadh Hadhsc	-0.1496598	0.00352987	0.94830139	-0.103021	0.00234943	0.94449252	-0.1497348	0.00411296	0.92853985
Q8BMS1	Hadha	-0.1563576	0.00523505	0.80001154	-0.1011668	0.00429683	0.68240026	-0.1640354	0.00718982	0.68442739
Q99JY0	Hadhb	-0.1188011	0.00616333	0.88770605	-0.0965956	0.00362345	0.92574966	-0.1583037	0.01809068	0.60022717
Q99KB8	Hagh Glo2	-0.1919244	0.14340253	0.14003454	-0.2742111	0.11725865	0.28089529	-0.234987	0.12035582	0.24108299
P35492	Hal Hsd Huth	-0.5549025	0.08052002	0.64622269	-0.4224514	0.06437449	0.57370363	-0.4122316	0.07272951	0.49329258
Q9WU19	Hao1 Gox1 H	-0.3609587	0.09428045	0.59445042	-0.3096549	0.08441191	0.59923425	-0.421671	0.08216089	0.68701168
P01942	Hba Hba-a1	-0.0347775	0.00414237	0.814998	-0.0319445	0.00932999	0.35824425	-0.0286855	0.00245291	0.85070958
P02088	Hbb-b1	-0.0328749	0.00389693	0.61794905	-0.0336105	0.00745373	0.27726955	-0.026043	0.00326184	0.5704572
Q8VDJ3	Hdlbp	-0.7603962	0.0504398	0.76969871	-0.6774369	0.04347291	0.77623547	-0.6864856	0.05176637	0.72412105

Q9R257	Hebp1 Hbp	-0.9374833	0.10379464	0.8717658	-0.5059753	0.08100627	0.73591923	-0.7475315	0.13062783	0.73183283
O09173	Hgd Aku Hgo	-0.2678044	0.02320424	0.65551007	-0.1469997	0.00596089	0.8850325	-0.2335902	0.01775827	0.70904582
Q99L13	Hibadh	-0.3590525	0.07162242	0.59650118	-0.2046348	0.05836494	0.39283382	-0.4709188	0.10767555	0.56047162
Q8QZS1	Hibch	-0.2006865	0.01808355	0.91800823	-0.1254173	0.0072945	0.95787609	-0.1820012	0.02028844	0.87023267
P70349	Hint1 Hint Pk	-0.1944804	0.00749984	0.9781796	-0.133031	0.00381347	0.98462694	-0.1911347	0.00626586	0.98205808
P43275	Hist1h1a H1a	-0.2790034	0.08870092	0.45189942	-0.2974703	0.1312387	0.26845717	-0.185433	0.11753667	0.16069544
P43276	Hist1h1b H1f	-0.0759279	0.01117984	0.79354671	-0.0670466	0.01193928	0.69254652	-0.0656571	0.00978795	0.77584934
P43274	Hist1h1e H1f	-0.052547	0.003486	0.90087872	-0.0419764	0.00251762	0.90553448	-0.0474252	0.00173952	0.9649484
P68433	Hist1h3a H3a	-0.0315268	0.00627795	0.7160612	-0.020909	0.00792284	0.36724687	-0.0296987	0.0076732	0.55523137
P84228	Hist1h3b H3-	-0.0256494	0.0065768	0.62825063	-0.0158429	0.00684305	0.29194093	-0.0243703	0.00724777	0.58562427
P62806	Hist1h4a; His	-0.0224508	0.00218614	0.68277536	-0.0116234	0.00190941	0.38983975	-0.0202002	0.00229128	0.58560539
P30681	Hmgb2 Hmg2	-0.3113004	0.1424528	0.32320326	-0.7286784	0.146336	0.83218833	-0.7477856	0.1856684	0.61862665
P38060	Hmgcl	-0.1512365	0.00655063	0.94671634	-0.1137326	0.00421078	0.95173058	-0.1420381	0.00951585	0.8778564
P54869	Hmgcs2	-0.225368	0.00364726	0.97100806	-0.1350247	0.00219761	0.96745333	-0.2538398	0.00901394	0.86289896
P14901	Hmox1	-0.7020322	0.11255387	0.64943853	-0.4984922	0.07140627	0.64349539	-0.6429879	0.10177981	0.62447222
O70252	Hmox2	-0.168279	0.04023366	0.81389856	-0.1442194	0.03823132	0.73999133	-0.1651327	0.06550427	0.47585842
O88569	Hnrnpa2b1 H	-0.2631861	0.02915436	0.95321241	-0.2985182	0.05396098	0.85956742	-0.46896	0.09776824	0.76672767
Q99020	Hnrnpab Cbf	-0.343973	0.13254803	0.35946877	-0.1944404	0.06817111	0.38491465	-0.2808084	0.10697169	0.34643829
Q60668	Hnrnpd Auf1	-0.1655225	0.01034817	0.95519894	-0.1003478	0.00544924	0.96035263	-0.1539719	0.00770679	0.96845809
Q9Z2X1	Hnrnpf Hnrpf	-0.034186	0.01226347	0.39304651	-0.0490557	0.0091857	0.67074614	-0.0163377	0.01104555	0.14404935
P61979	Hnrnpk Hnrp	-0.6549087	0.05490898	0.85052976	-0.304635	0.04185922	0.65416551	-0.6525799	0.06361011	0.79583859
Q8R081	Hnrnpl Hnrpl	-0.1269227	0.01363289	0.98859448	-0.3630791	0.24957789	0.34601658	-0.6675595	0.09376568	0.9102116
Q8VEK3	Hnrnpu Hnrp	-0.501999	0.0655471	0.66160638	-0.2478173	0.02630144	0.72307699	-0.3992733	0.04119433	0.7518874
Q9DCU9	Hoga1 Dhdps	-0.1300473	0.0072891	0.9636709	-0.0747023	0.01150934	0.7505686	-0.1107924	0.01197799	0.87699411
Q61646	Hp	NA	NA	NA	NA	NA	NA	-0.921073	0.5833241	0.45387659
P49429	Hpd	-0.7019337	0.03946556	0.78043185	-0.5400708	0.03065854	0.7507935	-0.7150844	0.03823482	0.78464772
Q91X72	Hpx Hpxn	-0.7111413	0.08135521	0.83590128	-0.5671064	0.08523451	0.67825383	-0.8646638	0.09391608	0.83294784
Q9ESB3	Hrg	-0.4352899	0.1350882	0.67496563	-0.2053007	0.05001237	0.7711776	-0.5561151	0.19531621	0.57467512
P52760	Hrsp12 Hrp1	-0.1858273	0.0062672	0.9723503	-0.1680678	0.03394889	0.45803067	-0.1790733	0.00552791	0.97491629
P50172	Hsd11b1 Hsd	-0.4975551	0.04338295	0.72457238	-0.3384442	0.02354041	0.80209762	-0.4687894	0.0370193	0.75870683
O08756	Hsd17b10 Er	-0.1260912	0.00435152	0.93852192	-0.1067748	0.01821412	0.33571282	-0.1873523	0.02375784	0.5217626
Q9EQ06	Hsd17b11 Dh	-0.2864076	0.02326266	0.93234219	-0.2537378	0.01435887	0.97502107	-0.2757545	0.01610757	0.96066596
O70503	Hsd17b12 Kil	-0.3299964	0.03994104	0.57237055	-0.1857638	0.01865323	0.62700158	-0.2940374	0.03529735	0.55785592
Q8VCR2	Hsd17b13 Sc	-0.6531367	0.05711528	0.65789369	-0.4265688	0.03801483	0.59983495	-0.5719137	0.05025343	0.62715086
P51658	Hsd17b2 Edh	-0.6829906	0.08858153	0.83204759	-0.2584817	0.00885285	0.98384301	-0.5209172	0.05898748	0.8571212
P51660	Hsd17b4 Edh	-0.7059059	0.0254433	0.80370552	-0.6334175	0.01856473	0.84717642	-0.6950042	0.02154867	0.83336555
Q9R092	Hsd17b6 Gm	-0.28689	0.07784827	0.36137998	-0.411627	0.0641376	0.58683048	-0.1844214	0.01432439	0.86441123
P50171	Hsd17b8 H2-	-0.1330711	0.00448389	0.98766483	-0.0878693	0.00364381	0.97813356	-0.2370433	0.07953343	0.52614836
P11499	Hsp90ab1 Hs	-0.5713421	0.03622501	0.7475636	-0.6493416	0.04609639	0.71012666	-0.5931452	0.04125694	0.68738993
P08113	Hsp90b1 Grp	-0.3273162	0.01810469	0.72819629	-0.30135	0.01772939	0.6863898	-0.3329817	0.01919686	0.69828562
P20029	Hspa5 Grp78	-0.4379081	0.0263564	0.66350509	-0.454737	0.020378	0.75225213	-0.4118968	0.02264298	0.69096472
P63017	Hspa8 Hsc70	-0.7769475	0.05210851	0.82548223	-0.7457169	0.05642921	0.77055972	-0.6977341	0.05677796	0.76264434
P38647	Hspa9 Grp75	-0.5088588	0.04763366	0.67880336	-0.3111607	0.0341785	0.57604334	-0.3785164	0.04512704	0.53561009
P63038	Hspd1 Hsp60	-0.1366673	0.00267332	0.96671008	-0.09328	0.00259794	0.92138319	-0.156917	0.0096162	0.72897267
Q64433	Hspe1	-0.1421385	0.00317082	0.98771173	-0.0947473	0.00408146	0.94893401	-0.1372593	0.00289065	0.9881668
Q9JKR6	Hyou1 Grp17	-0.3455891	0.03087494	0.63828571	-0.4414091	0.03162645	0.696208	-0.2861912	0.02724149	0.5707709
Q9DB29	Iah1	-0.2179537	0.00542916	0.99260911	-0.1671031	0.00736094	0.9735525	-0.2294439	0.00974914	0.97879433
P03975	Iap	-0.1011195	0.07812952	0.21825084	-0.0784954	0.02290809	0.49454853	-0.1339964	0.01100595	0.96109666
O88844	Idh1	-0.1465686	0.00627233	0.89951227	-0.1165166	0.00342403	0.94146253	-0.2111766	0.02865892	0.44397423
P54071	Idh2	-0.1561232	0.0119749	0.77623265	-0.100666	0.00534146	0.85962444	-0.1519795	0.02729638	0.39743483
Q9ESY9	Ifi30 Gilt Ip30	-0.2182685	0.14528376	0.22005133	-0.3414193	0.14920765	0.42791442	-0.1939275	0.05888891	0.78330829
P01867	Igh-3	-0.1642355	0.01672088	0.8893756	-0.1221095	0.00552284	0.97604066	-0.112677	0.01019327	0.92435248
Q9QZ85	Iigp1 Irga6	-0.7046163	0.04870234	0.72852337	-0.4237422	0.04096867	0.55724393	-0.6035954	0.05433296	0.61273827
Q8BU33	Ilvbl	-0.3523748	0.03853703	0.91267236	-0.3017632	0.04306666	0.92466543	-0.2936573	0.03932844	0.93305775
Q8CAQ8	Immt	-0.1960974	0.01973921	0.5781887	-0.0991809	0.00428045	0.86610329	-0.1444883	0.02157835	0.38706544
P40936	Inmt Temt	-0.1556316	0.00709087	0.97175839	-0.1207767	0.0024554	0.9926153	-0.1744048	0.00701624	0.97169302
Q9JKF1	Iqgap1	-0.1163731	0.04006305	0.51330995	-0.0437005	0.04332565	0.16907345	-0.1558125	0.03846217	0.59870247
Q3UQ44	Iqgap2	-0.4783139	0.03330444	0.75758757	-0.2747434	0.01839588	0.75597831	-0.3835413	0.02683788	0.76425143
Q60766	Irgm1 Ifi1 Iigp	-0.3958629	0.04776732	0.74912634	-0.2088173	0.01242421	0.90981849	-0.3452161	0.03874967	0.75324605
P85094	Isoc2a Isoc2	-0.1477249	0.00457287	0.97478029	-0.0858009	0.00658236	0.86288202	-0.1459738	0.00477829	0.97492852
P05555	Itgam	-0.4232059	0.15214195	0.49166278	-0.4651498	0.09062208	0.68706141	-0.4978224	0.13945998	0.53669294
P09055	Itgb1	-0.5667155	0.09197128	0.75984964	-0.4028903	0.07651689	0.66446332	-0.6460177	0.1184328	0.69593461
P11835	Itgb2	-0.783927	0.11075596	0.75793378	-0.8203376	0.12837328	0.73135284	-1.0180909	0.12256702	0.79309468
P97328	Khk	-0.1908793	0.00822456	0.95564485	-0.1458388	0.00555816	0.95958004	-0.1841825	0.0073866	0.95986029
Q91WN4	Kmo	-0.2286759	0.02470035	0.61790946	-0.1563641	0.02297038	0.42380533	-0.3090395	0.0352442	0.57858917
O08677	Kng1 Kng	-1.0993548	0.15854965	0.85734123	-0.7939126	0.071043	0.94692259	-0.9533054	0.12890143	0.82007679

P70168	Kpnb1 Impnb	-0.3302328	0.02498787	0.90188754	-0.254598	0.01282039	0.95635022	-0.2649413	0.02134226	0.88512722
P05784	Krt18 Kerd Kr	-0.1118915	0.0026314	0.94216008	-0.0764585	0.00124552	0.96442597	-0.1069748	0.00243335	0.9410805
P19001	Krt19 Krt1-19	-0.0957995	0.01942012	0.82955227	-0.1080711	0.02351003	0.70130091	-0.1457168	0.05207421	0.56616741
P11679	Krt8 Krt2-8	-0.1352514	0.01399276	0.55143178	-0.0876474	0.00246484	0.93562477	-0.1109233	0.00313164	0.93865005
Q9EP89	Lactb Lact1	-0.1255469	0.00700556	0.96398167	-0.1085158	0.00792383	0.9036374	-0.1187622	0.00826142	0.91987743
Q9CPY7	Lap3 Lapep	-0.3235086	0.07624178	0.45006444	-0.1530377	0.05631873	0.21475087	-0.2056165	0.07062095	0.25322153
Q3U9G9	Lbr	-0.4785882	0.11774924	0.62292484	-0.2146902	0.07247639	0.38528224	-0.6371496	0.12907335	0.65210342
P11672	Lcn2	-0.1228436	0.03206921	0.57153977	-0.0407617	0.02172475	0.20093273	-0.0471045	0.02023709	0.29416419
Q61233	Lcp1 Pls2	-0.2492088	0.02873148	0.49419781	-0.1897541	0.01413493	0.67950751	-0.3268252	0.04061432	0.46005428
P06151	Ldha Ldh-1 Ld	-0.280367	0.02705874	0.60192657	-0.2257075	0.02342243	0.52803332	-0.2831481	0.02300559	0.63786704
Q7TNG8	Ldhd	-0.2755298	0.0133385	0.97486866	-0.1708916	0.00676669	0.97852123	-0.3102233	0.07399429	0.61507929
Q9Z2I0	Letm1	-0.3417421	0.03952742	0.68108776	-0.2400291	0.03818428	0.49077619	-0.3453432	0.04837492	0.59285163
P16045	Lgals1 Gbp	-0.6794365	0.119022	0.76518608	-0.1221559	0.02881489	0.59962539	-0.4041549	0.08224557	0.66802607
O08573	Lgals9	-0.9262804	0.08093669	0.83971965	-0.6652013	0.05663756	0.8262872	-0.7959666	0.08108025	0.78115312
O89017	Lgmn Prsc1	-0.2590685	0.01132309	0.97759016	-0.1840355	0.01345219	0.94448975	-0.2333318	0.02293135	0.89613565
Q9D7I5	Lhpp	-0.1838255	0.0118127	0.95278678	-0.2288408	0.02077663	0.90998803	-0.1893856	0.03127098	0.75348443
Q9D0F3	Lman1 Ergic53	-0.4909201	0.04972459	0.55866808	-0.3909156	0.03661409	0.56155631	-0.4663465	0.04389777	0.57622371
Q9DBH5	Lman2	-0.7108417	0.05648854	0.86365068	-0.65856	0.05813983	0.82614843	-0.6467633	0.05876782	0.8177139
P48678	Lmna Lmn1	-0.1104939	0.03534353	0.22327689	-0.0617575	0.00461263	0.81756769	-0.0806094	0.00291146	0.95158683
Q9DBN5	Lonp2	-0.2291626	0.14704901	0.23288177	-0.3144713	0.13740995	0.4279887	-0.3784624	0.13054554	0.51233441
Q91YX5	Lpgat1 Fam34	-0.2650998	0.10490089	0.26188558	-0.3616601	0.10142754	0.37711733	-0.2467259	0.07103017	0.36489615
Q91ZX7	Lrp1 A2mr	-0.3197961	0.07008779	0.39415839	-0.2658227	0.07202729	0.3052506	-0.2839404	0.07728412	0.28418244
Q922Q8	Lrrc59	-0.1063886	0.01176542	0.78798537	-0.0830866	0.01081189	0.6943163	-0.0834083	0.01047588	0.70130256
P08071	Ltf	-0.512327	0.07550985	0.56116244	-0.2950496	0.08487197	0.23203065	-0.3428079	0.08091225	0.32666495
P25911	Lyn	-0.7143578	0.12758044	0.58764329	-0.6412812	0.0973508	0.64387902	-0.5358037	0.11687937	0.53864263
P08905	Lyz2 Lyz Lyzs	-0.8004298	0.08492764	0.8809851	-0.8047229	0.06915303	0.90630194	-0.7012879	0.14044918	0.59458013
P24668	M6pr 46mpr	-0.8413309	0.28389386	0.74538663	-0.6087906	0.27204438	0.45493732	-0.8372956	0.10499874	0.8882527
Q922B1	Macrod1 Lrp16	-0.3147631	0.0634592	0.67215313	-0.1329843	0.00672192	0.96546569	-0.3149666	0.05496741	0.71636526
P27046	Man2a1 Man2	-0.8098313	0.05798031	0.76478605	-0.5738547	0.0466562	0.68366052	-0.7629406	0.05545747	0.73854788
Q9CXI5	Manf Armet	-0.50516	0.07296656	0.66634364	-0.5794208	0.08426474	0.62806542	-0.4821803	0.06557288	0.67529125
Q8BW75	Maob	-0.2187589	0.01325861	0.71408306	-0.1235556	0.00243925	0.9521289	-0.2966631	0.02226452	0.60897525
Q9CW42	Marc1 Mosc1	-0.2596511	0.01295732	0.78960188	-0.1954773	0.00702216	0.87077352	-0.3251163	0.01681591	0.76787068
Q922Q1	Marc2 Mg87	-0.5335396	0.03491588	0.78487414	-0.3371758	0.02185338	0.78036682	-0.5750689	0.03212376	0.82283604
Q91X83	Mat1a	-0.4920736	0.05616604	0.74697353	-0.2852531	0.02478581	0.80541301	-0.544706	0.07011014	0.70712918
P04247	Mb	-0.1475148	0.0556719	0.77829517	-0.2178128	0.14062947	0.54534205	-0.2888276	0.0879642	0.78231152
P41317	Mbl2	-0.6007169	0.085387	0.57222669	-0.2696197	0.04298256	0.47782353	-0.4335287	0.088606	0.39939128
Q99MR8	Mccc1 Mcca	-0.1708569	0.01252051	0.91634583	-0.2565721	0.08041827	0.40426789	-0.212962	0.02479037	0.8806638
Q3ULD5	Mccc2	-0.3335904	0.05131038	0.60152837	-0.275772	0.07197125	0.32139419	-0.245082	0.05033724	0.41079847
P14152	Mdh1 Mor2	-0.1809109	0.01187271	0.8788718	-0.1249512	0.0034712	0.97078107	-0.2168333	0.02612104	0.66960837
P08249	Mdh2 Mor1	-0.1579307	0.01816383	0.50534568	-0.122799	0.01364139	0.48805973	-0.1767846	0.01717131	0.56683138
P06801	Me1 Mod-1 Me	-0.3773003	0.02715122	0.56945612	-0.2271527	0.01799805	0.48081539	-0.3221568	0.02317074	0.53502572
Q9DCS3	Mecr Nrbf1	-0.1884521	0.01903486	0.96079093	-0.1218732	0.01202096	0.96254226	-0.2318384	0.01525087	0.98718443
Q9DD20	Mettl7b	-0.5830841	0.04424203	0.73695042	-0.4529687	0.02781578	0.79116207	-0.5943484	0.03467151	0.82345895
Q91VS7	Mgst1	-0.328374	0.05797	0.72781179	-0.2546996	0.07033104	0.46647366	-0.2677669	0.01463719	0.95710064
Q80UM7	Mogs Gcs1	-0.1020922	0.00271255	0.99229447	-0.093263	0.00337547	0.98199121	-0.0901265	0.00333192	0.9838638
P63030	Mpc1 Brp44l	-0.2153025	0.01422804	0.96218255	-0.1627817	0.00875376	0.97463342	-0.2343656	0.02072123	0.95519891
P11247	Mpo	-0.2491833	0.05364576	0.84360217	-0.3838873	0.1179138	0.63853949	-0.2774176	0.09366908	0.42228626
Q99J99	Mpst	-0.3708901	0.07628007	0.68245847	-0.1708445	0.03071618	0.72051576	-0.2254397	0.0181471	0.92230851
P19258	Mpv17	-0.2230697	0.00890835	0.98122148	-0.1502706	0.00699475	0.97879261	-0.204294	0.00941018	0.97719353
Q61830	Mrc1	-0.5107374	0.08256431	0.61455555	-0.2220453	0.09106081	0.23835288	-0.4966961	0.09405991	0.51749192
Q99N96	Mrpl1	-0.1555579	0.00952228	0.95696941	-0.1068781	0.00674037	0.94725453	-0.1462784	0.00749367	0.97194172
Q9EQI8	Mrpl46	-0.1684193	0.01309973	0.94836335	-0.0973955	0.0038525	0.98459489	-0.1324422	0.01051008	0.95777957
Q61733	Mrps31 Imogn	-0.2285141	0.00884921	0.98814522	-0.189225	0.00613686	0.98959141	-0.1861238	0.01660376	0.94014585
P26041	Msn	-0.364036	0.04733229	0.52276826	-0.3203801	0.05854827	0.369931	-0.318339	0.04879856	0.44535421
P30204	Msr1 Scvr	NA	NA	NA	NA	NA	NA	-0.3229515	0.0967329	0.69032931
Q791V5	Mtch2	-0.1661968	0.00554347	0.972939	-0.1067786	0.00288885	0.9792147	-0.1659593	0.00318121	0.9905371
P00397	Mtco1 COI m	-0.1779669	0.00611401	0.98603478	-0.1169765	0.00730392	0.95530697	-0.1666013	0.00521237	0.98743492
P00405	Mtco2 COII m	-0.1586103	0.00369101	0.98087751	-0.1092933	0.00352548	0.96389387	-0.1618286	0.00443043	0.97301618
Q922D8	Mthfd1	-0.2486522	0.01470542	0.61897365	-0.1758667	0.01073057	0.56715429	-0.2740949	0.0146967	0.64432996
P03911	Mtnd4 mt-Nd	-0.1366447	0.02445467	0.81685984	-0.1014234	0.00596046	0.98302468	-0.1027714	0.03096799	0.73357001
O08601	Mttp Mtp	-0.370194	0.03960752	0.51581889	-0.3128709	0.02628424	0.60117107	-0.3397016	0.03326085	0.50559995
O88441	Mtx2 MNCb-	-0.3973856	0.04781765	0.68336702	-0.2024326	0.00752151	0.94766819	-0.3057754	0.04079816	0.6299308
P16332	Mut	-0.4374814	0.0544366	0.76355376	-0.3273572	0.07232238	0.67200155	-0.3367306	0.0506694	0.71044582
Q9EQK5	Mvp	-0.2907855	0.02361201	0.8212961	-0.2162125	0.02611079	0.63156727	-0.2293783	0.01361745	0.88464004
O08638	Myh11	NA	NA	NA	NA	NA	NA	NA	NA	NA
Q8VDD5	Myh9	-0.4256486	0.01313747	0.75102553	-0.3374416	0.01284336	0.65412789	-0.4423282	0.0141634	0.72713757

Q3THE2	Myl12b Mrlc	-0.8076015	0.06322744	0.84467907	-0.670394	0.05417136	0.83166022	-0.8349122	0.04001544	0.93352461
Q642K0	Myl6 mCG_1	-0.2622077	0.01904278	0.96439405	-0.1302358	0.02537189	0.76709277	-0.1199452	0.0564848	0.42907349
Q60605	Myl6 Myln	-0.6386771	0.05317646	0.80028032	-0.3381593	0.03043386	0.73725175	-0.5900457	0.04605016	0.80017129
P46735	Myo1b	-0.2108186	0.02259131	0.89699572	-0.1832845	0.01172036	0.94585212	-0.2370528	0.0300603	0.8497013
Q9QWR8	Naga	-0.0949092	0.07714394	0.33534301	-0.0988714	0.00693957	0.99024346	-0.2511449	0.01210184	0.99768343
Q99KQ4	Nampt Pbef1	-0.6012996	0.12632952	0.71568854	-0.7370009	0.10955062	0.76374956	-0.381978	0.09053879	0.59730844
Q9DB05	Napa Snapa	-0.9040099	0.09299719	0.91303889	-0.8958215	0.07732364	0.91793209	-0.9083578	0.08967339	0.88755231
Q8BLF1	Nceh1 Aadac	-0.2809125	0.01280237	0.96203504	-0.3077136	0.03660899	0.80604878	-0.2607845	0.01547957	0.92503795
P09405	Ncl Nuc	-0.3418762	0.07421595	0.47987215	-0.3542342	0.04380905	0.67139469	-0.3320647	0.06814118	0.46795885
P57716	Ncstn	-0.2051153	0.04395844	0.75671346	-0.1970207	0.08992792	0.26965995	-0.20307	0.16312125	0.16228443
Q9QYG0	Ndrg2 Kiaa12	-0.9579294	0.05447317	0.86081896	-0.6701164	0.0429996	0.80455085	-0.8657262	0.0402254	0.89906661
Q99LC3	Ndufa10	-0.2275852	0.01436522	0.80707034	-0.1316748	0.00228133	0.97999664	-0.211515	0.01747846	0.69259214
Q9D8B4	Ndufa11	-0.2848399	0.0178881	0.96941375	-0.2247259	0.01751544	0.95921058	-0.317674	0.02160731	0.96431015
Q7TMF3	Ndufa12	-0.3222084	0.06453575	0.675036	-0.1312009	0.00443204	0.98427547	-0.33226	0.06498788	0.66785166
Q9ERS2	Ndufa13 Grir	-0.1808866	0.00986392	0.93080346	-0.1140997	0.00413518	0.96330706	-0.1976822	0.03700025	0.5139051
Q9CQ75	Ndufa2	-0.1575214	0.00362483	0.99368569	-0.1048257	0.0041346	0.97868419	-0.1536587	0.00477584	0.98759752
Q9CQ91	Ndufa3	-0.1754546	0.00461371	0.99177069	-0.1088261	0.00451931	0.97642534	-0.1838133	0.00386388	0.99428851
Q62425	Ndufa4	-0.4251895	0.05031567	0.85613223	-0.2202074	0.0071044	0.98563731	-0.5524576	0.07160846	0.8322168
Q9CPP6	Ndufa5	-0.1837815	0.0062438	0.98633842	-0.1145477	0.0067445	0.94126324	-0.2052649	0.01008097	0.97415389
Q9DCJ5	Ndufa8	-0.1509318	0.00518094	0.98605757	-0.0995594	0.00384943	0.97949971	-0.1522364	0.00385687	0.991725
Q9DC69	Ndufa9	-0.2913255	0.03488274	0.52149054	-0.1541895	0.01443667	0.61636356	-0.2281607	0.02249905	0.60196193
Q9DCS9	Ndufb10	-0.1715499	0.0072954	0.94529409	-0.1061813	0.00247658	0.97922439	-0.157916	0.00525948	0.9646871
O09111	Ndufb11 Np1	-0.5629076	0.13730794	0.62695919	-0.2311595	0.07468356	0.44393419	-0.6389744	0.15881306	0.64268767
Q9CQC7	Ndufb4	-0.1988848	0.00911312	0.96553735	-0.1307236	0.00388193	0.98352125	-0.2861515	0.04970236	0.68845101
Q9CR61	Ndufb7	-0.1517917	0.00635531	0.9694115	-0.0978877	0.00342954	0.9760386	-0.1347956	0.01395171	0.81634719
Q9D6J5	Ndufb8	-0.1575492	0.00954985	0.95777167	-0.0988895	0.00327745	0.98485491	-0.1567092	0.00776944	0.96903487
Q9CQJ8	Ndufb9	-0.1553181	0.00660961	0.97873077	-0.1023703	0.00311301	0.98721928	-0.150579	0.00420309	0.98997292
Q9CQ54	Ndufc2	-0.1457751	0.0074642	0.96949802	-0.0985203	0.00353183	0.98232613	-0.1473489	0.00405974	0.99022804
Q91VD9	Ndufs1	-0.1740306	0.0040089	0.94961005	-0.1111587	0.00242771	0.94628757	-0.1871034	0.01040413	0.77666197
Q91WD5	Ndufs2	-0.224802	0.01960892	0.60170228	-0.1269968	0.00280943	0.95068346	-0.2195282	0.0240612	0.48328714
Q9DCT2	Ndufs3	-0.1992116	0.01931162	0.58336139	-0.1206429	0.00303842	0.94656436	-0.1611927	0.01680712	0.53174386
Q9CXZ1	Ndufs4	-0.2534086	0.01329236	0.9811038	-0.2676296	0.03021565	0.86733309	-0.2777246	0.01263451	0.98571963
Q99LY9	Ndufs5	-0.1945964	0.0435402	0.44413677	-0.1094442	0.00285267	0.98067841	-0.1582606	0.00505227	0.97515486
P52503	Ndufs6 Ip13	-0.3349704	0.02855341	0.84627193	-0.1591103	0.00291243	0.99037696	-0.461902	0.05328132	0.73569213
Q9DC70	Ndufs7	-0.1847139	0.00976913	0.94203048	-0.1332385	0.00518022	0.95939386	-0.1818385	0.00779534	0.95775603
Q8K3J1	Ndufs8	-0.0989079	0.00564908	0.96232974	-0.0697403	0.00238841	0.98384514	-0.0882648	0.00533569	0.9546483
Q91YT0	Ndufv1	-0.2191632	0.01558049	0.70198703	-0.1295616	0.00256033	0.96097138	-0.2685975	0.02979236	0.48882018
Q9D6J6	Ndufv2	-0.175336	0.00579695	0.97443647	-0.1127068	0.00292993	0.98142913	-0.1663696	0.00595559	0.96895821
O70131	Ninj1	-0.2940604	0.03261455	0.93126554	-0.2367946	0.02022439	0.93839243	-0.28525	0.05030383	0.84274693
O55125	Nipsnap1	-0.1694298	0.02592126	0.38937435	-0.1254363	0.02694633	0.23638648	-0.1427654	0.00707046	0.84990909
Q9CQE1	Nipsnap3b N	-0.1720402	0.00631331	0.98409723	-0.1212152	0.00602819	0.96653399	-0.1616444	0.00314181	0.99511288
Q6GQT9	Nomo1	-0.1310099	0.0039478	0.98479805	-0.1126194	0.00514874	0.96568687	-0.1639835	0.08081008	0.2046859
Q99K48	Nono	NA	NA	NA	NA	NA	NA	NA	NA	NA
Q61937	Npm1	-0.3804318	0.06154296	0.56019307	-0.3352814	0.04439829	0.64056153	-0.322001	0.07086014	0.39220741
Q9R1J0	Nsdhl	-0.3961081	0.1033277	0.6201862	-0.3907743	0.05105323	0.81840431	-0.4729669	0.11309618	0.61388637
P11928	Oas1a Oias1	-0.3095387	0.0993782	0.46864265	-0.3680448	0.18102482	0.2562092	-0.4798186	0.14939075	0.4839531
P29758	Oat	-0.5704205	0.09276348	0.49876386	-0.4510688	0.07083588	0.50973549	-0.5263102	0.08157031	0.51631651
Q9CZ30	Ola1 Gtpbp9	-0.3637496	0.07025574	0.70904533	-0.1905074	0.01125401	0.96954889	-0.5134577	0.13029021	0.66001591
P58281	Opa1	-0.2104159	0.02104276	0.90908174	-0.137243	0.01826377	0.81286245	-0.2004572	0.02198043	0.93271345
Q78XF5	Ostc	-0.0826151	0.0023136	0.9906767	-0.0633023	0.00176352	0.98925126	-0.0744544	0.00276966	0.98232851
P11725	Otc	-0.1170237	0.00241627	0.96901624	-0.0744303	0.00228912	0.9272067	-0.1191573	0.00277418	0.95893763
Q9D0K2	Oxct1 Oxct S	-0.0272443	0.00804808	0.43309624	-0.0419084	0.01660172	0.27264331	-0.0304491	0.01465708	0.21243256
P09103	P4hb Pdia1	-0.3313223	0.01758793	0.67099795	-0.2456249	0.01116681	0.69934498	-0.3009856	0.01452451	0.69551019
P50580	Pa2g4 Ebp1 F	-0.1757675	0.00639801	0.97924029	-0.1354037	0.00451878	0.97927753	-0.1601973	0.00352379	0.99231791
P29341	Pabpc1 Pabp	-0.31426	0.16720433	0.4140058	-0.4225368	0.10877844	0.79044878	-0.3342153	0.07783475	0.69740126
P16331	Pah	-0.7798417	0.0808795	0.78807909	-0.5924814	0.06445053	0.76472236	-0.5865472	0.07461961	0.7202387
O88428	Papss2 Atpsk	-0.4290627	0.05220211	0.64612341	-0.1966448	0.01087639	0.89097411	-0.4078423	0.06056612	0.53761084
Q6TCG2	Paqr9	-0.7067127	0.11883007	0.74667429	-0.8972147	0.11152458	0.84359106	-0.6692062	0.09465662	0.79359344
Q9DCG6	Pbld1 Mawb	-0.1548397	0.0182949	0.87749847	-0.1007889	0.00371183	0.98136586	-0.1335389	0.00767237	0.96189747
Q05920	Pc Pcx	-0.2720832	0.02489292	0.41129504	-0.1594643	0.01815291	0.2794227	-0.23706	0.0216065	0.38909706
P61458	Pcbd1 Dcoh I	-0.1492745	0.01048692	0.94408639	-0.1434393	0.01933411	0.79722254	-0.1756298	0.0294993	0.73166249
Q99MN9	Pccb	-0.1308218	0.01292567	0.89513814	-0.0776714	0.00696985	0.89868805	-0.098637	0.00620081	0.95113441
Q9Z2V4	Pck1 Pepck	-0.2058702	0.05703978	0.61952971	-0.1764728	0.02448613	0.91219074	-0.2960041	0.03710041	0.91386219
Q9CQF9	Pcyox1 Kiaa0	-0.5717449	0.04203318	0.71986678	-0.4888172	0.04521577	0.60911586	-0.5101809	0.04610592	0.61392533
Q9WU78	Pdcd6ip Aip1	-0.9242679	0.12717723	0.86845871	-0.6343404	0.14778686	0.64817892	-0.6955567	0.12962239	0.74222947
P35486	Pdha1 Pdha-	-0.1212376	0.01685894	0.77516216	-0.0981933	0.00970662	0.84341002	-0.1208684	0.01357813	0.79846892

Q9D051	Pdhb	-0.1996416	0.05470048	0.30748693	-0.23269	0.0682712	0.24396168	-0.1869238	0.05971936	0.21869993
D3Z6P0	Pdia2 Pdip	-0.0522016	0.09967509	0.02234592	-0.0198504	0.00528349	0.58532944	-0.019483	0.01311602	0.1807661
P27773	Pdia3 Erp Erp	-0.2689837	0.0085858	0.89512082	-0.2290205	0.00793332	0.86685676	-0.2560515	0.0119517	0.78730029
P08003	Pdia4 Cai Erp	-0.3203718	0.04806129	0.63994703	-0.2447576	0.02976374	0.69986596	-0.2521486	0.02968324	0.72771007
Q921X9	Pdia5 Pdir	-0.1196529	0.02096576	0.36362998	-0.0899261	0.00197994	0.96990865	-0.1034281	0.00193446	0.97877164
Q922R8	Pdia6 Txndc7	-0.3043206	0.02731505	0.59354432	-0.2574623	0.02050075	0.61676884	-0.3129311	0.02236511	0.67794891
Q9JIL4	Pdzk1 Cap70	-0.7858688	0.07207441	0.83790029	-0.536105	0.05399732	0.79128602	-0.75727	0.06421384	0.85282707
P70296	Pebp1 Pbp Pe	-0.2203664	0.02876582	0.5659989	-0.1225455	0.00485346	0.92862523	-0.1645437	0.00374432	0.9747622
Q99MZ7	Pecr	-0.7362589	0.08208486	0.87020245	-0.5646043	0.08529177	0.75786995	-0.5460903	0.09910248	0.70021257
P62962	Pfn1	-0.272706	0.02793407	0.73705816	-0.1839303	0.00534831	0.97125717	-0.2431688	0.01110806	0.93557518
Q9DBJ1	Pgam1	-0.168984	0.0114007	0.94820865	-0.3388937	0.07304519	0.60591165	-0.3864174	0.09943598	0.53739475
Q9DCD0	Pgd	-0.5741995	0.07998134	0.81114392	-0.3123471	0.08613847	0.48432033	-0.485898	0.12903853	0.52169293
P09411	Pgk1 Pgk-1	-0.1623725	0.00800303	0.89169019	-0.1475532	0.01591525	0.58490623	-0.1651421	0.02367033	0.47406934
Q9CQ60	Pgls	-0.1658195	0.02643743	0.81381828	-0.1730788	0.00475062	0.99178096	-0.1260082	0.02794826	0.64887308
Q9D0F9	Pgm1 Pgm2	-0.1537417	0.01151192	0.89019538	-0.1214915	0.0081098	0.90704254	-0.2352669	0.03367241	0.66132567
O55022	Pgrmc1 Pgrm	-0.7206932	0.05596367	0.81758997	-0.5878724	0.04201393	0.83389061	-0.7099795	0.04761302	0.84753279
Q80UU9	Pgrmc2	-0.7061356	0.06910178	0.90469868	-0.3759083	0.06558393	0.78495935	-0.6550408	0.07351308	0.87831538
P67778	Phb	-0.1276532	0.00164406	0.98400465	-0.0888486	0.00188093	0.95098642	-0.1257801	0.00295219	0.9453194
O35129	Phb2 Bap Bca	-0.1232629	0.00218659	0.98941416	-0.0894452	0.00303261	0.95394358	-0.1246613	0.00371274	0.96656351
Q7M6Y3	Picalm Calm	-0.3677793	0.03662593	0.93508402	-0.3077067	0.03085836	0.98028246	-0.3201587	0.04439758	0.89655357
Q9D826	Pipox Pso	-0.3497049	0.01161148	0.98694294	-0.343384	0.00995799	0.99166039	-0.3777529	0.0633395	0.73233714
P53810	Pitpna Pitpn	-0.1062306	0.01683793	0.85043855	-0.3664025	0.12157708	0.47596428	-0.0836366	0.01157472	0.82598269
P53811	Pitpnb	-0.3179715	0.07172291	0.66278235	-0.2232579	0.03368716	0.82993931	-0.1181653	0.02431028	0.77143991
P53657	Pklr	-0.2360274	0.02506132	0.50197695	-0.1915262	0.01949954	0.51186756	-0.2494212	0.0160582	0.7132325
P52480	Pkm Pk3 Pkm	-0.3603947	0.046294	0.50670898	-0.1951307	0.03612197	0.34665002	-0.3003171	0.035752	0.5575227
Q99P27	Pla2g12b Pla	-0.5519189	0.13798663	0.76188663	-0.4143497	0.22776531	0.62331481	-0.3127059	0.10660934	0.51817765
Q8VCI0	Plbd1	-0.1659076	0.12709409	0.12434627	-0.0464984	0.01375282	0.4167232	-0.0719794	0.02547518	0.34735214
Q8BG07	Pld4	-0.1195182	0.06406948	0.17864017	-0.048313	0.00935411	0.57151504	-0.1312355	0.05089089	0.24050665
Q9QXS1	Plec Plec1	-0.2402107	0.01183732	0.97861177	-0.1549354	0.0244491	0.80063135	-0.2077145	0.02555351	0.91675261
P20918	Plg	-0.503838	0.17611972	0.57698826	-0.3162715	0.05176032	0.90323175	-0.265409	0.06102482	0.72989162
P43883	Plin2 Adfp Ad	-0.4881029	0.11708157	0.5915566	-0.3682479	0.12869759	0.40556635	-0.6540648	0.13818591	0.63280371
Q99K51	Pls3	-0.3020959	0.01587125	0.9679401	-0.1856383	0.00563707	0.98905604	-0.2405634	0.01064891	0.98078133
Q8C165	Pm20d1	-0.492166	0.06447353	0.74448096	-0.3580513	0.04873013	0.69225944	-0.390474	0.03760022	0.82422065
Q6P8U6	Pnlip	-0.3731585	0.09250426	0.28412828	-0.3359233	0.06441438	0.37155547	-0.2127882	0.06307766	0.22588396
P23492	Pnp Np Pnp1	-0.2040517	0.1022423	0.26583862	-0.1809585	0.06374683	0.36531689	-0.270453	0.09343183	0.39192758
P52430	Pon1 Pon	-0.8139206	0.05110678	0.80612376	-0.5497441	0.02428727	0.88741592	-0.768961	0.05015706	0.77817613
Q62086	Pon2	-0.1825116	0.04549093	0.76299379	-0.2370862	0.02558087	0.93471006	-0.1468012	0.02693392	0.69560105
Q62087	Pon3	-0.222377	0.01826493	0.88638575	-0.1983286	0.0256249	0.86938097	-0.2036365	0.0164314	0.86975468
P37040	Por	-0.4305995	0.02190485	0.75264209	-0.2941281	0.0142866	0.75304397	-0.4486985	0.02167017	0.7564982
Q9D819	Ppa1 Pp Pyp	-0.12925	0.01735065	0.86044766	-0.1366616	0.00869812	0.96106762	-0.1613177	0.01136697	0.96641193
P17742	Ppia	-0.288704	0.03791313	0.69874585	-0.1654572	0.00342385	0.98773422	-0.3604107	0.04360389	0.71674227
P24369	Ppib	-0.527544	0.09087591	0.70649418	-0.3995034	0.06467714	0.71780075	-0.4956452	0.06843413	0.75524123
P35700	Prdx1 Msp23	-0.214232	0.00486476	0.97437577	-0.1611341	0.00359901	0.97140791	-0.2103761	0.00472473	0.97300764
P20108	Prdx3 Aop1 M	-0.0861186	0.01201587	0.81062696	-0.1658162	0.07679523	0.24981812	-0.2400285	0.07606142	0.43376254
O08807	Prdx4	-0.4432212	0.05811116	0.68299842	-0.3790322	0.05154573	0.63559979	-0.3409386	0.04958287	0.58169938
P99029	Prdx5 Prdx6	-0.1736821	0.00638323	0.97114134	-0.1353668	0.00559125	0.95909329	-0.1683913	0.00499524	0.97847401
O08709	Prdx6 Aop2 L	-0.2118724	0.01643087	0.74806026	-0.1654125	0.01499458	0.66248162	-0.1963352	0.00456708	0.97058931
Q9JK53	Prelp	-0.0808819	0.05479009	0.42076112	-0.0394991	0.06079258	0.2968429	-0.1465318	0.04446321	0.91568868
Q9WU79	Prodh Pro1	-0.5132818	0.05708201	0.68028588	-0.422736	0.05319793	0.59489934	-0.4725157	0.06195243	0.61123113
P07146	Prss2 Try2	-0.1920083	0.08742118	0.54669044	-0.081326	0.03748972	0.40200632	-0.1149727	0.04576772	0.55793708
Q61096	Prtn3	-0.1340878	0.03152965	0.85772494	-0.4138835	0.37011766	0.38470622	-0.3075856	0.2936323	0.21527044
Q61207	Psap Sgp1	-0.4074883	0.03961856	0.82141074	-0.2028131	0.00654148	0.97169593	-0.7592018	0.05958169	0.86196912
P49722	Psma2 Lmpc3	-0.2449048	0.01233049	0.98256488	-0.1968545	0.01331754	0.96895728	-0.2237522	0.0090204	0.98400755
Q9R1P1	Psmb3	-0.5294394	0.09876132	0.70543578	-0.3425991	0.08647024	0.52858481	-0.5162552	0.09222858	0.70676271
O35593	Psmd14 Pad1	-0.2691887	0.01333466	0.98074704	-0.2207779	0.02612215	0.9469722	-0.2031963	0.02263367	0.95271727
Q8VDM4	Psmd2	-0.6896377	0.0639411	0.8925782	-0.3790301	0.08106874	0.53499212	-0.4387958	0.0861326	0.63372761
P97371	Psme1	-1.0825428	0.52237962	0.58873408	-2.0239309	0.26836779	0.93429287	-0.8986201	0.23943936	0.77882394
P97372	Psme2 Pa28b	-0.2364176	0.00830267	0.98183621	-0.1782632	0.00746885	0.98105606	-0.2218567	0.00625264	0.99055845
P17225	Ptbp1 Ptb	-0.3235167	0.04029729	0.78169323	-0.194381	0.00678033	0.97624352	-0.2370337	0.00647848	0.98673224
Q60866	Pter	-0.21141	0.01013873	0.98193293	-0.1669145	0.00956005	0.96517193	-0.2043658	0.01741108	0.95826764
Q8BWM0	Ptges2 Gbf1	-0.2430409	0.01066642	0.97925249	-0.1704345	0.01504544	0.90163232	-0.2965217	0.02912303	0.90406993
P22437	Ptgs1 Cox-1 C	-0.3645435	0.04097732	0.77482509	-0.2653259	0.04417454	0.62118429	-0.4991844	0.05527021	0.75830087
Q8K2C9	ptplad1 hacd	-0.1860637	0.01357166	0.93998699	-0.1673801	0.0059327	0.98393043	-0.1786545	0.00744054	0.98127744
Q66GT5	Ptpmt1 Plip	-0.2186341	0.00907317	0.98473682	-0.1475973	0.00707122	0.97319519	-0.1944856	0.02053401	0.89077246
P06800	Ptprc Ly-5	-0.4719468	0.14056324	0.48438277	-0.5028995	0.12102003	0.5705068	-0.2145147	0.07651293	0.37680948
P32848	Pvalb Pva	-0.2076756	0.19211073	0.28033474	-0.472508	0.21100105	0.62569002	-0.1963626	0.00556866	0.99919641

P42925	Pxmp2 Pmp2	-0.3247547	0.02096854	0.96383644	-0.3062838	0.01317155	0.98721981	-0.2686657	0.02952587	0.90195848
Q9EPB4	Pycard Asc	-0.0774788	0.10774803	0.14701656	-0.0321677	0.01216128	0.6998959	-0.08377	0.05144496	0.39863164
Q9ET01	Pygl	-0.1407338	0.01177966	0.57616057	-0.1043035	0.01603616	0.27595572	-0.1349512	0.00430678	0.89028476
Q8BVI4	Qdpr Dhpr	-0.2736782	0.04393698	0.43693033	-0.1796517	0.02596531	0.48912539	-0.2347549	0.03322167	0.49966398
Q8R404	Qil1	-0.7834943	0.09629343	0.84655341	-0.6605837	0.0693496	0.86632743	-0.8200803	0.05064858	0.95973161
Q91X91	Qprt	-0.4334061	0.09947332	0.61269742	-0.099315	0.01218021	0.82605348	-0.275499	0.0697217	0.54567128
P61027	Rab10	-0.2795249	0.01309503	0.97433961	-0.2226338	0.00420602	0.99538158	-0.3896458	0.0676222	0.73452376
Q91V41	Rab14	-0.3847416	0.07236338	0.7386875	-0.2018147	0.01025396	0.9675298	-0.352994	0.05337744	0.79902802
P35292	Rab17	-0.1633331	0.14315543	0.39426358	-0.099003	0.02232303	0.83100524	-0.111246	0.12614313	0.27999335
P62821	Rab1A Rab1	-0.322259	0.00898807	0.99075156	-0.2629446	0.00725622	0.98945093	-0.3761515	0.04802595	0.82513775
P51150	Rab7a Rab7	-0.8395373	0.0884367	0.78283285	-0.6362034	0.06509968	0.77329184	-0.6433787	0.09487621	0.63006232
Q99JI6	Rap1b	-0.3429595	0.05638374	0.75509188	-0.1791505	0.01187875	0.94201807	-0.1833246	0.02514372	0.80350567
Q8BU31	Rap2c	-0.5417816	0.09460596	0.73211466	-0.3755139	0.07948621	0.61452337	-0.5209246	0.12119822	0.58695861
O89086	Rbm3	-0.4520195	0.10754688	0.77939753	-0.5798321	0.15546101	0.77667482	-0.2579617	0.06593434	0.75377826
Q9QYF1	Rdh11 Arsdr1	NA	NA	NA	NA	NA	NA	-0.1530742	0.02942484	0.93118395
O88451	Rdh7 Crad2	-0.6431067	0.04356004	0.851543	-0.3334354	0.03132766	0.72485937	-0.6114933	0.03803078	0.86311939
P26043	Rdx	-0.2745343	0.03519205	0.61560293	-0.1611685	0.01611709	0.71427965	-0.2035329	0.02605847	0.63543879
Q9JM62	Reep6 Dp1l1	-0.2569062	0.02970962	0.86171097	-0.1970663	0.01481476	0.92668004	-0.2164489	0.02321277	0.87872343
Q64FW2	Retsat Ppsig	-0.2049437	0.0899584	0.42576914	-0.3499051	0.03851422	0.93223312	-0.3020907	0.08300121	0.68825735
Q64374	Rgn Smp30	-0.1498842	0.00457168	0.94964128	-0.1103878	0.00233322	0.97051641	-0.1522266	0.0066985	0.88820999
Q9CXW4	Rpl11	-0.117444	0.00259812	0.9941616	-0.1032316	0.00273608	0.99026113	-0.105637	0.00224545	0.99416054
P35979	Rpl12	-0.0877879	0.00167279	0.98639042	-0.0966442	0.00205068	0.98057424	-0.0756977	0.00165323	0.98081884
P47963	Rpl13	-0.0563796	0.0102036	0.71785121	-0.0597504	0.01023159	0.70895954	-0.0500066	0.00914862	0.69681068
Q9CR57	Rpl14	-0.1010175	0.00588698	0.96084169	-0.1041162	0.00335066	0.98570778	-0.0901671	0.00311903	0.98468272
Q9CZM2	Rpl15	-0.0792125	0.01136571	0.66020103	-0.0744171	0.00824286	0.75116568	-0.1002506	0.01397608	0.65584117
Q9CPR4	Rpl17	-0.1120627	0.00418532	0.98353705	-0.1008987	0.00350474	0.98455722	-0.1038668	0.00323919	0.98751447
P35980	Rpl18	-0.1135971	0.00262452	0.99416266	-0.1025462	0.00293793	0.98944208	-0.1064051	0.00241727	0.99333554
P62717	Rpl18a	-0.1120279	0.00292027	0.99191187	-0.1131533	0.00359763	0.98604523	-0.0993072	0.00272735	0.99028985
P84099	Rpl19	-0.164147	0.00711683	0.97794026	-0.1472699	0.00484216	0.98509082	-0.157863	0.00584802	0.98247248
P67984	Rpl22	-0.0946755	0.00169475	0.99205286	-0.0956118	0.00231715	0.9832525	-0.0810972	0.00213274	0.98166865
Q9D7S7	Rpl22l1	-0.3248614	0.02270434	0.96693879	-0.4347398	0.12633922	0.66369289	-0.3002217	0.00896483	0.9946785
P62830	Rpl23	-0.1101517	0.00390928	0.9694728	-0.1124195	0.00445803	0.95782585	-0.0979579	0.00293744	0.97629698
Q8BP67	Rpl24	-0.1320732	0.00380414	0.99014262	-0.1277443	0.00341261	0.99010761	-0.1251186	0.00298925	0.9926343
P61358	Rpl27	-0.1148712	0.00345045	0.98928894	-0.1122927	0.00262604	0.99240172	-0.1002456	0.00385483	0.98113953
P14115	Rpl27a	-0.1419007	0.01098013	0.94350745	-0.1104941	0.00865859	0.96446519	-0.168698	0.01609355	0.90900015
P27659	Rpl3	-0.0861957	0.00164854	0.96847128	-0.0874634	0.00176176	0.96326204	-0.0946173	0.01296614	0.35440965
P62889	Rpl30	-0.1006375	0.00354899	0.94035792	-0.1038112	0.00430803	0.91491676	-0.0904246	0.00301529	0.94236733
P62900	Rpl31	-0.1147597	0.00265311	0.99362713	-0.1061979	0.00319329	0.98926657	-0.1020113	0.0031617	0.98766614
Q9D8E6	Rpl4	-0.110458	0.00233721	0.98893102	-0.1092841	0.00311919	0.98004029	-0.1023094	0.00254914	0.98351453
P47962	Rpl5	-0.0869294	0.00279898	0.98771211	-0.0954414	0.00358451	0.9806348	-0.0826182	0.00502778	0.95406721
P47911	Rpl6	-0.1137228	0.00239885	0.98639426	-0.1109627	0.00294166	0.97666247	-0.1022494	0.00191565	0.98786398
P14148	Rpl7	-0.1415845	0.0291017	0.38381528	-0.1076353	0.00185224	0.98713774	-0.1456298	0.02372151	0.4789623
P12970	Rpl7a Surf-3	-0.1199912	0.00252741	0.98903007	-0.1153803	0.00276547	0.98584132	-0.1099508	0.00391981	0.96801199
P62918	Rpl8	-0.0946504	0.00380389	0.95231794	-0.1539402	0.04385414	0.27801165	-0.0877051	0.00343286	0.94773027
P51410	Rpl9	-0.1176829	0.01883414	0.35804737	-0.1043943	0.00229116	0.96648156	-0.1344191	0.02158223	0.346995
P14869	Rplp0 Arbp	-0.0901152	0.00185774	0.97352106	-0.0926578	0.00159711	0.97962665	-0.1041118	0.01379954	0.45203647
P47955	Rplp1	-0.1094212	0.00331706	0.97842052	-0.1044938	0.0026005	0.98295401	-0.1563422	0.03626902	0.40765433
P99027	Rplp2	-0.1438998	0.00464624	0.96771654	-0.1247867	0.00279425	0.98420826	-0.1274886	0.0027068	0.98446754
Q91YQ5	Rpn1	-0.118279	0.00222496	0.95926654	-0.087815	0.0010902	0.97873032	-0.1177576	0.00164142	0.97554891
Q9DBG6	Rpn2	-0.137063	0.00193721	0.97754322	-0.1099372	0.00135819	0.97981131	-0.1258315	0.0019893	0.97088158
P63325	Rps10	-0.0963897	0.00526529	0.96543101	-0.0926964	0.00813067	0.96295715	-0.0905786	0.0033955	0.98205938
P62281	Rps11	-0.713268	0.10671622	0.80241751	-0.6270116	0.0810847	0.83286016	-0.4797899	0.08625792	0.7041343
P63323	Rps12	-0.0790291	0.00278776	0.98528772	-0.080101	0.00874321	0.93328382	-0.064234	0.00293496	0.97357674
Q6ZWZ6	Rps12 LOC10	-0.0863252	0.0029477	0.98620133	-0.086957	0.00491518	0.97204886	-0.0717004	0.00264816	0.98257573
P62264	Rps14	-0.1192433	0.0029554	0.98547157	-0.1183486	0.00243898	0.98783334	-0.1033415	0.00429298	0.96023008
P62843	Rps15 Rig	-0.1206056	0.00643594	0.93353986	-0.113796	0.00284184	0.98223525	-0.1107188	0.00498898	0.94802849
P62245	Rps15a	-0.108347	0.00444595	0.98019431	-0.1115662	0.0034979	0.98642494	-0.1096687	0.00578798	0.96505504
P14131	Rps16	-0.110517	0.0047185	0.97859408	-0.1082752	0.00399932	0.98125759	-0.098883	0.00351818	0.98380998
P63276	Rps17	-0.1248613	0.00290343	0.98666247	-0.1195805	0.00300542	0.98506636	-0.1133303	0.00308198	0.98042305
P62270	Rps18	-0.1020156	0.0026037	0.99224384	-0.0988036	0.00289184	0.98898622	-0.0914946	0.00237059	0.99134849
Q9CZX8	Rps19	-0.0939761	0.00314019	0.97284436	-0.1013026	0.00279501	0.97840062	-0.0828648	0.00319239	0.96147074
P25444	Rps2 Llrep3 F	-0.1131426	0.00233241	0.97837937	-0.1111293	0.00283293	0.972816	-0.1007805	0.00254749	0.96426482
P62267	Rps23	-0.105043	0.00232233	0.98792805	-0.0997215	0.0035385	0.97543647	-0.1009911	0.00472874	0.94803742
P62849	Rps24	-0.0923413	0.00349181	0.98313055	-0.0950423	0.00400814	0.97570606	-0.0772371	0.00387709	0.96828205
P62855	Rps26	-0.1155762	0.00381864	0.97343392	-0.1270315	0.00460262	0.96332599	-0.0972188	0.00381805	0.96002154
P62858	Rps28	-0.136322	0.0208961	0.80974111	-0.1148793	0.00482402	0.98608958	-0.1395605	0.01536875	0.86381859

P62908	Rps3	-0.1172829	0.00387494	0.94922776	-0.1210676	0.00240476	0.97951777	-0.1089804	0.00317376	0.95776119
P97351	Rps3a	-0.1037461	0.00469469	0.9367027	-0.099781	0.00306485	0.97515947	-0.0886681	0.002899	0.96592625
P62702	Rps4x Rps4	-0.0946494	0.00438617	0.95488625	-0.1046667	0.00695358	0.91888674	-0.0817956	0.00387921	0.95695268
P97461	Rps5	-0.1018916	0.00431731	0.95704432	-0.115649	0.00548026	0.94886316	-0.0782032	0.00512694	0.89602019
P62754	Rps6	-0.1065338	0.0033727	0.98811579	-0.1095136	0.00420914	0.97973767	-0.0868262	0.00499021	0.95882637
P62082	Rps7	-0.1349267	0.00520904	0.93323463	-0.1211323	0.00317576	0.97259127	-0.1576294	0.01793937	0.59295773
P62242	Rps8	-0.0899298	0.00217586	0.97101005	-0.0977069	0.00240034	0.96617935	-0.0884044	0.02055734	0.25163224
P14206	Rpsa Lamr1 F	-0.0950471	0.002365	0.98475762	-0.0909653	0.00316119	0.97756889	-0.0826371	0.00200272	0.98438931
Q99PL5	Rrbp1	-0.7078682	0.04547043	0.80958873	-0.5848721	0.04300248	0.75201667	-0.7303135	0.03893369	0.85224945
Q01730	Rsu1 Rsp1	-0.2679081	0.01029336	0.98402137	-0.1688978	0.00992448	0.96340928	-0.2247704	0.01194318	0.97791225
P50543	S100a11 S10	-0.1679688	0.01251957	0.96774237	-0.3471849	0.20414732	0.41963715	-0.2313993	0.04758251	0.77161432
P27005	S100a8 Caga	-0.4818361	0.06421073	0.66789126	-0.3681531	0.06588328	0.5456555	-0.5048864	0.07924051	0.55161152
Q60710	Samhd1 Mg1	-0.1497523	0.06399001	0.33239255	-0.1153051	0.01564153	0.85791488	-0.1460806	0.01558777	0.90704868
Q8BGH2	Samm50	-0.1149957	0.01713025	0.93758402	-0.1016847	0.00824397	0.97438164	-0.1352079	0.00812482	0.99282986
Q9CQC9	Sar1b Sara1b	-0.2960185	0.01601693	0.94994014	-0.2763108	0.01357144	0.97874947	-0.2814187	0.01377511	0.96307958
Q99LB7	Sardh	-0.2155061	0.02112644	0.47939681	-0.1696667	0.02354145	0.29027678	-0.2219126	0.02329157	0.40384825
Q61009	Scarb1 Srb1	-0.3358391	0.02601977	0.95967568	-0.331286	0.04135328	0.96977848	-0.3189061	0.04455279	0.91108912
P32020	Scp2 Scp-2	-0.7340078	0.04288603	0.74361183	-0.5085387	0.02698926	0.74425134	-0.6186675	0.03950242	0.667831
Q8K2B3	Sdha	-0.2264065	0.01199708	0.70087289	-0.1119015	0.00185947	0.95415667	-0.2345757	0.01267311	0.68302069
Q9CQA3	Sdhb	-0.1501434	0.00667078	0.88012366	-0.0980596	0.00368448	0.89285495	-0.1409184	0.00653389	0.86114942
Q8VBT2	Sds	-0.5238177	0.03777611	0.69344658	-0.2973352	0.02669191	0.55623096	-0.5535771	0.03661581	0.73834664
Q99J08	Sec14l2	-0.3313265	0.03841738	0.56614445	-0.235114	0.02264272	0.62029713	-0.3834261	0.0411516	0.59537499
O08547	Sec22b Sec22	-0.5894282	0.11488921	0.56823093	-0.7541556	0.09876032	0.78469119	-0.6718911	0.12134831	0.59347268
Q01405	Sec23a Sec23	-0.1950779	0.01399896	0.96043307	-0.1882846	0.01673156	0.93364587	-0.2002934	0.02015599	0.93380441
Q3UPL0	Sec31a Sec31	-0.2825671	0.01550816	0.96792892	-0.2473926	0.01213369	0.97650967	-0.2308348	0.02494901	0.93450095
P61620	Sec61a1 Sec6	-0.2293804	0.00631256	0.986551	-0.197642	0.00605712	0.98246743	-0.2487278	0.00449921	0.99220819
P17563	Selenbp1 Lps	-0.1732574	0.01145976	0.95408576	-0.130269	0.00680378	0.9657526	-0.185282	0.01200981	0.95200179
Q9CY58	Serbp1 Pairb	-0.2052081	0.02401199	0.87956927	-0.1614701	0.0233144	0.81345258	-0.1520666	0.02727706	0.75656895
Q00897	Serpina1d Dc	-0.5456741	0.08987048	0.75443328	-0.4278618	0.08385407	0.70298428	-0.5874574	0.16431159	0.49578238
P07759	Serpina3k M(	-0.1693661	0.11815677	0.15738777	-0.3542343	0.11512018	0.42141053	-0.0375551	0.01881143	0.23464562
Q99JR1	Sfxn1 F	-0.1441597	0.00363046	0.96747984	-0.092735	0.00364074	0.91406	-0.1431488	0.00497269	0.94417169
Q925N2	Sfxn2	-0.3688288	0.11412075	0.46536666	-0.1230526	0.00955083	0.92222076	-0.1015767	0.02692182	0.54260811
Q91V61	Sfxn3	-0.1911809	0.01665262	0.93608071	-0.1362983	0.005745	0.98083159	-0.1905585	0.01515526	0.94613984
Q8R0X7	Sgpl1	-0.3534411	0.039863	0.90763506	-0.6590976	0.07745145	0.86813234	-0.265121	0.01018275	0.99122663
P50431	Shmt1 Shmt	-0.0966458	0.01827949	0.55959123	-0.0565607	0.01084118	0.54200812	-0.0856521	0.01446621	0.57416384
Q62230	Siglec1 Sa Sn	-0.2512786	0.10952617	0.3968413	-0.156922	0.04635431	0.56011879	-0.3903898	0.16976065	0.51401493
O55242	Sigmar1 Oprs	-0.7526064	0.07244373	0.89993999	-0.5815962	0.07166917	0.82467879	-0.7744323	0.07839342	0.88244927
O08705	Slc10a1 Ntcp	-0.9926057	0.10891587	0.87375867	-0.526972	0.08067812	0.75293037	-0.9410644	0.09959437	0.87290174
P53986	Slc16a1 Mct1	-0.242357	0.01026085	0.97894312	-0.1783877	0.00599284	0.98444554	-0.2328316	0.0079934	0.98490896
Q9QZD8	Slc25a10 Dic	-0.2574249	0.0052777	0.97620115	-0.184457	0.01450823	0.70696882	-0.2546929	0.0062354	0.96752533
Q9CR62	Slc25a11	-0.1447435	0.00360682	0.98471374	-0.1025005	0.0039967	0.95777099	-0.1414645	0.00297785	0.98817748
Q8BH59	Slc25a12 Ara	-0.0903058	0.01503952	0.5718008	-0.0647443	0.00909431	0.59850336	-0.093143	0.01443237	0.59799532
Q9QXX4	Slc25a13 Ara	-0.1507513	0.00949827	0.60860351	-0.1046504	0.0056691	0.63249179	-0.1610244	0.01123753	0.5441597
Q9WVD5	Slc25a15 Orr	-0.1942259	0.00547275	0.95525265	-0.1481147	0.00457172	0.9383175	-0.1951317	0.00815162	0.91097235
Q9Z2Z6	Slc25a20 Cac	-0.139126	0.00850681	0.87847934	-0.0902721	0.00458708	0.9000668	-0.1790789	0.02336593	0.61353052
Q9D6M3	Slc25a22 Gc1	-0.32142	0.03693971	0.60226217	-0.2440075	0.02963038	0.53900918	-0.3258884	0.04443462	0.51825424
Q8VEM8	Slc25a3	-0.1933611	0.00937257	0.9362103	-0.1241633	0.0047356	0.9622083	-0.1693208	0.00719314	0.95026542
Q8R0Y8	Slc25a42	-0.1572486	0.00780313	0.97129895	-0.1239736	0.01063847	0.93141294	-0.1397167	0.00910464	0.94768385
P51881	Slc25a5 Ant2	-0.1714474	0.03041105	0.46207855	-0.1020954	0.0028832	0.96609907	-0.1840966	0.02163911	0.63838109
O35488	Slc27a2 Acsv	-0.456269	0.02832929	0.66444692	-0.3041132	0.01646873	0.70164413	-0.4638075	0.0228745	0.73398729
Q4LDG0	Slc27a5 Acsb	-0.5128888	0.02249679	0.79625066	-0.3390439	0.01580821	0.76158461	-0.5207781	0.02298651	0.78330075
P14246	Slc2a2 Glut-2	-0.1749286	0.05008727	0.6702823	-0.2354663	0.02795809	0.91017819	-0.2376995	0.04396286	0.80680993
Q75N73	Slc39a14 Fad	-0.6764593	0.15978652	0.61967572	-0.8270245	0.11509517	0.79886261	-0.9009034	0.11033889	0.85836643
P10852	Slc3a2 Mdu1	-0.2235944	0.05686994	0.44860569	-0.3118001	0.08775579	0.38695659	-0.4520505	0.10448123	0.48346612
P04919	Slc4a1 Ae1	-0.0443192	0.02542258	0.06601119	-0.0408928	0.01211228	0.18564541	-0.0280474	0.00419207	0.48781505
Q9JJL3	Slco1b2 Oatp	-0.9753808	0.06620047	0.90419989	-0.586503	0.05649535	0.78797184	-0.9045535	0.07264378	0.85168907
Q78PY7	Snd1	-0.2585828	0.01475987	0.70407846	-0.2136214	0.01401248	0.63256562	-0.2898757	0.01920598	0.63137298
P62315	Snrpd1	-0.1000848	0.00651169	0.96330082	-0.0634101	0.00556308	0.92194332	-0.0953961	0.00519733	0.96560618
P08228	Sod1	-0.1642295	0.00619966	0.93224622	-0.1268772	0.00256584	0.97682936	-0.1511751	0.00536487	0.93522086
Q64442	Sord Sdh1	-0.2236914	0.0204275	0.59388555	-0.1474014	0.0040291	0.9369927	-0.2068805	0.01299765	0.75099558
Q9CYN2	Spcs2 Spc25	-0.8404408	0.07902324	0.90408513	-0.4636823	0.09548881	0.62745731	-0.6932084	0.11090494	0.76502114
Q64105	Spr	-0.1453077	0.01114651	0.90906256	-0.1008692	0.00523435	0.95376989	-0.1633461	0.00956965	0.95728702
P08032	Spta1 Spna1	-0.0333252	0.00506809	0.68373002	-0.0450451	0.01129349	0.38888484	-0.0440628	0.01409166	0.3397578
P16546	Sptan1 Spna2	-0.2215723	0.00720128	0.94227121	-0.1757699	0.00332174	0.97357443	-0.2459178	0.02077297	0.67655769
Q9R112	Sqrdl	-0.6464306	0.08714216	0.59795039	-0.4042594	0.06992431	0.47461401	-0.483088	0.08118203	0.50291545
P47758	Srprb	-0.2296084	0.00897581	0.98199218	-0.1828832	0.00449598	0.99160986	-0.2238313	0.01029023	0.97325885

Q62093	Srsf2 Pr264 S	-0.2746461	0.0656891	0.59295473	-0.1237333	0.00519644	0.97590243	-0.1998719	0.00984859	0.9694021
Q9CYR0	Ssbp1	-0.2273091	0.09464794	0.32462145	-0.2479537	0.06124619	0.53932448	-0.2493802	0.06605552	0.54290898
Q9CY50	Ssr1	-0.4573346	0.1089533	0.59485808	-0.3827618	0.07923574	0.62502057	-0.4553828	0.07828976	0.72241923
Q9DCF9	Ssr3	-0.1787821	0.07516828	0.65345585	-0.1954108	0.02140533	0.92251495	-0.1353295	0.03350123	0.890817
Q62186	Ssr4	-0.1524796	0.00362998	0.97891788	-0.1265314	0.00247439	0.98345203	-0.1382634	0.00314383	0.97924236
Q9JMD3	Stard10 Pctp	-0.3653127	0.04357581	0.65511171	-0.1657107	0.00400281	0.97496929	-0.3345548	0.04384031	0.59281496
P42225	Stat1	-0.3402906	0.11893241	0.53906501	-0.1688846	0.02312623	0.84209658	-0.3661199	0.32560818	0.17404487
Q923B6	Steap4 Tiarp	-0.6368722	0.06542682	0.70841774	-0.4249996	0.05558688	0.58775926	-0.6017406	0.05995726	0.6864873
P54116	Stom Epb7.2	-0.2164515	0.00985887	0.9757096	-0.1880142	0.01120856	0.95583828	-0.2115576	0.01424249	0.94841832
Q99JB2	Stoml2 Slp2	-0.1592304	0.00515066	0.94651934	-0.1173992	0.00372332	0.93774709	-0.1395657	0.00561242	0.91021256
P50427	Sts	-0.4308165	0.05385358	0.73562119	-0.3365127	0.04232927	0.68546849	-0.3385632	0.03669136	0.75923724
P46978	Stt3a Itm1	-0.1818916	0.01747254	0.90785032	-0.0640003	0.02117115	0.60365959	-0.1199765	0.03812493	0.47376391
Q3TDQ1	Stt3b Simp	-0.1977727	0.00920533	0.97466149	-0.1664742	0.00353791	0.99416284	-0.1886301	0.00469159	0.99263137
Q9Z2I9	Sucla2	-0.1467707	0.00590414	0.98095136	-0.0967278	0.00423997	0.97563022	-0.133015	0.00657472	0.97151706
Q9WUM5	Suclg1	-0.3046085	0.05127325	0.50933786	-0.1893037	0.03785312	0.40993351	-0.3002618	0.06140318	0.38622702
Q9Z2I8	Suclg2	-0.2272683	0.02585638	0.64243472	-0.0993667	0.00880387	0.73470138	-0.1892667	0.02431848	0.55280754
P52840	Sult1a1 St1a1	-0.2597934	0.00806938	0.97829195	-0.2142913	0.00466041	0.98646926	-0.3608291	0.04910689	0.68350675
P52843	Sult2a1 Sta1	-0.3130109	0.07465944	0.59428194	-0.1699729	0.00906805	0.96167989	-0.247127	0.04831268	0.66806954
Q8R086	Suox	-0.1725125	0.00758274	0.90236992	-0.1318275	0.01707734	0.46340955	-0.2003489	0.0295747	0.44601899
Q64310	Surf4 Surf-4	-0.2544879	0.01727533	0.9517567	-0.2652372	0.01946802	0.94405459	-0.2118974	0.01793166	0.92086525
Q9WVA4	Tagln2 Kiaa0	-0.4662717	0.09822474	0.55592725	-0.1274837	0.02472816	0.62422087	-0.4995105	0.10882437	0.51301067
Q93092	Taldo1 Tal Ta	-0.1201928	0.06332606	0.47385028	-0.0877358	0.04354572	0.44808767	-0.0357687	0.04079538	0.13326108
P21958	Tap1 Abcb2	-0.1348225	0.01856171	0.77862409	-0.0742626	0.02386883	0.37694822	-0.1032868	0.02119604	0.61285764
P36371	Tap2 Abcb3	-0.3143017	0.07090525	0.53613792	-0.0295828	0.01963451	0.13952434	-0.1451834	0.05886851	0.25256313
Q9R233	Tapbp Tapa	-0.6440885	0.10393718	0.64647414	-0.5477676	0.07217063	0.75197935	-0.5433481	0.07944221	0.67038933
Q9D0R2	Tars	-0.8036711	0.09423732	0.75188556	-0.6847974	0.09118206	0.68447914	-0.6096632	0.07899113	0.70438482
Q8QZR1	Tat	-0.1643835	0.04014372	0.70548643	-0.2681859	0.0891875	0.50116357	-0.3425685	0.17644813	0.32026549
Q9CY27	Tecr Gpsn2	-0.5617866	0.0783829	0.81063264	-0.2556296	0.00768156	0.98839749	-0.5401618	0.06174787	0.85478929
Q921I1	Tf Trf	-0.805337	0.03157936	0.88201026	-0.4037337	0.02706416	0.70303567	-0.7974235	0.04110044	0.80018316
P82198	Tgfbi	-0.2697644	0.08039367	0.50583277	-0.1758143	0.10921639	0.1561888	-0.3331325	0.13911467	0.32334903
Q9JLF6	Tgm1	-0.1145367	0.00648632	0.91223254	-0.0811845	0.00757527	0.78746022	-0.1080889	0.00630854	0.91577367
P21981	Tgm2	-0.2993611	0.02901751	0.60668418	-0.2737186	0.02796526	0.5813138	-0.2214091	0.01685935	0.69134366
Q62264	Thrsp S14	-0.4647251	0.07955407	0.82978556	-0.3092802	0.03436882	0.89997718	-0.4614286	0.14754057	0.61979754
P62075	Timm13 Tim1	-0.2175905	0.01545543	0.94291328	-0.1365236	0.00814692	0.95251353	-0.2728973	0.02032156	0.93275984
Q9WV98	Timm9 Tim9	-0.1337469	0.00408603	0.98892407	-0.0905549	0.00578118	0.94601952	-0.1328469	0.00712797	0.96392424
P40142	Tkt	-0.2430913	0.05439143	0.32758866	-0.0808935	0.00625837	0.78411082	-0.1695859	0.04494291	0.24448297
P26039	Tln1 Tln	-0.3774969	0.02226147	0.60980064	-0.2657562	0.0171165	0.56053316	-0.4023746	0.02823209	0.54732902
Q9D1D4	Tmed10 Tmp	-0.3758881	0.04215867	0.61866407	-0.2661855	0.02725327	0.62189453	-0.286357	0.0265174	0.68349761
Q9CXE7	Tmed5	-0.2653755	0.03068475	0.92573861	-0.1702557	0.04203862	0.80394421	-0.2778887	0.05620952	0.83016981
Q91XE8	Tmem205	-0.5649338	0.08662702	0.77993483	-0.2162829	0.00584091	0.98989276	-0.675966	0.06741794	0.88549354
Q5F285	Tmem256	-0.1687236	0.00490762	0.99077938	-0.1161924	0.00637181	0.95959935	-0.1623774	0.00292803	0.9957907
Q8VBT0	Tmx1 Txndc	-0.8976504	0.08082802	0.91133194	-0.7363266	0.08545244	0.84135843	-0.8338787	0.05183036	0.95217837
Q921Z5	Tnfaip8	-0.2340845	0.03852082	0.92486452	-0.2160879	0.0273166	0.93991826	-0.1340315	0.06511762	0.45867488
Q9CZW5	Tomm70a D1	-0.7751057	0.08403229	0.79454691	-0.6730547	0.09089376	0.67834447	-0.6501552	0.08899453	0.67242553
P17751	Tpi1 Tpi	-0.1612318	0.00613549	0.93374458	-0.1191961	0.00297077	0.9646463	-0.1762732	0.01584265	0.69628613
O89023	Tpp1 Cln2	-0.172149	0.07063829	0.66439994	-0.0966848	0.01587963	0.86069562	-0.0945861	0.02395984	0.69004992
P63028	Tpt1 Trt	-0.1684986	0.04072379	0.70978084	-0.099666	0.02027629	0.82853872	-0.177601	0.0348847	0.81202503
Q9CQN1	Trap1 Hsp75	-0.7739219	0.07557805	0.86049374	-0.7041789	0.06672219	0.88131474	-0.9063294	0.09470922	0.87568999
Q9DCK3	Tspan4 Tm4s	-0.2075044	0.03017605	0.94034089	-0.274863	0.1271891	0.48294599	NA	NA	NA
P52196	Tst	-0.1298357	0.0097308	0.88558883	-0.0987903	0.00459068	0.95072873	-0.130362	0.00473588	0.96805954
Q8BWP5	Ttpa	-0.3373577	0.07272257	0.4097489	-0.2760289	0.0623616	0.37252595	-0.2041038	0.061858	0.26627151
P07309	Ttr	-0.9007291	0.13498517	0.80189558	-0.1912716	0.01578556	0.91294543	-0.8602449	0.10464362	0.84920849
P68373	Tuba1c Tuba	-0.2263606	0.0246944	0.87503165	-0.5191767	0.06742441	0.80898326	-0.4293081	0.11637014	0.51146003
P68368	Tuba4a Tuba	-0.3987466	0.09109106	0.61491658	-0.1908366	0.00487057	0.99096304	-0.3922397	0.07384451	0.68457423
P99024	Tubb5	-0.2931064	0.06174396	0.50600887	-0.4725464	0.07410618	0.63871234	-0.2500301	0.06483304	0.41459768
Q8BFR5	Tufm	-0.1435288	0.00697391	0.90012106	-0.0892258	0.00601965	0.79688379	-0.1406582	0.01023682	0.79394356
P10639	Txn Txn1	-0.1733002	0.00649895	0.98340406	-0.1323455	0.0046615	0.9829281	-0.2771951	0.06111891	0.61274162
Q91W90	Txndc5 Tlp46	-0.0841395	0.02345329	0.53917806	-0.1115585	0.01827178	0.75647987	-0.0432416	0.00947525	0.63444518
Q9JLT4	Txnrd2 Trxr2	-0.1206819	0.00454331	0.98327685	-0.0861295	0.00571586	0.94192328	-0.1194207	0.00626594	0.9654471
Q02053	Uba1 Sbx Ub	-0.2328233	0.01105261	0.94264283	-0.186371	0.00875848	0.9339924	-0.2140598	0.01004382	0.93998678
P68037	Ube2l3 Ubce	-0.2393217	0.01883068	0.95280851	-0.2163477	0.01675472	0.9487869	-0.2281733	0.02687309	0.96004965
Q8CCJ3	Ufl1 Kiaa077	-0.2552696	0.01679419	0.9352322	-0.3661778	0.06015531	0.61701196	-0.2728046	0.05201562	0.59145522
P61961	Ufm1	-0.5900907	0.06278607	0.88039538	-0.3962678	0.07678342	0.65546418	-0.4990365	0.09024821	0.70167393
O70475	Ugdh	-0.3788965	0.04371343	0.70790443	-0.4530261	0.0542736	0.64708207	-0.5428963	0.07770449	0.63548154
Q6P5E4	Uggt1 Gt Ugg	-0.077852	0.02368413	0.54556902	-0.0933332	0.01332134	0.8169357	-0.0613859	0.02117181	0.45671734
Q91ZJ5	Ugp2	-0.4236868	0.05474422	0.63790555	-0.4517089	0.05856322	0.64321711	-0.4874844	0.0787231	0.6053404

Q63886	Ugt1a1 Ugt1	-0.3338991	0.0260393	0.68963185	-0.2425345	0.0101669	0.87405457	-0.3334151	0.02190117	0.74819108
P70691	Ugt1a2 Ugt1	-0.6023017	0.15966686	0.56400752	-0.6166444	0.16581577	0.4969433	-0.3247102	0.09406279	0.4782616
Q64435	Ugt1a6 Ugt1	-0.2603857	0.01171602	0.96295861	-0.2083777	0.01214025	0.9364292	-0.2849827	0.03464014	0.74636842
Q62452	Ugt1a9 Ugt1	-0.3623105	0.07952688	0.41715027	-0.4495105	0.08548678	0.47143308	-0.3785996	0.0786855	0.41977542
Q8BWQ1	Ugt2a3	-0.2514866	0.0103149	0.87877511	-0.1931462	0.00477875	0.94668499	-0.2752993	0.02237623	0.64585722
Q8R084	Ugt2b1 mCG	-0.3540758	0.06062997	0.66735136	-0.2156467	0.00544094	0.9899172	-0.3354103	0.04639514	0.7438256
P17717	Ugt2b17 Ugt	-0.3898434	0.03477477	0.71538483	-0.2300771	0.02194539	0.67056252	-0.3334473	0.03366114	0.64930597
Q8JZZ0	Ugt3a2	-0.4188358	0.06449888	0.62779904	-0.2063367	0.04653171	0.42138607	-0.2105773	0.03545041	0.61595012
P25688	Uox	-0.6874568	0.03582452	0.78475796	-0.6476317	0.03384689	0.76896377	-0.7184671	0.02539747	0.88012268
Q9D855	Uqcrb	-0.188762	0.00491143	0.98401186	-0.1353488	0.00306133	0.98538112	-0.3527123	0.04466758	0.69782726
Q9CZ13	Uqcrc1	-0.1397589	0.00165926	0.98214204	-0.0990462	0.00125698	0.97595092	-0.1512419	0.00423736	0.90098704
Q9DB77	Uqcrc2	-0.1511708	0.00204922	0.97526883	-0.1027704	0.00118356	0.98011112	-0.1563927	0.00221733	0.9730088
Q9CR68	Uqcrfs1	-0.4015667	0.05036722	0.66515006	-0.2406529	0.05404449	0.33705062	-0.3216683	0.05464606	0.49748484
P99028	Uqcrh	-0.1376377	0.00361824	0.99177539	-0.0956837	0.00446705	0.9703899	-0.1354616	0.00277347	0.99458004
Q9CQ69	Uqcrq	-0.2044946	0.00544683	0.99155843	-0.1465459	0.00618966	0.97563304	-0.4229153	0.06838129	0.74634183
Q8VC12	Uroc1	-0.2298043	0.01109104	0.9147687	-0.1478942	0.00377417	0.97275947	-0.2999428	0.02946751	0.70669932
Q78IK2	Usmg5 Dapit	-0.2017757	0.01051553	0.96843709	-0.1307612	0.00887462	0.93942	-0.1977473	0.01632319	0.91862856
Q62465	Vat1 Vat-1	-0.6364336	0.08930847	0.92698505	-0.1921441	0.04000125	0.79362412	-0.1225397	0.01389996	0.95105168
Q64727	Vcl	-0.1489117	0.00375706	0.97698928	-0.1200795	0.00422654	0.95054016	-0.1450465	0.00494491	0.95663755
Q01853	Vcp	-0.4071523	0.03963455	0.57187966	-0.3061469	0.03364921	0.48188662	-0.3568048	0.03562051	0.52993879
Q60932	Vdac1 Vdac5	-0.2230029	0.01686339	0.62483449	-0.1213292	0.00856572	0.6180304	-0.2649888	0.02221328	0.56179837
Q60930	Vdac2 Vdac6	-0.1838209	0.00442094	0.97134611	-0.1199472	0.00229296	0.97889425	-0.18526	0.00353211	0.98039933
Q60931	Vdac3	-0.1488607	0.00580169	0.94815239	-0.0968355	0.00292072	0.96151259	-0.1956256	0.02521806	0.60676226
P20152	Vim	-0.1389728	0.01859321	0.7666964	-0.1454561	0.02553469	0.66975651	-0.1158768	0.01699296	0.74400086
Q9CRC0	Vkorc1	-0.2399008	0.0155779	0.95183862	-0.1679413	0.00438837	0.99053135	-0.1939701	0.00426007	0.99376851
P29788	Vtn	-0.9531406	0.10144959	0.88919115	-0.6509201	0.10797736	0.72189346	-0.7161041	0.16527373	0.59085368
Q8CC88	Vwa8 Kiaa05	-0.4122246	0.11779733	0.52680185	-0.5316177	0.10552637	0.61333174	-0.4978698	0.1140407	0.55959442
Q00519	Xdh	-0.2405723	0.01788235	0.9476398	-0.1950953	0.0145998	0.93213841	-0.223429	0.02452945	0.87363982
Q3TNA1	Xylb	-0.1499173	0.01215425	0.92689231	-0.1249076	0.00524853	0.97587762	-0.2807686	0.06061566	0.6226957
Q9CQV8	Ywhab	-0.1234518	0.01858406	0.80046406	-0.095654	0.01239681	0.85619137	-0.0861127	0.02209266	0.65506553
P62259	Ywhae	-0.2383422	0.03071625	0.75065314	-0.1589098	0.01267876	0.8870629	-0.2553014	0.01429317	0.94379412
P61982	Ywhag	-0.2234149	0.0071059	0.99097764	-0.1725742	0.00792102	0.97334249	-0.2273259	0.00657642	0.99170032
P68254	Ywhaq	-0.2045109	0.03723975	0.75099085	-0.2616107	0.01367369	0.98919063	-0.6859073	0.0820131	0.89736517
P63101	Ywhaz	-0.2148681	0.01404852	0.87966676	-0.1441331	0.00743207	0.9170942	-0.2721409	0.04067811	0.59870264
Q8BGC4	Zadh2	-0.0475653	0.02260728	0.32969604	-0.0191547	0.00988756	0.23823702	-0.01228	0.005227	0.3556468
Q80W54	Zmpste24 Fa	-0.4904078	0.07032089	0.8020934	-0.5088605	0.08900142	0.7001444	-0.5412243	0.10371385	0.67687487
Q91V76		-0.8150998	0.10775899	0.83874659	-0.5997761	0.06674415	0.8613359	-0.7200188	0.07247338	0.89972933
P01837		-0.4022363	0.04929254	0.72704031	-0.237899	0.0315123	0.6950976	-0.4591304	0.07713152	0.5767749
Q9CQE8		-0.2476023	0.00700373	0.99049001	-0.2089579	0.00974329	0.98290399	-0.2190272	0.01232787	0.96044547
Q9DCS2		-0.2231953	0.00749602	0.99105708	-0.2385912	0.01976099	0.95418185	-0.224113	0.01007847	0.98212424
P03987		-0.1703023	0.02881516	0.79512882	-0.1625108	0.01507113	0.90644832	-0.1519512	0.02366108	0.82086678
P01864		-0.1297974	0.01318567	0.89805466	-0.1058655	0.01618609	0.76693502	-0.0638201	0.02311301	0.36967659
P18525		-0.1264107	0.03238821	0.83546594	-0.2158115	0.22271795	0.19010996	-0.1255692	0.02994093	0.85428961
P04945		-0.1001177	0.03015184	0.61165895	-0.0968642	0.02320945	0.65932223	-0.0501139	0.05482101	0.07712004
Q3UNZ8		-0.0260604	0.02174085	0.22322186	-0.1592594	0.02670344	0.89891162	-0.1433993	0.01293136	0.96092891
