# Supplementary material for: Subacute calorie restriction and rapamycin discordantly alter mouse liver proteome homeostasis and reverse aging effects
Source: Aging Cell. 2015 Mar 23;14(4):547–57. doi: 10.1111/acel.12317 (PMC4531069; doi:10.1111/acel.12317)
Supplement: Supplementary file 3 [file acel0014-0547-sd3.pdf]

Figure S1. Immunoblot analysis of mTOR downstream targets. (A, D) S6 phosphorylation was significantly decreased by CR in young and old livers and ORP. (C) Phosphorylation of 4ebp1 significantly decreased with YRP but no change was detected with aging or in other treatment groups. (B, E) eEF2 phosphorylation increased significantly with young and old rapamycin treatments. Significance tested with unpaired t-test. (n=4-6 per groups). * $p<0.05$, ** $p<0.001$, *** $p<0.0001$

Figure S2. Topograph calculation of protein turnover rate and peak alignment for isotopomer abundance. (A) An example of the calculation of peptide turnover rate, as described previously (Hsieh *et al.* 2012). Topograph calculates % newly synthesized peptide from AUC of labeled peptide isotopologues. The % new synthesis for all peptides uniquely matching to Carbamoyl-phosphate synthase (uniprot ID: Q8C196) plotted over sampling time is shown. As shown, synthesis is greatly reduced in OCR (pink symbol) compared to OCL (brown symbol). (B) A logarithamic transformation of the equation for exponential decay allows the determination of the protein turnover rate (slope) by linear regression. Carbamoyl-phosphate synthase turnover rate is greatly reduced by OCR (smaller slope) compared to OCL (larger slope). (C) Topograph realigns the MS/MS chromatograms of peptide ions from different samples. For two given MS/MS chromatograms, the MS/MS scan number for peptides identified in both samples were plotted against each other in a scatter plot. A LOESS regression was used to find the best fit line through the data

points. For peptides that were identified in one sample, we used the identified peptide's MS/MS scan number and the LOESS regression to identify the corresponding retention time in the other samples.

Figure S3. Body weights of young (A) and old (B) mouse cohorts over the course of the experiment. Both young and old groups were acclimated to the synthetic chow for 3 weeks. Mice were individually housed and CR or RP started a week later. After 10 weeks on the treatments, mice were switched to a $^{3}H_{2}$-leucine diet and n=3 mice were euthanized at 3, 7, 12, and 17 days following start of labeled diet.

Figure S4. Linear regression fits of peptide abundance (peptide isotopomer area) over the labeling period indicate no significant change in abundance. For each identified peptide, the consistency of abundances over the labeling period of 17 days was assessed, as shown by representative plots in (A). The sum of all isotopic peak areas of a peptide was plotted vs. time, and an orthogonal regression was performed to determine whether the abundance was either increasing or decreasing over time. For each treatment group, the slopes of these peptide regressions were plotted as a histogram, shown in (B), to determine whether the distribution of slopes was different from zero (no overall changes in abundance), greater than zero (increasing abundances), or less than zero (decreasing abundances). The p-value and correlation coefficient for these estimates are also reported.

Figure S5. Liver total and mitochondrial protein turnover slope correlation. Protein turnover rates calculated for the same proteins in the total and mitochondrial protein fractions were correlated with linear regression analysis. All $R^2 > 0.83$ and slopes are > 0.88 indicating the high degree of correlation among the measured turnover rates.

Figure S6. Heatmap of mitochondrial protein half-life log_2 ratios. BCAA, brached-chain amino acid degradation, TCA, tricarboxylic acid, FAO, fatty acid beta oxidation. Red indicates longer half-life in the numerator and blue in the denominator. The component protein ID's and half-life log2 ratios are listed in Table S4.

Figure S7. Mitochondrial aconitase and citrate synthase activity in liver. (A) Aconitase activity was significantly decreased with rapamycin treatment in old mice (p = 0.03). (B) Citrate synthase activity was not significantly affected by age or treatments.

Figure S8. The ratios of mitochondrial DNA-to-nuclear DNA (mtDNA/nDNA) did not alter with treatment. (A) The relative mt DNA copy number was determined by quantifying the ratio of ND1 (mtDNA-encoded) to Cytochrome P450 1A1 (nuclear gene). (B-D) Mitochondrial biogenesis markers (B) Nrf1, (C) Nrf2, and (D) downstream mt transcription factor A (TFAM) are unchanged by the treatments. n = 4-9.

Figure S9. Mitochondrial deletion frequency in mouse liver. Deletion frequency was assayed in n=12 mice in each group. The deletion load increased 100-fold with aging but no change was observed with the sub-acute treatments.

Figure S10. Global protein translation increased with aging and linear trend analysis of CR and RP effects on polysome loading. (A,B) Polysome loading with sub-acute CR and RP are plotted as a fraction of control for each ribosome/ polysome peak. (C) Polysome loading in old control as a fraction of young control. (n=5-7 per group)

Figure S11. Rapamycin blood level measurements from n=11-17 mice from the two age groups. Rapamcin blood levels were tested 4 weeks after the start of the treatment and at the time of euthanasia. There was no significant difference in the level of rapamycin in the blood between the two time points. The rapamycin blood concentration of young mice averaged 76 $\pm$ 8 ng/ul and old mice averaged 44 $\pm$ 4 ng/ul.

Figure S12. Precursor pool enrichment of ^{3}H-leucine over time and days until initial appearance of label. (A) The number of days taken for the label to appear in the liver was significantly shorter in the young cohorts compared to the old cohorts indicating either reduced absorption in the gut or reduced catabolism in the old mice. (B) Each successive column of a single color indicates the days

of euthanization from the start of $^{3}H_2$-Leucine containing diet (i.e. 3, 7, 12, and 17 days).

Figure S13. Relative standard error of the half-life estimates in Liver and Heart. (A) Liver RSE's are slightly higher for low half-lives (<3 days), lower in the middle range (3-11 days) and high or variable for the long half-lives (16-24 days). (B) Heart RSE's are slightly higher for the low half-lives (<3 days) and relatively low for the rest of the half-life ranges.

References

Hsieh EJ, Shulman NJ, Dai DF, Vincow ES, Karunadharma PP, Pallanck L, Rabinovitch PS , MacCoss MJ (2012). Topograph, a software platform for precursor enrichment corrected global protein turnover measurements. *Mol Cell Proteomics*. **11**, 1468-1474.
